# Peptide and Protein Cysteine Modification Enabled by Hydrosulfuration of Ynamide

**DOI:** 10.1021/acscentsci.4c01148

**Published:** 2024-08-21

**Authors:** Changliu Wang, Zhenguang Zhao, Reem Ghadir, Dechun Yang, Zhenjia Zhang, Zhe Ding, Yuan Cao, Yuqing Li, Rosi Fassler, Dana Reichmann, Yujie Zhang, Yongli Zhao, Can Liu, Xiaobao Bi, Norman Metanis, Junfeng Zhao

**Affiliations:** † Affiliated Cancer Hospital, Guangdong Provincial Key Laboratory of Major Obstetric Diseases, School of Pharmaceutical Sciences, Guangzhou Medical University, Guangzhou 511436, Guangdong P. R. China; ‡ Institute of Chemistry, The Hebrew University of Jerusalem, Jerusalem 9190401, Israel; § Collaborative Innovation Center of Yangtze River Delta Region Green Pharmaceuticals & College of Pharmaceutical Sciences, Zhejiang University of Technology, Hangzhou 310014, Zhejiang P. R. China; ∥ National Research Center for Carbohydrate Synthesis, College of Chemistry and Chemical Engineering, Jiangxi Normal University, Nanchang 330022, Jiangxi P. R. China; ⊥ Department of Process Development, BeiGene Guangzhou Biologics Manufacturing Co., Ltd., Guangzhou 510700, Guangdong P. R. China; # The Alexander Silberman Institute of Life Science, ^@^The Center for Nanoscience and Nanotechnology, The Hebrew University of Jerusalem, Jerusalem 9190401, Israel; □ Institute of Chemistry, The Alexander Silberman Institute of Life Science, The Center for Nanoscience and Nanotechnology, Casali Center for Applied Chemistry, The Hebrew University of Jerusalem, Jerusalem 9190401, Israel

## Abstract

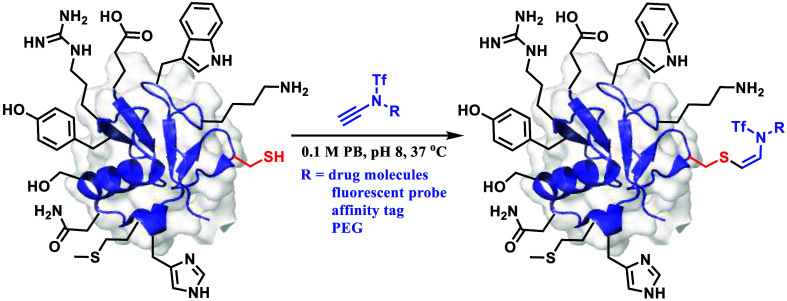

Efficient functionalization of peptides and proteins has widespread applications in chemical biology and drug discovery. However, the chemoselective and site-selective modification of proteins remains a daunting task. Herein, a highly efficient chemo-, regio-, and stereoselective hydrosulfuration of ynamide was identified as an efficient method for the precise modification of peptides and proteins by uniquely targeting the thiol group of cysteine (Cys) residues. This novel method could be facilely operated in aqueous buffer and was fully compatible with a wide range of proteins, including small model proteins and large full-length antibodies, without compromising their integrity and functions. Importantly, this reaction provides the *Z*-isomer of the corresponding conjugates exclusively with superior stability, offering a precise approach to peptide and protein therapeutics. The potential application of this method in peptide and protein chemical biology was further exemplified by Cys-bioconjugation with a variety of ynamide-bearing functional molecules such as small molecule drugs, fluorescent/affinity tags, and PEG polymers. It also proved efficient in redox proteomic analysis through Cys-alkenylation. Overall, this study provides a novel bioorthogonal tool for Cys-specific functionalization, which will find broad applications in the synthesis of peptide/protein conjugates.

## Abstract 2

Highly regio- and stereoselective hydrosulfuration of ynamide bearing a strong electron-withdrawing group has been identified as an efficient tool for peptide and protein cysteine modification.

## Introduction

**Figure 1:**
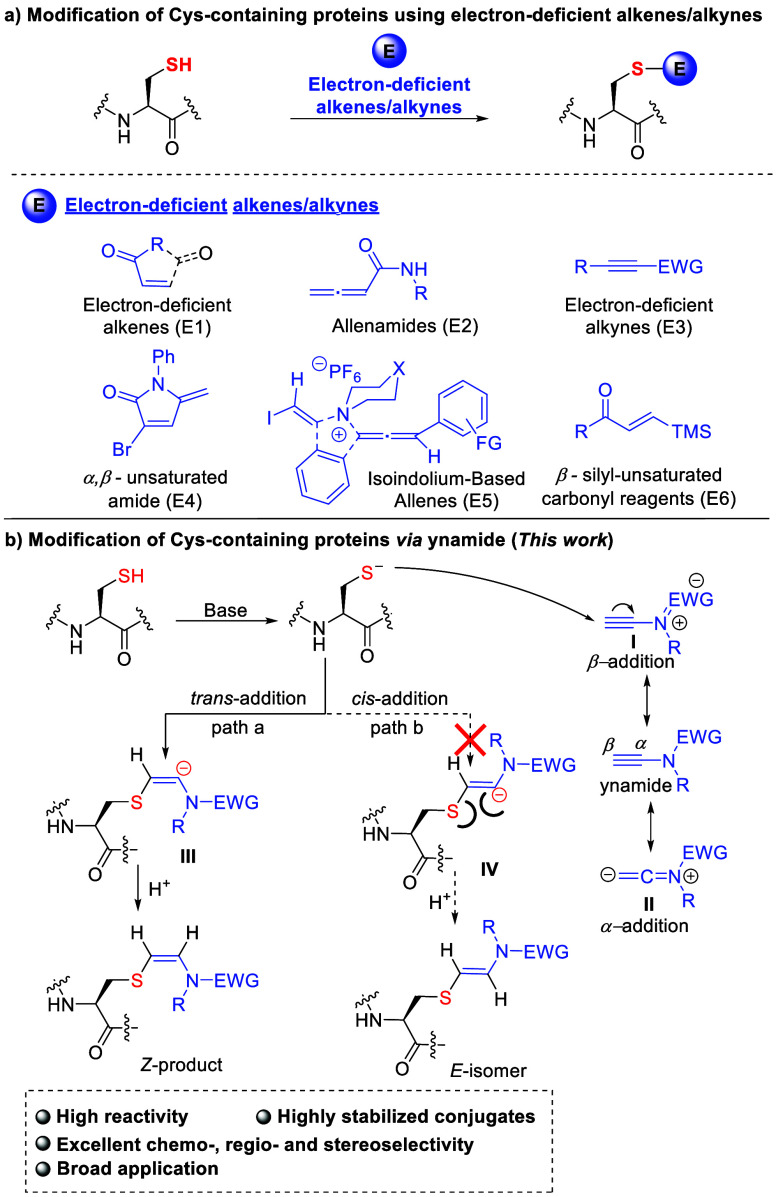
Cys-specific modifications with electron-deficient alkenes/alkynes.

Selective chemical protein functionalization is a cornerstone of chemical biology, which enables the precision engineering of target biomolecules with different purposes, in turn facilitating the investigation of biological processes^1^ and the development of targeted therapeutics.^2^ In recent years, a wide variety of protein bioconjugation chemistries,^3^ including bioorthogonal click chemistry recognized by the 2022 Nobel Prize in Chemistry,^4^ have been developed and used widely in a broad range of applications, such as fluorescent labeling,^5^ conjugate-based therapeutics,^6^ and activity-based chemical proteomic strategies.^7^ With time, the advanced exponential impact of precise protein functionalization led to increased attention focused on novel efficient bioconjugation methods. Genetic code expansion is a unique technique to achieve site-selective protein modification by incorporating a designed amino acid into the recombinant target protein.^8^ Alternatively, direct conjugation enables the chemical attachment of designed molecules to predetermined sites of the native proteins without preinstalled functional groups.^9^ The challenges associated with their development are manifold, which is caused by the requirement of high efficiency, unique reactivity, excellent selectivity, and mild aqueous conditions.^10^ Among the developed chemoselective protein modifications,^11^ cysteine (Cys)-based strategies,^12^ including N-terminal^13^ or C-terminal^13b^ specific approaches, are particularly attractive and have been widely applied for a diverse array of functionalizations of native proteins and the generation of protein conjugates, due to its unique nucleophilicity, solvent exposure, and low natural abundance. This is especially true for those in the reduced form on the protein surfaces, which are readily accessible for chemical modifications.^14^ Various Cys-specific modifications have been developed, including Michael addition,^15^ hypervalent iodine-mediated reactions,^16^ metal-mediated reactions,^17^ nucleophilic substitution,^18^ umpolung of thiols,^19^ radical transformations,^20^ desulfurative coupling,^21^ and disulfide rebridging.^22^ Among these strategies, Michael addition with maleimides is the most widely used to modify the proteins of interest both in vivo and in vitro.^23^ Although the rapid kinetics of these reactions has attracted considerable attention,^24^ two major drawbacks including the low chemoselectivity (for details, see Figures S66–S76 in Supporting Information) and poor stability of the conjugated products in the presence of external thiols (like glutathione) plagued their applications (Figure 1a).^25^ Additionally, a diversity of compounds based on a cyclic 1,3-dicarbonyl scaffold were developed to probe the cysteine sulfenic acid (Cys-SOH), which could also be used for the Cys labeling but in the presence of oxidants.^26^ Meanwhile, Cys-specific protein modification techniques have been adapted for chemoproteomic cysteine profiling, which enables the characterization of reactive hotspots of interest and provides insights for the discovery of new druggable sites.^27^ Cys-alkylation with iodoacetamide is one critical step of sample preparation for bottom-up proteomic analysis;^28^ however, nonspecific alkylation at other residues rather than Cys was detected in many cases.^29^ Recent progresses employing innovative Michael acceptors,^30^ such as allenamide,^31^ vinylheteroarenes,^32^ phosphorus-containing electron-deficient alkynes,^33^ and isoindolium-based allenes,^34^ provide promising results. Especially, the introduction of silicon substituents at the β-position of α,β-unsaturated carbonyl compounds developed by Loh’s group significantly improves chemoselectivity and avoids retro-Michael addition^35^ (Figure 1a). Despite significant advancements, complex biological systems still present challenges in achieving stereo- and regioselectivity for the synthesis of a precise isomer of the conjugated products,^36^ which is crucial for chemoproteomic analysis and drug discovery.^37^ Moreover, ensuring the stability of these products is also an ongoing challenge (Figure 1a).

Ynamides, characterized by their strong polarization and the presence of an electron-donating nitrogen atom attached directly to the alkyne C–C triple bond, offer an optimal balance between stability and reactivity.^38^ This balance is further tuned by an electron-withdrawing group (EWG) on the nitrogen, allowing for selective chemical transformations at the α- or β-position via two polarized resonance structures (Figure 1b, I and II).^38^ We recently have disclosed that ynamides could be used as a class of novel general coupling reagents for amide^39^ and ester^40^ bond formation, exploiting the protonation-initiated α-addition of ynamides with carboxylic acids, which has also been applied to the carboxyl residues profiling in live cells.^41^ However, these active ester conjugates are susceptible when exposed to competing nucleophiles, such as amino and hydroxyl groups, to rerelease the ynamide in its hydrolyzed form.^39^ On the other hand, the radical hydrothiolations of internal^42^ and terminal^43^ ynamides through β-addition suffered harsh and bioincompatible conditions. Intriguingly, base-promoted β-hydroamidation^44^ and β-hydrophosphorylation^45^ of ynamides via “umpolung” chemistry exclusively afforded the stable *Z*-isomer of the corresponding conjugated products. This “umpolung” chemistry inspired us to explore the reactivity of ynamide as a Michael addition acceptor for the thiol group of cysteine. Here, we report a highly chemo-, regio-, and stereoselective β-hydrosulfuration of ynamide for Cys-specific functionalization of peptides and proteins. The excellent chemo- and regioselectivity was governed by the basic reaction conditions and the designer ynamide bearing a strong electron-withdrawing group, which suppressed the protonation-initiated α-addition (Figure 1b).

## Results and Discussion

### Rational Design of a Biocompatible β-Hydrosulfuration of Ynamide

**Figure 2:**
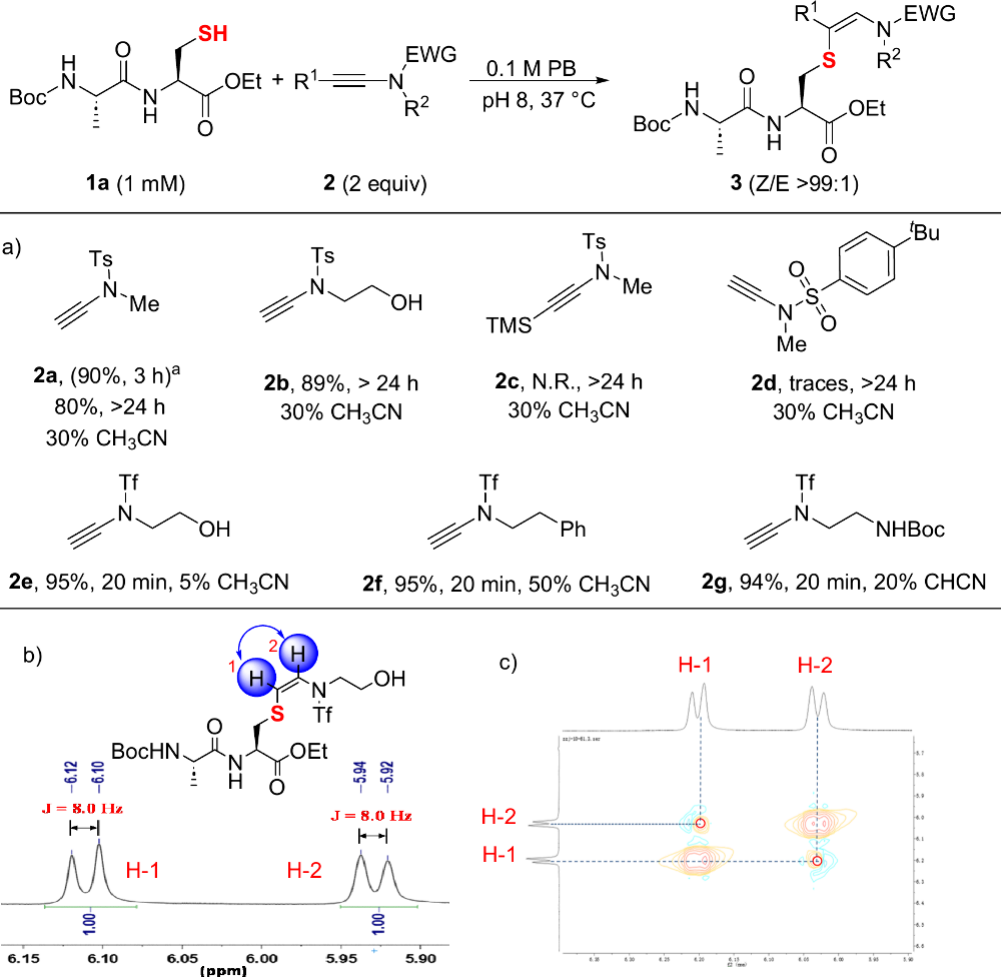
Rational design of ynamides for cysteine modification. a) The stereoselectivity of all reactions was determined by ^1^H NMR, and the yields presented were isolated yields. PB = phosphate buffer. ^*a*^MeCN was the solvent. b) Zoom-in of ^1^H NMR spectra (the protons of the characteristic alkene moiety are colored in blue) for the crude product of **1a** and **2e**. c) Zoom-in of the characteristic alkene moiety of ^1^H–^1^H NOESY spectrum for the crude product of **1a** and **2e**.

Our investigation into ynamide chemistry was initiated with the insight that the presence of an electron-withdrawing group (EWG) on the nitrogen atom played a pivotal role in facilitating β-addition, as demonstrated by the resonance structure I (Figure 1b). This understanding guided the rational design of ynamides tailored for selective β-hydrosulfuration with cysteine under basic reaction conditions, to circumvent the competing protonation-initiated α-carboxylation, particularly for peptides that possess a carboxylic group. To this end, *N*-methylynetoluenesulfonamide (MYTsA, **2a**), the first-generation ynamide coupling reagent for peptide synthesis,^39^ was tested in our model reaction as a reference. As shown in Figure 2a, the β-hydrosulfuration of **2a** (2 equiv) with a model dipeptide Boc-Ala-Cys-OEt (**1a**, 1 mM) proceeded smoothly in acetonitrile (MeCN) using Na_2_CO_3_ (2 equiv) as a base to give the conjugate product (**3aa**) in 90% yield with excellent stereoselectivity (*Z*:*E* > 99:1). Further optimization was performed to implement this chemistry under biocompatible conditions, such as aqueous media, near-neutral pH (6–8), and physiological temperature. In parallel, the poor water-solubility of **2a** led to a sluggish reaction in an MeCN/phosphate buffer mixture (pH 8, 3:7), giving **3aa** in 80% yield over 24 h. Enhancing the solubility by replacing the methyl group of R^2^ with a 2-hydroxyethyl group (**2b**) led to a more efficient reaction, with an 89% yield of **3ab**. The subsequent evaluation of internal ynamide (**2c**) revealed no reactivity under identical conditions, which suggests the R^1^ substituent’s crucial role. Substituting the *p*-toluenesulfonyl (Ts) group of **2a** with a trifluoromethylsulfonyl (triflyl, Tf, **2e**) group enhanced both the solubility in water and the reaction efficiency due to its stronger electron-withdrawing ability and increased hydrophilicity. This modification facilitated the reaction to completion within 20 min in phosphate buffer with minimal MeCN (5%), yielding the product (**3ae**) as a single *Z*-isomer in 95% yield, confirmed by NMR (*Z*:*E* > 99:1; see zoom-in ^1^H NMR spectra in Figure 2b and details in SI). Further exploration with ynamides (**2f**) and (**2g**), featuring varied functional groups, showed comparable efficiency. This underscores the versatility of ynamide functionalization via R^2^ group modification, promising broad applicability in synthesizing diverse conjugates. The exclusive formation of the *Z*-isomer might be attributed to a low-energy transition state of *trans*-addition, whereas the formation of the *E*-isomer was discouraged due to an unfavorable charge repulsion (Figure 1b), a conclusion supported by DFT studies by Hentzet al.^44,46^

In line with our proposal, the conjugation of the optimal ynamide (**2e**) and peptide (**1a**) was sensitive to pH and was sluggish under neutral conditions (pH 6–7.5, Figure S14). Furthermore, employing 5 equiv of **2e** resulted in a substantial improvement, with a quantitative conversion within 5 min. The apparent second-order rate constant for the thiol conjugation with the hydrosulfuration of glutathione (GSH) to ynamide (**2e**) as the model reaction was found to be 0.2399 ± 0.002 M^–1^ s^–1^ (Figure S64 in SI). To further dissect the reaction mechanism, the reaction was carried out in the presence of different types of radical scavengers; no negative effect on the reaction efficacy was observed. In addition, the thiyl radical chemistry of ynamide was examined in aqueous medium, but no reaction was detected (see the SI for details, Schemes S15–S16). These results excluded a radical mechanism for this transformation.

### Chemoselectivity Study

**Scheme 1:**
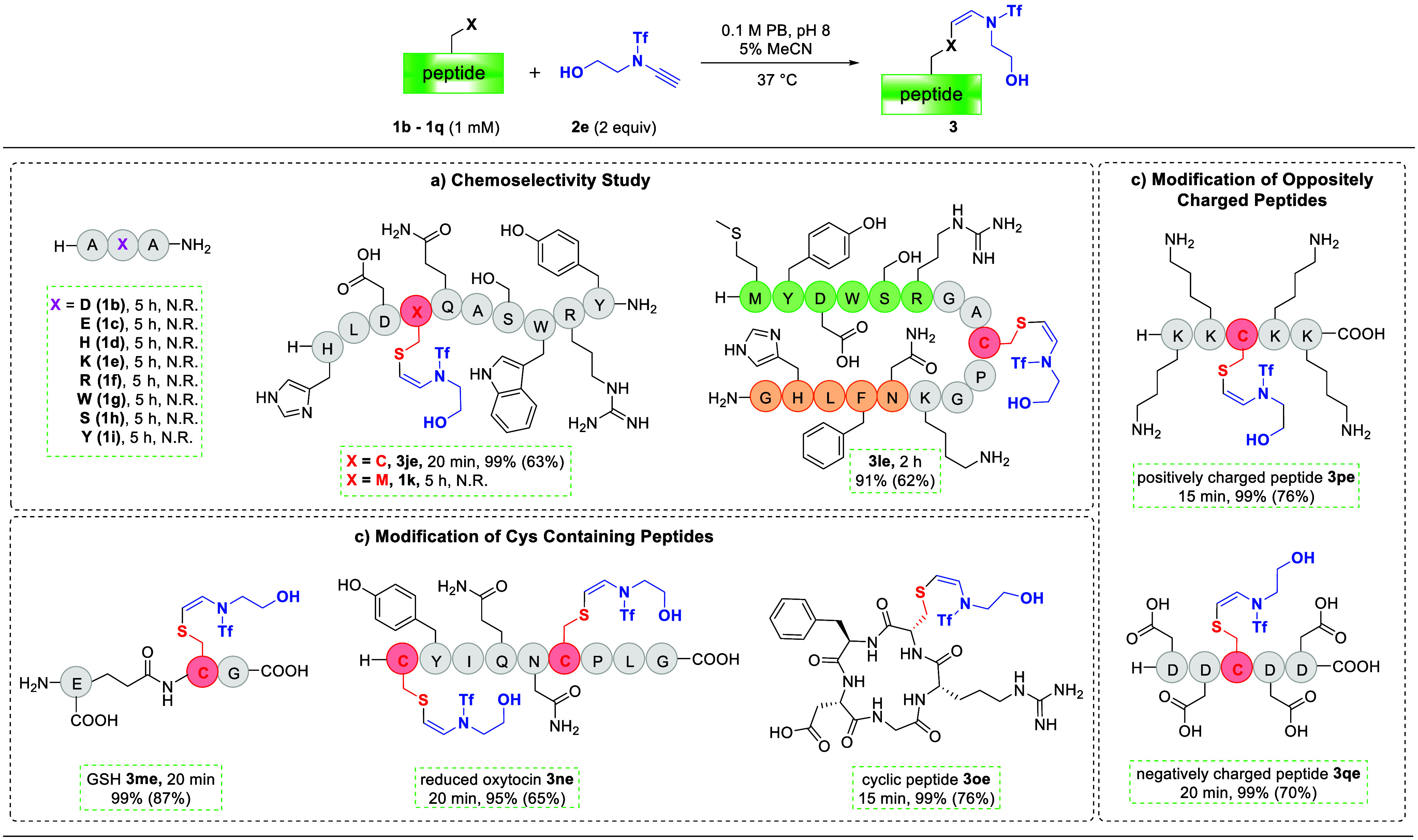
Systematic Study of Cys Modification by Ynamide in the Presence of Other Residues Reaction conditions: peptides (**1b**–**1q**, 1 mM), ynamide (**2e**, 2 equiv), in 0.1 M PB containing 5% MeCN, pH 8, at 37 °C. All yields presented were determined by integrated areas of HPLC peaks (at 220 nm), whereas isolated yields are in parentheses. GSH = reduced glutathione; N.R. = no reaction.

With the optimized reaction conditions in hand, we moved forward to investigate the chemoselectivity of this strategy in the presence of other unprotected amino acid side chains (Scheme 1a). When tripeptides containing Asp and Glu (**1b** and **1c**) were treated with **2e** under the standard reaction conditions, no reaction could be detected even after extended reaction times (5 h), indicating that the normal α-addition of carboxylic acids to ynamides was completely suppressed.^39^ This was attributed to the fact that the carboxyl group was deprotonated under basic reaction conditions and thus prohibited protonation-initiated α-addition. Furthermore, considering that the potential β-hydroamidation of ynamides with indole^46^ and amide^44^ in the presence of strong base, tripeptides containing histidine (His, **1d**), lysine (Lys, **1e**), arginine (Arg, **1f**), and tryptophan (Trp, **1g**) were investigated. Interestingly, all of these side chains were kept intact. The unprotected nucleophilic hydroxyl group of serine (Ser, **1h**) and tyrosine (Tyr, **1i**) was also compatible under optimized reaction conditions. Next, the chemoselective modification of Cys was performed in the presence of all possible reactive side chains (**1j** and **1l**). Notably, the reactions of **1j** and **1l** were completed within 20 min and 2 h, respectively, providing a single conjugated product in excellent yields, and the modification site was confirmed to be on the Cys residue by trypsin digestion of **3le** (Figure S29). As expected, no modification could be detected when Cys was substituted with Met (**1k**). All the chemoselectivity studies toward the side chain functional groups in peptides demonstrated that the base-promoted β-addition of ynamide was orthogonal to the thiol group of Cys.

### The Scope of Peptides in the Reaction

The robustness of this methodology was further evaluated with complex peptide substrates (**1m**–**1q**, Scheme 1b and 1c). Reduced glutathione (GSH, **1m**) could be modified by ynamide **2e** in excellent yield under the standard reaction conditions. The reduced oxytocin (**1n**), containing one terminal and one internal Cys residue, could be bis-functionalized in excellent yield within 20 min, while oxidized oxytocin was inert, indicating that the position of Cys in the sequence did not affect the efficiency of the modification. Moreover, a potent αVβ3 integrin-binding cyclic peptide^18g^ (**1o**) was employed in this transformation to provide the desired Cys-specific modified product in 76% yield. Finally, two peptides with highly positively and negatively charged sequences, **1p** and **1q**, respectively, were selected as key examples to evaluate the influence of the electrostatic effect on this transformation. The Cys-specific modifications of both **1p** and **1q** proceeded smoothly under the standard conditions, illustrating that highly charged peptides did not have a negative effect on the reaction.

### Ynamide-Based Peptide Modification Places Ynamide as a Biologically Relevant Molecule

**Scheme 2:**
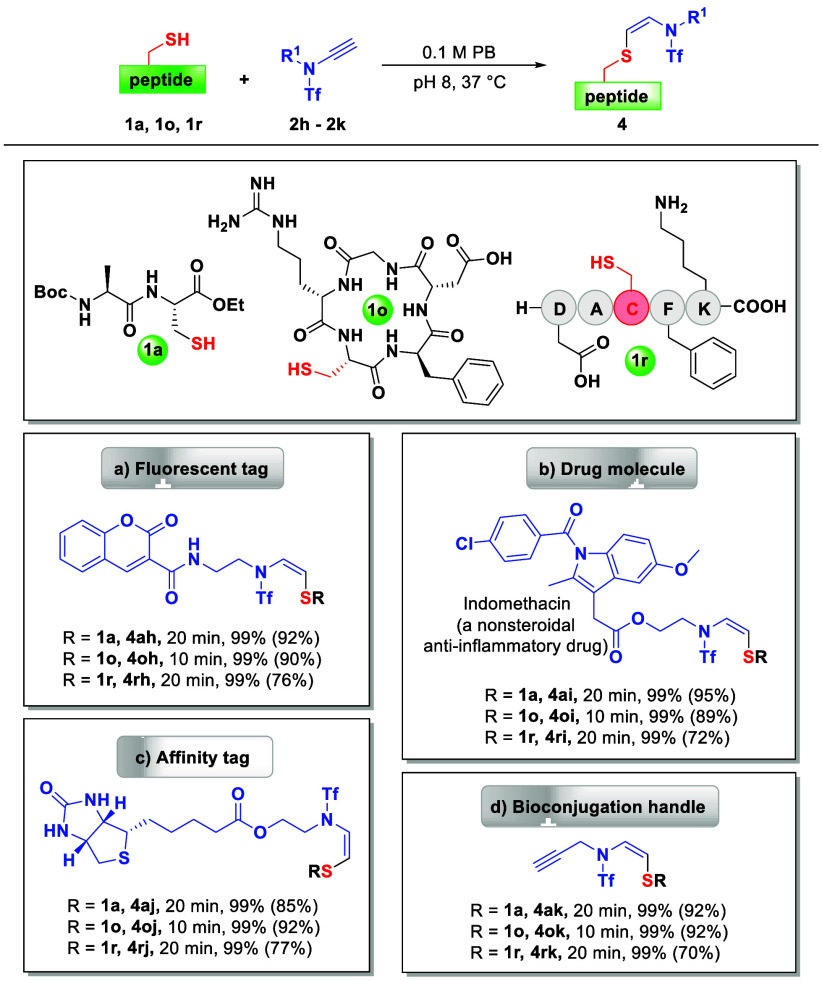
Selective Cys Modification of Peptides with Ynamides Containing Various Biologically Relevant Tags and Probes Reaction condition: peptides **1a** (1 mM)/**1o** (0.5 mM)/**1r** (1 mM), and ynamides **2h**–**2k** (2 equiv), 100 mM PB, pH 8, 37 °C; a–b) 50% MeCN; c) 5% MeCN; (D) 10% DMSO. All yields presented were determined by integrated areas of HPLC peaks (at 220 nm), whereas isolated yields were in parentheses.

To demonstrate the applicability of this strategy in peptide and protein chemical biology, different biologically relevant functional moieties, including fluorescent tag (coumarin, **2h**), complex drug molecule (indomethacin, **2i**), affinity tag (biotin, **2j**), and alkyne handle for bioconjugation (**2k**), were introduced to ynamides and evaluated by employing the Cys-containing peptides (Scheme 2). The Cys-selective modification of protected peptide (**1a**), cyclic peptide (**1o**), and unprotected peptide (**1r**) with a variety of important ynamide-based functional molecules (**2h**–**2k**) proceeded smoothly in buffer with MeCN as the cosolvent (for solubility reason) to afford the desired products in excellent yields (Scheme 2).

### One-Pot Dual Functionalization of Cys with Click Chemistry

**Scheme 3:**
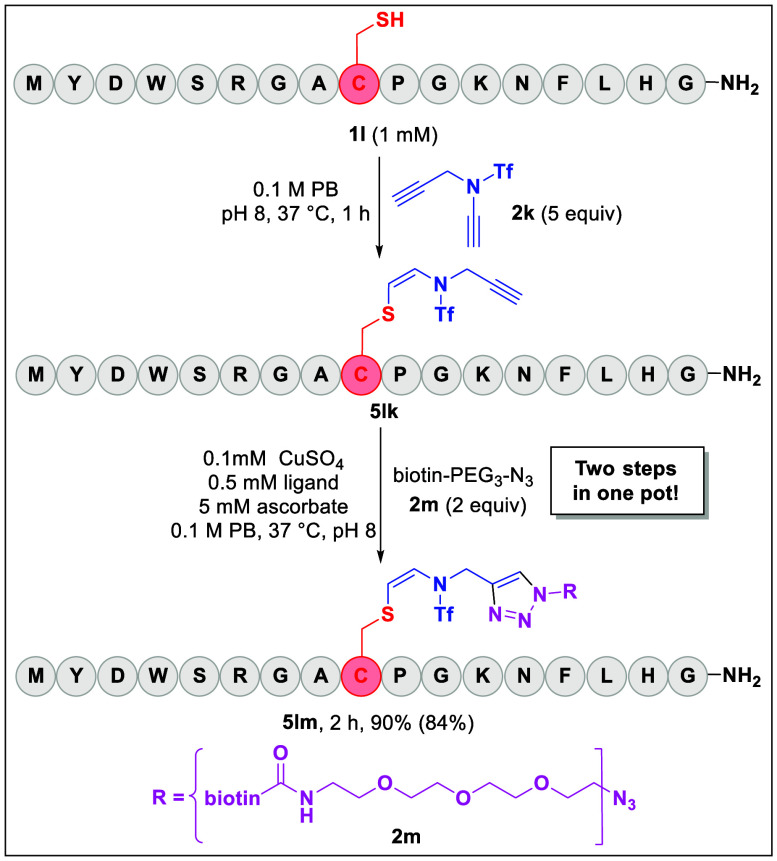
Cys-Specific Dual Functionalization Reaction conditions: 1) Peptide **1l** (1 mM) and ynamide **2k** (5 equiv) were incubated in 100 mM PB, pH 8, at 37 °C for 1 h. 2) Azide **2m** (2 equiv), CuSO_4_ (0.1 mM), ligand (bathophenan-throlinedisulfonic acid disodium salt, 0.5 mM), and sodium ascorbate (5 equiv) were added to the reaction, which was incubated at 37 °C for 30 min

Considering the great importance of click reactions,^47^ the compatibility of our Cys-specific modification with a click reaction was studied. As shown in Scheme 3, the Cys of peptide **1l** was first modified efficiently by ynamide **2k** to give the *Z*-conjugated product **5lk** containing an additional C–C triple bond, which readily took part in the proceeding click reaction with biotin-PEG_3_-azide (**2m**) to anchor a biotin tag to the peptide in a one-pot manner to give the target dual functionalized product (**5lm**) in 90% yield (Scheme 3). The excellent efficiency of such a one-pot, two-step dual functionalization unambiguously illustrated the potential application and flexibility of our strategy to anchor a biotin tag or other useful functional groups to a peptide of interest.

### Stability Study of the Peptide Conjugates

The stability of the conjugated products is of the utmost importance for developing a powerful peptide and protein modification strategy.^6^ No deterioration was observed when the conjugated product **3ae** (1 mM) was incubated in buffer solutions with pH 2, 8, 10, and 13, respectively, for 10 h (Figure S57). Furthermore, the ynamide-modified peptide, **3ae** (10 μM), did not undergo β-syn elimination, which was commonly encountered in certain methods when treated with a large excess of H_2_O_2_ (at pH 4, 8, and 10 at 37 °C), even after extended time (24 h, Figure S59). Further, **3ae** remained intact after 10 h of incubation in bovine serum (Figure S60). Moreover, the cleavage of **3ae** in the presence of external thiol to regenerate the unmodified **1a**, which occurred in other methods,^16b,33a^ was not detected in our hands (using 2-mercaptoethanol, Figure S58). In contrast, the iodoacetamide (IAM) peptide conjugate decomposed under the aforementioned conditions (Figures S61–S63). The superior stability of the modified products further enhances the application potential in the construction of peptide and protein conjugations.

### Modification of Cys-Containing Proteins and Antibody

**Figure 3:**
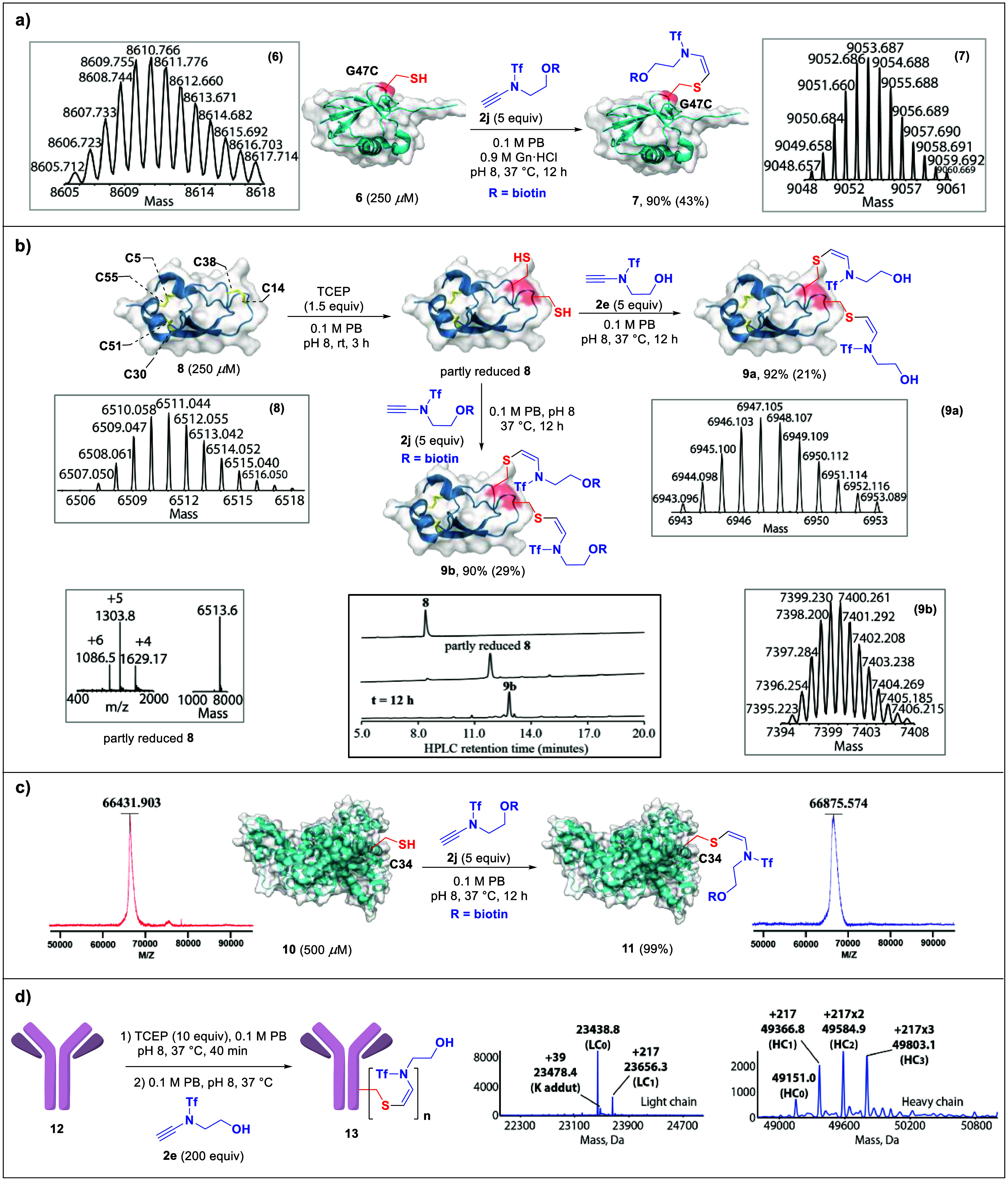
Chemoselective modification of ubiquitin variant, partly reduced BPTI, BSA, and partly reduced antibody trastuzumab. a) Ubiquitin(G47C) (**6**, 250 μM) and **2j** (5 equiv) were incubated in 100 mM PB (containing 0.9 M Gn·HCl, pH 8) at 37 °C for 12 h. b) Reduction of C14–C38 disulfide in BPTI (**8**, 250 μM) by 1.5 equiv of TCEP in 100 mM PB (pH 8) at room temperature for 3 h; dual modifications of C14 and C38: **2e**/**2j** (5 equiv) in 100 mM PB (pH 8) at 37 °C for 12 h. Partly reduced **8** (Found 6513.6 Da, Calcd. 6513.5 Da); the HR-MS spectra of the products **9a** and **9b** are also provided. c) The conversion of BSA modification is 99%; no BSA (**10**) was observed after 12 h. d) Trastuzumab (**12**, 0.5 mg/mL) was incubated with 10 equiv of TCEP in 100 mM PB (pH 8), 37 °C for 40 min followed by adding 200 equiv of ynamide (**2e**) for 20 h. Deconvoluted MS spectra of partly reduced trastuzumab labeled with **2e**. LC, light chain; HC, heavy chain; subscripts, number of labeling.

Next, we investigated the application of ynamide β-hydrosulfuration for protein Cys modification. Biotinylated ynamide (**2j**) was readily prepared and used as a model substrate for the protein Cys modification. A ubiquitin(G47C) variant (**6**, 250 μM, prepared by chemical protein synthesis, see the SI for details)^48^ was treated with 5 equiv of **2j** at pH 8 and 37 °C, to afford the biotin-labeled ubiquitin(G47C*) (**7**) in 43% isolated yield (HPLC purification) after 12 h (Figure 3a). In contrast, no modification of ubiquitin(G47A) variant^49^ was observed under the same reaction conditions even after 18 h, confirming the Cys selectivity. The efficient chemo-, regio-, and stereoselective biotinylation of the ubiquitin(G47C) variant (**6**) unambiguously illustrated the potential application of this strategy in the construction of protein conjugates. Furthermore, the modification of bovine pancreatic trypsin inhibitor (BPTI), a 58-residue Cys-rich protein with three disulfide bonds, was also examined.^50^ First, BPTI (**8**, 250 μM) was treated with 1.5 equiv of tris(2-carboxyethyl)phosphine (TCEP), which selectively reduced the partly solvent exposed Cys14–Cys38 disulfide bond,^50^ leaving the other two buried disulfide bonds intact, followed by the addition of 10 equiv (due to low accessibility of the Cys14 and Cys38 residues) of **2e**/**2j**. After 12 h, the corresponding bis-modified proteins (**9a** and **9b**) were isolated in 21% and 29% yields (HPLC purification), respectively (Figure 3b). Although 8% oxidation of the reduced Cys residues to form disulfide bonds of native **8** were detected in both cases, no perturbation on other disulfides of BPTI was observed (a detailed description of MS/MS analysis for the selective modification of Cys14 and Cys38 in BPTI with ynamide **2e** was included in the SI, see Figures S53–S55). BSA (**10**) is another disulfide-rich protein with 17 disulfide bonds and one free Cys residue (Cys34). When BSA was treated with ynamide **2j**, only the monoadduct was observed as confirmed by MALDI-TOF (Figure 3c). The compatibility of the disulfide bond in this transformation implied the application potential of this protocol in complex biological systems. For example, a Her2-targeting IgG therapeutic, the antibody trastuzumab^52^ (**12**), was chosen as a model to probe the feasibility of using ynamide for antibody–drug conjugates (ADC) study. The conjugation was conducted using a typical reduction-addition procedure.^33a^ A 40 min reduction with 10 equiv of TCEP at 37 °C was performed to unlock the solvent-exposed interchain disulfide bridges. The reduced antibody was then incubated with prototypical ynamide **2e** at 37 °C for 20 h, while the heterogeneous *N*-glycan was removed to simplify the analysis. Labeling was confirmed according to LC-MS analysis (Figure 3d). A labeling degree of 3.7 per antibody could be observed, including partially one ynamide (**2e**, +217.5 Da, LC_1_) labeling in the light chain, as well as partially one ynamide (+215.2 Da, HC_1_), two ynamides (+433.5 Da, HC_2_), and three ynamides (+651.5 Da, HC_3_) labeling in the heavy chain (Figure 3d). In a control reaction without TCEP reduction, no labeling was observed, showing the high chemoselectivity of ynamides toward reduced Cys residues. Considering I. the high chemo-, regio-, and stereoselectivity of the reaction and II. the great stability of the formed C–S bond, ynamides hold great potential in ADC-based therapeutics.

### Generation of a Trifunctional Protein by Ynamide-Based Bioconjugation with Fluorescein or PEG Polymers

**Figure 4:**
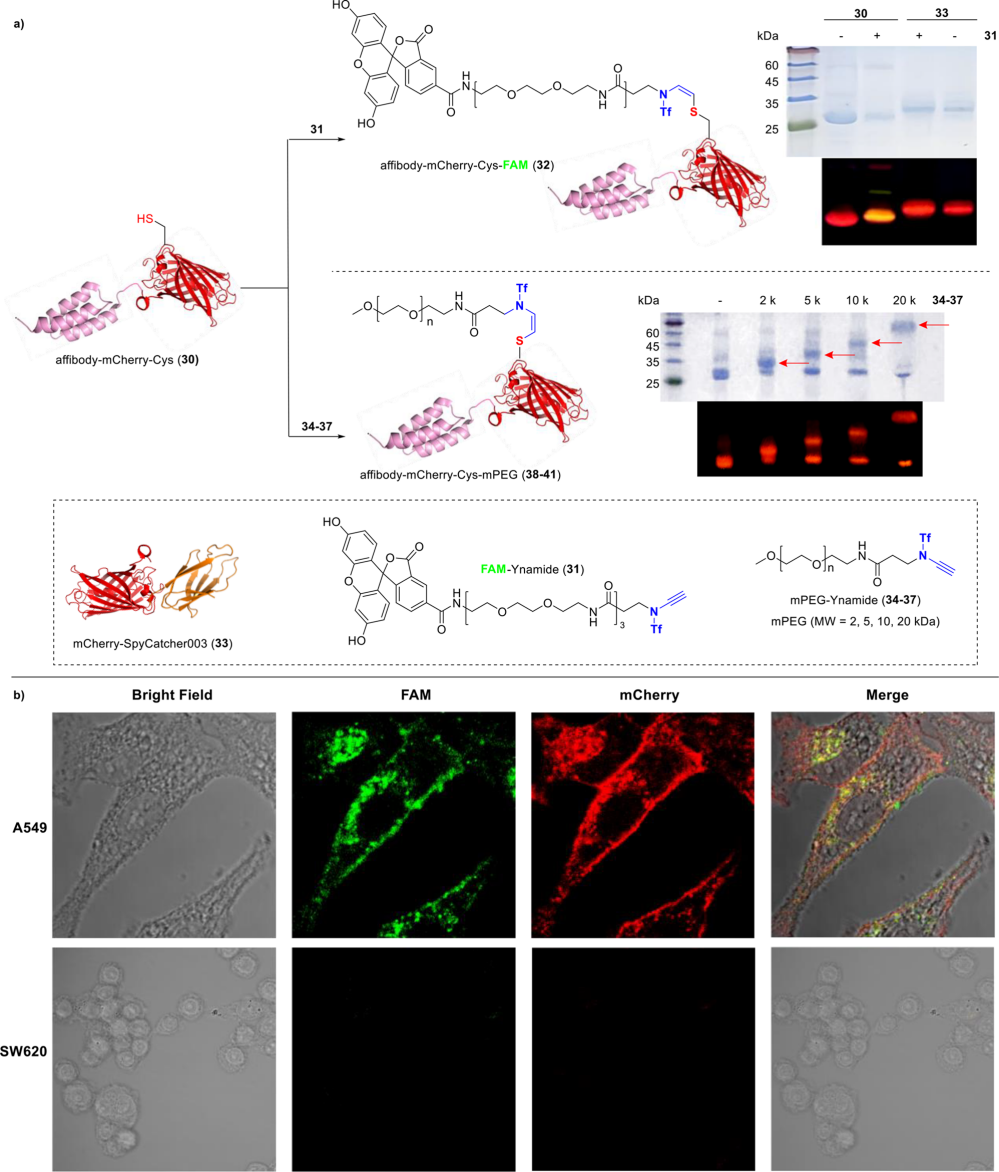
Generation of a trifunctional protein with fluorescein or PEG polymers. (A) Site-specific fluorescent labeling of affibody-mCherry-Cys (**30**) and mCherry-SpyCatcher003 (**33**) with FAM-ynamide (**31**) or ynamide-PEG (**34**: 2 kDa, **35**: 5 kDa, **36**: 10 kDa, **37**: 20 kDa), analyzed by SDS-PAGE and fluorescent gel analysis; red arrowheads indicate the PEGylated proteins with different sizes. (B) Confocal microscopy images of A549 (EGFR-positive) and SW620 (EGFR-negative) cells after labeling with affibody-mCherry-FAM (**32**) at 500 nM. Scale bar = 10 μm.

To further test whether ynamide-based bioconjugation compromises the activity or function of proteins, we prepared a dual-functional protein with tumor targeting and fluorescence emission capabilities as a demonstration (Figure S78). In this hybrid protein, mCherry serves as an easily detectable tracing moiety, while the anti-EGFR affibody functions as the targeting component for the specific detection of the frequently overexpressed EGFR antigen in various tumors.^53^ A single Cys residue was incorporated into this protein to conjugate with ynamide (Affibody-mCherry-Cys, **30**, Figure 4 and Figure S79). To endow this bifunctional protein with additional properties, a water-soluble ynamide-fluorescent labeling reagent (FAM-ynamide, **31**) containing a hydrophilic short-chain poly(ethylene glycol) (PEG) was used. Prereduced affibody-mCherry-Cys (**30**, 100 μM) treated with TCEP was exposed to ynamide-FAM (**31**, 1 mM) in PB (pH 8.0) for 12 h at 37 °C, successfully yielding a trifunctional protein affibody-mCherry-Cys-FAM (**32**) with one EGFR-targeting moiety and two-color fluorescence elements.

This was evidenced by the yellow fluorescence observed in the gel due to the merged FAM and mCherry signals along with a slight upward gel shift. Conversely, a negative control with mCherry-SpyCatcher003 (**33**) lacking a Cys residue showed no such effects, highlighting the thiol specificity of the conjugation (Figure 4a and Figure S80). To test if this trifunctional protein retained its targeting ability toward EGFR, it was applied to two cancer cell lines (A549 and SW620). After 40 min of treatment with 500 nM and 1 μM of **32**, respectively, only EGFR-positive A549 cells displayed colocalized green and red fluorescence while no significant fluorescence was detected in EGFR-negative SW620 cells (Figure 4b and Figure S81), proving the targeting capability remains after labeling (Figure 4b). This data firmly showed that the ynamide bioconjugation method could be a fully biocompatible tool to decorate proteins without compromising their integrity and normal functions, thereby offering significant potential for both diagnostics and therapeutics. Subsequently, given the efficacy of PEG polymers in enhancing the stability of various protein therapeutics,^54^ we wondered whether our conjugation method could also facilitate the preparation of PEGylated proteins. To this end, ynamide-functionalized PEG polymers of various molecular weights (**34**: 2 kDa, **35**: 5 kDa, **36**: 10 kDa, and **37**: 20 kDa) were prepared and used to conjugate with **30**, adopting a procedure similar to that described above (Figure 4a). Then the reaction mixture was directly used for SDS-PAGE analysis without purification. Results showed successful attachment across all PEG sizes to the protein, as evidenced by the appearance of bands corresponding to the higher molecular weights of the PEGylated products (**38**–**41**), visualized by Coomassie blue staining and red fluorescence (Figure 4a). These results again highlight that ynamide-based conjugation maintains high efficiency even with large-molecular-weight PEGs, underscoring its versatility for protein functionalization across a wide range of molecular sizes. This approach is particularly promising for developing enhanced protein therapeutics.^55^

### Implementation of Ynamide As the Protein Alkenylation Reagent in Proteomics Analysis of *E. coli* Lysate Samples

**Figure 5:**
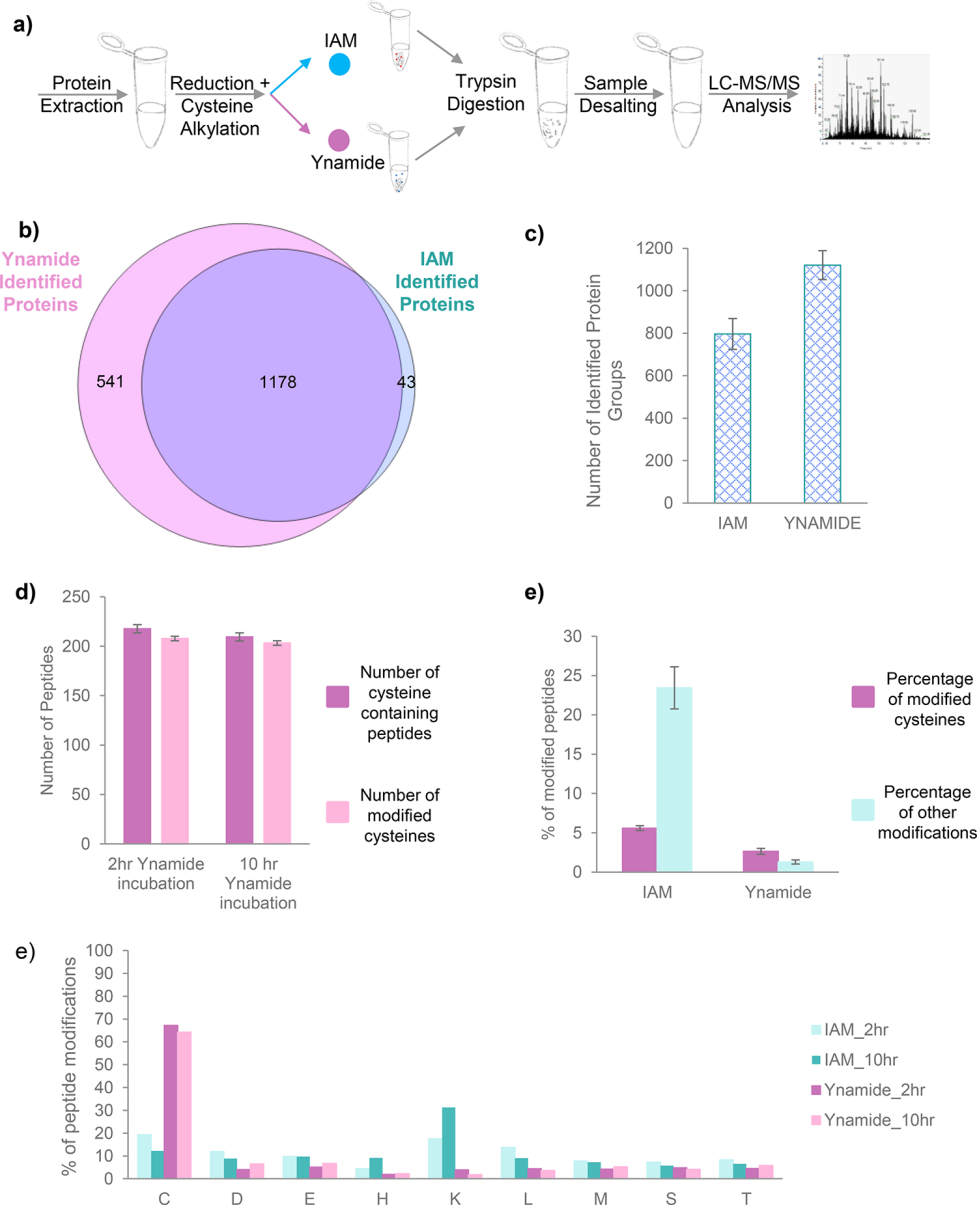
Proteomic implementation and analysis of ynamide in *E. coli* lysate samples. a) Analysis of *E. coli* lysate samples was performed according to a standard proteomic workflow where samples were lysed and then reduced followed by cysteine alkylation or alkenylation by either IAM or ynamide (**2e**), respectively. All samples were then digested with trypsin, desalted, and analyzed by LC-MS/MS as described in methods. b) Proteomic analysis in both IAM and ynamide samples has identified 1178 *E. coli* proteins (shown in purple). 43 proteins were uniquely identified in the IAM-labeled sample (shown in cyan), whereas 541 proteins were only identified in the ynamide-labeled samples (shown in pink). c) Number of identified protein groups in IAM- and ynamide-modified samples. d) Comparison of the number of Cys-containing peptides (in purple) that were identified, and number of cysteines that were found modified (in light pink), between samples incubated with ynamide for 2 and 10 h. e) Percentage of modified cysteines (purple) or other modified amino acids (lime) in IAM- or ynamide-labeled samples out of all detected peptide–spectrum matches (PSMs). f) Percentage of IAM or ynamide modification detected on cysteine and noncysteine amino acids in IAM (in shades of turquoise) or ynamide (in pink) treated samples after 2 or 10 h of incubation.

Alkylation or alkenylation of protein cysteines is the seminal part of almost all proteomic workflows used today in biology. To examine the specificity and applicability of the ynamide probe in a biological system, we performed shotgun proteomic analysis of *E. coli* lysate samples using alkylation of fully reduced cysteines by either iodoacetamide (IAM), which is by far the most commonly used reagent in the proteomic analysis,^51^ or ynamide (**2e**). *E. coli* proteins were extracted, and reduced with excess DTT, followed by treatment with either IAM or ynamide **2e**. After trypsin digestion, the peptides were desalted and analyzed by LC-MS/MS following a standard proteomic workflow, as described in the methods section (see Figure 5a and SI for more details). Three biological replicates were used in each labeling method, while 0.3 μg of *E. coli* peptides was analyzed in each sample. The analysis revealed 1178 overlapped proteins, which were identified in both IAM- and ynamide-labeled samples; however, an additional 541 proteins were identified only in the ynamide-labeled samples, and 43 proteins were identified in the IAM-labeled sample (Figure 5b, c). Importantly, an incubation time of 2 h with ynamide was sufficient to obtain merely full cysteine labeling, similar to the efficiency of 10 h incubation (Figure 5d). Furthermore, 10 h alkenylation with ynamide did not reduce the labeling efficiency or lead to additional nonspecific modification of cysteines or other amino acids. In contrast, IAM alkylation led to a large fraction of nonspecific modifications of noncysteine residues (Figure 5e), which was also observed by other groups.^29b,56^ While ynamide may interact with the same type of noncysteine amino acids as IAM, the degree of such nonspecific modification is significantly lower than in the IAM-labeled sample (Figure 5e, f; see also details in the SI including Figure S119 for noncysteine carbamidomethylation upon iodoacetamide treatment in two proteomics experiments of *E. coli* and *S. cerevisiae* proteomes). Additionally, there are several studies that highlighted the deleterious effects of halogenated acetamide on methionine- and/or tryptophan-containing peptides, particularly in proteomic analyses; for example, see ref (51). This makes ynamide a preferable thiol-trapping reagent that can be potentially used in redox proteomics and complex proteomic platforms combining quantifications of multiple PTMs.

## Conclusion

In summary, we have developed a novel base-promoted chemo-, regio-, and stereoselective ynamide β-hydrosulfuration which proceeded in a “click” manner, and successfully applied it to peptide and protein Cys modification. The three flexible sites on ynamides, including the alkynyl moiety and two substituents on the nitrogen atom, offered great chemical space for optimization and diversification. The strong electron-withdrawing triflyl group played a crucial role, which guaranteed the stability and improved the β-addition reactivity of ynamide toward Cys. In addition, the other substituent on the nitrogen atom offered a handle for functional expansion with various functional moieties. This method exhibited excellent chemoselectivity for thiol groups in the presence of other peptide side chains including carboxylic acid, free amino, primary amide, hydroxyl group, guanidinium group, as well as NH of imidazole and indole. The compatibility with click chemistry (performed in one pot) extended the application of this bioconjugation technique. The robustness of this method in chemical biology was further exemplified by applications in chemical modifications of peptides (both linear and cyclic peptides) and proteins, including the chemoselective biotinylation of ubiquitin(G47C) variant, as well as the regioselective modification of Cys14 and Cys38 in bovine pancreatic trypsin inhibitor (BPTI), Cys34 of BSA, and the antibody trastuzumab. The thiol-specific ynamide bioconjugation technique successfully labeled EGFR-targeting affibodies for fluorescence marking of cancer cells, and efficiently conjugated PEG polymers of varying molecular weights to proteins, demonstrating its potential for diagnostic and therapeutic application. Moreover, the specificity and applicability of ynamide in a biological system was emphasized by large-scale proteomics and compared to the most common reagent, iodoacetamide. Ynamide was found to be a superior reagent for thiol trapping, as it was much more specific for Cys modification, which is one of the major drawbacks of iodoacetamide. This finding opens new opportunities to utilize ynamide as a basis for deriving new probes for redox proteomic pipeline, which relies on a high specificity of differential alkylating reagents. Furthermore, ynamide-modified peptide conjugates displayed remarkable stability under rigorous conditions, including acidic, basic, and oxidative conditions, as well as competition with external thiols. This study provides a useful chemical tool for peptide and protein precise modification.

## Supporting Information Available

Materials and methods, experimental procedures, characterization, HPLC and LC-MS data, as well as HRMS, ^1^H and ^13^C NMR spectra (PDF)
