# Supplementary material for: Peptide and Protein Cysteine Modification Enabled by Hydrosulfuration of Ynamide
Source: ACS Cent Sci. 2024 Aug 21;10(9):1742–54. doi: 10.1021/acscentsci.4c01148 (PMC11428291; doi:10.1021/acscentsci.4c01148)
Supplement: Supplementary file 1 — oc4c01148_si_001.pdf [file oc4c01148_si_001.pdf]

Supplementary Information

Peptide and Protein Cysteine Modification Enabled by Hydrosulfuration of Ynamide

Changliu Wang[1,4†], Zhenguang Zhao[2†], Reem Ghadir[2†], Dechun Yang[3†], Zhenjia Zhang[1†], Zhe Ding[4], YuanCao[5], Yuqing Li[4], Rosi Fassler[6], Dana Reichmann[6,7], YujieZhang[5], Yongli Zhao[4], Can Liu[1], Xiaobao Bi[3]*, Norman Metanis[2,6,7,8]*, Junfeng Zhao[1]*

[1]Affiliated Cancer Hospital, Guangdong Provincial Key Laboratory of of Major Obstetric Diseases, School of Pharmaceutical Sciences, Guangzhou Medical University, Guangzhou 511436, Guangdong, P. R. China.

[2]Institute of Chemistry, The Hebrew University of Jerusalem, Jerusalem 9190401, Israel.

[3]Collaborative Innovation Center of Yangtze River Delta Region Green Pharmaceuticals & College of Pharmaceutical Sciences, Zhejiang University of Technology, Hangzhou 310014, Zhejiang, P. R. China.

[4]College of Chemistry and Chemical Engineering, Jiangxi Normal University, Nanchang 330022, Jiangxi, P. R. China.

[5]Department of Process Development, BeiGene Guangzhou Biologics Manufacturing Co., Ltd., Guangzhou 510700, Guangdong, P. R. China.

[6]The Alexander Silberman Institute of Life Science, [7]The Center for Nanoscience and Nanotechnology, [8]Casali Center for Applied Chemistry, The Hebrew University of Jerusalem, Jerusalem 9190401, Israel.

These authors contributed equally: Changliu Wang, Zhenguang Zhao, Reem Ghadir, Dechun Yang and Zhenjia Zhang.

E-mail: zhaojf@gzhmu.edu.cn; metanis@mail.huji.ac.il; xbbi@zjut.edu.cn

Content

1. General Remarks 3

2. General Procedures for Preparation of Ynamides, Peptides and Proteins 4

3. Optimization of Reaction Conditions for Cys Modification with Ynamides 24

4. Systematic Study of Cys Modification by Ynamides in the Presence of Other Amino Acid Residues 35

5. One-Pot Dual Functionalization of Cys with Click Chemistry 75

6. Modification of Cys-Containing Proteins 77

7. Stability Study and Reaction Rate study of the Peptide Conjugates 85

8. Trastuzumab Reduction 89

9. The Chemoselectivity Evaluation of Cys Modification by Different Reagents 91

10. Radical Experiments 99

11. Generation of a trifunctional protein by ynamide-based bioconjugation with fluorescein or PEG polymers 101

12. Supplementary Figures 107

13. Proteomic Implementation and Analysis of Ynamide in E. coli Lysate Samples 121

14. References 123

15. NMR Spectrum 125

1. General Remarks

All Fmoc-amino acids were purchased from CS Bio Co. (Menlo Park, CA) and Matrix Innovation (Quebec City, Canada) Small organic molecules were purchased from Leyan, Energy Chemical. Bovine pancreatic trypsin inhibitor (WT-BPTI) was a generous gift of Bachem AG. *E.coli* DH5-*α* sensory state cell and BL21 (DE3) sensory state cell, Gel extraction kit and Fetal Bovine Serum were available from TransGen Biotech (Beijing, China). Dulbecco’s modified Eagle’s medium (DMEM), 1×PBS (pH 7.4), and Penicillin streptomycin solution were purchased from Solarbio (Beijing, China). Hoechst 33342 dye were purchased from Beyotime Biotechnology (Shanghai, China). Phanta max superfidelity DNA polymerase was purchased from Vazyme (Nanjing, China). ^{1}H- and ^{13}C spectra were recorded on a Bruker (400 MHz for ^{1}H, 100 MHz for ^{13}C respectively) instrument, and are internally referenced to residual solvent signals, $CDCl_3$ referenced at δ 7.26 and 77.00 ppm, DMSO-d_6 referenced at δ 2.50 and 39.8 ppm. Data for ^{1}H is reported as follows: chemical shift (δ ppm), integration, multiplicity (s= singlet, d = doublet, t = triplet, q = quartet, m = multiplet), broad peaks (br), coupling constant (Hz) and assignment. Data for ^{13}C NMR are reported in terms of chemical shift (δ ppm), multiplicity (s = singlet, d = doublet, t = triplet, q = quartet, m = multiplet), coupling constant (Hz) and no special nomenclature is used for equivalent carbons. HRMS (ESI) spectra were obtained by the electrospray ionization time-of-flight (ESI-TOF) mass spectrometry. Flash column chromatography purification of compound was carried out by gradient elution using ethyl acetate (EA) in light petroleum ether (PE) or gradient elution using methanol (MeOH) in dichloromethane (DCM) Unless otherwise noted, materials obtained from commercial suppliers were used without further purification. Analytical RP-HPLC was performed on an UltiMate 3000 HPLC system with appropriate columns and elution conditions. Confocal microscope model is Nikon AX R. NdeI and KpnI restriction endonuclease were purchased from New England Biolabs (Beijing, China).

IPTG and Ampicillin were available from Sangon (Shanghai, China). Ni-NTA Beads were purchased from Smart-Lifescience (Xian, China). Tris(2-carboxyethyl) phosphine hydrochloride (TCEP) were purchased from Bide Pharmatech Ltd (Shanghai, China). The model of gel imager is CLINX GenoSens 2150(T)/2250(T). All oligonucleotide sequences were synthesized and purified by Qinko Biotechnology (Beijing, China). The SimpliAmp^{TM} PCR instrument used for the gene fragments synthesised in the experiment was purchased from Thermo Fisher Scientific (Shanghai, China). The plasmids in the experiment were sequenced at Qinko Biotechnology to determine the gene sequences. Commercial sources were used to get other unspecified reagents.

2. General Procedures for Preparation of Ynamides, Peptides and Proteins

(1) General procedures for preparation of ynamides ***2a-2d***[1]

(a) *N*, 4-dimethylbenzenesulfonamide (2.0 mmol), NaH (4.0 mmol, 2.0 equiv) were combined in an oven-dried Schlenk tube equipped with a stir-bar. After the addition of all solid reagents, 20.0 mL of DMSO was added to the tube via a syringe, and the reaction mixture was allowed to stir at rt for 15 min. The Schlenk tube was heated to 70 °C. Then 1,1-dichloroethene (4.0 mmol, 2.0 equiv) was added to the Schlenk tube slowly via a syringe. The reaction mixture was stirred at 70 °C for 48 h and then cooled to room temperature. The reaction was quenched by cold water and extracted three times with methylene chloride. The combined organic layers were dried over anhydrous Na_2SO_4 and evaporated under vacuum. The desired products were obtained in 80% yield after purification by flash chromatography on silica gel with PE/ EA.

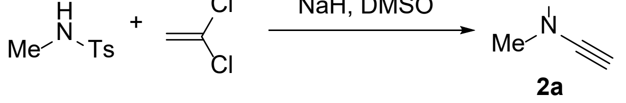

Scheme S1. General procedures for preparation of ynamide **2a**

(b) Step 1: In a 100 mL three-neck round-bottom flask equipped with a stir-bar,

$CuCl_2$ (0.4 mmol, 20%), N-(2-hydroxyethyl)-4-methylbenzenesulfonamide (10.0 mmol, 5.0 equiv) and Na_2CO_3 (4.0 mmol, 2.0 equiv) were combined. The reaction flask was purged with oxygen gas for 15 minutes. A solution of pyridine (4 mmol, 2.0 equiv) in 20.0 ml of toluene was added to the reaction flask via a syringe. Two balloons filled with oxygen gas were connected to the reaction flask via needles. The flask was placed in an oil-bath and heated to 70 °C. A solution of terminal alkyne (2.0 mmol) in 20.0 ml of toluene was added slowly to the flask over 4 h by using a syringe pump. After addition of terminal alkyne/toluene solution, the reaction mixture was allowed to stir at 70 °C for another 16 hours and then cooled to room temperature. The crude mixture was concentrated under vacuum and then purified by flash chromatography on silica gel with PE/EA to afford the ynamide **2b**[1].

Step 2: To a 10 ml round-bottom flask, TMS-ynamide **2b**[1] (2 mmol), CH_3OH and K_2CO_3 (2.0 equiv) were added. The reaction mixture was stirred at room temperature under air until TMS-ynamide **2b**[1] was fully consumed. The reaction mixture was treated with H_2O at room temperature and aqueous layer was extracted three times with Et_2O. The combined organic layers were washed with brine, dried over Na_2SO_4, filtered and evaporated in vacuo. Crude product was purified by flash chromatography to afford the terminal ynamide **2b** in quantitative yield.

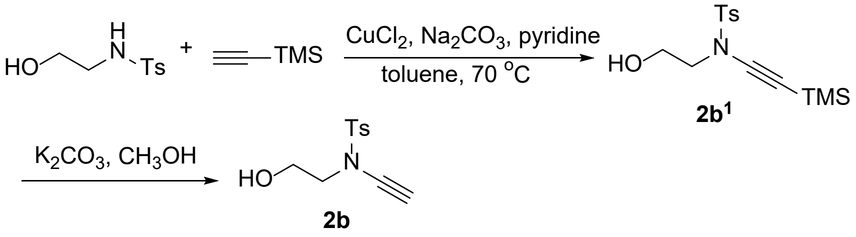

Scheme S2. General procedures for preparation of ynamide **2b**

(c) In a 100 mL three-neck round-bottom flask equipped with a stir-bar, $CuCl_2$ (0.4 mmol, 20%), *N*, 4-dimethylbenzenesulfonamide (10.0 mmol, 5.0 equiv) and Na_2CO_3 (4.0 mmol, 2.0 equiv) were added. The reaction flask was purged with oxygen gas for 15 minutes. A solution of pyridine (4 mmol, 2.0 equiv) in 20.0 ml of toluene was added to the reaction flask via a syringe. Two balloons filled with oxygen gas were connected to the reaction flask via needles. The flask was placed in an oil-bath and

heated to 70 °C. A solution of terminal alkyne (2.0 mmol) in 20.0 ml of toluene was added slowly to the flask over 4 h by using a syringe pump. After addition of terminal alkyne/toluene solution, the reaction mixture was allowed to stir at 70 °C for another 16 hours and then cooled to room temperature. The crude mixture was concentrated under vacuum and then purified by flash chromatography on silica gel with PE/EA to afford the ynamide **2c**[1].

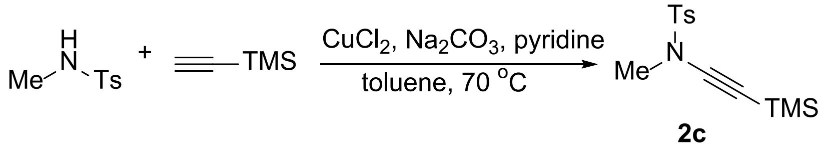

2c

Scheme S3. General procedure for preparation of ynamide **2c**

(d) N-(4-(tert-butyl)phenyl)-4-methylbenzenesulfonamide (2.0 mmol), NaH (4.0 mmol, 2.0 equiv) were combined in an oven-dried Schlenk tube equipped with a stir-bar. After the addition of all solid reagents, 20.0 mL of DMSO was added to the tube via a syringe, and the reaction mixture was allowed to stir at room temperature for 15 min. The Schlenk tube was heated to 70 °C. Then 1,1-dichloroethene (4.0 mmol, 2.0 equiv) was added to the Schlenk tube slowly via a syringe. The reaction mixture was stirred at 70 °C for another 48 h and then cooled to room temperature. The reaction was quenched by cold water and extracted three times with methylene chloride. The combined organic layers were dried over anhydrous Na_2SO_4 and evaporated under vacuum. The desired products were obtained in 80% yield after purification by flash chromatography on silica gel with PE/EA.

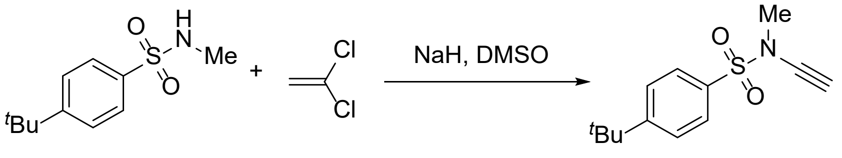

2d

Scheme S4. General procedures for preparation of ynamide **2d**

(2) General procedures for preparation of ynamides ***2e-2g, 2k***

(a) A 100 mL round-bottom flask equipped with a stir bar was charged with primary

amine (10 mmol), triethylamine (2 equiv, 20 mmol). The flask was allowed to put in an ice bath, and then trifluoromathanesulfonyl chloride (1.2 equiv, 12 mmol) was added to the flask dropwise. The reaction mixture was allowed to stir at 0 °C until primary amine was completely consumed. After the reaction was completed, the reaction mixture was treated with saturated $Na_2S_2O_3$ solution, the aqueous layer was then extracted with DCM (3 × 10 mL). The combined organic layer was washed with brine, dried over anhydrous $MgSO_4$, filtered and evaporated under vacuum. The residue was purified by flash chromatography to afford target product RNHTf.

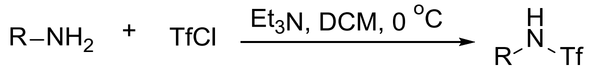

Scheme S5. General procedures for preparation of RNHTf

(b) In a 250 mL round-bottom flask, (diacetoxyiodo)benzene (12.88 g, 40 mmol) was dissolved in DCM (200 mL), which was cooled down to 0 °C with ice bath. Then trifluoromethanesulfonic anhydride (5.64 g, 20 mmol) in 20 mL DCM was added dropwise to the reaction mixture for 24 min while stirring. The reaction was stirred for 40 min at the same conditions. Later bis(trimethylsilyl)acetylene (6.82 g, 40 mmol) was added to the reaction mixture, which was stirred at 0 °C for additional 5 h. Upon completion of the reaction, the volume of organic solvent was reduced under reduced pressure, diethylether was then added to help the precipitation of the product (12.2 g, 27 mmol, 68% yield, white solid).

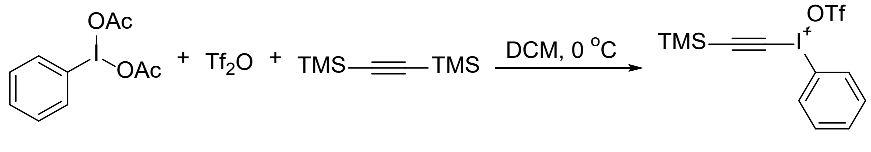

Scheme S6. General procedures for preparation of hypervalent iodine reagent

(c) A 100 mL round-bottom flask was charged with RNHTf (2 mmol), K_2CO_3 (5 mmol, 2.5 equiv) and 20 mL DMF. The reaction mixture was stirred at room temperature for 5 min. Then reaction flask was placed in a 0 °C cold bath and stirred for 15 min. Then hypervalent iodine reagent (2.4 mmol, 1.2 equiv) in DCM (20 mL)

was added slowly to the reaction mixture, which was stirred at 0 °C for 40 min. Upon reaction completion, the reaction mixture was diluted with H_2O and the aqueous phase was extracted with DCM (3 x 15 mL). The combined organic layers were washed with brine and dried over anhydrous sodium sulfate, filtered, and concentrated under reduced pressure. Purification of the product **2** was performed via flash chromatography.

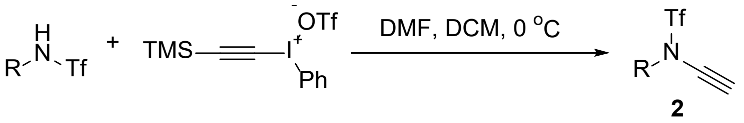

Scheme S7. General procedures for preparation of ynamides **2e-2g, 2k**

(3) General procedures for preparation of ynamides ***2i & 2j***

A 50 mL round-bottom flask was charged with biotin or indomethacin (1 mmol), **2e** (1.2 mmol, 1.2 equiv), 1-ethyl-3-(3-dimethylaminopropyl)carbodiimide (EDCI) (1.2 mmol, 1.2 equiv), and 4-dimethylaminopyridine (DMAP) (2 mmol, 2 equiv) with 10 mL DMF. Reaction was stirred at room temperature for 4 h. After reaction completion, the organic solvent was evaporated under reduced pressure. Later product **2** was purified by silica gel column chromatography.

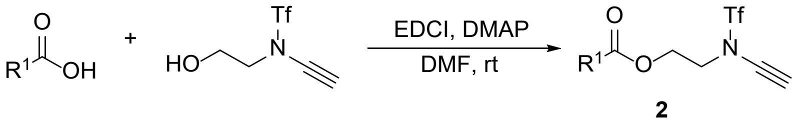

Scheme S8. General procedures for preparation of ynamides **2i & 2j**

(4) General procedures for preparation of ynamide ***2h***

Step 1: A 100 mL round-bottom flask was charged with 2-oxo-2H-chromene-3-carboxylic acid (5.0 mmol), *tert*-butyl (2-aminoethyl) carbamate (6.0 mmol, 1.2 equiv), EDCI (6.0 mmol, 1.2 equiv) and DCM (50.0 mL). The reaction mixture was allowed to stir for 30 min at room temperature. Later the product was purified by silica gel column chromatography.

Step 2: A 50 mL round-bottom flask was charged with *tert*-butyl

(2-(2-oxo-2H-chromene-3-carboxamido)ethyl)carbamate (2.0 mmol), then 50% TFA in DCM, which was stirred at room temperature for 20 min. The reaction was quenched by cold water and the product was extracted three times with 2.0 M $NaHCO_3$ followed by the purification with silica gel column chromatography.

Step 3: A 50.0 mL round-bottom flask was charged with N-(2-aminoethyl)-2-oxo-2H-chromene-3-carboxamide (2.0 mmol), Et_3N (2.4 mmol, 1.2 equiv), 20.0 mL DCM, which was placed in a 0 °C cold bath and stirred for 5 min. Then TfCl (2.4 mmol, 1.2 equiv) was added slowly to the reaction mixture, after that, the reaction was stirred at room temperature for 30 min. Upon reaction completion, the product was extracted three times with DCM followed by the purification with silica gel column chromatography.

Step 4: A 25 mL round-bottom flask was charged with 2-oxo-N-(2-((trifluoromethyl) sulfonamido)ethyl)-2H-chromene-3-carboxamide (2 mmol), K_2CO_3 (5 mmol, 2.5 equiv) and 10 mL DMF. The reaction mixture was stirred at 0 °C for 5 min. Hypervalent iodine (2.4 mmol, 1.2 equiv) in DCM (10.0 mL) was added slowly to the reaction mixture, which was retained at 0 °C for additional 30 min. The reaction was quenched by cold water and the product was extracted three times with DCM followed by the purification with silica gel column chromatography.

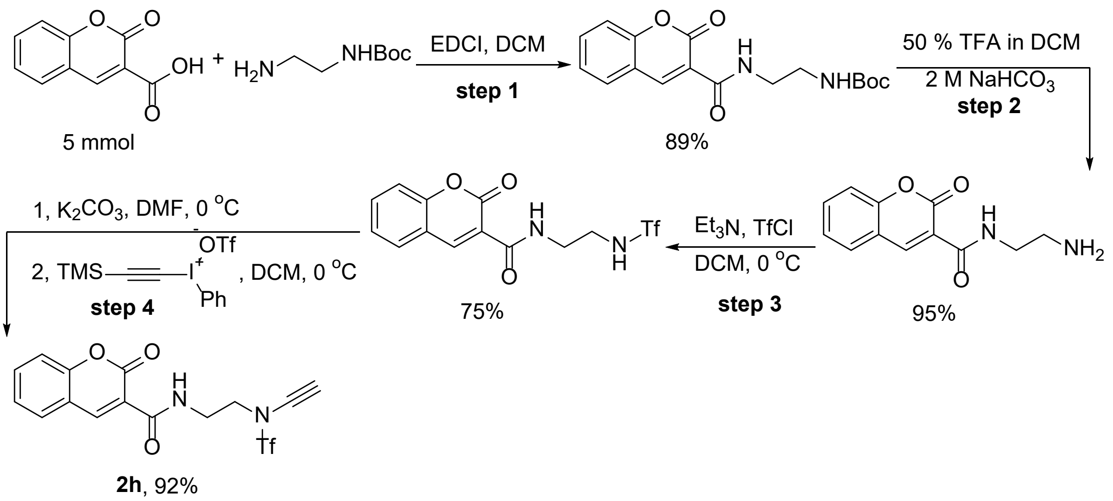

Scheme S9. General procedures for preparation of ynamide **2h**

(5) General procedures for preparation of ynamide ***31***[d]

Step 1: A 50.0 mL round-bottom flask was charged with tert-butyl 3-aminopropanoate hydrochloride (5.0 mmol), Et_3N (17.5 mmol, 3.5 equiv), 50.0 mL DCM, which was placed in a 0 °C cold bath and stirred for 5 min. Then TfCl (6.0 mmol, 1.2 equiv) was added slowly to the reaction mixture, after that, the reaction was stirred at room temperature for 30 min. Upon reaction completion, the product was extracted three times with DCM followed by the purification with silica gel column chromatography.

Step 2: A 50 mL round-bottom flask was charged with *tert*-butyl 3-((trifluoromethyl) sulfonamido)propanoate (2.0 mmol), then 25% TFA in DCM, which was stirred at room temperature for 1 h. The reaction was quenched by cold water and the product was extracted three times with EtOAc followed by the purification with silica gel column chromatography.

Step 3: A 50.0 mL round-bottom flask was charged with 3-((trifluoromethyl) sulfonamido)propanoic acid (2.0 mmol), 1-phenylbuta-2,3-dien-1-one (2.4 mmol, 1.2 equiv), 10.0 mL DCM, which was stirred at 50 °C for 5 h. After reaction completed, the organic solvent was evaporated under reduced pressure. Later product was purified by silica gel column chromatography.

Step 4: A 25 mL round-bottom flask was charged with (*E*)-4-oxo-4-phenylbut-2-en-2-yl 3-((trifluoromethyl)sulfonamido)propanoate (2 mmol), K_2CO_3 (5 mmol, 2.5 equiv) and 10 mL DMF. The reaction mixture was stirred at 0 °C for 5 min. Hypervalent iodine (2.4 mmol, 1.2 equiv) in DCM (10.0 mL) was added slowly to the reaction mixture, which was retained at 0 °C for additional 20 min. The reaction was quenched by cold water and the product was extracted three times with DCM followed by the purification with silica gel column chromatography.

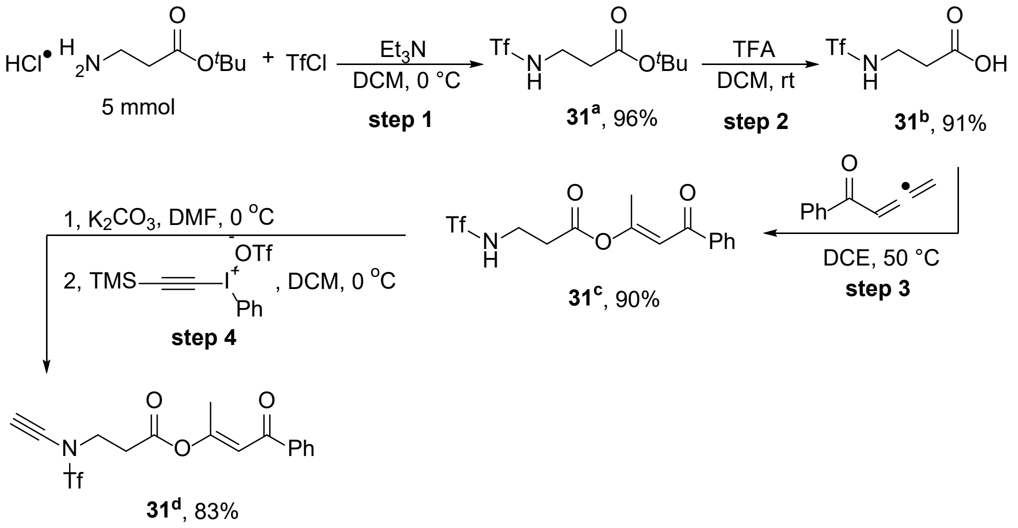

Scheme S10. General procedures for preparation of ynamide **31**[d]

*(6) General procedures for preparation of Ynamide-FAM **31***

Ynamide-PEG-OH were synthesized manually on a 0.2 mmol scale by Fmoc-SPPS. After 2-chlortrityl resin-(PEG)2-Fmoc is synthesized using solid phase synthesis standard conditions, the Fmoc protection is removed. Add Tf-Ynamide activated ester (2 equiv), HOBt (0.5 equiv), DMF (2 mL) and DCM (2 ml), and allowed to couple for 2 h with constant shaking. after reaction for 2 h, the product is treated with a cocktail (AcOH: TFE: DCM = 10: 20: 70) for 2 h at room temperature., and the crude product is washed with PE and dried. The product was transferred to a 10mL round-bottomed flask, with acetylene amide (2 eq.), DCM 5 mL, and reacted at 45°C for 48 h. The resulting resins were washed with DMF (×3), DCM (×3) and dried. The compounds were cleaved off resin using a cocktail (AcOH: TFE: DCM = 10: 20: 70) for 2 h at room temperature. and the crude product is washed with PE and dried. The product was transferred to a 10mL round-bottomed flask, with acetylene amide (2 equiv), DCM 5 mL, and reacted at 45°C for 48 h. After reaction completed, the organic solvent was evaporated under reduced pressure. Later product was purified by silica gel column chromatography.

Step 1: A 10 mL round-bottom flask was charged with 5-FAM-COOH (0.2 mmol), DIC (0.24 mmol, 1.2 equiv), Oyxma (0.24 mmol, 1.2 equiv) and DMF (5 mL). The reaction mixture was allowed to stir for 5 min at room temperature. Then *tert*-butyl

(2-(2-(2-aminoethoxy)ethoxy)ethyl)carbamate (0.8 mmol, 4.0 equiv) was added to solution and the reaction mixture was stirred for 48 h. After reaction completed, the organic solvent was evaporated under reduced pressure. Later product was purified by silica gel column chromatography.

Step 2: A 10 mL round-bottom flask was charged with *tert*-butyl (2-(2-(2-(3',6'-dihydroxy-3-oxo-3H-spiro[isobenzofuran-1,9'-xanthene]-5 carboxamido)ethoxy)ethoxy)ethyl)carbamate (0.12 mmol), 2 M HCl (5 mL). The reaction mixture was allowed to stir for 1 h at room temperature. After reaction completed, the organic solvent was evaporated under reduced pressure. After removing excess HCl from the reaction, 4 (0.1 mmol), DIEA (0.24 mmol), HOBt (0.1 mmol) and DMF (5 mL) were added to the round-bottled flask. The reaction mixture was stirred for 48 h. After reaction completed, the organic solvent was evaporated under reduced pressure. Later product was purified by silica gel plate chromatography.

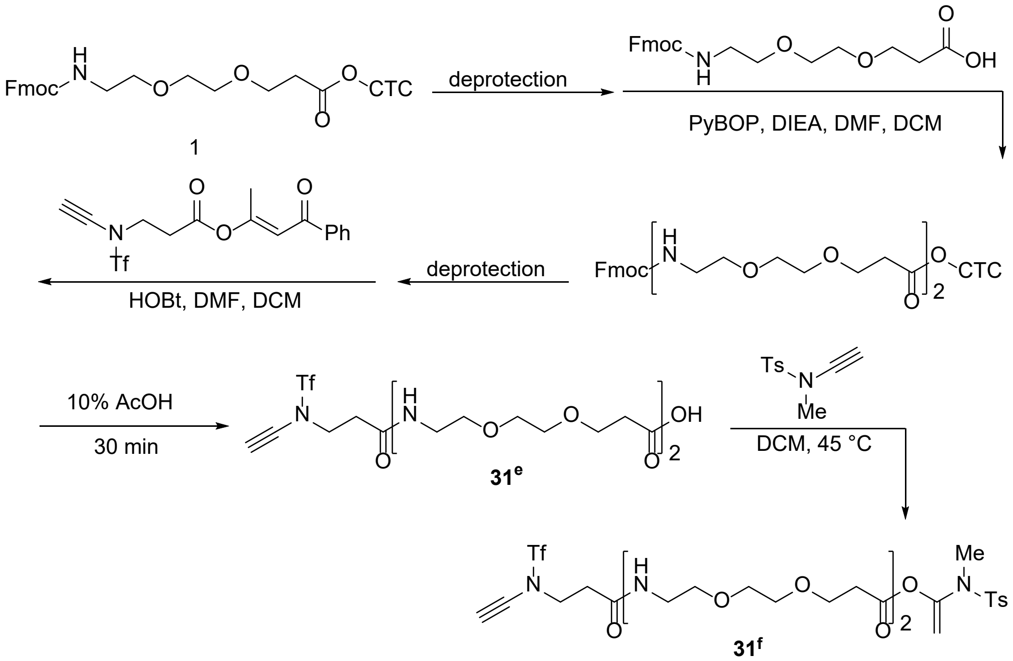

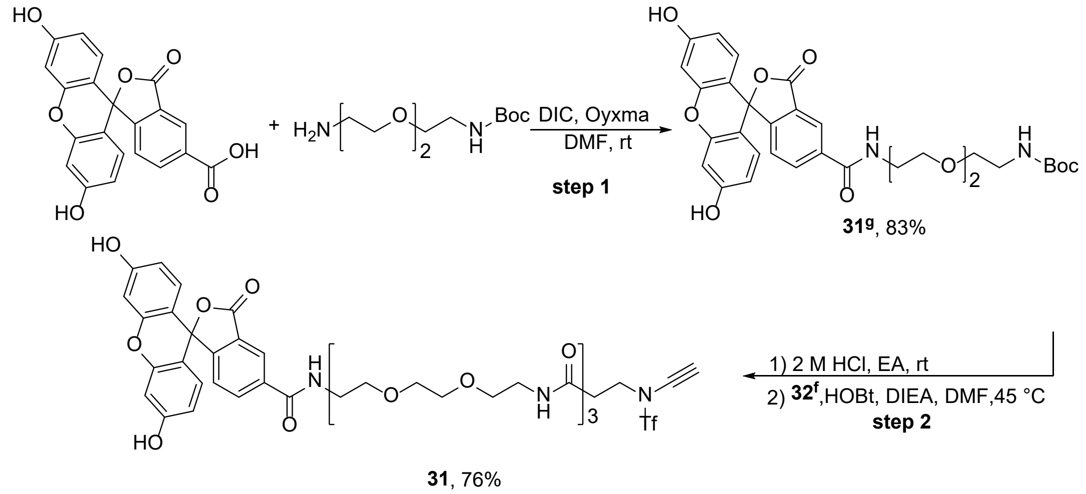

Scheme S11. General procedures for preparation of ynamide **31**

(7) General procedures for preparation of (2 KDa, 5 KDa, 10 KDa, 20KDa) mPEG-Ynamide

A 50 mL round-bottom flask was charged with mPEG-NH_2 (50 mg), Tf-Ynamide activated ester (1.2 equiv), 1 mL MeCN. Reaction was stirred at room temperature for 24 h. After reaction completion, the organic solvent was evaporated under reduced pressure. The reaction solution was dissolved by adding an appropriate amount of DCM, adding n-hexane precipitated products under the condition of ice bath, centrifuge the suspension and wash with n-hexane for three times, and then the products could be obtained after drying.

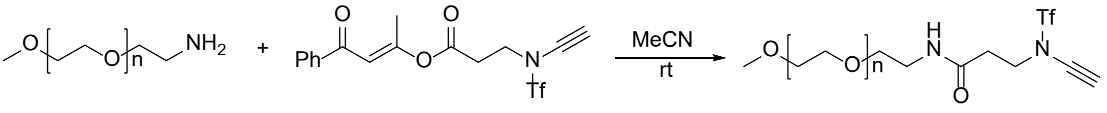

34 - 37

Scheme S12. General procedures for preparation of ynamide **34-37**

*(8) General procedures for dipeptide Boc-Ala-Cys-OEt (**1a**) synthesis*

Step 1: A 250 mL round-bottom flask was charged with Boc-Ala-OH (10 mmol), EDCI (12 mmol, 1.2 equiv), 1-hydroxy-2,5-pyrrolidinedione (HOSu) (12 mmol, 1.2 equiv) and DCM (100 mL). The reaction mixture was allowed to stir for 30 min at room temperature. Then $(H\text{-}Cys\text{-}OEt)_2$ (6 mmol, 0.6 equiv) was added to solution and the reaction mixture was stirred for 30 min. After reaction completed, the organic solvent was evaporated under reduced pressure. Later product $(Boc\text{-}Ala\text{-}Cys\text{-}OEt)_2$

was purified by silica gel column chromatography.

Step 2: A100 mL round-bottom flask was charged with (Boc-Ala-Cys-OEt)$_2$ (5 mmol), ^{n}Bu$_3$P (20 mmol, 4 equiv), 0.5 mL H_2O, and 30 mL DCM. The reaction mixture was allowed to stir for 1 h at room temperature. Later the product was purified by silica gel column chromatography.

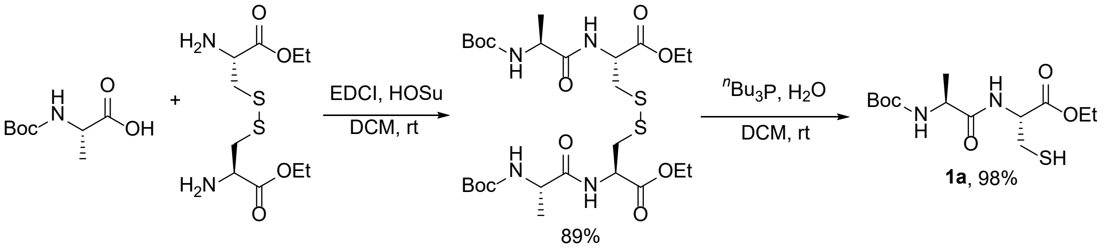

Scheme S13. General procedures for preparation of dipeptide **1a**

(9) General procedure for Fmoc solid-phase peptide synthesis (SPPS)

Peptides were synthesized manually on a 0.2 mmol scale by Fmoc-SPPS. Fmoc deprotection was carried out with 20% piperdine in DMF (2 x 10 min). Fmoc-amino acids (1 mmol in 5 mL of DMF, 4 equiv) were activated with PyBOP (0.6 mmol, 3 equiv) and DIEA (1.2 mmol, 6 equiv) for 5 min and allowed to couple for 30 min with constant shaking. The resulting resins were washed with DMF (×3), DCM (×3) and dried.

The peptides were cleaved off resin using a cocktail (TFA: triisopropylsilane (TIPS): PhSMe: H_2O = 95: 2.5: 2.5: 2.5) for 2.5 h at room temperature. The cleavage mixture was filtered, and the resin was washed with TFA. The combined solutions were concentrated by nitrogen gas bubbling, to which 2-fold volumes of cold diethyether was added dropwise. The precipitated crude peptides were centrifuged, ether was removed, and the crude peptides were dissolved in MeCN:water (1:1) containing 0.1% TFA and lyophilized.

*(10) General procedure for cyclic peptide **1o** synthesis*

Step 1: The linear peptide H-Asp(O^{t}Bu)-Phe(D)-Cys(Trt)-Arg(Pbf)-Gly-OH (1 mmol) was dissolved in DMF (400 mL, c = 2.5 x 10^{-3} M for very high dilution conditions), DPPA (3 equiv) and K_2CO_3 (5 equiv) were added and the mixture was stirred for 4 h

at room temperature in a dark room. The DMF was evaporated under high vacuum. The protected cyclic peptide was precipitated in water and precipitates were collected by centrifugation at 5000 rpm for 5 min and washed three times by Et_2O.

Step 2: The protected cyclic peptide was dissolved in TFA : H_2O : TIPS : PhSMe (95% : 2.5% : 2.5% : 2.5%). The reaction mixture was allowed to stir for 2 h at 37 °C temperature and monitored by LC-MS. The solvent was evaporated under reduced pressure. And the residue was washed by cold ether (4 × 5.0 mL). The protected cyclic peptide was obtained without further purification.

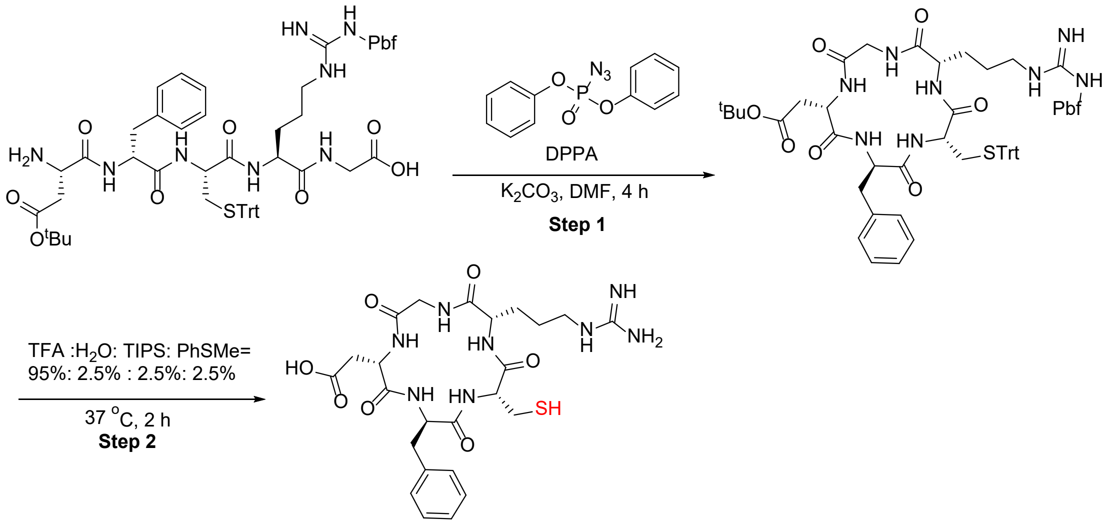

Scheme S14. General procedure for cyclic peptide **1o** synthesis

(11) *The chemical synthesis of* ***Ubiquitin(G47C) (6)***

The sequence of wildtype of ubiquitin:

```
            10          20          30          40          50
MQIFVKTLTG  KTITLEVEPS  DTIENVKAKI  QDKEGIPPDQ  QRLIFAGKQL
            60          70
EDGRTLSDYN  IQKESTLHLV  LRLRGG
```

Ubiquitin consists of 76 amino acids without Cys residue, so the solvent exposed Gly47 was substituted with Cys (shown in *red*). Ubiquitin(G47C) (**6**) was prepared from two segments: ubiquitin(1-46)-MPAA (**6a**) and ubiquitin(47-76)(G47C) (**6b**) with one native chemical ligation (NCL) reaction[2]. The synthesis of each segment and NCL reaction are described in detail below.

(12) *The synthesis of ubiquitin(1-46)-MPAA (**6a**)*

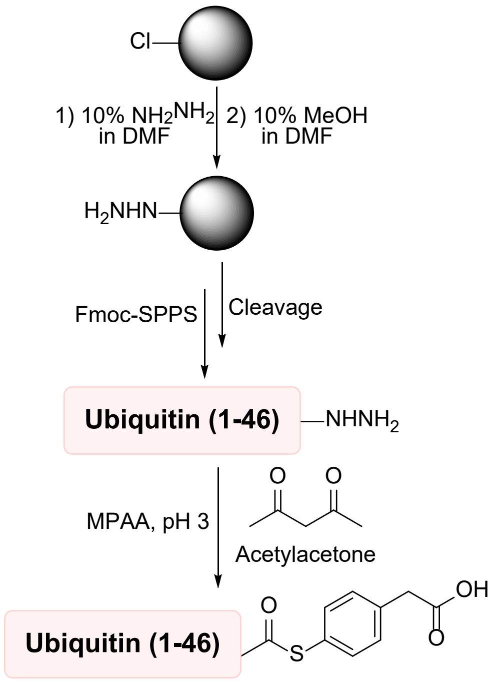

Figure S1. Chemical synthesis of ubiquitin (1-46)-MPAA (**6a**)

Hydrazine loading on resin: 2-chlorotrityl chloride resin (0.25 mmol scale, 0.5 mmol/g) was swelled in DCM: DMF (1:1) for 2 h. The resin was treated twice with fresh 10% hydrazine in DMF for 30 min and drained. The resin was then treated twice with 10% MeOH in DMF for 30 min. After that, the resin was washed well with DCM and DMF.

Synthesis of ubiquitin(1-46)-NHNH2: Ubiquitin(1-46)-NHNH2 was synthesized on hydrazine-functionalized 2-chlorotrityl resin using automatic Fmoc-peptide synthesizer. After completion of synthesis the resin was washed by DCM and cleaved according to the general procedure.

Thioestrification of ubiquitin(1-46)-NHNH2: Crude ubiquitin(1-46)-NHNH2 was dissolved in phosphate buffer (0.2 M, 6 M Gn·HCl, pH 3), acetylacetone (25 equiv) and MPAA (50 mM) were then added to the peptide solution[3]. The reaction mixture was incubated at room temperature for 3 h. The purification of ubiquitin(1-46)-MPAA (**6a**) was conducted by RP-HPLC using C4 column with a gradient of 5-70% B (0.1% TFA in MeCN) over 20 min to obtain ubiquitin(1-46)-MPAA (**6a**) in 30% yield (mass

Found 5334.4 Da, Calcd. 5334.8 Da) The results are shown in **Figure S2**.

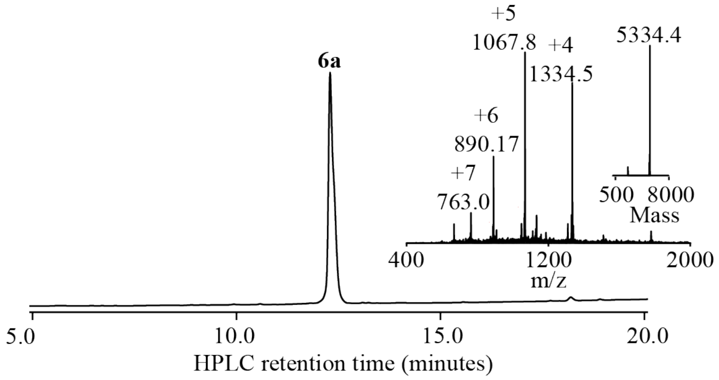

Figure S2. HPLC trace and ESI-MS of ubiquitin (1-46)- MPAA (**6a**)

(13) *The synthesis of ubiquitin(47-76)(G47C)* ***(6b)***

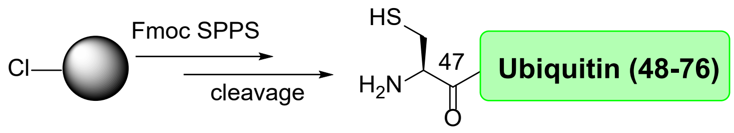

Figure S3. Chemical synthesis of ubiquitin(47-76)(G47C) (**6b**)

The synthesis of ubiquitin(47-76)(G47C) (**6b**) was carried out on 2-chlorotrityl chloride resin (0.25 mmol scale, 0.5 mmol/g) on automated Fmoc-peptide synthesizer. Upon synthesis completion the peptide was cleaved and treated as described previously. The crude ubiquitin(47-76)(G47C) was purified by Prep RP-HPLC (C18 column) to obtain **6b** in ~ 10% yield (mass Found 3442.8 Da, Calcd. 3443.8 Da). The results are shown in **Figure S4**.

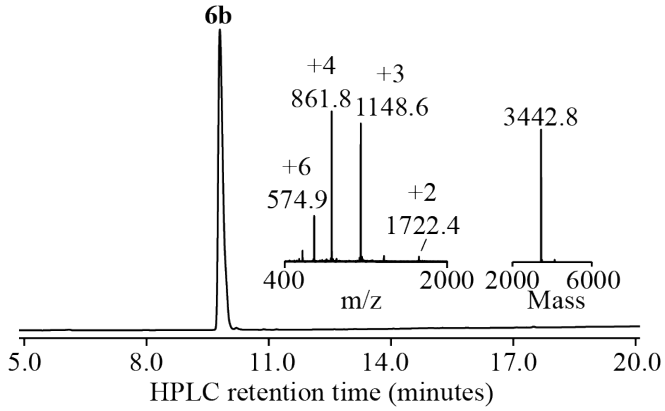

Figure S4. HPLC trace and ESI-MS ubiquitin(47-76)(G47C), **6b.**

14) The native chemical ligation (NCL) of ubiquitin(1-46)-MPAA thioester ***(6a)*** *and*

*ubiquitin(47-76)(G47C) (**6b**).*

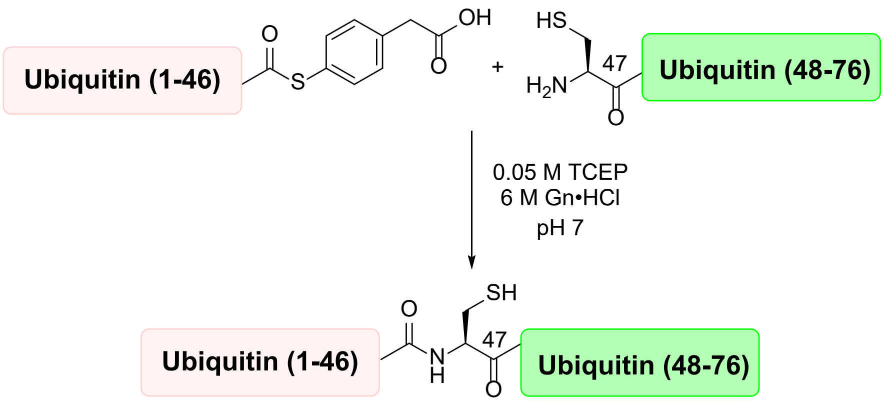

Figure S5. Chemical synthesis of ubiquitin(G47C) (**6**) by NCL

Ubiquitin(1-46)-MPAA (**6a**, 11.4 mg, 2.1 μmol, 1mM) and ubiquitin(47-76)(G47C) (**6b**, 9.8 mg, 2.8 μmol, 1.3 mM) were dissolved in 2.13 mL of degassed PB (0.1 M, 6 M Gn·HCl, 0.05 M TCEP, at pH 7) and incubated at 37 ºC for 3 h. The reaction progress was monitored by analytical RP-HPLC (C4 column) with a gradient of 5-70% B (0.1% TFA in MeCN) over 20 min at 220 nm (Figure S8). The purification was conducted by semi-prep RP-HPLC (C4 column) to afford the corresponding protein ubiquitin(G47C) (**6**) in ~ 28% yield (mass Found 8607.1 Da, Calcd. 8608.6 Da). The results are shown in **Figure S6.**

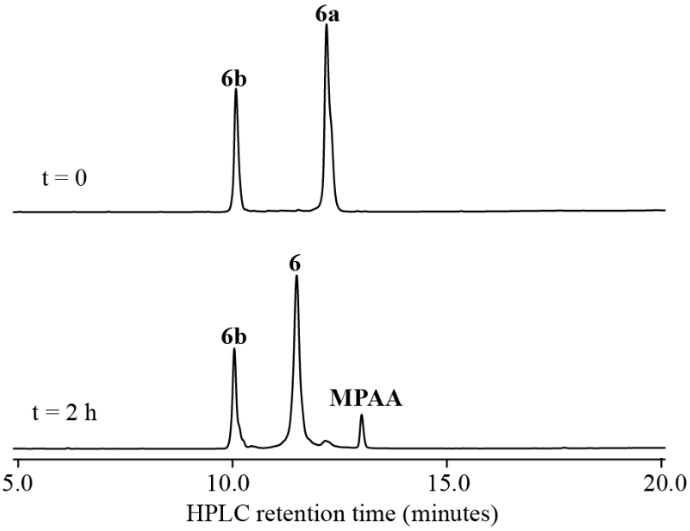

Figure S6. Chemical synthesis of ubiquitin(G47C) (**6**) by NCL.

*N-ethynyl-N,4-dimethylbenzenesulfonamide (**2a**)*[1]

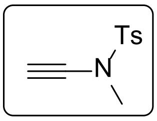

White solid, 89%, 372.0 mg, purified by flash chromatography using PE/EA (8:1) as eluent, Rf = 0.5 in PE/EA (5:1) ^{1}H NMR (400 MHz, $CDCl_3$) δ 7.80 (d, *J* = 8.3 Hz, 2H), 7.37 (d, *J* = 8.3 Hz, 2H), 3.06 (s, 3H), 2.69 (s, 1H), 2.46 (s, 3H) ^{3}C NMR (101 MHz, $CDCl_3$) δ 144.9, 133.3, 130.0, 127.8, 77.6, 57.5, 38.7, 21.7.

*N,4-dimethyl-N-((trimethylsilyl)ethynyl)benzenesulfonamide (**2b**)*[4]

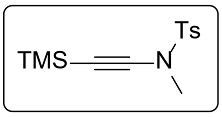

White solid, 70%, 394.0 mg, purified by flash chromatography using PE/EA (8:1) as eluent, Rf = 0.6 in PE/EA (5:1) ^{1}H NMR (400 MHz, $CDCl_3$) δ 7.63 (d, *J* = 8.1 Hz, 2H), 7.21 (d, *J* = 8.1 Hz, 2H), 2.89 (s, 3H), 2.31 (s, 3H), 0.00 (s, 9H) ^{13}C NMR (101 MHz, $CDCl_3$) δ 144.7, 133.2, 129.6, 127.9, 96.6, 71.3, 39.0, 21.6, 0.0.

*4-(tert-butyl)-N-ethynyl-N-methylbenzenesulfonamide (**2c**)*[1]

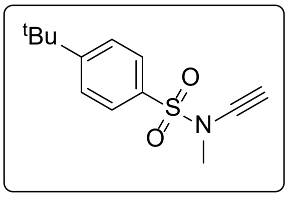

Colourless oil, 85%, 426.7 mg, purified by flash chromatography using PE/EA (6:1) as eluent, Rf = 0.4 in PE/EA (5:1) ^{1}H NMR (400 MHz, $CDCl_3$) δ 7.84 (d, *J* = 8.6 Hz, 2H), 7.58 (d, *J* = 8.6 Hz, 2H), 3.08 (d, *J* = 2.7 Hz, 3H), 2.70 (s, 1H), 1.36 (s, 9H) ^{13}C NMR (101 MHz, $CDCl_3$) δ 157.8, 133.4, 127.7, 126.2, 77.7, 57.5, 38.8, 35.3, 31.0.

*N-ethynyl-N-(2-hydroxyethyl)-4-methylbenzenesulfonamide (**2d**)*[5]

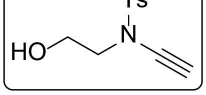

White solid, 85%, 406.3 mg, purified by flash chromatography using PE/EA (10:1) as eluent, Rf = 0.5 in PE/EA (5:1) ^{1}H NMR (400 MHz, $CDCl_3$) δ 8.14 (s, 1H), 7.68 (d, *J* = 15.0 Hz, 2H), 7.37 (d, *J* = 14.9 Hz, 2H), 3.62 (t, *J* = 7.4 Hz, 2H), 3.35 (t, *J* = 7.4 Hz, 2H), 2.43 (s, 3H) ^{13}C NMR (101 MHz, $CDCl_3$) δ 145.2, 134.3, 130.0, 127.9, 76.0, 60.0, 59.5, 53.7, 21.8.

*N-ethynyl-1,1,1-trifluoro-N-(2-hydroxyethyl)methanesulfonamide (**2e**)*

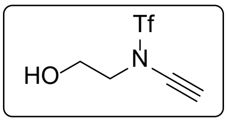

Yellow oil, 95%, 412.3 mg, purified by flash chromatography using PE/EA (4:1) as eluent, Rf = 0.4 in PE/EA (2:1) ^{1}H NMR (400 MHz, $CDCl_3$) δ 3.95 (d, *J* = 4.6 Hz, 2H), 3.73 (t, *J* = 5.2 Hz, 2H), 2.85 (s, 1H), 2.50 (s, 1H) ^{13}C NMR (101 MHz, $CDCl_3$) δ 119.6 (q, J = 320.0 Hz), 71.6, 59.9, 59.9, 58.8, 55.1. HRMS (ESI-TOF) m/z: $C_5H_7F_3NO_3S^+$ $[M + H]^+$ Calcd. 218.0093; Found: 218.0085.

*N-ethynyl-1,1,1-trifluoro-N-phenethylmethanesulfonamide (**2f**)*

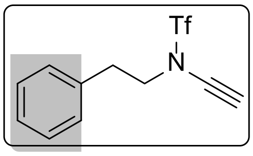

White solid, 92%, 509.7 mg, purified by flash chromatography using PE/EA (10:1) as eluent, Rf = 0.5 in PE/EA (5:1) ^{1}H NMR (400 MHz, $CDCl_3$) δ 7.33 (t, *J* = 7.2 Hz, 2H), 7.27 (d, *J* = 7.2 Hz, 1H), 7.22 (d, *J* = 8.4 Hz, 2H), 3.83 – 3.72 (m, 2H), 3.12 – 3.04 (m, 2H), 2.83 (s, 1H) ^{13}C NMR (101 MHz, $CDCl_3$) δ 136.1, 128.9, 128.85, 127.26, 119.6 (q, J = 320.0 Hz), 71.78, 60.15, 54.62, 34.37. HRMS (ESI-TOF) m/z: $C_{11}H_{11}F_3NO_2S^+$ $[M + H]^+$ Calcd. 278.0457; Found: 278.0467.

*tert-butyl (2-((N-ethynyl-1,1,1-trifluoromethyl)sulfonamido)ethyl)carbamate (**2g**)*

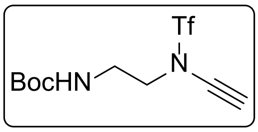

Yellow oil, 95%, 601.0 mg, purified by flash chromatography using PE/EA (5:1) as eluent, Rf = 0.4 in PE/EA (3:1) ^{1}H NMR (400 MHz, $CDCl_3$) δ 4.93 (s, 1H), 3.72 (s, 2H), 3.47 (d, *J* = 5.0 Hz, 2H), 2.83 (s, 1H), 1.45 (s, 9H) ^{13}C NMR (101 MHz, $CDCl_3$) δ 155.7, 119.6 (q, J = 320.0 Hz), 80.1, 71.6, 59.9, 52.7, 38.5, 28.3. HRMS (ESI-TOF) m/z: $C_{10}H_{15}F_3N_2NaO_4S^+$ $[M + Na]^+$ Calcd. 339.0597; Found: 339.0599.

*N-(2-((N-ethynyl-1,1,1-trifluoromethyl)sulfonamido)ethyl)-2-oxo-2H-chromene-3-carboxamide (**2h**)*

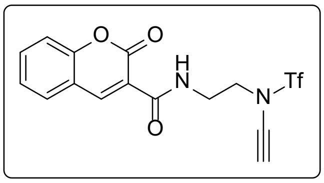

Yellow solid, 92%, 714.4 mg, purified by flash chromatography using PE/EA (2:1) as eluent, Rf = 0.5 in PE/EA (1:1) ^{1}H NMR (400 MHz, $CDCl_3$) δ 9.14 (s, 1H),

8.92 (s, 1H), 7.70 (t, J = 8.9 Hz, 2H), 7.48 – 7.33 (m, 2H), 3.85 (dd, J = 10.2, 4.6 Hz, 4H), 2.88 (d, J = 6.1 Hz, 1H) ^{13}C NMR (101 MHz, $CDCl_3$) δ 162.4, 161.3, 154.6, 148.8, 134.4, 130.0, 125.4, 119.6 (q, J = 320.0 Hz), 118.5, 117.8, 116.7, 71.6, 60.2, 52.1, 37.9. HRMS (ESI-TOF) m/z: $C_{15}H_{12}F_3N_2O_5S^+$ $[M + H]^+$ Calcd. 389.0414; Found: 389.0416.

*2-((N-ethynyl-1,1,1-trifluoromethyl)sulfonamido)ethyl 2-(1-(4-chlorobenzoyl)-5-methoxy-2-methyl-1H-indol-3-yl)acetate (**2i**)*

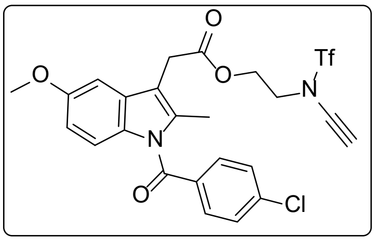

White solid, 90%, 501.3 mg, purified by flash chromatography using PE/EA (3:1) as eluent, Rf = 0.4 in PE/EA (2:1) ^{1}H NMR (400 MHz, $CDCl_3$) δ 7.65 (d, J = 8.2 Hz, 2H), 7.45 (d, J = 8.2 Hz, 2H), 6.95 (s, 1H), 6.87 (d, J = 9.0 Hz, 1H), 6.66 (d, J = 8.8 Hz, 1H), 4.52 – 4.27 (m, 2H), 3.82 (s, 5H), 3.72 (s, 2H), 2.81 (s, 1H), 2.37 (s, 3H) ^{13}C NMR (101 MHz, $CDCl_3$) δ 170.5, 168.3, 156.1, 139.2, 136.2, 133.9, 131.2, 130.8, 130.6, 129.1, 119.6 (q, J = 322.0 Hz), 115.0, 111.9, 111.8, 101.1, 71.2, 60.2, 55.7, 52.1, 29.9, 13.3. HRMS (ESI-TOF) m/z: $C_{24}H_{21}ClF_3N_2O_6S^+$ $[M + H]^+$ Calcd. 557.0755; Found: 557.0758.

*2-((N-ethynyl-1,1,1-trifluoromethyl)sulfonamido)ethyl 5-((3aS,4S,6aR)-2-oxohexahydro-1H-thieno[3,4-d]imidazol-4-yl)pentanoate (**2j**)*

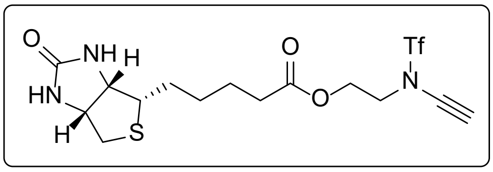

White solid, 80%, 354.4 mg, purified by flash chromatography using DCM/MeOH (200:1) as eluent, Rf = 0.6 in DCM/MeOH (10:1) ^{1}H NMR (400 MHz, $CDCl_3$) δ 6.20 (s, 1H), 5.79 (s, 1H), 4.55 – 4.46 (m, 1H), 4.38 (t, J = 4.9 Hz, 2H), 4.35 – 4.25 (m, 1H), 3.84 (t, J = 4.9 Hz, 2H), 3.16 (d, J = 4.6 Hz, 1H), 2.91 (dd, J = 11.4, 6.2 Hz, 2H), 2.74 (d, J = 12.8 Hz, 1H), 2.39 (t, J = 7.4 Hz, 2H), 1.80 – 1.57 (m, 4H), 1.47 (dd, J = 15.1, 7.5 Hz, 2H) ^{13}C NMR (101 MHz, $CDCl_3$) δ 173.2, 164.0, 119.5 (q, J = 322.0

Hz), 71.3, 62.0, 60.2, 60.2, 59.7, 55.5, 52.1, 40.6, 33.6, 28.3, 28.2, 24.5. HRMS (ESI-TOF) m/z: $C_{15}H_{21}F_3N_3O_5S_2^+$ [M + H]$^+$ Calcd. 444.0869; Found: 444.0876.

*N-ethynyl-1,1,1-trifluoro-N-(prop-2-yn-1-yl)methanesulfonamide (**2k**)*

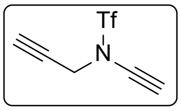

Yellow oil, 95%, 399.0 mg, purified by flash chromatography using PE/EA (10:1) as eluent, Rf = 0.5 in PE/EA (5:1) ^{1}H NMR (400 MHz, $CDCl_3$) δ 4.32 (d, *J* = 1.9 Hz, 2H), 2.81 (s, 1H), 2.49 (d, *J* = 2.2 Hz, 1H) ^{13}C NMR (101 MHz, $CDCl_3$) δ 119.4 (q, J = 322.0 Hz), 76.18, 74.19, 71.04, 60.54, 43.34. HRMS (ESI-TOF) m/z: $C_6H_5F_3NO_2S^+$ [M + H]$^+$ Calcd. 211.9988; Found: 212.0003.

*(E)-4-oxo-4-phenylbut-2-en-2-yl-3-((N-ethynyl-1,1,1-trifluoromethyl)sulfonamido)propanoate (**31^d**)*

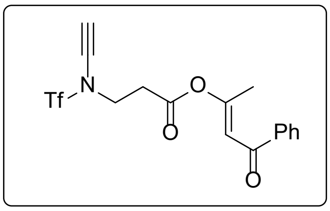

Yellow oil, 79% yield, R$_f$ = 0.6 (PE/EA = 4:1). ^{1}H NMR (400 MHz, Chloroform-*d*) δ 7.91 (d, *J* = 7.5 Hz, 2H), 7.55 (t, *J* = 7.3 Hz, 1H), 7.46 (t, *J* = 7.5 Hz, 2H), 6.82 (s, 1H), 3.99 (t, *J* = 7.0 Hz, 2H), 3.00 (t, *J* = 7.1 Hz, 2H), 2.89 (s, 1H), 2.41 (s, 3H). ^{13}C NMR (100 MHz, $CDCl_3$) δ 190.2, 167.1, 162.9, 138.6, 133.1, 128.7, 128.2, 119.7 (q, *J* = 322.0 Hz), 114.1, 71.3, 60.8, 48.7, 33.3, 18.8. HRMS (ESI-TOF) m/z: $C_{16}H_{14}F_3NNaO_5S^+$ [M + Na]$^+$ Calcd. 412.0437; Found:412.0432.

*ethyl (tert-butoxycarbonyl)-L-alanyl-L-cysteinate (**1a**)*

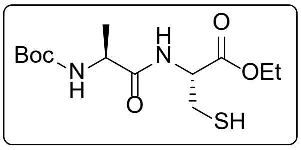

White solid, 91%, 2.9 g, purified by flash chromatography using PE/EA (2:1) as eluent, Rf = 0.5 in PE/EA (1:1) ^{1}H NMR (400 MHz, $CDCl_3$) δ 7.45 (s, 1H), 5.62 (d, *J* = 7.1 Hz, 1H), 4.94 – 4.65 (m, 1H), 4.27 (s, 1H), 4.15 (q, *J* = 7.1 Hz, 2H), 3.28 – 2.88 (m, 2H), 1.37 (s, 9H), 1.31 (d, *J* = 7.0 Hz, 3H), 1.21 (t, *J* = 7.1 Hz, 3H) ^{13}C NMR (101 MHz, $CDCl_3$) δ 173.2, 170.0, 155.7, 79.9, 61.7, 52.1, 49.8, 40.7, 28.3, 18.2, 14.0. HRMS (ESI-TOF) m/z: $C_{13}H_{25}N_2O_5S^+$ [M + H]$^+$ Calcd. 321.1479; Found: 321.1479.

N^2-(N-(((S)-2-amino-4-(tert-butoxy)-4-oxobutanoyl)-D-phenylalanyl)-S-trityl-L-cysteinyl)-N^w-((2,2,4,6,7-pentamethyl-2,3-dihydrobenzofuran-5-yl)sulfonyl)-L-arginylglycine

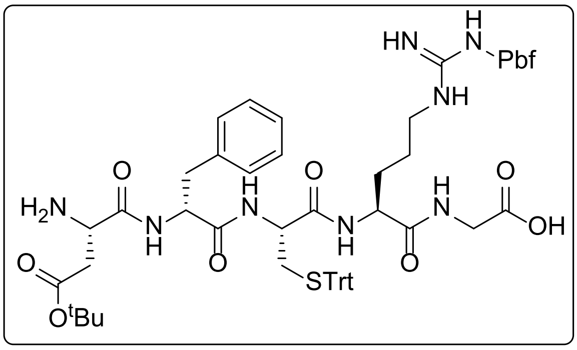

White solid, 80%, 917.6 mg, purified by recrystallization. ^{1}H NMR (400 MHz, DMSO) δ 8.55 (s, 1H), 8.55 (s, 1H), 7.89 (d, *J* = 3.4 Hz, 2H), 7.50 – 7.28 (m, 13H), 7.22 (dd, *J* = 14.8, 8.6 Hz, 6H), 7.20 – 7.02 (m, 5H), 6.62 (s, 3H), 4.61 (s, 1H), 4.27 (dd, *J* = 17.1, 9.7 Hz, 2H), 3.68 (s, 1H), 3.61 (s, 2H), 3.02 (d, *J* = 4.4 Hz, 3H), 2.94 (s, 2H), 2.82 (d, *J* = 8.3 Hz, 1H), 2.62 – 2.44 (m, 6H), 2.40 (d, *J* = 24.6 Hz, 5H), 2.00 (s, 3H), 1.91 (s, 4H), 1.73 (d, *J* = 16.4 Hz, 2H), 1.53 (s, 3H), 1.40 (s, 6H), 1.37 (s, 9H) ^{13}C NMR (101 MHz, DMSO) δ 172.6, 172.5, 171.4, 171.2, 171.2, 169.7, 157.9, 156.7, 144.7, 137.7, 137.7, 134.7, 131.9, 129.7, 129.6, 128.5, 127.2, 126.8, 124.8, 116.8, 86.7, 81.1, 66.4, 54.5, 54.4, 52.7, 52.3, 50.9, 43.0, 38.3, 34.2, 29.8, 28.8, 28.2, 25.6, 21.6, 21.6, 19.4, 18.1, 12.7. HRMS (ESI-TOF) m/z: $C_{60}H_{75}N_8O_{11}S_2^+$ [M + H]$^+$ Calcd. 1147.4991; Found: 1147.4994.

*2-((2S,5R,8R,11S)-5-benzyl-11-(3-guanidinopropyl)-8-(mercaptomethyl)-3,6,9,12,15-pentaoxo-1,4,7,10,13-pentaazacyclopentadecan-2-yl)acetic acid (**1o**)*

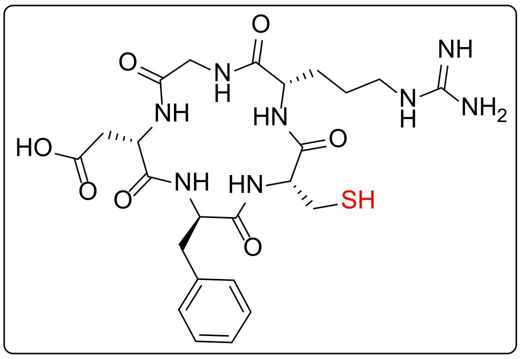

White solid, 99%, 572.2 mg, purified by recrystallization. ^{1}H NMR (400 MHz, DMSO) δ 9.01 (s, 1H), 8.25 (d, *J* = 3.8 Hz, 1H), 8.15 (t, *J* = 8.1 Hz, 2H), 7.96 (d, *J* = 8.2 Hz, 2H), 7.56 (d, *J* = 5.1 Hz, 1H), 7.22 – 6.95 (m, 5H), 4.65 (d, *J* = 6.0 Hz, 1H), 4.52 (d, *J* = 7.0 Hz, 1H), 4.36 – 4.18 (m, 1H), 4.19 – 3.93 (m, 2H), 3.32 – 3.19 (m, 1H), 3.09 (s, 2H), 3.00 (dd, *J* = 13.4, 7.6 Hz, 1H), 2.82 (dd, *J* = 13.5, 6.2 Hz, 1H),

2.70 (dd, J = 15.0, 9.2 Hz, 3H), 2.52 (s, 1H), 2.43 – 2.30 (m, 1H), 2.20 (t, J = 8.3 Hz, 1H), 1.80 (s, 1H), 1.53 (dd, J = 12.8, 9.0 Hz, 1H), 1.48 – 1.30 (m, 2H) ^{13}C NMR (101 MHz, DMSO) δ 172.2, 171.4, 171.3, 170.6, 170.5, 169.8, 157.1, 137.9, 129.7, 128.6, 126.7, 65.4, 57.6, 54.7, 52.7, 49.4, 43.7, 40.8, 37.4, 35.8, 28.4, 26.3. HRMS (ESI-TOF) m/z: $C_{24}H_{35}N_8O_7S^+$ $[M + H]^+$ Calcd. 579.2344; Found: 579.2354.

3. Optimization of Reaction Conditions for Cys Modification with Ynamides

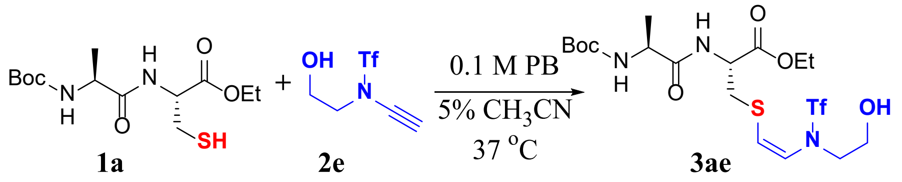

entry	pH	1a (mM/μM)	2e (equiv)	time	conversion	yield[a]
1	6.0	1 mM	2.0	1 h	< 1%	-
2	7.0	1 mM	2.0	1 h	< 1%	-
3	7.5	1 mM	2.0	1 h	10%	-
4	8.0	1 mM	2.0	20 min	> 99%	95%
5	8.0	1 mM	3.0	15 min	> 99%	92%
6	8.0	1 mM	5.0	5 min	> 99%	92%
7[b]	8.0	1 mM	2.0	50 min	> 99%	95%
8	8.0	100 μM	2.0	40 min	> 99%	92%
9	8.0	50 μM	2.0	140 min	> 99%	91%
10	8.0	10 μM	2.0	80 min	80%	72%
11	8.0	10 μM	5.0	20 min	> 99%	93%

Table S1. Optimization of the conjugation of ynamide with cysteine-containing peptide. [f]Isolated yield. [g]Room temperature.

(1) *Preliminary study of the modification of peptide **1a** with ynamide **2a***

The peptide **1a** (0.01 mmol) and ynamide **2a** (0.02 mmol, 2 equiv) were dissolved in 10 mL PB (0.1 M, pH 8.0) and 10 mL CH3CN at 37 ° C. The reaction progress was monitored by HPLC (using Jupiter 5 μm C18 4.6 × 250 mm^2) with a gradient of 10-95% solvent B in 30 min at 220 nm and LC-MS. The peptide conjugate **3aa** was isolated in 85% after 24 h.

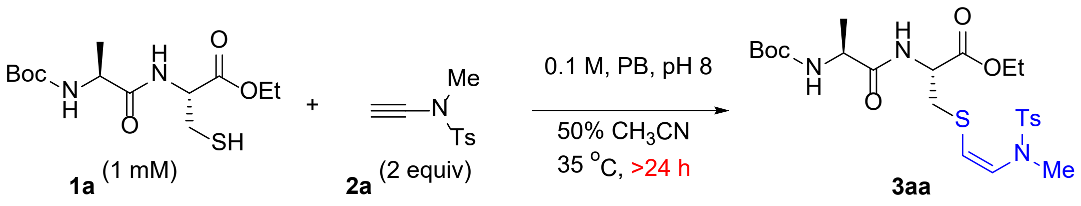

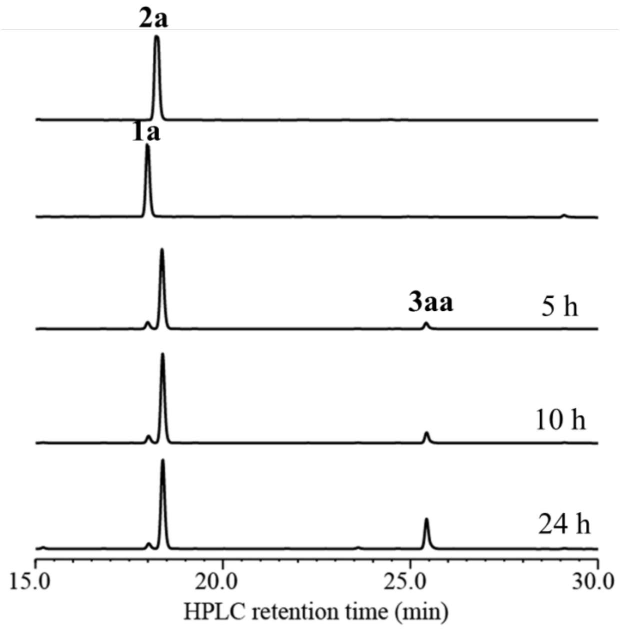

Figure S7. Preliminary study of the modification of peptide **1a** with ynamide **2a**

(2) *The modification of peptide **1a** with ynamide **2b***

The peptide **1a** (0.01 mmol) and ynamide **2b** (0.02 mmol, 2 equiv) were dissolved in 10 mL PB (0.1 M, pH 8.0) and 3 mL CH_3CN at 37 °C. The reaction progress was monitored by HPLC (using Jupiter 5 µm C18 4.6 × 250 mm^2) with a gradient 30-95% solvent B in 30 min at 220 nm and LC-MS. The peptide conjugate **3ab** was isolated in 89% after 24 h.

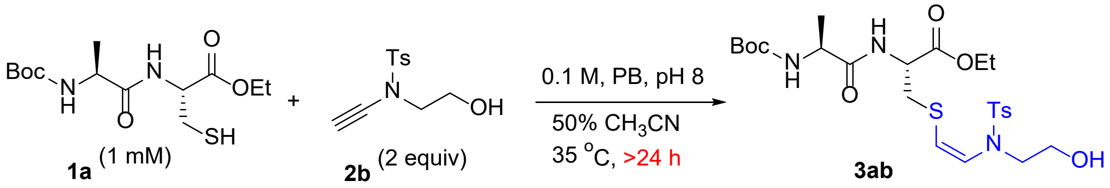

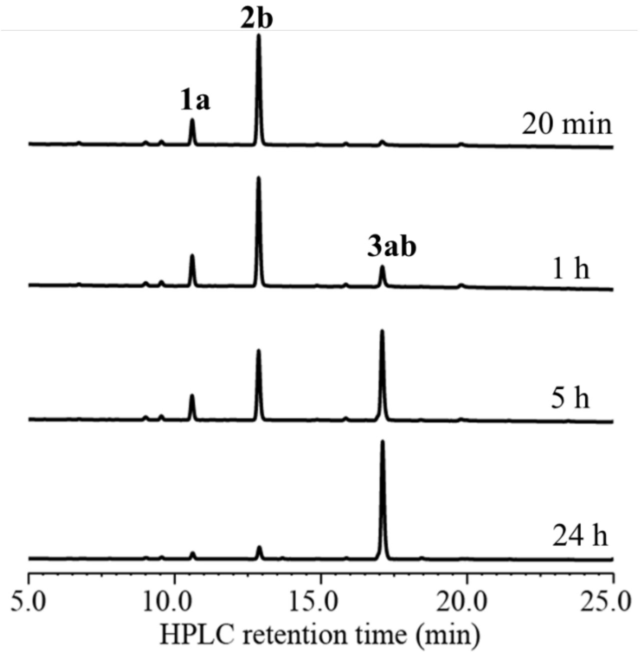

Figure S8. Preliminary study of the modification of peptide **1a** with ynamide **2b**

(3) *The modification of peptide **1a** with ynamide **2c***

The peptide **1a** (0.01 mmol) and ynamide **2c** (0.02 mmol, 2 equiv) were dissolved in 10 mL PB (0.1 M, pH 8.0) and 10 mL CH_3CN at 37 °C. The reaction progress was monitored by HPLC (using Jupiter 5 µm C18 4.6 × 250 mm^2) with a gradient 30-95% solvent B in 30 min at 220 nm and LC-MS. No new products were detected within 24 h.

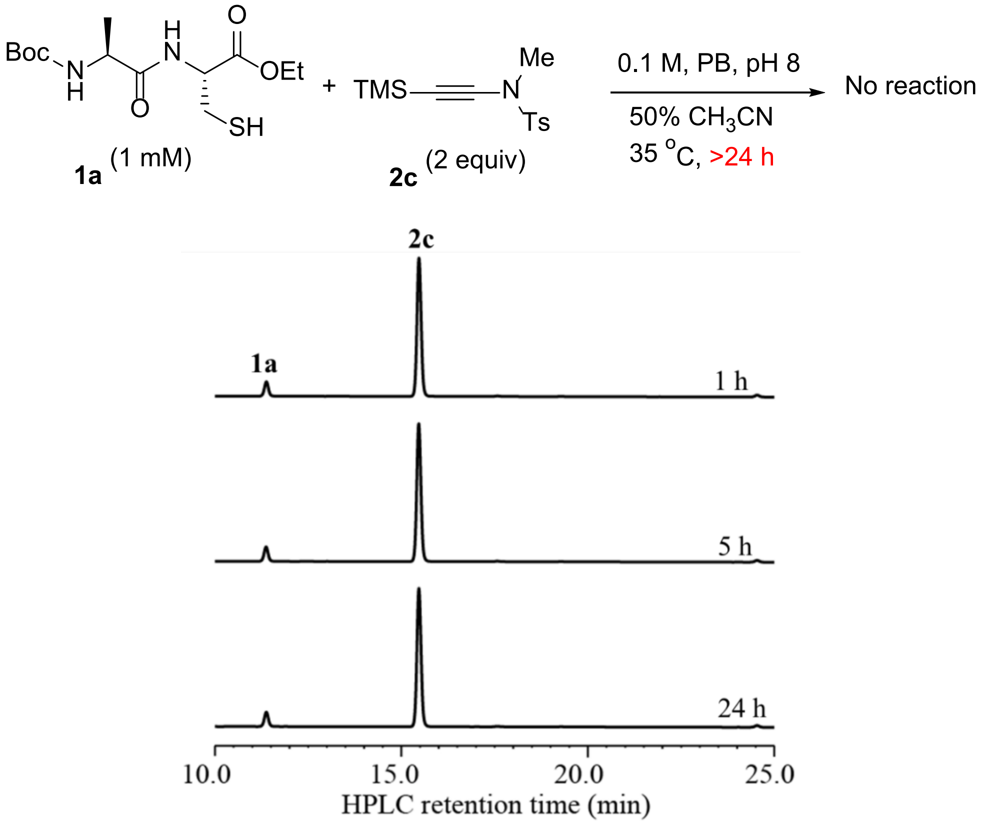

Figure S9. Preliminary study of the modification of peptide **1a** with ynamide **2c**

(4) *The modification of peptide **1a** with ynamide **2d***

The peptide **1a** (0.01 mmol) and ynamide **2d** (0.02 mmol, 2 equiv) were dissolved in 10 mL PB (0.1 M, pH 8.0) and 10 mL CH_3CN at 37 °C. The reaction progress was monitored by HPLC (using Jupiter 5 μm C18 4.6 × 250 mm^2) with a gradient of 10-95% solvent B in 30 min at 220 nm and LC-MS. No new product was detected within 24 h.

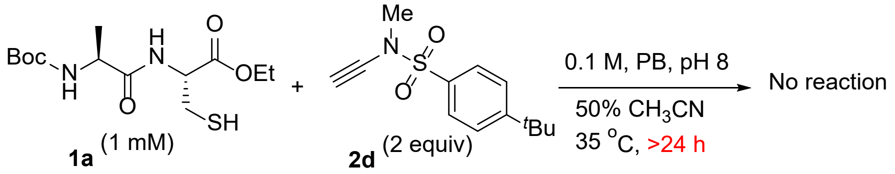

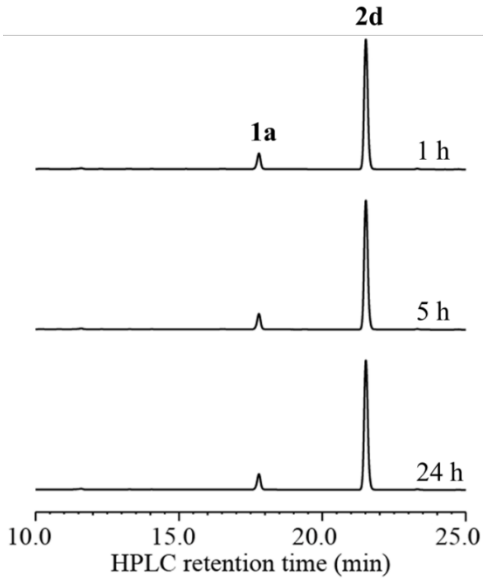

Figure S10. Preliminary study of the modification of peptide **1a** with ynamide **2d**

(5) *The modification of peptide **1a** with ynamide **2e***

The peptide **1a** (0.01 mmol) and ynamide **2e** (0.02 mmol, 2 equiv) were dissolved in 10 mL PB (0.1 M, pH 8.0) and 0.5 mL CH_3CN at 37 °C. The reaction progress was monitored by HPLC (using Jupiter 5 μm C18 4.6 × 250 mm^2) with a gradient of 10-95% solvent B in 30 min at 220 nm and LC-MS. The peptide conjugate **3ae** was isolated in 95% after 20 min.

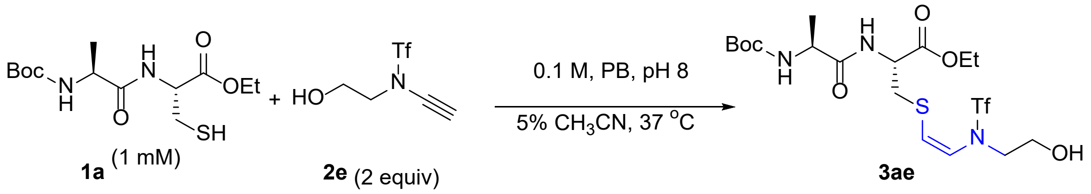

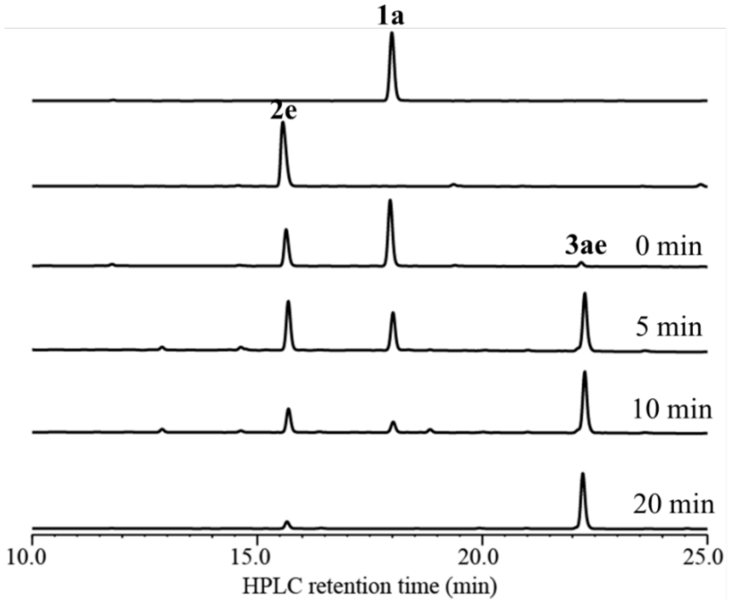

Figure S11. The modification reaction of peptide **1a** with ynamide **2e**

(6) *The modification of peptide **1a** with ynamide **2f***

The peptide **1a** (0.01 mmol) and ynamide **2f** (0.02 mmol, 2 equiv) were dissolved in 10 mL PB (0.1 M, pH 8.0) and 10 mL CH_3CN at 37 °C. The reaction progress was monitored by HPLC (using Jupiter 5 μm C18 4.6 × 250 mm^2) with a gradient of 10-95% solvent B in 30 min at 220 nm and LC-MS. The peptide conjugate **3af** was isolated in 95% after 20 min.

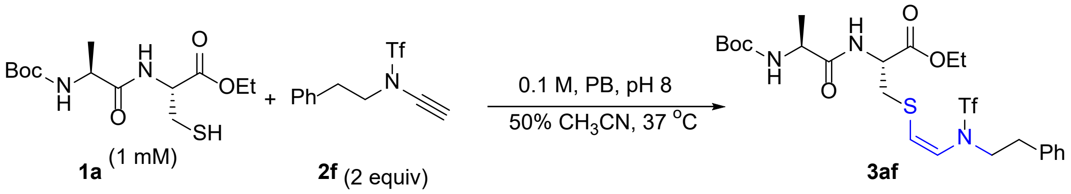

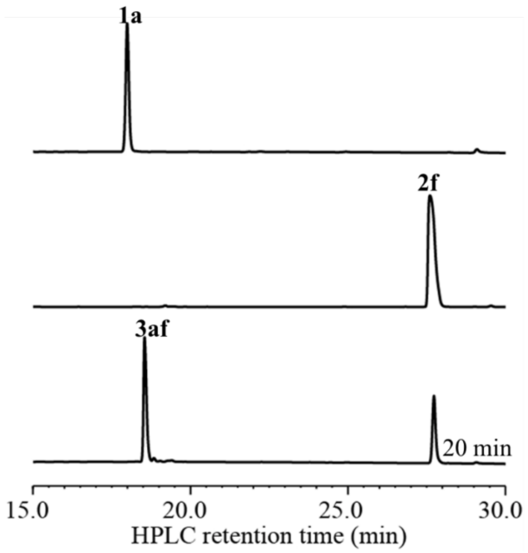

Figure S12. The modification reaction of peptide **1a** with ynamide **2f**

(7) *The modification of peptide* ***1a*** *with ynamide* ***2g***

The peptide **1a** (0.01 mmol) and ynamide **2g** (0.02 mmol, 2 equiv) were dissolved in 10 mL PB (0.1 M, pH 8.0) and 4 mL CH_3CN at 37 °C. The reaction progress was monitored by HPLC (using Jupiter 5 μm C18 4.6 × 250 mm^2) with a gradient of 10-95% solvent B in 30 min at 220 nm and LC-MS. The peptide conjugate **3ag** was isolated in 94% after 20 min.

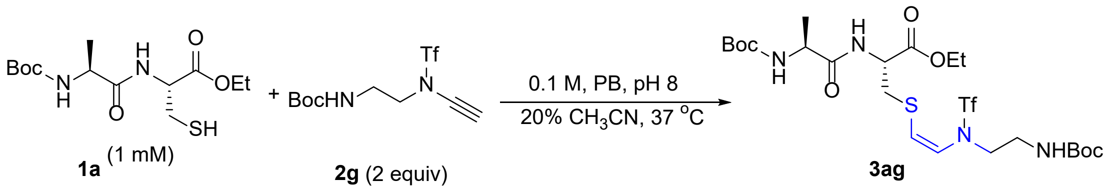

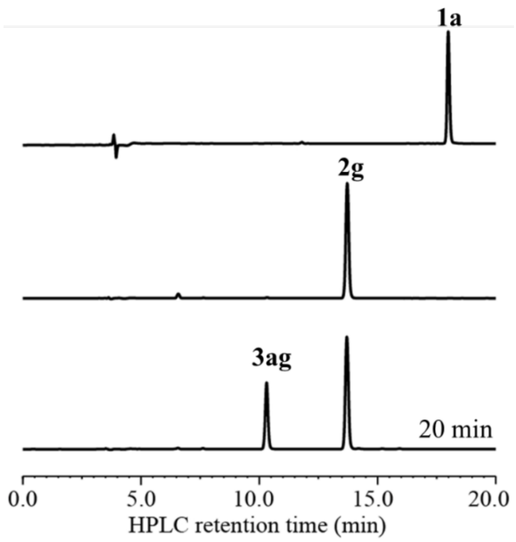

Figure S13. The modification reaction of peptide **1a** with ynamide **2g**

(8) *The effect of pH on peptide modification with ynamide* ***2e***

The peptide **1a** (0.01 mmol) and ynamide **2e** (0.02 mmol, 2 equiv) were dissolved in 10 mL PB (0.1 M), which pH were 5.0, 6.0, 7.0, and 8.0, respectively, and 0.5 mL CH_3CN at 37 °C. The reaction progress was monitored by HPLC (using Jupiter 5 μm C18 4.6 × 250 mm^2) with a gradient of 10-95% solvent B in 30 min at 220 nm and LC-MS.

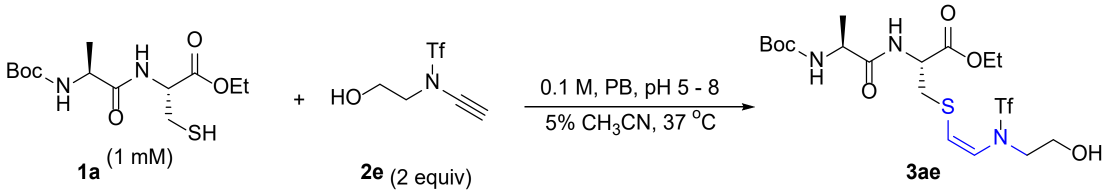

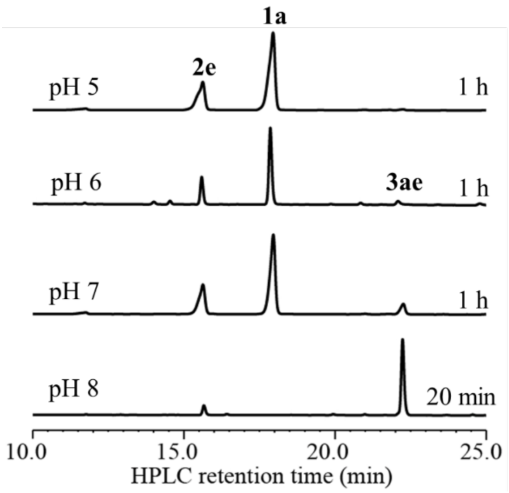

Figure S14. The effect of pH on peptide modification with ynamide **2e**

(9) *The effect of the loading of ynamide **2e** on peptide modification*

The peptide **1a** (0.01 mmol) and ynamide **2e** (n equiv) were dissolved in 10 mL PB (0.1 M, pH 8.0) and 0.5 mL CH_3CN at 37 °C. The reaction progress was monitored by HPLC (using Jupiter 5 µm C18 4.6 × 250 mm^2) with a gradient of 10-95% solvent B in 30 min at 220 nm and LC-MS. (n = 2.0, 3.0, 5.0, respectively).

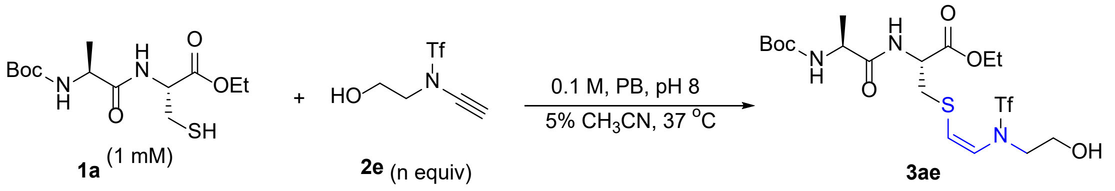

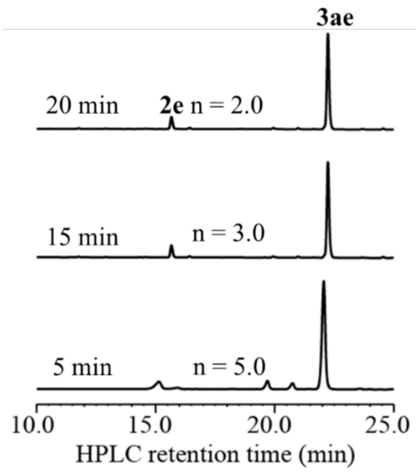

Figure S15. The effect of the loading of ynamide **2e** on peptide modification

(10) *The effect of the concentration of reagents on peptide modification*

The peptide **1a** (0.01 mmol) and ynamide **2e** (n equiv) were dissolved in x mL PB (0.1 M, pH 8.0) and y mL CH_3CN at 37 °C. The reaction progress was monitored by HPLC (using Jupiter 5 μm C18 4.6 × 250 mm^2) with a gradient of 10-95% solvent B in 30 min at 220 nm and LC-MS. (n = 2.0, 3.0, 5.0, respectively).

From top to bottom: 1. n = 2.0, x = 95, y = 5

2. n = 2.0, x = 190, y = 10

3. n = 2.0, x = 950, y = 50

4. n = 5.0, x = 950, y = 50

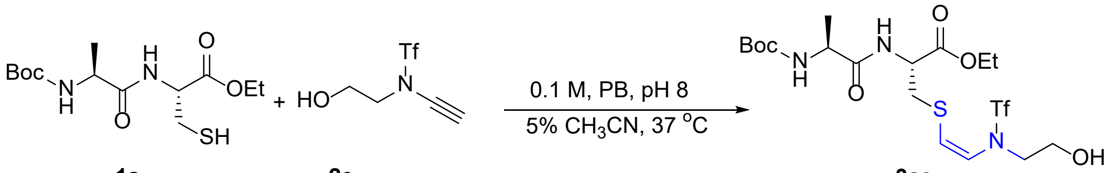

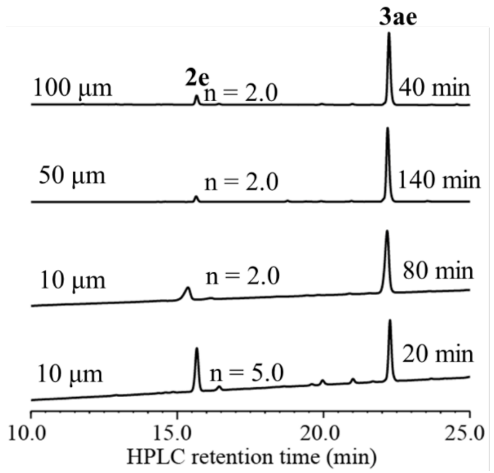

Figure S16. The effect of the concentration of reagents on peptide modification

4. Systematic Study of Cys Modification by Ynamides in the Presence of Other Amino Acid Residues

1) *Chemoselectivity study of ynamide **2e** in the presence of peptide **1b***

The peptide **1b** (0.01 mmol) and ynamide **2e** (0.02 mmol, 2 equiv) were dissolved in 10 mL PB (0.1 M, pH 8.0) and 0.5 mL CH_3CN at 37 °C. The reaction progress was monitored by HPLC (using Jupiter 5 μm C18 4.6 × 250 mm^2) with a gradient of 10-95% solvent B in 30 min at 220 nm and LC-MS. No product was detected within 5 h.

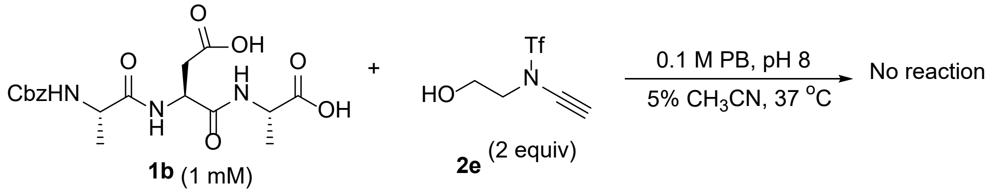

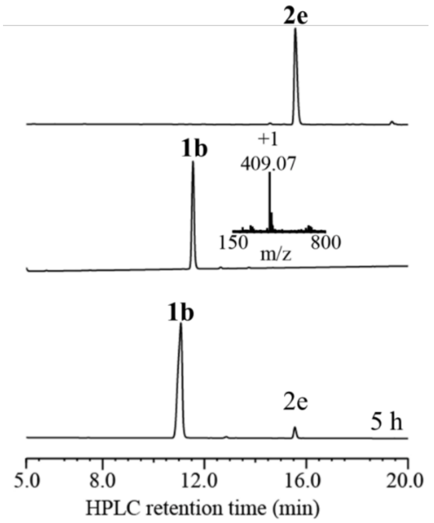

Figure S17. Chemoselectivity study of ynamide **2e** in the presence of peptide **1b**

2) *Chemoselectivity study of ynamide* ***2e*** *in the presence of peptide* ***1c***

The peptide **1c** (0.01 mmol) and ynamide **2e** (0.02 mmol, 2 equiv) were dissolved in 10 mL PB (0.1 M, pH 8.0) and 0.5 mL CH_3CN at 37 °C. The reaction progress was monitored by HPLC (using Jupiter 5 μm C18 4.6 × 250 mm^2) with a gradient of 10-95% solvent B in 30 min at 220 nm and LC-MS. No product was detected within 5 h.

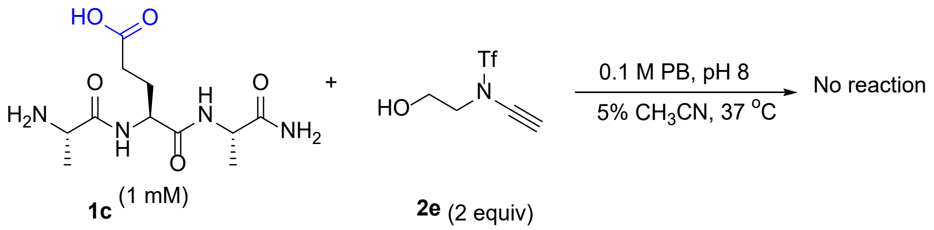

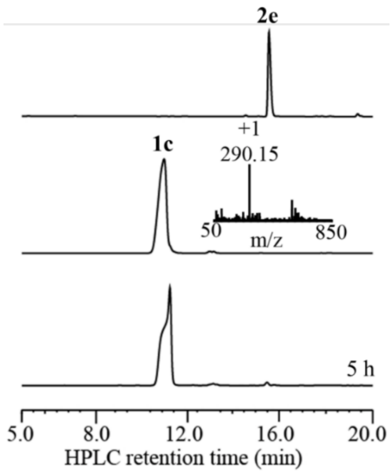

Figure S18. Chemoselectivity study of ynamide **2e** in the presence of peptide **1c**

3) *Chemoselectivity study of ynamide* ***2e*** *in the presence of peptide* ***1d***

The peptide **1d** (0.01 mmol) and ynamide **2e** (0.02 mmol, 2 equiv) were dissolved in 10 mL PB (0.1 M, pH 8.0) and 0.5 mL CH_3CN at 37 °C. The reaction progress was monitored by HPLC (using Jupiter 5 μm C18 4.6 × 250 mm^2) with a gradient of 10-95% solvent B in 30 min at 220 nm and LC-MS. No product was detected within 5 h.

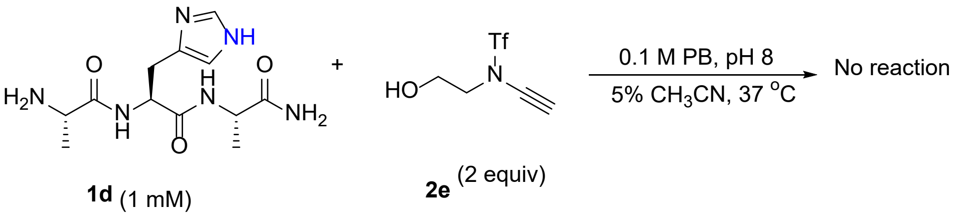

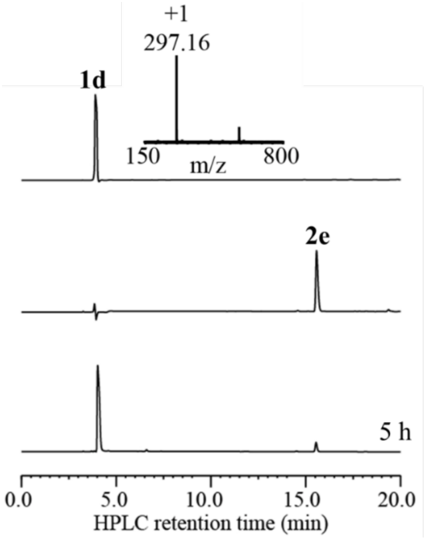

Figure S19. Chemoselectivity study of ynamide **2e** in the presence of peptide **1d**

4) *Chemoselectivity study of ynamide **2e** in the presence of peptide **1e***

The peptide **1e** (0.01 mmol) and ynamide **2e** (0.02 mmol, 2 equiv) were dissolved in 10 mL PB (0.1 M, pH 8.0) and 0.5 mL CH_3CN at 37 °C. The reaction progress was monitored by HPLC (using Jupiter 5 μm C18 4.6 × 250 mm^2) with a gradient of 10-95% solvent B in 30 min at 220 nm and LC-MS. No product was detected within 5 h.

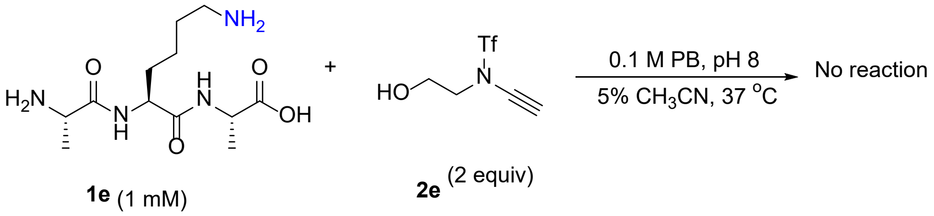

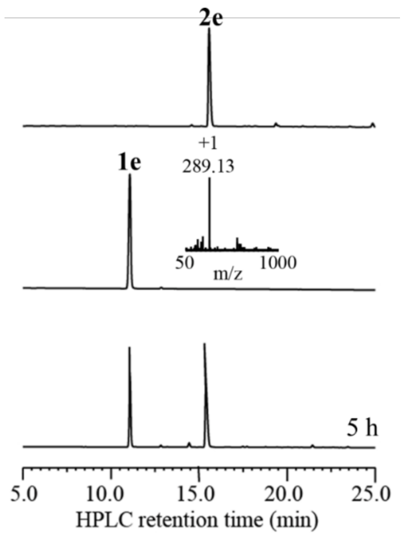

Figure S20. Chemoselectivity study of ynamide **2e** in the presence of peptide **1e**

5) *Chemoselectivity study of ynamide **2e** in the presence of peptide **1f***

The peptide **1f** (0.01 mmol) and ynamide **2e** (0.02 mmol, 2 equiv) were dissolved in 10 mL PB (0.1 M, pH 8.0) and 0.5 mL CH_3CN at 37 °C. The reaction progress was monitored by HPLC (using Jupiter 5 µm C18 4.6 × 250 mm^2) with a gradient of 10-95% solvent B in 30 min at 220 nm and LC-MS. No product was detected within 5 h.

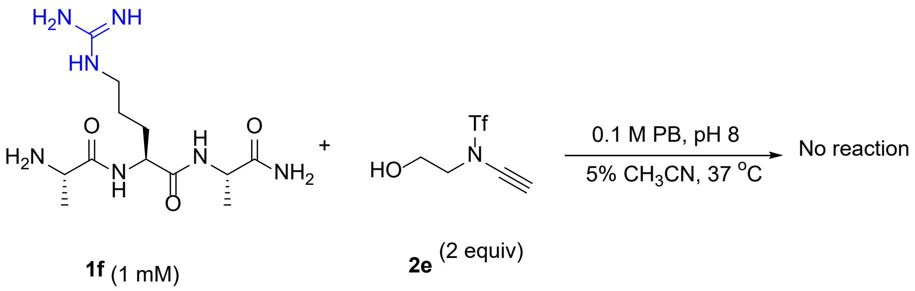

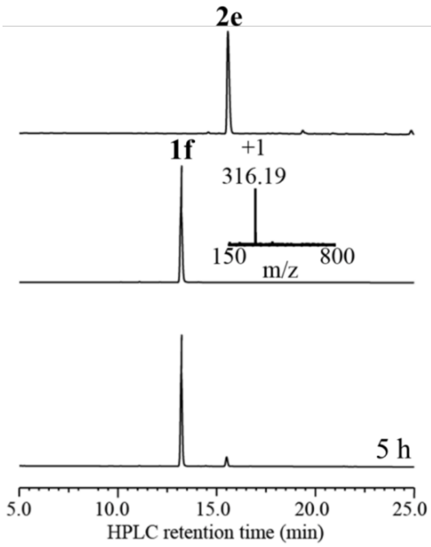

Figure S21. Chemoselectivity study of ynamide **2e** in the presence of peptide **1f**

6) *Chemoselectivity study of ynamide **2e** in the presence of peptide **1g***

The peptide **1g** (0.01 mmol) and ynamide **2e** (0.02 mmol, 2 equiv) were dissolved in 10 mL PB (0.1 M, pH 8.0) and 0.5 mL CH_3CN at 37 °C. The reaction progress was monitored by HPLC (using Jupiter 5 μm C18 4.6 × 250 mm^2) with a gradient of 10-95% solvent B in 30 min at 220 nm and LC-MS. No product was detected within 5 h.

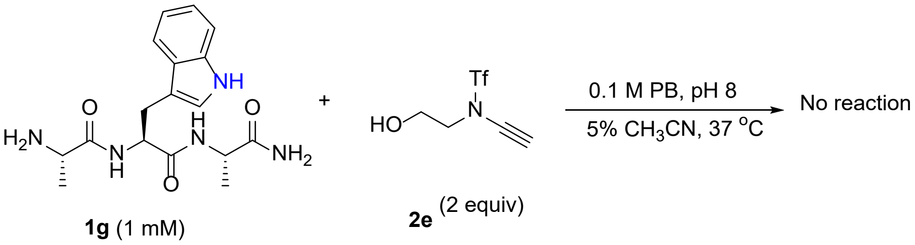

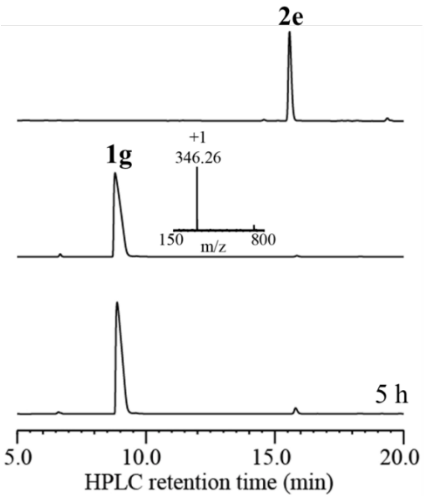

Figure S22. Chemoselectivity study of ynamide **2e** in the presence of peptide **1g**

7) *Chemoselectivity study of ynamide* ***2e*** *in the presence of peptide* ***1h***

The peptide **1h** (0.01 mmol) and ynamide **2e** (0.02 mmol, 2 equiv) were dissolved in 10 mL PB (0.1 M, pH 8.0) and 0.5 mL CH_3CN at 37 °C. The reaction progress was monitored by HPLC (using Jupiter 5 μm C18 4.6 × 250 mm^2) with a gradient of 10-95% solvent B in 30 min at 220 nm and LC-MS. No product was detected within 5 h.

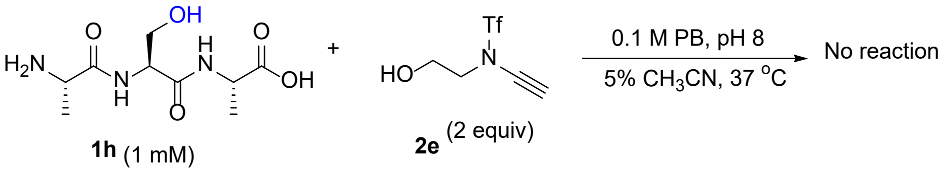

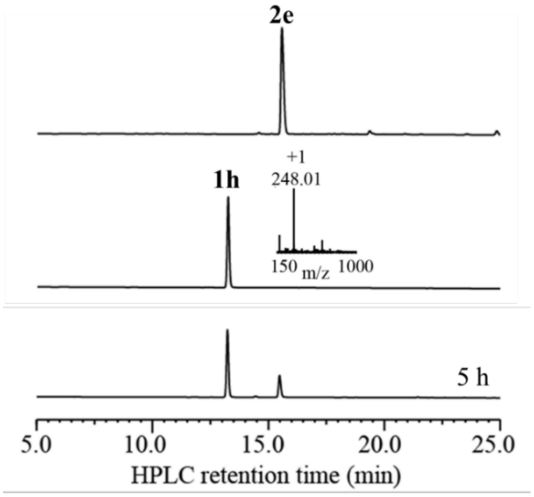

Figure S23. Chemoselectivity study of ynamide **2e** in the presence of peptide **1h**

8) *Chemoselectivity study of ynamide* ***2e*** *in the presence of peptide* ***1i***

The peptide **1i** (0.01 mmol) and ynamide **2e** (0.02 mmol, 2 equiv) were dissolved in 10 mL PB (0.1 M, pH 8.0) and 0.5 mL CH_3CN at 37 °C. The reaction progress was monitored by HPLC (using Jupiter 5 μm C18 4.6 × 250 mm^2) with a gradient of 10-95% solvent B in 30 min at 220 nm and LC-MS. No product was detected within 5 h.

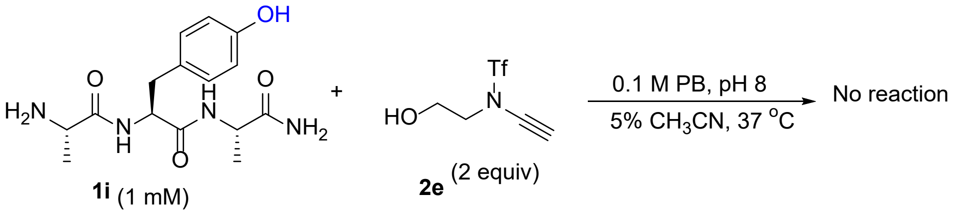

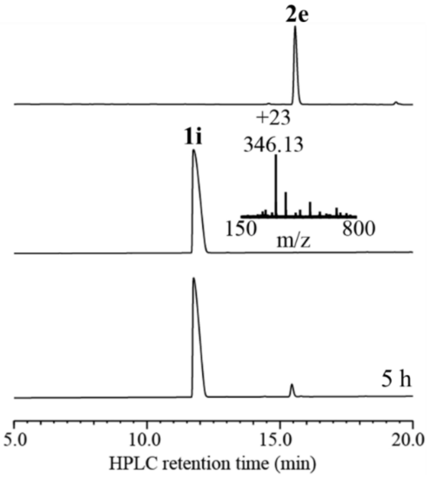

Figure S24. Chemoselectivity study of ynamide **2e** in the presence of peptide **1i**

9) *Chemoselectivity study of ynamide **2e** in the presence of peptide **1j***

The peptide **1j** (0.01 mmol) and ynamide **2e** (0.02 mmol, 2 equiv) were dissolved in 10 mL PB (0.1 M, pH 8.0) and 0.5 mL CH_3CN at 37 °C. The reaction progress was monitored by HPLC (using Jupiter 5 μm C18 4.6 × 250 mm^2) with a gradient of 10-95% solvent B in 30 min at 220 nm and LC-MS. After 20 min, the product **3je** was isolated in 63% yield. The product was characterized by ESI-MS ($[M + H]^+$ Found 1509.99, Calcd. 1509.60).

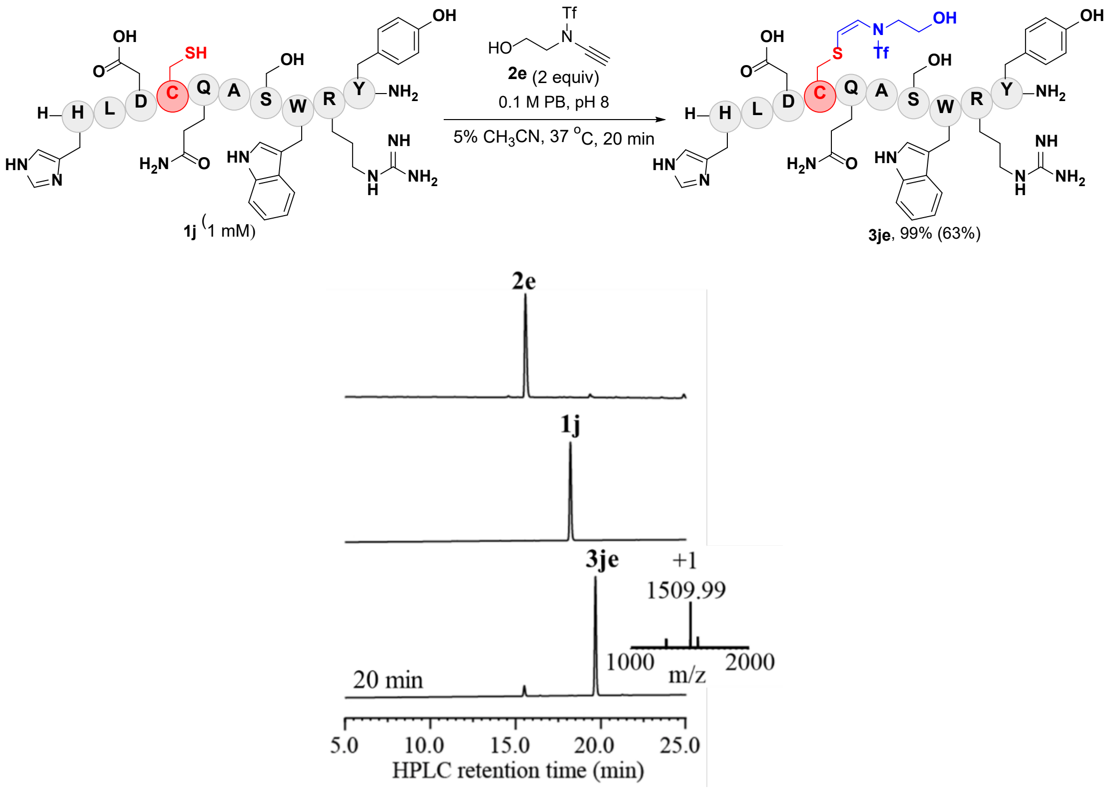

Figure S25. Chemoselectivity study of ynamide **2e** in the presence of peptide **1j**

10) *Chemoselectivity study of ynamide **2e** in the presence of peptide **1k***

The peptide **1k** (0.01 mmol) and ynamide **2e** (0.02 mmol, 2 equiv) were dissolved in 10 mL PB (0.1 M, pH 8.0) and 0.5 mL CH_3CN at 37 °C. The reaction progress was monitored by HPLC (using Jupiter 5 μm C18 4.6 × 250 mm^2) with a gradient of 10-95% solvent B in 30 min at 220 nm and LC-MS. No product was detected within 24 h.

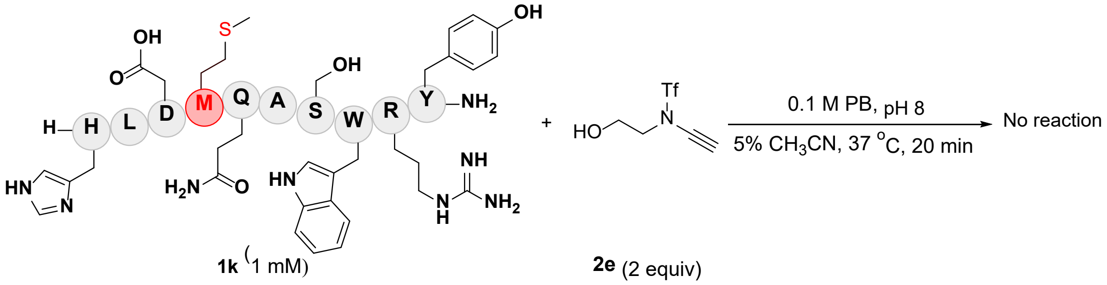

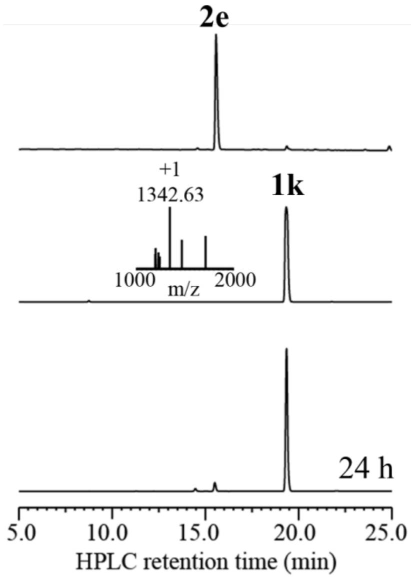

Figure S26. Chemoselectivity study of ynamide **2e** in the presence of peptide **1k**

11) *The modification of more complex peptide **1l** with ynamide **2e***

Peptide **1l** was synthesized as described in the general synthetic approach described previously. The crude peptide **1l** was purified by Prep RP-HPLC (C18 column) with ~ 30% isolated yield (mass Found 1937.5, Calcd. 1936.8). The results are shown in **Figure S75**.

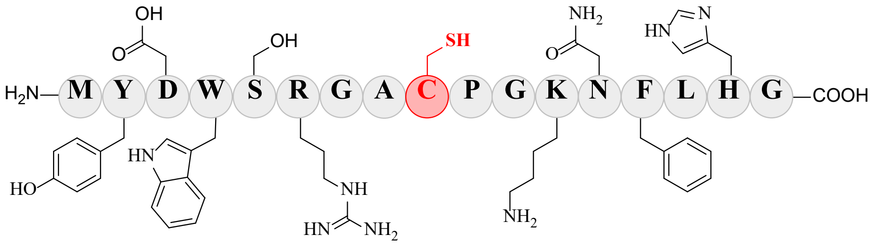

Figure S27. The sequence of peptide **1l**.

Peptide **1l** (3.8 mg, 1.96 µmol, 16.3 mM) was dissolved in 120 µL water as peptide **1l** stock solution. Ynamide **2e** (11.4 mg, 52.5 µmol, 105 mM) was dissolved in 500 µL MeCN as ynamide **2e** stock solution. 9.2 µL of peptide **1l** stock solution (0.15 µmol) and 7.1 µL of ynamide **2e** stock (5 equiv) were diluted in 133.7 µL PB (0.1 M, pH 8, final concentration of **1l** is 1mM), and the reaction mixture was incubated at 37 °C. Reaction progress was monitored by RP-HPLC (XSelect C18 column (3.5 µm, 130 Å, 4.6 × 150 mm) with a gradient of 5-70% B (0.1% TFA in MeCN) over 25 min at 220 nm (**Figure S28**) After 2 h, the reaction was completed to afford the corresponding product **3le** with 91% (based on HPLC integration) and 62% isolated yield. (mass Found 2154.5, Calcd. 2153.9) The results are shown in **Figure S77**.

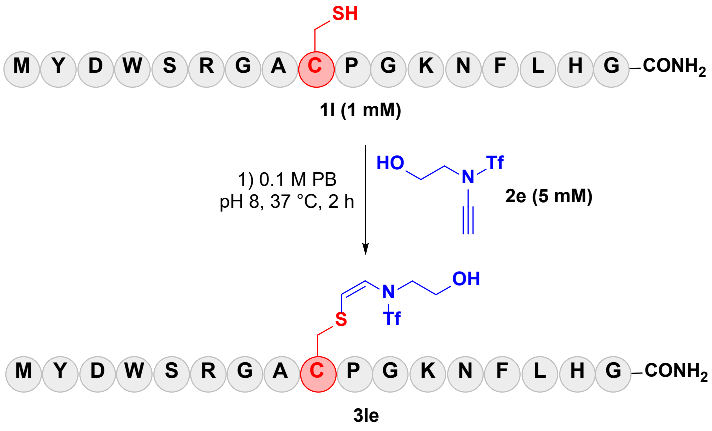

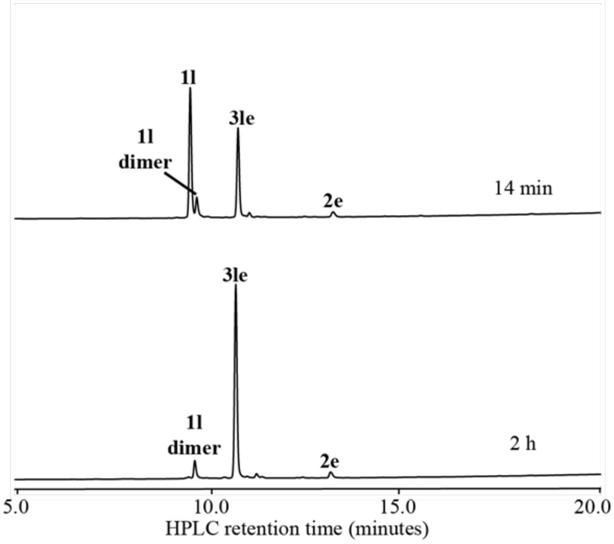

Figure S28. The modification reaction of model peptide **1l** with ynamide (**2e**)

Trypsin digestion of modified peptide 3le

The modified peptide **3le** (1.8 mg, 0.83 μmol) was dissolved in 1.8 mL of Tris buffer (50 mM Tris-HCl, pH 8, 8 M urea) and incubated at 37 °C bath for 1 h. Then, reaction was removed from the heating bath and allowed to reach room temperature. The concentration of urea was reduced to 0.9 M by addition 14.5 mL of 50 mM NH_4HCO_3. 60 μL of trypsin stock (1 mL/mg) was then added to the reaction solution (as 1: 30 ratio of trypsin : peptide), which was incubated at 37 °C for 15 min. Aliquots of the reaction were quenched by formic acid in water and checked by RP-HPLC (XSelect C18 column (3.5 μm, 130 Å, 4.6 × 150 mm) with a gradient of 5-70% B (0.1% TFA in MeCN) over 20 min at 220 nm).

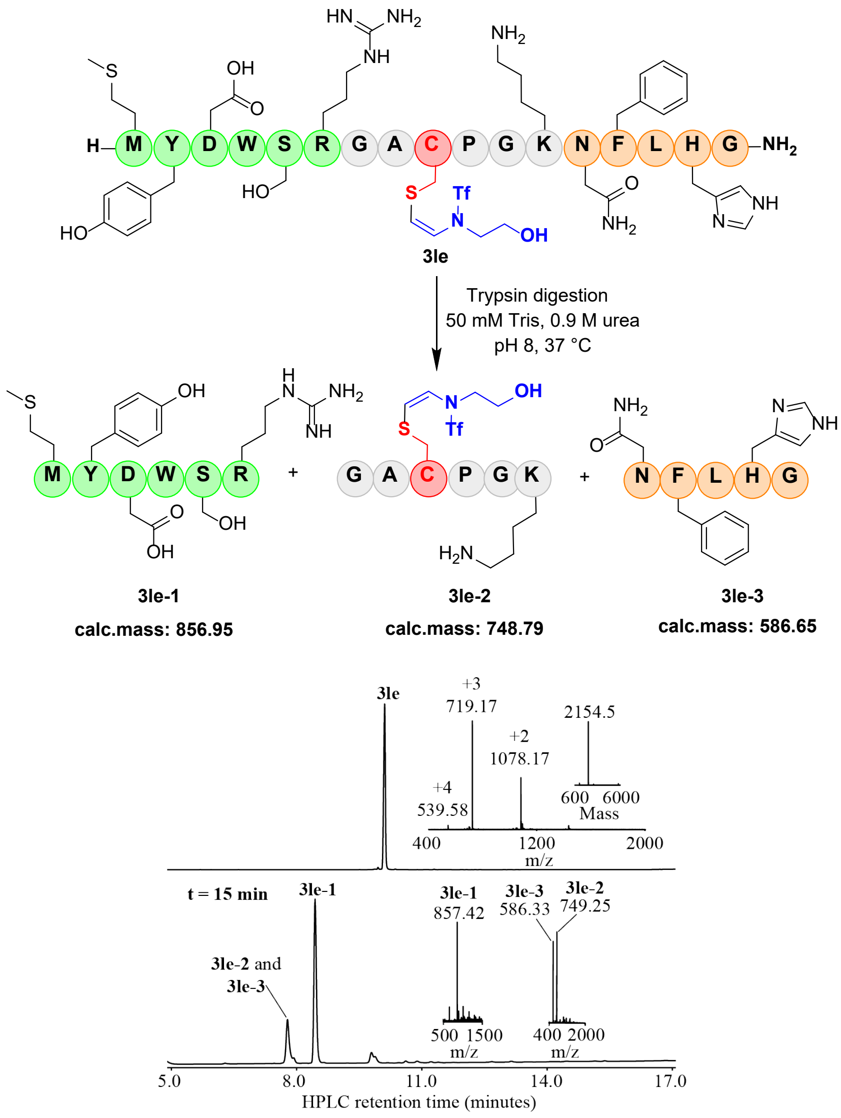

Figure S29. Trypsin digestion of the modified peptide **3le**

11) *The modification of peptide **1m** with ynamide **2e***

The peptide **1m** (0.01 mmol) and ynamide **2e** (0.02 mmol, 2 equiv) were dissolved in 10 mL PB (0.1 M, pH 8.0) and 0.5 mL CH_3CN at 37 °C. The reaction progress was monitored by HPLC (using Jupiter 5 μm C18 4.6 × 250 mm^2) with a gradient of 10-95% solvent B in 30 min at 220 nm and LC-MS. After 20 min, the product **3me** was isolated in 87% yield. The product was characterized by ^{1}H NMR (400 MHz, D_2O) δ 6.57 (d, *J* = 6.8 Hz, 1H), 6.05 (d, *J* = 6.7 Hz, 1H), 4.57 (dd, *J* = 8.6, 5.1 Hz, 1H), 3.99 (dd, *J* = 11.0, 4.4 Hz, 1H), 3.96 (s, 2H), 3.75 – 3.52 (m, 4H), 3.29 (dd, *J* = 14.5, 5.1 Hz, 1H), 3.08 (dd, *J* = 14.5, 8.7 Hz, 1H), 2.62 – 2.44 (m, 2H), 2.26 – 2.08 (m, 2H) ^{13}C NMR (101 MHz, D_2O) δ 174.3, 172.9, 172.0, 171.8, 133.4, 120.3, 119.8 (q, J = 323.0 Hz), 58.4, 53.7, 52.5, 52.3, 41.2, 34.7, 31.1, 25.6; HRMS (ESI-TOF) m/z: $C_{15}H_{24}F_3N_4O_9S_2^-$ [M - H]$^-$ Calcd. 523.0791; Found: 523.0786.

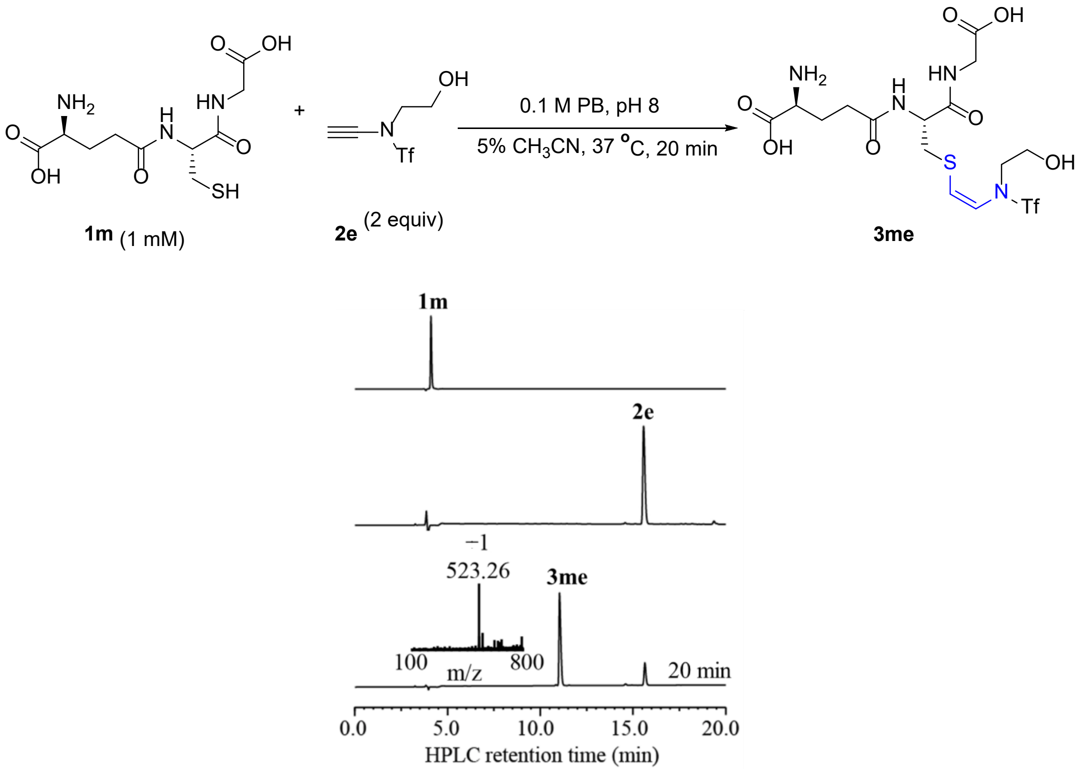

Figure S30. The modification of peptide **1m** with ynamide **2e**

12) *The modification of* ***reduced oxytocin (1n)*** *with ynamide* ***2e***

The peptide **1n** (0.01 mmol) and ynamide **2e** (0.02 mmol, 2 equiv) were dissolved in 10 mL PB (0.1 M, pH 8.0) and 0.5 mL CH_3CN at 37 °C. The reaction progress was monitored by HPLC (using Jupiter 5 μm C18 4.6 × 250 mm^2) with a gradient of 10-95% solvent B in 30 min at 220 nm and LC-MS. After 20 min, the product **3ne** was isolated in 65% yield. The product was characterized by ESI-MS ($[M + H]^+$ Found 1443.17, Calcd. 1443.46).

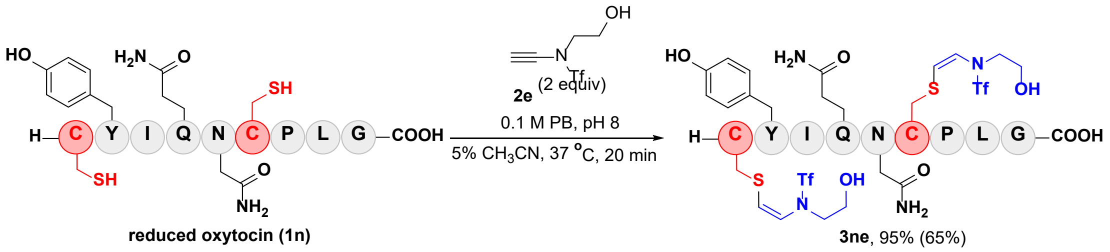

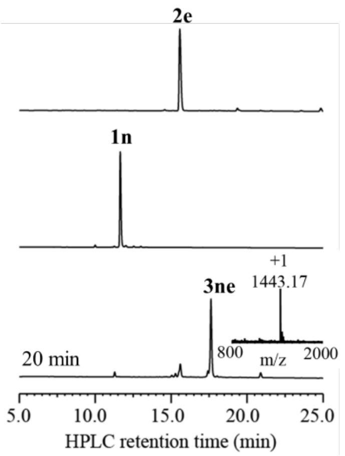

Figure S31. The modification of reduced oxytocin (**1n**) with ynamide **2e**

13) *The modification of cyclic peptide **1o** with ynamide **2e***

The peptide **1o** (0.01 mmol) and ynamide **2e** (0.02 mmol, 2 equiv) were dissolved in 10 mL PB (0.1 M, pH 8.0) and 0.5 mL CH_3CN at 37 °C. The reaction progress was monitored by HPLC (using Jupiter 5 μm C18 4.6 × 250 mm^2) with a gradient of 10-95% solvent B in 30 min at 220 nm and LC-MS. After 20 min, the product **3oe** was isolated in 76% yield. The product was characterized by ESI-MS ($[M + K]^+$ Found 834.17, Calcd. 834.19).

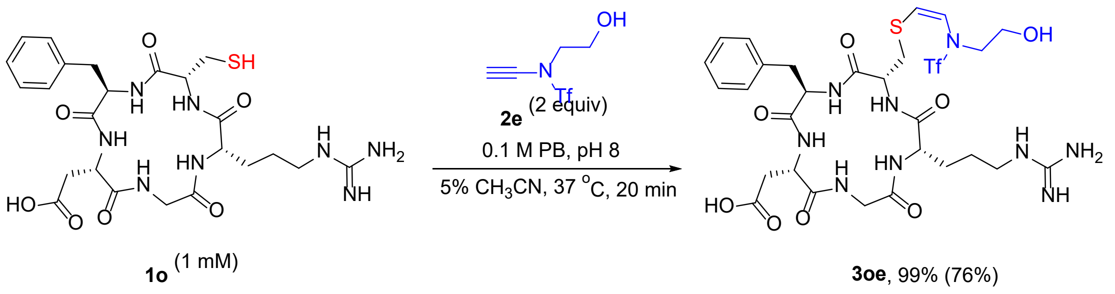

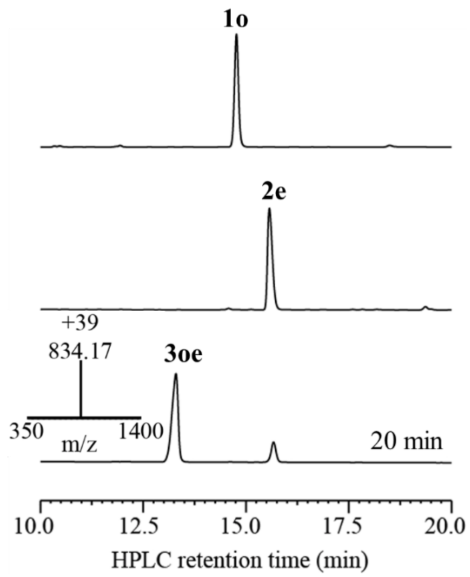

Figure S32. The modification of cyclic peptide **1o** with ynamide **2e**

14) *The modification of peptide **1p** with ynamide **2e***

The peptide **1p** (0.01 mmol) and ynamide **2e** (0.02 mmol, 2 equiv) were dissolved in 10 mL PB (0.1 M pH 8.0) and 0.5 mL CH_3CN at 37 °C. The reaction progress was monitored by HPLC (using Jupiter 5 μm C18 4.6 × 250 mm^2) with a gradient of 10-95% solvent B in 30 min at 220 nm and LC-MS. After 20 min, the product **3pe** was isolated in 76% yield. The product was characterized by ESI-MS ($[M + H]^+$ Found 851.44, Calcd. 851.41).

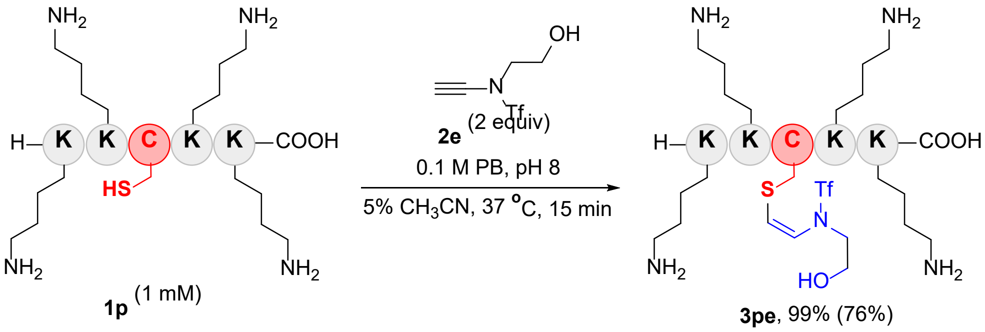

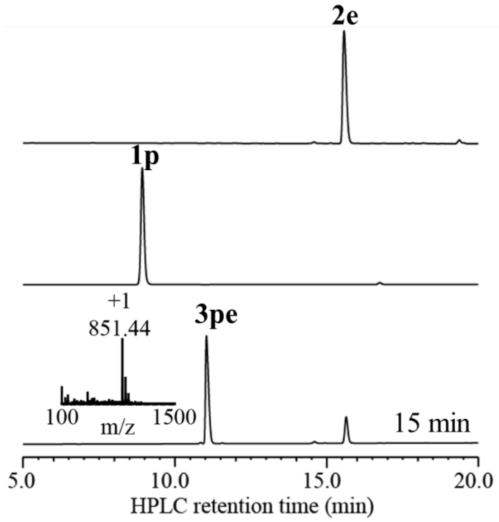

Figure S33. The modification of peptide **1p** with ynamide **2e**

15) *The modification of peptide **1q** with ynamide **2e***

The peptide **1q** (0.01 mmol) and ynamide **2e** (0.02 mmol, 2 equiv) were dissolved in 10 mL PB (0.1 M, pH 8.0) and 0.5 mL CH_3CN at 37 °C. The reaction progress was monitored by HPLC (using Jupiter 5 μm C18 4.6 × 250 mm^2) with a gradient of 10-95% solvent B in 30 min at 220 nm and LC-MS. After 20 min, the product **3qe** was isolated in 70% yield. The product was characterized by ESI-MS ($[M - H]^-$ Found 797.32, Calcd. 797.12).

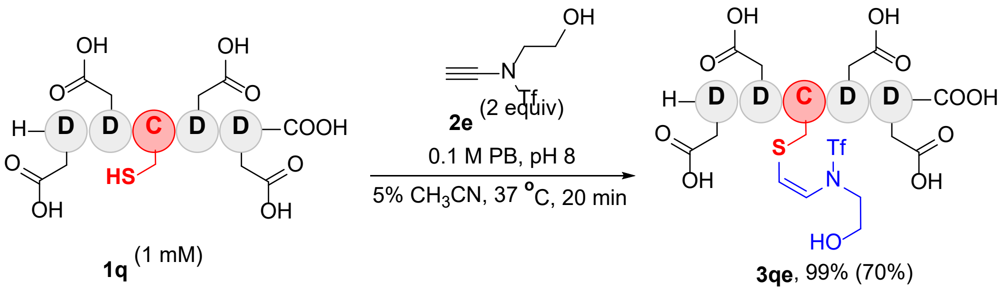

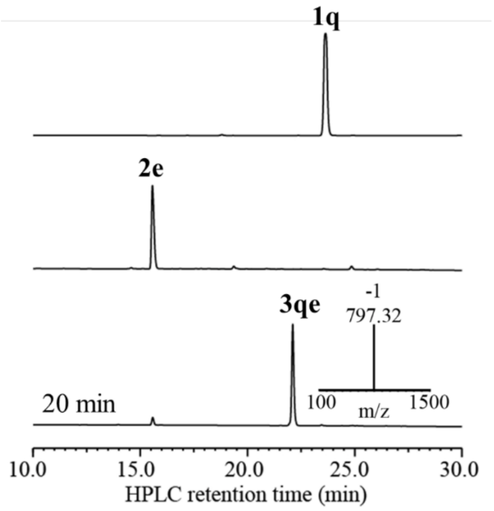

Figure S34. The modification of peptide **1q** with ynamide **2e**

16) *The modification of peptide **1a** with ynamide **2h***

The peptide **1a** (0.1 mmol) and ynamide **2h** (0.2 mmol, 2 equiv) were dissolved in 100 mL PB (0.1 M, pH 8.0) and 100 mL CH_3CN at 37 °C. The reaction progress was monitored by HPLC (using Jupiter 5 μm C18 4.6 × 250 mm^2) with a gradient of 10-95% solvent B in 30 min at 220 nm and LC-MS. After 20 min, the product **4ah** was isolated in 92% yield. The product was characterized by ^{1}H NMR (400 MHz, $CDCl_3$) δ 9.18 (t, J = 5.7 Hz, 1H), 8.99 (s, 1H), 7.80 (d, J = 7.8 Hz, 1H), 7.69 (t, J = 7.8 Hz, 1H), 7.52 – 7.33 (m, 2H), 6.28 (d, J = 6.9 Hz, 1H), 5.98 (d, J = 6.9 Hz, 1H), 5.63 (d, J = 5.8 Hz, 1H), 4.90 (s, 1H), 4.49 (s, 1H), 4.21 (m, 2H), 3.82 (d, J = 5.0 Hz, 2H), 3.71 (s, 2H), 3.53 – 3.27 (m, 2H), 1.45 (d, J = 11.2 Hz, 3H), 1.40 (s, 9H), 1.29 (s, 3H) ^{13}C NMR (101 MHz, $CDCl_3$) δ 173.4, 169.5, 162.4, 161.4, 155.3, 154.5, 149.1, 134.3, 132.4, 130.1, 125.3, 119.9 (q, J = 323.0 Hz), 119.7, 118.7, 117.9, 116.7, 79.7, 62.1, 53.1, 50.1, 48.5, 38.5, 36.6, 28.3, 18.8, 14.1; HRMS (ESI-TOF) m/z: [M + H]$^+$ Calcd. $C_{28}H_{36}F_3N_4O_{10}S_2^+$ 709.1819; Found: 709.1823.

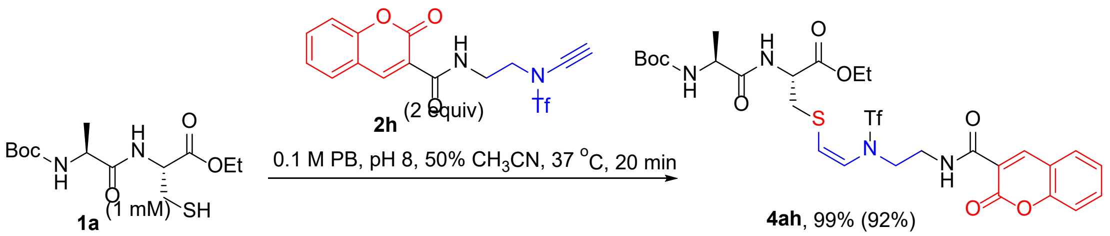

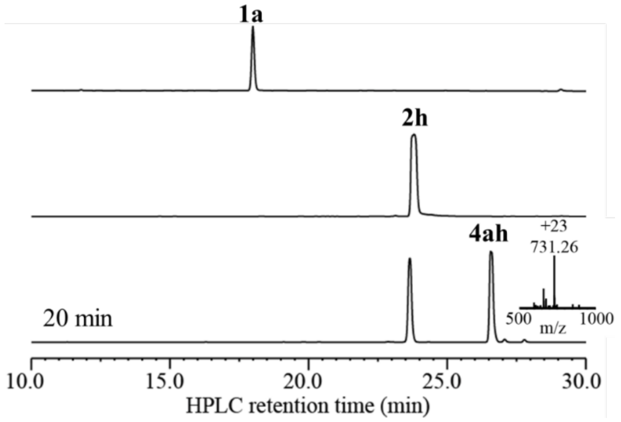

Figure S35. The modification of peptide **1a** with ynamide **2h**

17) *The modification of peptide **1a** with ynamide **2i***

The peptide **1a** (0.1 mmol) and ynamide **2i** (0.2 mmol, 2 equiv) were dissolved in 100 mL PB (0.1 M, pH 8.0) and 100 mL CH_3CN at 37 °C. The reaction progress was monitored by HPLC (using Jupiter 5 μm C18 4.6 × 250 mm^2) with a gradient 30-95% solvent B in 30 min at 220 nm and LC-MS. After 20 min, the product **4ai** was isolated in 95% yield. The product was characterized by ^{1}H NMR (400 MHz, $CDCl_3$) δ 7.68 (d, J = 8.1 Hz, 2H), 7.47 (d, J = 8.1 Hz, 2H), 7.11 (s, 1H), 6.99 (s, 1H), 6.90 (d, J = 8.9 Hz, 1H), 6.67 (d, J = 9.0 Hz, 1H), 6.18 (d, J = 6.2 Hz, 1H), 5.87 (d, J = 6.7 Hz, 1H), 5.22 (s, 1H), 4.76 (d, J = 4.6 Hz, 1H), 4.21 (d, J = 6.8 Hz, 5H), 3.83 (s, 5H), 3.75 (s, 2H), 3.34 – 3.07 (m, 2H), 2.38 (s, 3H), 1.43 (s, 9H), 1.33 (d, J = 7.1 Hz, 3H), 1.27 (d, J = 7.4 Hz, 3H) ^{13}C NMR (101 MHz, $CDCl_3$) δ 173.0, 169.5, 168.3, 156.1, 139.3, 136.2, 133.9, 132.0, 131.2, 130.9, 130.7, 129.1, 119.9 (q, J = 323.0 Hz),119.5, 114.9, 112.0, 111.6, 101.5, 80.1, 62.2, 61.1, 55.7, 52.9, 50.2, 48.1, 36.1, 30.1, 28.3, 18.1, 14.1, 13.3; HRMS (ESI-TOF) m/z: $C_{37}H_{45}ClF_3N_4O_{11}S_2^+$ [M + H]$^+$ Calcd. 877.2161; Found: 877.2164.

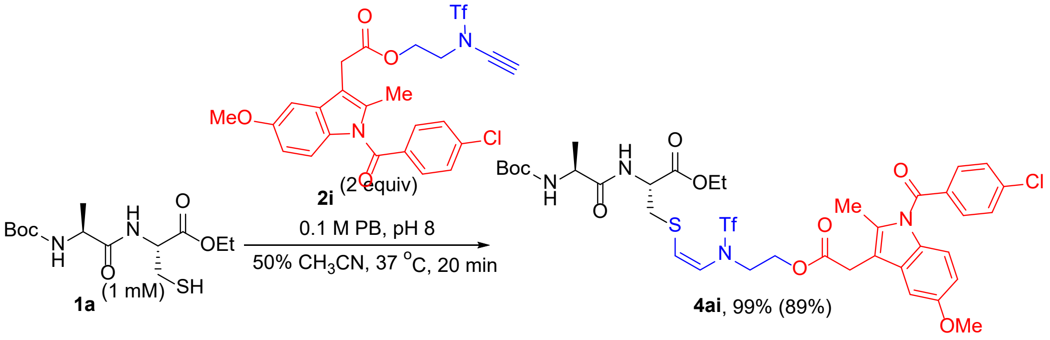

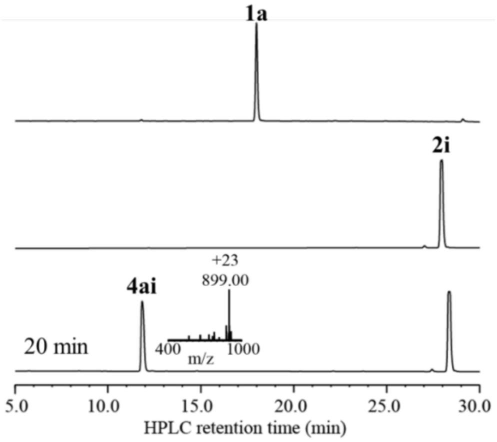

Figure S36. The modification of peptide **1a** with ynamide **2i**

18) *The modification of peptide **1a** with ynamide **2j***

The peptide **1a** (0.1 mmol) and ynamide **2j** (0.2 mmol, 2 equiv) were dissolved in 100 mL PB (0.1 M, pH 8.0) and 5 mL CH_3CN at 37 °C. The reaction progress was monitored by HPLC (using Jupiter 5 μm C18 4.6 × 250 mm^2) with a gradient of 10-95% solvent B in 30 min at 220 nm and LC-MS. After 20 min, the product **4aj** was isolated in 85% yield. The product was characterized by ^1H NMR (400 MHz, $CDCl_3$) δ 7.55 (s, 1H), 6.33 (d, *J* = 6.2 Hz, 2H), 6.12 – 5.86 (m, 2H), 5.57 (s, 1H), 4.79 (d, *J* = 5.4 Hz, 1H), 4.51 (s, 1H), 4.32 (s, 1H), 4.23 (d, *J* = 6.4 Hz, 4H), 3.85 (s, 2H), 3.24 (m, 3H), 2.91 (dd, *J* = 12.4, 4.0 Hz, 1H), 2.75 (d, *J* = 12.7 Hz, 1H), 2.38 (t, *J* = 7.0 Hz, 2H), 1.68 (d, *J* = 6.5 Hz, 4H), 1.46 (d, *J* = 18.0 Hz, 9H), 1.36 (d, *J* = 6.7 Hz, 3H), 1.30 (t, *J* = 6.9 Hz, 3H) ^{13}C NMR (101 MHz, $CDCl_3$) δ 173.5, 173.4, 169.7, 164.1, 155.6, 132.0, 119.7 (q, J = 323.0 Hz), 119.7, 79.9, 62.2, 62.1, 60.7, 60.1, 55.6, 53.0, 50.1, 48.2, 40.5, 36.1, 33.8, 29.6, 28.4, 28.3, 28.2, 24.6, 18.2, 14.1; HRMS (ESI-TOF) m/z: $C_{27}H_{46}F_3N_6O_{10}S_3^+$ $[M + H]^+$ Calcd. 764.2275; Found: 764.2296.

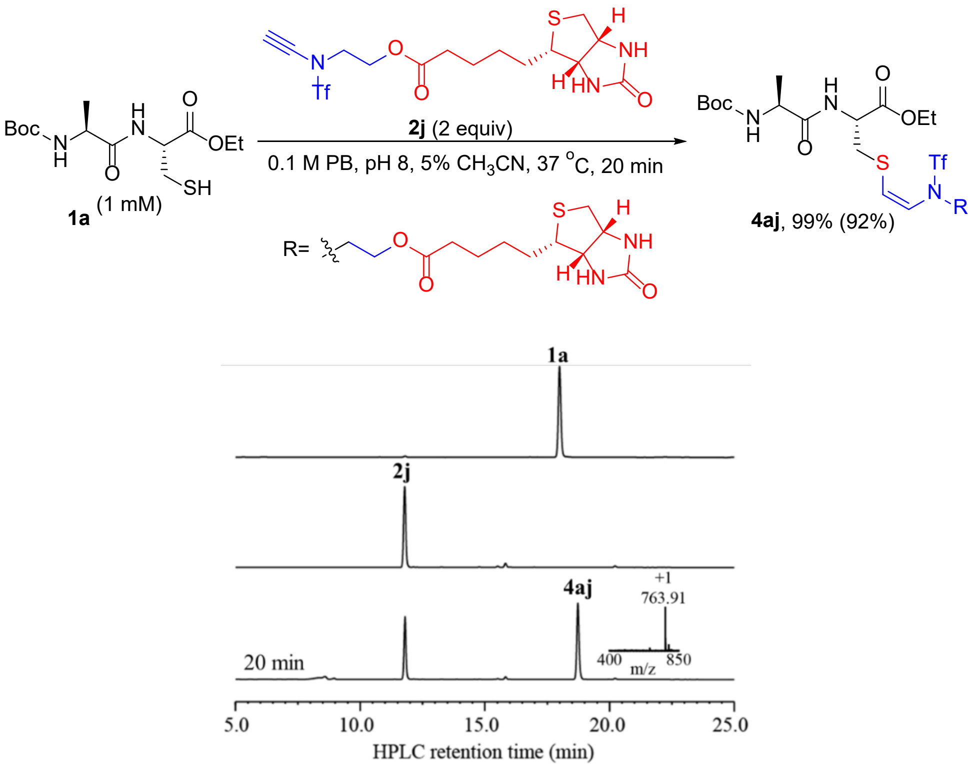

Figure S37. The modification of peptide **1a** with ynamide **2j**

19) *The modification of peptide **1a** with ynamide **2k***

The peptide **1a** (0.1 mmol) and ynamide **2k** (0.2 mmol, 2 equiv) were dissolved in 100 mL PB (0.1 M, pH 8.0) and 10 mL DMSO at 37 °C. The reaction progress was monitored by HPLC (using Jupiter 5 μm C18 4.6 × 250 mm^2) with a gradient of 10-95% solvent B in 30 min at 220 nm and LC-MS. After 20 min, the product **4ak** was isolated in 92% yield. The product was characterized by ^{1}H NMR (400 MHz, $CDCl_3$) δ 7.07 (d, *J* = 6.0 Hz, 1H), 6.37 (d, *J* = 6.6 Hz, 1H), 6.11 (d, *J* = 6.6 Hz, 1H), 5.12 (d, *J* = 7.0 Hz, 1H), 4.95 – 4.70 (m, 1H), 4.42 (q, *J* = 18.0 Hz, 2H), 4.23 (m, 3H), 3.30 (m, 2H), 2.46 (s, 1H), 1.45 (s, 9H), 1.37 (d, *J* = 6.9 Hz, 3H), 1.30 (t, *J* = 7.1 Hz, 3H) ^{13}C NMR (101 MHz, $CDCl_3$) δ 172.8, 169.5, 155.4, 133.0, 120.0, 119.7 (q, J = 323.0 Hz), 80.2, 76.1, 74.6, 62.3, 53.0, 50.3, 39.4, 36.2, 28.3, 18.1, 14.0; HRMS (ESI-TOF) m/z: $C_{19}H_{29}F_3N_3O_7S_2^+$ $[M + Na]^+$ Calcd. 554.1213; Found: 554.1215.

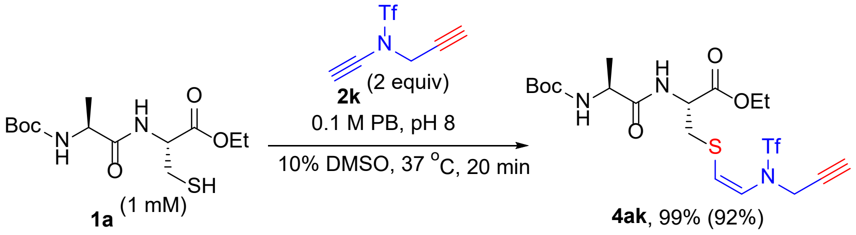

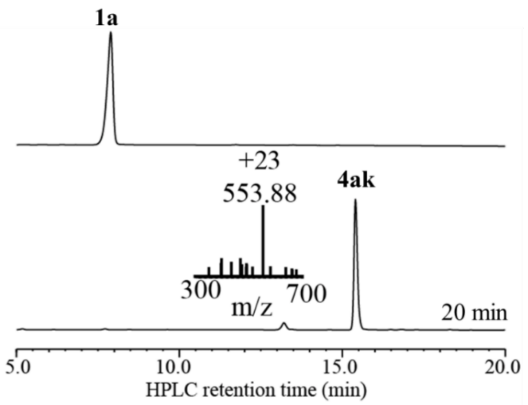

Figure S38. The modification of peptide **1a** with ynamide **2k**

20) *The modification of cyclic peptide **1o** with ynamide **2h***

The peptide **1o** (0.1 mmol) and ynamide **2h** (0.2 mmol, 2 equiv) were dissolved in 200 mL PB (0.1 M, pH 8.0) and 200 mL CH_3CN at 37 °C. The reaction progress was monitored by HPLC (using Jupiter 5 μm C18 4.6 × 250 mm^2) with a gradient of 10-95% solvent B in 30 min at 220 nm and LC-MS. After 10 min, the product **4oh** was isolated in 90% yield. The product was characterized by ^{1}H NMR (400 MHz, DMSO) δ 8.98 (t, *J* = 5.8 Hz, 1H), 8.92 (s, 1H), 8.37 – 8.24 (m, 1H), 8.17 (t, *J* = 7.9 Hz, 2H), 8.06 – 7.96 (m, 2H), 7.78 (t, *J* = 7.8 Hz, 1H), 7.59 (s, 1H), 7.56 – 7.42 (m, 2H), 7.24 (m, 6H), 6.76 (d, *J* = 6.8 Hz, 1H), 6.13 (d, *J* = 6.7 Hz, 1H), 4.67 (d, *J* = 6.0 Hz, 1H), 4.53 (d, *J* = 7.0 Hz, 1H), 4.26 (d, *J* = 4.9 Hz, 1H), 4.21 – 4.07 (m, 1H), 3.78 (t, *J* = 5.3 Hz, 2H), 3.55 (dd, *J* = 11.1, 5.5 Hz, 2H), 3.28 (dd, *J* = 15.0, 3.1 Hz, 1H), 3.02 (dd, *J* = 13.5, 7.6 Hz, 1H), 2.82 (dd, *J* = 14.7, 7.3 Hz, 2H), 2.78 – 2.65 (m, 2H), 2.39 (dd, *J* = 16.1, 5.5 Hz, 1H), 2.22 (t, *J* = 8.4 Hz, 1H), 1.93 – 1.68 (m, 1H), 1.46 (dd, *J* = 28.1, 21.2 Hz, 3H), 1.29 – 1.12 (m, 3H) ^{13}C NMR (101 MHz, DMSO) δ 173.1, 172.4, 171.8, 171.0, 163.3, 157.6, 139.0, 129.3, 128.4, 126.4, 119.8 (q, J = 320.0 Hz), 119.2, 65.4, 61.5, 60.5, 59.7, 56.5, 55.8, 53.3, 48.4, 43.3, 36.7, 33.7, 28.4, 24.7, 19.0, 15.6; HRMS (ESI-TOF) m/z: $C_{39}H_{48}F_3N_{10}O_{12}S_2^-$ [M - H]$^-$ Calcd. 967.2696; Found: 967.2644.

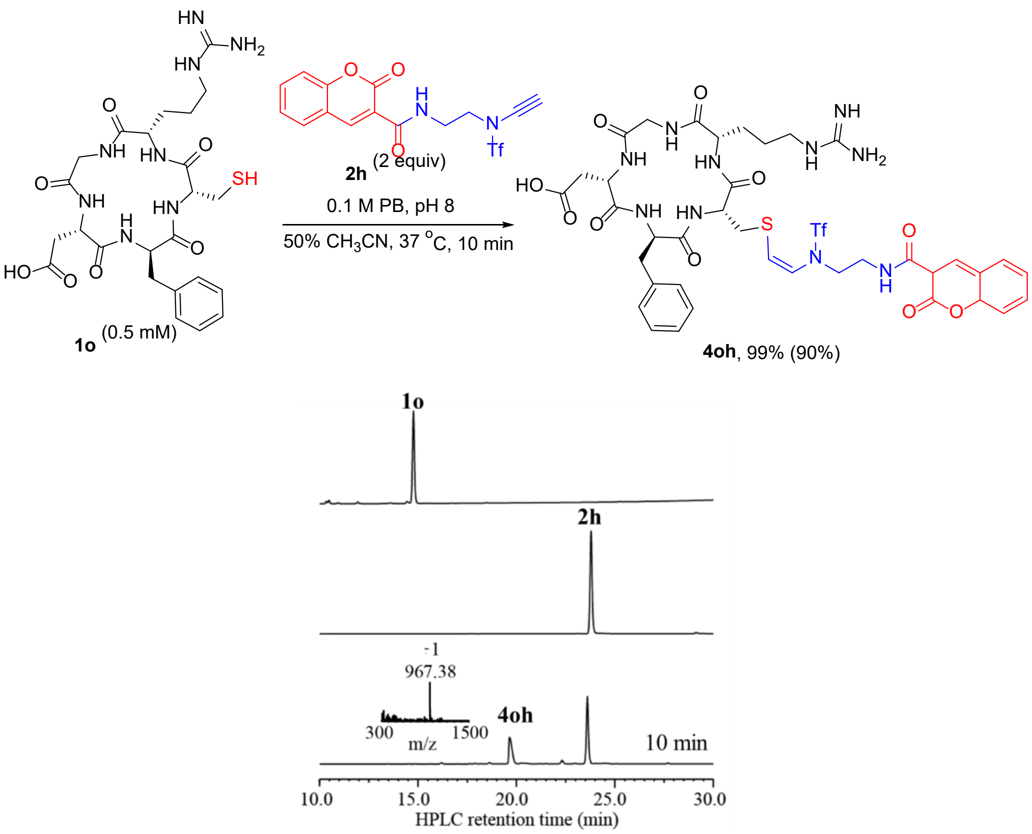

Figure S39. The modification of cyclic peptide **1o** with ynamide **2h**

21) *The modification of cyclic peptide **1o** with ynamide **2i***

The peptide **1o** (0.1 mmol) and ynamide **2i** (0.2 mmol, 2 equiv) were dissolved in 200 mL PB (0.1 M, pH 8.0) and 200 mL CH_3CN at 37 °C. The reaction progress was monitored by HPLC (using Jupiter 5 μm C18 4.6 × 250 mm^2) with a gradient of 10-95% solvent B in 30 min at 220 nm and LC-MS. After 10 min, the product **4oi** was isolated in 89% yield. The product was characterized by ^{1}H NMR (400 MHz, DMSO) δ 8.43 – 8.19 (m, 3H), 8.10 (d, *J* = 8.1 Hz, 1H), 7.83 (d, *J* = 8.0 Hz, 1H), 7.72 – 7.61 (m, 4H), 7.50 (d, *J* = 26.3 Hz, 1H), 7.29 – 7.08 (m, 6H), 7.02 (d, *J* = 2.1 Hz, 1H), 6.92 (d, *J* = 9.0 Hz, 1H), 6.80 – 6.59 (m, 2H), 6.04 (d, *J* = 6.8 Hz, 1H), 4.62 (d, *J* = 7.9 Hz, 1H), 4.52 (dd, *J* = 14.2, 7.0 Hz, 1H), 4.14 (m, 3H), 3.41 – 3.33 (m, 1H), 3.31 – 3.19 (m, 1H), 3.17 – 3.05 (m, 3H), 2.76 (dd, *J* = 13.9, 7.1 Hz, 1H), 2.66 (dd, *J* = 15.9, 8.4 Hz, 1H), 2.34 (dd, *J* = 16.1, 5.4 Hz, 1H), 2.23 (s, 3H), 1.83 (d, *J* = 19.3 Hz, 1H), 1.72 (d, *J* = 12.0 Hz, 2H), 1.68 – 1.59 (m, 2H), 1.54 (d, *J* = 8.7 Hz, 1H), 1.49 – 1.37 (m, 3H), 1.27 – 1.19 (m, 1H), 1.12 (m, 3H) ^{13}C NMR (101 MHz, DMSO) δ 172.1, 171.2, 171.1, 170.8, 170.5, 170.3, 169.7, 168.3, 157.2, 156.1, 138.2, 138.1, 136.1, 134.5, 133.2, 131.7, 131.0, 130.7, 129.5, 129.5, 128.5, 126.6, 119.8 (q, J = 320.0 Hz), 119.5, 117.7, 112.8, 112.0, 102.0, 78.3, 68.7, 62.3, 61.2, 55.8, 54.2, 52.9, 49.4, 48.4, 35.8, 30.7, 25.8, 24.3, 13.6; HRMS (ESI-TOF) m/z: $C_{48}H_{55}ClF_3N_{10}O_{13}S_2^+$ $[M + H]^+$ Calcd. 1135.3027; Found: 1135.3031.

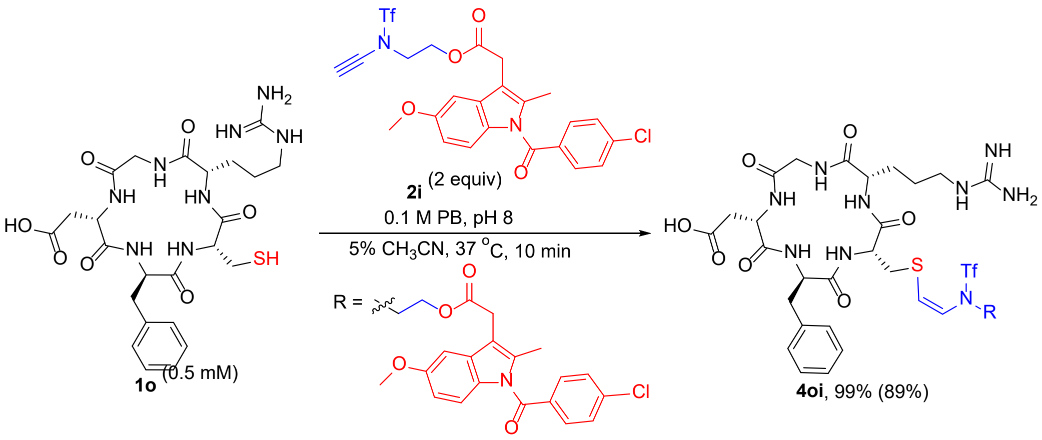

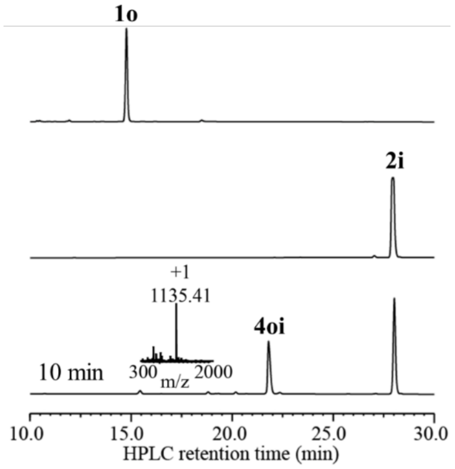

Figure S40. The modification of cyclic peptide **1o** with ynamide **2i**

22) *The modification of cyclic peptide **1o** with ynamide **2j***

The peptide **1o** (0.1 mmol) and ynamide **2j** (0.2 mmol, 2 equiv) were dissolved in 200 mL PB (0.1 M, pH 8.0) and 10 mL CH_3CN at 37 °C. The reaction progress was monitored by HPLC (using Jupiter 5 μm C18 4.6 × 250 mm^2) with a gradient of 10-95% solvent B in 30 min at 220 nm and LC-MS. After 10 min, the product **4oj** was isolated in 92% yield. The product was characterized by ^{1}H NMR (400 MHz, DMSO) δ 8.37 (d, J = 119.4 Hz, 2H), 7.77 (s, 2H), 7.19 (dd, J = 15.5, 6.5 Hz, 6H), 6.73 (d, J = 5.8 Hz, 1H), 6.45 (d, J = 32.0 Hz, 2H), 6.02 (d, J = 5.6 Hz, 1H), 4.57 (d, J = 68.1 Hz, 2H), 4.31 (s, 1H), 4.13 (t, J = 37.2 Hz, 4H), 3.76 (s, 1H), 3.41 (m, 6H), 3.21 (s, 2H), 3.09 (s, 3H), 2.81 (s, 1H), 2.63 – 2.53 (m, 2H), 2.30 (s, 2H), 2.05 (d, J = 32.0 Hz, 1H), 1.73 (d, J = 36.2 Hz, 1H), 1.68 – 1.20 (m, 8H), 1.12 – 0.98 (m, 4H) ^{13}C NMR (101 MHz, DMSO) δ 173.1, 172.4, 171.8, 171.0, 163.3, 157.6, 139.0, 129.3, 128.4, 126.4, 119.9 (q, J = 320.0 Hz), 119.2, 116.0, 65.4, 61.5, 60.5, 59.7, 56.5, 55.8, 53.3, 48.4, 43.3, 36.7, 33.7, 28.4, 24.7, 19.0, 15.6; HRMS (ESI-TOF) m/z: $C_{39}H_{55}F_3N_{11}O_{12}S_3^+$ $[M + H]^+$ Calcd. 1022.3140; Found: 1022.3117.

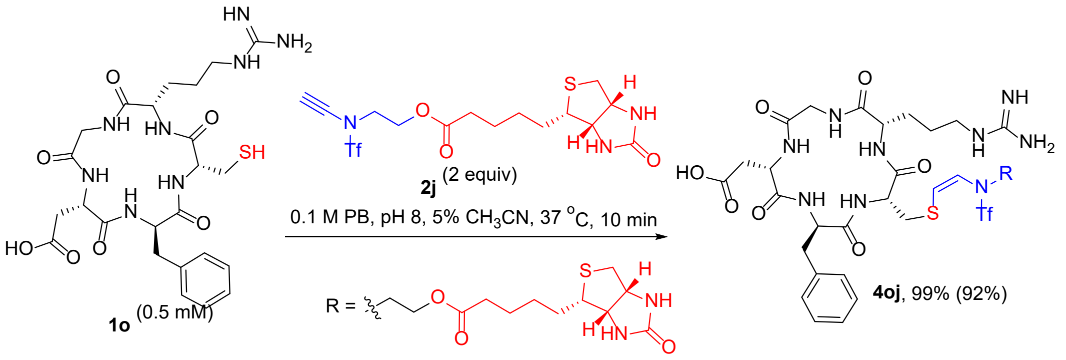

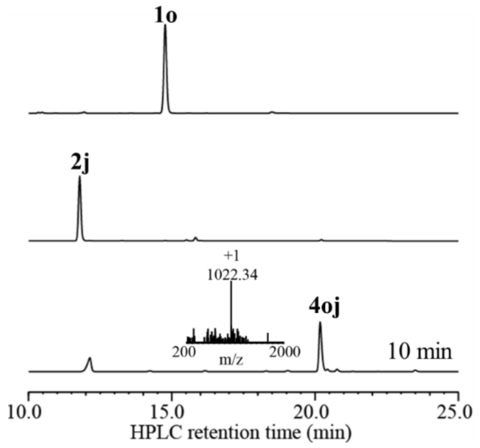

Figure S41. The modification of cyclic peptide **1o** with ynamide **2j**

23) *The modification of cyclic peptide* ***1o*** *with ynamide* ***2k***

The peptide **1o** (0.1 mmol) and ynamide **2k** (0.2 mmol, 2 equiv) were dissolved in 200 mL PB (0.1 M, pH 8.0) and 20 mL DMSO at 37 °C. The reaction progress was monitored by HPLC (using Jupiter 5 μm C18 4.6 × 250 mm^2) with a gradient of 10-95% solvent B in 30 min at 220 nm and LC-MS. After 10 min, the product **4ok** was isolated in 92% yield. The product was characterized by ^{1}H NMR (400 MHz, DMSO) δ 8.31 – 8.19 (m, 1H), 8.19 – 8.06 (m, 1H), 7.96 (dd, J = 8.0, 4.4 Hz, 1H), 7.57 (s, 1H), 7.35 – 7.12 (m, 5H), 6.79 (d, J = 6.7 Hz, 1H), 6.14 (d, J = 6.6 Hz, 1H), 4.64 (dd, J = 14.1, 8.1 Hz, 1H), 4.58 – 4.47 (m, 1H), 4.42 (d, J = 1.9 Hz, 2H), 4.32 – 3.95 (m, 2H), 3.52 (t, J = 2.1 Hz, 1H), 3.36 – 3.20 (m, 1H), 3.09 (s, 2H), 3.02 (d, J = 7.6 Hz, 1H), 2.82 (dd, J = 14.7, 7.3 Hz, 3H), 2.76 – 2.62 (m, 2H), 2.43 – 2.29 (m, 1H), 2.19 (t, J = 8.4 Hz, 1H), 1.78 (d, J = 6.9 Hz, 1H), 1.56 – 1.33 (m, 2H), 1.23 (d, J = 7.3 Hz, 3H) ^{13}C NMR (101 MHz, DMSO) δ 172.1, 171.4, 171.3, 170.6, 170.5, 169.8, 157.1, 137.9, 134.5, 129.7, 129.6, 128.6, 126.72, 119.8 (q, J = 320.0 Hz), 119.0, 77.4, 77.1, 57.6, 54.7, 52.7, 49.4, 43.7, 37.4, 35.8, 28.4, 27.56, 26.3, 25.1, 16.1; HRMS (ESI-TOF) m/z: $C_{30}H_{39}F_3N_9O_9S_2^+$ [M + H]$^+$ Calcd. 790.2259; Found: 790.2278.

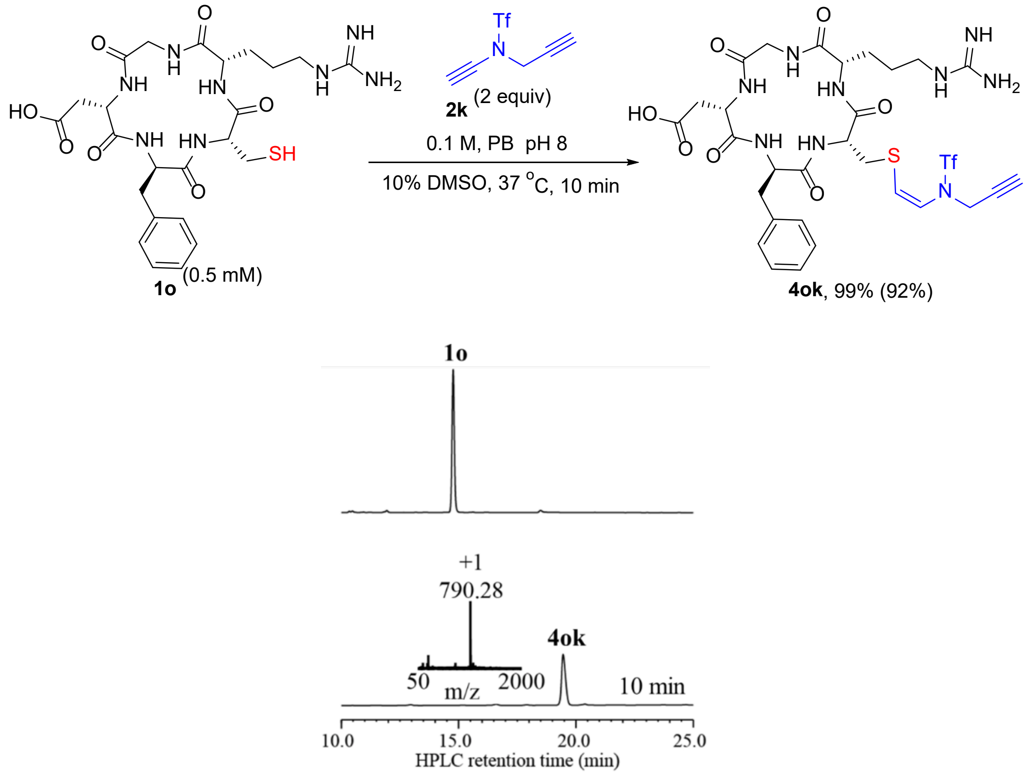

Figure S42. The modification of cyclic peptide **1o** with ynamide **2k**

24) *The modification of peptide **1r** with ynamide **2h***

The peptide **1r** (0.01 mmol) and ynamide **2h** (0.02 mmol, 2 equiv) were dissolved in 10 mL PB (0.1 M, pH 8.0) and 10 mL CH_3CN at 37 °C. The reaction progress was monitored by HPLC (using Jupiter 5 μm C18 4.6 × 250 mm^2) with a gradient of 10-95% solvent B in 30 min at 220 nm and LC-MS. After 20 min, the product **4rh** was isolated in 76% yield. The product was characterized by ESI-MS ($[M + H]^+$ Found 971.35, Calcd. 971.29).

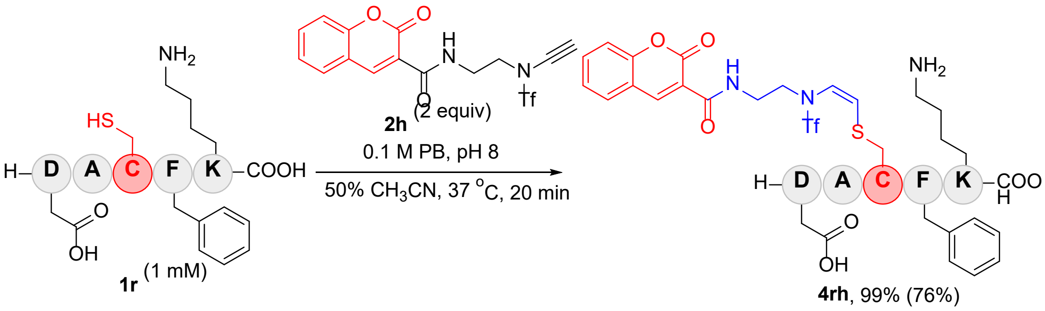

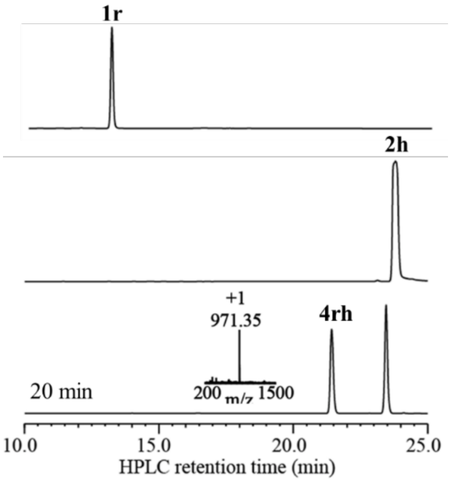

Figure S43. The modification of peptide **1r** with ynamide **2h**

25) *The modification of peptide **1r** with ynamide **2i***

The peptide **1r** (0.01 mmol) and ynamide **2i** (0.02 mmol, 2 equiv) were dissolved in 10 mL PB (0.1 M, pH 8.0) and 10 mL CH_3CN at 37 °C. The reaction progress was monitored by HPLC (using Jupiter 5 μm C18 4.6 × 250 mm^2) with a gradient 10-100% solvent B in 30 min at 220 nm and LC-MS. After 20 min, the product **4ri** was isolated in 72% yield. The product was characterized by ESI-MS ($[M + H]^+$ Found 1139.21, Calcd. 1139.32).

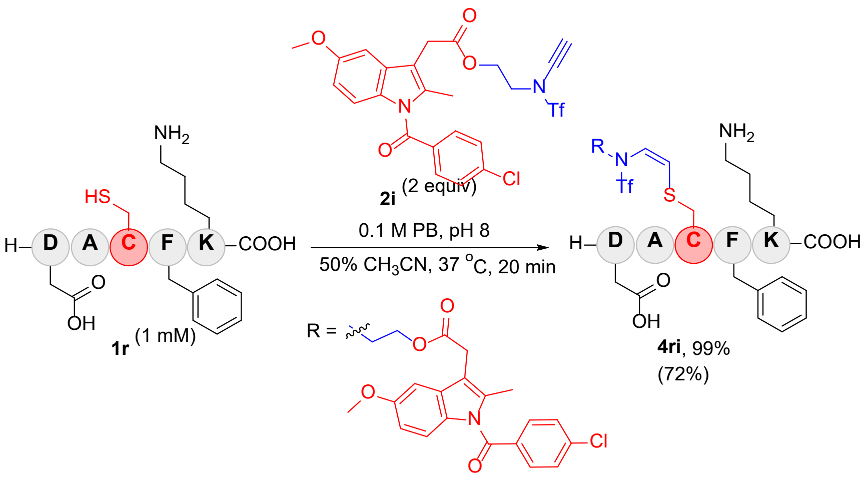

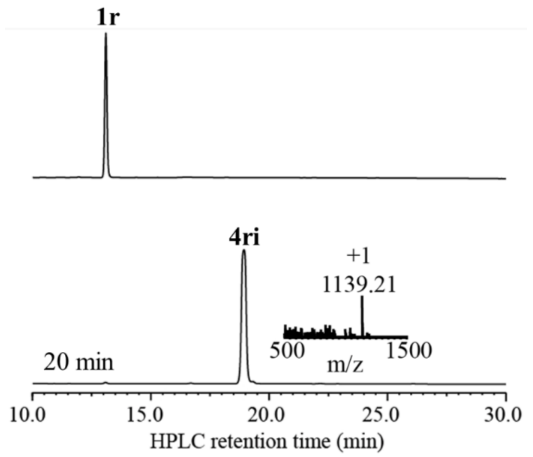

Figure S44. The modification of peptide **1r** with ynamide **2i**

26) *The modification of peptide **1r** with ynamide **2j***

The peptide **1r** (0.01 mmol) and ynamide **2j** (0.02 mmol, 2 equiv) were dissolved in 10 mL PB (0.1 M, pH 8.0) and 0.5 mL CH3CN at 37 °C. The reaction progress was monitored by HPLC (using Jupiter 5 μm C18 4.6 × 250 mm^2) with a gradient of 10-95% solvent B in 30 min at 220 nm and LC-MS. After 20 min, the product **4rj** was isolated in 77% yield. The product was characterized by ESI-MS ($[M + H]^+$ Found 1026.28, Calcd. 1026.33).

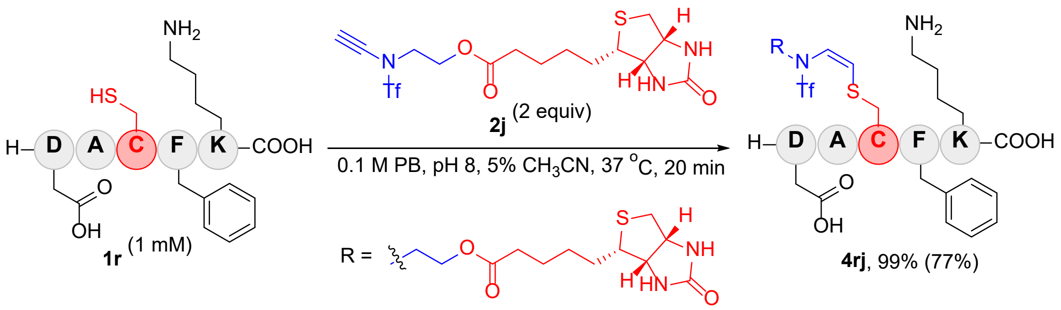

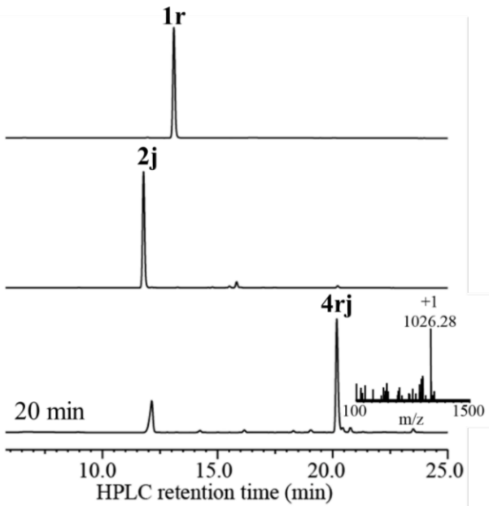

Figure S45. The modification of peptide **1r** with ynamide **2j**

27) *The modification of peptide **1r** with ynamide **2k***

The peptide **1r** (0.01 mmol) and ynamide **2k** (0.02 mmol, 2 equiv) were dissolved in 10 mL PB (0.1 M, pH 8.0) and 1 mL DMSO at 37 °C. The reaction progress was monitored by HPLC (using Jupiter 5 μm C18 4.6 × 250 mm^2) with a gradient of 10-95% solvent B in 30 min at 220 nm and LC-MS. After 20 min, the product **4rk** was isolated in 70% yield. The product was characterized by ESI-MS ($[M + K]^+$ Found 832.16, Calcd. 832.20).

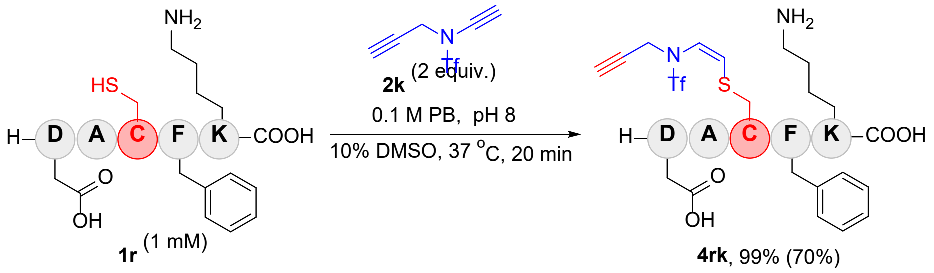

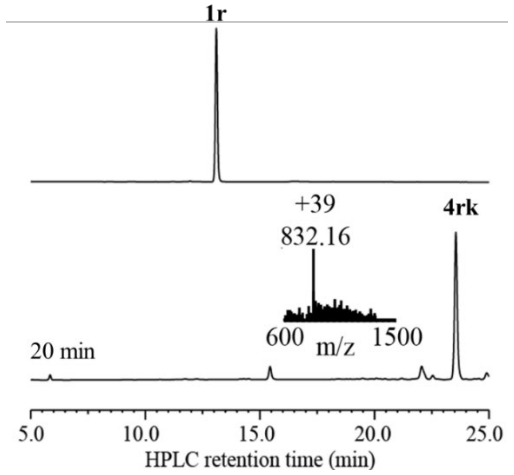

Figure S46. The modification of peptide **1r** with ynamide **2k**

*Ethyl-N-((tert-butoxycarbonyl)-L-alanyl)-S-((Z)-2-((N,4-dimethylphenyl)sulfonamido)vinyl)-L-cysteinate (**3aa**)*

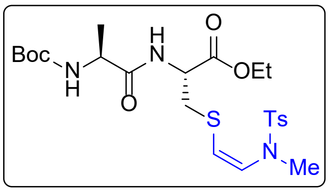

White solid, 89%, 41.7 mg, purified by flash chromatography using PE/EA (3:1) as eluent, Rf = 0.5 in PE/EA (2:1) ^{1}H NMR (400 MHz, $CDCl_3$) δ 7.62 (d, *J* = 8.2 Hz, 2H), 7.26 (d, *J* = 8.1 Hz, 2H), 6.94 (d, *J* = 5.4 Hz, 1H), 5.98 (d, *J* = 7.5 Hz, 1H), 5.57 (d, *J* = 7.4 Hz, 1H), 5.02 (s, 1H), 4.73 (dt, *J* = 7.6, 4.6 Hz, 1H), 4.14 (qd, *J* = 7.1, 3.3 Hz, 3H), 3.17 – 3.01 (m, 2H), 2.92 (s, 3H), 2.37 (s, 3H), 1.38 (s, 9H), 1.30 (d, *J* = 7.1 Hz, 3H), 1.21 (t, *J* = 7.1 Hz, 3H) ^{13}C NMR (101 MHz, $CDCl_3$) δ 171.7, 168.8, 154.4, 142.9, 133.2, 128.8, 126.4, 125.0, 118.5, 79.0, 61.0, 51.7, 49.1, 35.6, 34.8, 27.3, 20.5, 17.2, 13.1. HRMS (ESI-TOF) m/z: $C_{23}H_{35}N_3NaO_7S_2^+$ $[M + Na]^+$ Calcd. 552.1809; Found: 552.1814.

*Ethyl-N-((tert-butoxycarbonyl)-L-alanyl)-S-((Z)-2-((1,1,1-trifluoro-N-(2-hydroxyethyl)methyl)sulfonamido)vinyl)-L-cysteinate (**3ae**)*

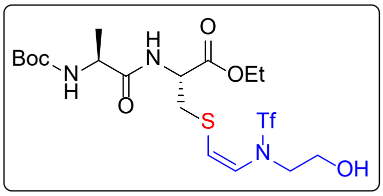

White solid, 95%, 51.0 mg, purified by flash chromatography using PE/EA (2:1) as eluent, Rf = 0.5 in PE/EA (1:1) ^{1}H NMR (400 MHz, $CDCl_3$) δ 7.47 (t, *J* = 17.0 Hz, 1H), 6.22 (d, *J* = 6.8 Hz, 1H), 6.04 (d, *J* = 6.8 Hz, 1H), 5.46 (t, *J* = 10.5 Hz, 1H), 4.84 (dt, *J* = 7.7, 4.6 Hz, 1H), 4.22 (tt, *J* = 31.5, 15.9 Hz, 3H), 3.82 (d, *J* = 6.9 Hz, 3H), 3.67 (t, *J* = 8.6 Hz, 1H), 3.31 (dd, *J* = 14.6, 4.2 Hz, 1H), 3.27 – 3.15 (m, 1H), 1.45 (s, 9H), 1.37 – 1.32 (m, 3H), 1.28 (dd, *J* = 14.3, 5.9 Hz, 3H) ^{13}C NMR (101 MHz, $CDCl_3$) δ 173.2, 169.8, 155.6, 131.2, 121.6, 120.0 (q, J = 323.0 Hz), 80.3, 62.3, 60.9, 53.1, 52.0, 49.9, 36.3, 28.3, 18.2, 14.0. HRMS (ESI-TOF) m/z: Calcd. $C_{18}H_{30}F_3N_3NaO_8S_2^+$ $[M + Na]^+$ 560.1319; Found: 560.3034.

*Ethyl-N-((tert-butoxycarbonyl)-L-alanyl)-S-((Z)-2-((1,1,1-trifluoro-N-phenethylmethyl)sulfonamido)vinyl)-L-cysteinate (**3af**)*

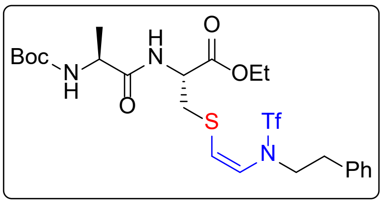

White solid, 95%, 56.8 mg, purified by flash chromatography using PE/EA (3:1) as eluent, Rf = 0.6 in PE/EA (2:1) ^{1}H NMR (400 MHz, $CDCl_3$) δ 7.24 (dd, J = 11.1, 4.2 Hz, 2H), 7.16 (dd, J = 15.1, 4.4 Hz, 3H), 7.00 (s, 1H), 6.15 (d, J = 6.9 Hz, 1H), 5.92 (d, J = 7.0 Hz, 1H), 4.98 (d, J = 9.4 Hz, 1H), 4.82 – 4.56 (m, 1H), 4.14 (m, 2H), 4.10 (s, 1H), 3.71 (dd, J = 9.0, 5.2 Hz, 2H), 3.36 – 3.04 (m, 2H), 2.85 (t, J = 7.8 Hz, 2H), 1.36 (s, 9H), 1.27 (d, J = 7.1 Hz, 3H), 1.20 (t, 3H) ^{13}C NMR (101 MHz, $CDCl_3$) δ 172.8, 169.5, 155.4, 137.1, 130.4, 128.9, 128.7, 126.9, 120.3, 119.9 (q, J = 320.0 Hz), 80.2, 62.3, 53.0, 51.5, 50.2, 36.3, 35.5, 28.3, 18.0, 14.1. HRMS (ESI-TOF) m/z: for $C_{24}H_{35}F_3N_3O_7S_2^+$ [M + H]$^+$ Calcd 598.1863; Found: 598.1855.

*Ethyl-(R,Z)-13-(2-((tert-butoxycarbonyl)amino)acetamido)-2,2-dimethyl-4-oxo-8-((trifluoromethyl)sulfonyl)-3-oxa-11-thia-5,8-diazatetradec-9-en-14-oate (**3ag**)*

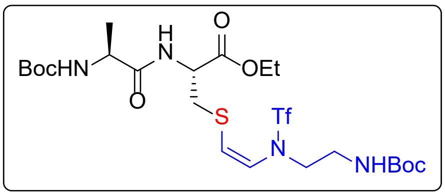

White solid, 94%, 59.8 mg, purified by flash chromatography using PE/EA (3:1) as eluent, Rf = 0.6 in PE/EA (2:1) ^{1}H NMR (400 MHz, $CDCl_3$) δ 7.25 (d, J = 24.1 Hz, 1H), 6.13 (s, 1H), 5.51 (s, 1H), 5.10 (s, 1H), 4.73 (s, 1H), 4.17 (dd, J = 14.1, 7.0 Hz, 3H), 3.69 (s, 1H), 3.61 – 3.45 (m, 1H), 3.24 (dt, J = 40.6, 14.2 Hz, 4H), 1.37 (s, 18H), 1.31 (d, J = 7.0 Hz, 3H), 1.23 (t, J = 7.1 Hz, 3H) ^{13}C NMR (101 MHz, $CDCl_3$) δ 173.3, 169.5, 156.0, 155.6, 131.2, 120.2, 119.9 (q, J = 323.0 Hz), 80.0, 62.2, 53.5, 50.1, 48.8, 38.9, 36.2, 28.3, 28.3, 18.0, 14.1. HRMS (ESI-TOF) m/z: $C_{23}H_{39}F_3N_4NaO_9S_2$ [M + Na]$^+$ Calcd. 659.2003; Found: 659.2006.

*N^5-((R)-1-((carboxymethyl)amino)-1-oxo-3-(((Z)-2-((1,1,1-trifluoro-N-(2-hydroxyethyl)methyl)sulfonamido)vinyl)thio)proPEn-2-yl)-L-glutamine (**3me**)*

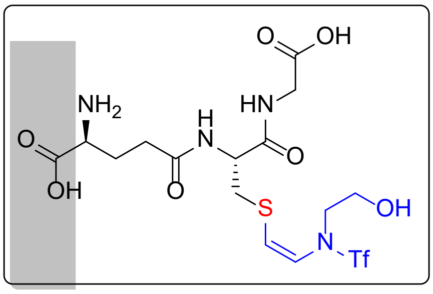

White solid, 95%, 56.8 mg, purified by recrystallization. ^{1}H NMR (400 MHz, D_2O) δ 6.57

(d, J = 6.8 Hz, 1H), 6.05 (d, J = 6.7 Hz, 1H), 4.57 (dd, J = 8.6, 5.1 Hz, 1H), 3.99 (dd, J = 11.0, 4.4 Hz, 1H), 3.96 (s, 2H), 3.75 – 3.52 (m, 4H), 3.29 (dd, J = 14.5, 5.1 Hz, 1H), 3.08 (dd, J = 14.5, 8.7 Hz, 1H), 2.62 – 2.44 (m, 2H), 2.26 – 2.08 (m, 2H) ^{13}C NMR (101 MHz, D_2O) δ 174.3, 172.9, 172.0, 171.8, 133.4, 120.3, 119.8 (q, J = 323.0 Hz), 58.4, 53.7, 52.5, 52.3, 41.2, 34.7, 31.1, 25.6. HRMS (ESI-TOF) m/z: $C_{15}H_{24}F_3N_4O_9S_2^+$ [M + H]$^+$ Calcd. 525.0931; Found: 525.0937.

*Ethyl-N-((tert-butoxycarbonyl)-L-alanyl)-S-((Z)-2-((1,1,1-trifluoro-N-(2-(2-oxo-2H-chromene-3-carboxamido)ethyl)methyl)sulfonamido)vinyl)-L-cysteinate **(4ah)***

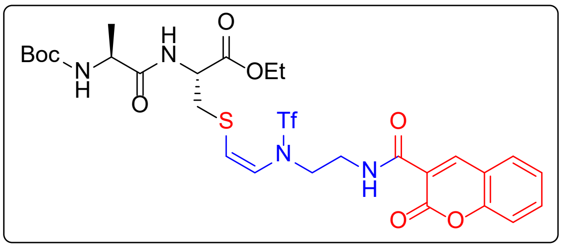

Yellow solid, 92%, 65.2 mg, purified by flash chromatography using PE/EA (2:1) as eluent, Rf = 0.6 in PE/EA (1:1) ^{1}H NMR (400 MHz, $CDCl_3$) δ 9.18 (t, J = 5.7 Hz, 1H), 8.99 (s, 1H), 7.80 (d, J = 7.8 Hz, 1H), 7.69 (t, J = 7.8 Hz, 1H), 7.52 – 7.33 (m, 2H), 6.28 (d, J = 6.9 Hz, 1H), 5.98 (d, J = 6.9 Hz, 1H), 5.63 (d, J = 5.8 Hz, 1H), 4.90 (s, 1H), 4.49 (s, 1H), 4.21 (m, 2H), 3.82 (d, J = 5.0 Hz, 2H), 3.71 (s, 2H), 3.53 – 3.27 (m, 2H), 1.45 (d, J = 11.2 Hz, 3H), 1.40 (s, 9H), 1.29 (s, 3H) ^{13}C NMR (101 MHz, $CDCl_3$) δ 173.4, 169.5, 162.4, 161.4, 155.3, 154.5, 149.1, 134.3, 132.4, 130.1, 125.3, 119.9 (q, J = 323.0 Hz), 119.7, 118.7, 117.9, 116.7, 79.7, 62.1, 53.1, 50.1, 48.5, 38.5, 36.6, 28.3, 18.8, 14.1. HRMS (ESI-TOF) m/z: [M + H]$^+$ Calcd. $C_{28}H_{36}F_3N_4O_{10}S_2^+$ 709.1819; Found: 709.1823.

*Ethyl-N-((tert-butoxycarbonyl)-L-alanyl)-S-((Z)-2-((N-(2-(2-(1-(4-chlorobenzoyl)-5-methoxy-2-methyl-1H-indol-3-yl)acetoxy)ethyl)-1,1,1-trifluoromethyl)sulfonamido)vinyl)-L-cysteinate **(4ai)***

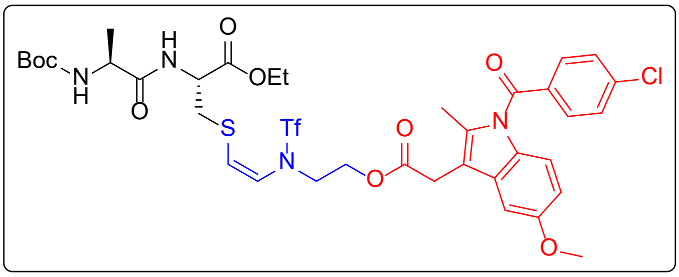

White solid, 95%, 83.3 mg, purified by flash chromatography using PE/EA (3:1) as eluent, Rf = 0.6 in

PE/EA (2:1) ^{1}H NMR (400 MHz, $CDCl_3$) δ 7.68 (d, J = 8.1 Hz, 2H), 7.47 (d, J = 8.1 Hz, 2H), 7.11 (s, 1H), 6.99 (s, 1H), 6.90 (d, J = 8.9 Hz, 1H), 6.67 (d, J = 9.0 Hz, 1H), 6.18 (d, J = 6.2 Hz, 1H), 5.87 (d, J = 6.7 Hz, 1H), 5.22 (s, 1H), 4.76 (d, J = 4.6 Hz, 1H), 4.21 (d, J = 6.8 Hz, 5H), 3.83 (s, 5H), 3.75 (s, 2H), 3.34 – 3.07 (m, 2H), 2.38 (s, 3H), 1.43 (s, 9H), 1.33 (d, J = 7.1 Hz, 3H), 1.27 (d, J = 7.4 Hz, 3H) ^{13}C NMR (101 MHz, $CDCl_3$) δ 173.0, 169.5, 168.3, 156.1, 139.3, 136.2, 133.9, 132.0, 131.2, 130.9, 130.7, 129.1, 119.9 (q, J = 323.0 Hz),119.5, 114.9, 112.0, 111.6, 101.5, 80.1, 62.2, 61.1, 55.7, 52.9, 50.2, 48.1, 36.1, 30.1, 28.3, 18.1, 14.1, 13.3. HRMS (ESI-TOF) m/z: $C_{37}H_{45}ClF_3N_4O_{11}S_2^+$ [M + H]$^+$ Calcd. 877.2161; Found: 877.2164.

(6S,9R,Z)-9-(ethoxycarbonyl)-2,2,6-trimethyl-4,7-dioxo-14-((trifluoromethyl)sulfonyl)-3-oxa-11-thia-5,8,14-triazahexadec-12-en-16-yl-4-((3aS,4S,6aR)-2-oxohexahydro-1H-thieno[3,4-d]imidazol-4-yl)butanoate ***(4aj)***

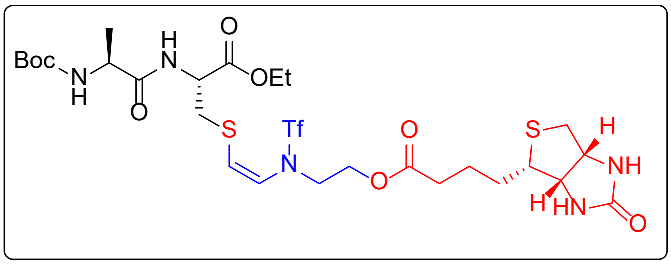

White solid, 85%, 63.8 mg, purified by flash chromatography using DCM/MeOH (20:1) as eluent, Rf = 0.7 in DCM/MeOH (10:1)

^{1}H NMR (400 MHz, $CDCl_3$) δ 7.55 (s, 1H), 6.33 (d, J = 6.2 Hz, 2H), 6.12 – 5.86 (m, 2H), 5.57 (s, 1H), 4.79 (d, J = 5.4 Hz, 1H), 4.51 (s, 1H), 4.32 (s, 1H), 4.23 (d, J = 6.4 Hz, 4H), 3.85 (s, 2H), 3.24 (m, 3H), 2.91 (dd, J = 12.4, 4.0 Hz, 1H), 2.75 (d, J = 12.7 Hz, 1H), 2.38 (t, J = 7.0 Hz, 2H), 1.68 (d, J = 6.5 Hz, 4H), 1.46 (d, J = 18.0 Hz, 9H), 1.36 (d, J = 6.7 Hz, 3H), 1.30 (t, J = 6.9 Hz, 3H) ^{13}C NMR (101 MHz, $CDCl_3$) δ 173.5, 173.4, 169.7, 164.1, 155.6, 132.0, 119.7 (q, J = 323.0 Hz), 119.7, 79.9, 62.2, 62.1, 60.7, 60.1, 55.6, 53.0, 50.1, 48.2, 40.5, 36.1, 33.8, 29.6, 28.4, 28.3, 28.2, 24.6, 18.2, 14.1. HRMS (ESI-TOF) m/z: $C_{27}H_{46}F_3N_6O_{10}S_3^+$ [M + NH_4]$^+$ Calcd. 766.2384; Found: 766.2296.

*Ethyl-N-((tert-butoxycarbonyl)-L-alanyl)-S-((Z)-2-((1,1,1-trifluoro-N-(prop-2-yn-1-yl) methyl)sulfonamido)vinyl)-L-cysteinate **(4ak)***

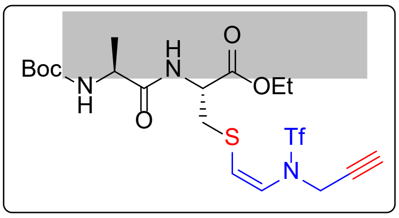

White solid, 92%, 48.9 mg, purified by flash chromatography using PE/EA (5:1) as eluent, Rf = 0.5 in PE/EA (3:1) ^{1}H NMR (400 MHz, $CDCl_3$) δ 7.07 (d, *J* = 6.0 Hz, 1H), 6.37 (d, *J* = 6.6 Hz, 1H), 6.11 (d, *J* = 6.6 Hz, 1H), 5.12 (d, *J* = 7.0 Hz, 1H), 4.95 – 4.70 (m, 1H), 4.42 (q, *J* = 18.0 Hz, 2H), 4.23 (m, 3H), 3.30 (m, 2H), 2.46 (s, 1H), 1.45 (s, 9H), 1.37 (d, *J* = 6.9 Hz, 3H), 1.30 (t, *J* = 7.1 Hz, 3H) ^{13}C NMR (101 MHz, $CDCl_3$) δ 172.8, 169.5, 155.4, 133.0, 120.0, 119.7 (q, J = 323.0 Hz), 80.2, 76.1, 74.6, 62.3, 53.0, 50.3, 39.4, 36.2, 28.3, 18.1, 14.0. HRMS (ESI-TOF) m/z: $C_{19}H_{29}F_3N_3O_7S_2^+$ $[M + H]^+$ Calcd. 532.1394; Found: 532.1395.

*2-((2R,5R,8R,11S)-5-benzyl-11-(3-guanidinopropyl)-3,6,9,12,15-pentaoxo-8-((((Z)-2-((1,1,1-trifluoro-N-(2-(2-oxo-3,8a-dihydro-2H-chromene-3-carboxamido)ethyl)methyl) sulfonamido)vinyl)thio)methyl)-1,4,7,10,13-pentaazacyclopentadecan-2-yl)acetic acid **(4oh)***

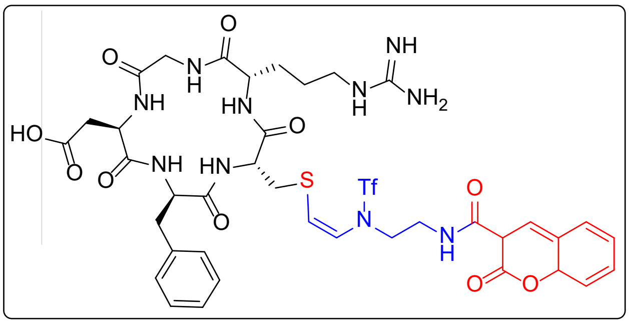

Yelow solid, 90%, 87.2 mg, purified by recrystallization. ^{1}H NMR (400 MHz, DMSO) δ 8.98 (t, *J* = 5.8 Hz, 1H), 8.92 (s, 1H), 8.37 – 8.24 (m, 1H), 8.17 (t, *J* = 7.9 Hz, 2H), 8.06 – 7.96 (m, 2H), 7.78 (t, *J* = 7.8 Hz, 1H), 7.59 (s, 1H), 7.56 – 7.42 (m, 2H), 7.24 (m, 6H), 6.76 (d, *J* = 6.8 Hz, 1H), 6.13 (d, *J* = 6.7 Hz, 1H), 4.67 (d, *J* = 6.0 Hz, 1H), 4.53 (d, *J* = 7.0 Hz, 1H), 4.26 (d, *J* = 4.9 Hz, 1H), 4.21 – 4.07 (m, 1H), 3.78 (t, *J* = 5.3 Hz, 2H), 3.55 (dd, *J* = 11.1, 5.5 Hz, 2H), 3.28 (dd, *J* = 15.0, 3.1 Hz, 1H), 3.02 (dd, *J* = 13.5, 7.6 Hz, 1H), 2.82 (dd, *J* = 14.7, 7.3 Hz, 2H), 2.78 – 2.65 (m, 2H), 2.39 (dd, *J* = 16.1, 5.5 Hz, 1H), 2.22 (t, *J* = 8.4 Hz, 1H), 1.93 – 1.68 (m, 1H), 1.46 (dd, *J* = 28.1, 21.2 Hz, 3H), 1.29 –

1.12 (m, 3H) ^{13}C NMR (101 MHz, DMSO) δ 173.1, 172.4, 171.8, 171.0, 163.3, 157.6, 139.0, 129.3, 128.4, 126.4, 119.8 (q, J = 320.0 Hz), 119.2, 65.4, 61.5, 60.5, 59.7, 56.5, 55.8, 53.3, 48.4, 43.3, 36.7, 33.7, 28.4, 24.7, 19.0, 15.6. HRMS (ESI-TOF) m/z: $C_{39}H_{47}F_3N_{10}O_{12}S_2^-$ [M - H]$^-$ Calcd. 967.2696; Found: 967.2644.

*2-((2R,5R,8R,11S)-5-benzyl-8-((((Z)-2-((N-(2-(2-(1-(4-chlorobenzoyl)-5-methoxy-2-methyl-1H-indol-3-yl)acetoxy)ethyl)-1,1,1-trifluoromethyl)sulfonamido)vinyl)thio)methyl)-11-(3-guanidinopropyl)-3,6,9,12,15-pentaoxo-1,4,7,10,13-pentaazacyclopentadecan-2-yl)acetic acid (**4oi**)*

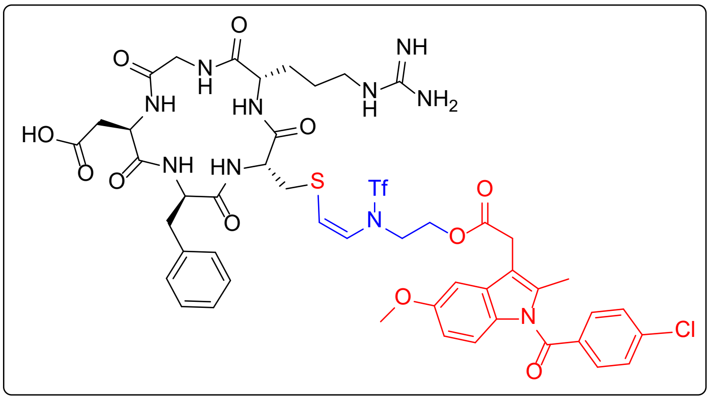

White solid, 89%, 101.0 mg, purified by recrystallization. ^{1}H NMR (400 MHz, DMSO) δ 8.43 – 8.19 (m, 3H), 8.10 (d, *J* = 8.1 Hz, 1H), 7.83 (d, *J* = 8.0 Hz, 1H), 7.72 – 7.61 (m, 4H), 7.50 (d, *J* = 26.3 Hz, 1H), 7.29 – 7.08 (m, 6H), 7.02 (d, *J* = 2.1 Hz, 1H), 6.92 (d, *J* = 9.0 Hz, 1H), 6.80 – 6.59 (m, 2H), 6.04 (d, *J* = 6.8 Hz, 1H), 4.62 (d, *J* = 7.9 Hz, 1H), 4.52 (dd, *J* = 14.2, 7.0 Hz, 1H), 4.14 (m, 3H), 3.41 – 3.33 (m, 1H), 3.31 – 3.19 (m, 1H), 3.17 – 3.05 (m, 3H), 2.76 (dd, *J* = 13.9, 7.1 Hz, 1H), 2.66 (dd, *J* = 15.9, 8.4 Hz, 1H), 2.34 (dd, *J* = 16.1, 5.4 Hz, 1H), 2.23 (s, 3H), 1.83 (d, *J* = 19.3 Hz, 1H), 1.72 (d, *J* = 12.0 Hz, 2H), 1.68 – 1.59 (m, 2H), 1.54 (d, *J* = 8.7 Hz, 1H), 1.49 – 1.37 (m, 3H), 1.27 – 1.19 (m, 1H), 1.12 (m, 3H) ^{13}C NMR (101 MHz, DMSO) δ 172.1, 171.2, 171.1, 170.8, 170.5, 170.3, 169.7, 168.3, 157.2, 156.1, 138.2, 138.1, 136.1, 134.5, 133.2, 131.7, 131.0, 130.7, 129.5, 129.5, 128.5, 126.6, 119.8 (q, J = 320.0 Hz), 119.5, 117.7, 112.8, 112.0, 102.0, 78.3, 68.7, 62.3, 61.2, 55.8, 54.2, 52.9, 49.4, 48.4, 35.8, 30.7, 25.8, 24.3, 13.6. HRMS (ESI-TOF) m/z: $C_{48}H_{55}ClF_3N_{10}O_{13}S_2^+$ [M + H]$^+$ Calcd. 1135.3027; Found: 1135.3031.

*2-((2R,5R,8R,11S)-5-benzyl-11-(3-guanidinopropyl)-3,6,9,12,15-pentaoxo-8-((((Z)-2-((1,1,1-trifluoro-N-(2-((5-((3aS,4S,6aR)-2-oxohexahydro-1H-thieno[3,4-d]imidazol-4-yl)pentanoyl)oxy)ethyl)methyl)sulfonamido)vinyl)thio)methyl)-1,4,7,10,13-pentaazacyclopentadecan-2-yl)acetic acid (**4oj**)*

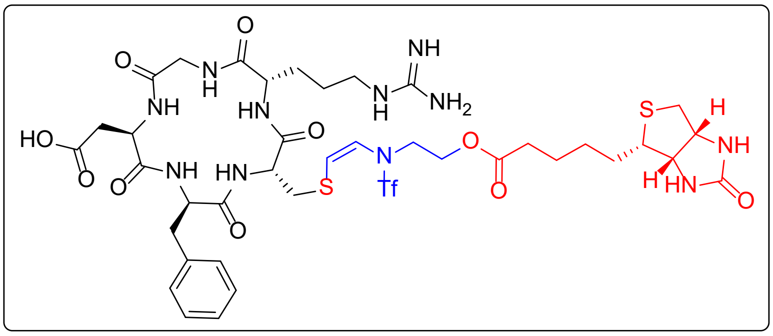

White solid, 92%, 94.4 mg, purified by recrystallization. ^{1}H NMR (400 MHz, DMSO) δ 8.37 (d, *J* = 119.4 Hz, 2H), 7.77 (s, 2H), 7.19 (dd, *J* = 15.5, 6.5 Hz, 6H), 6.73 (d, *J* = 5.8 Hz, 1H), 6.45 (d, *J* = 32.0 Hz, 2H), 6.02 (d, *J* = 5.6 Hz, 1H), 4.57 (d, *J* = 68.1 Hz, 2H), 4.31 (s, 1H), 4.13 (t, *J* = 37.2 Hz, 4H), 3.76 (s, 1H), 3.41 (m, 6H), 3.21 (s, 2H), 3.09 (s, 3H), 2.81 (s, 1H), 2.63 – 2.53 (m, 2H), 2.30 (s, 2H), 2.05 (d, *J* = 32.0 Hz, 1H), 1.73 (d, *J* = 36.2 Hz, 1H), 1.68 – 1.20 (m, 8H), 1.12 – 0.98 (m, 4H) ^{13}C NMR (101 MHz, DMSO) δ 173.1, 172.4, 171.8, 171.0, 163.3, 157.6, 139.0, 129.3, 128.4, 126.4, 119.9 (q, J = 320.0 Hz), 119.2, 116.0, 65.4, 61.5, 60.5, 59.7, 56.5, 55.8, 53.3, 48.4, 43.3, 36.7, 33.7, 28.4, 24.7, 19.0, 15.6. HRMS (ESI-TOF) m/z: $C_{39}H_{55}F_3N_{11}O_{12}S_3^+$ $[M + H]^+$ Calcd. 1022.3140; Found: 1022.3117.

*2-((2R,5R,8R,11S)-5-benzyl-11-(3-guanidinopropyl)-3,6,9,12,15-pentaoxo-8-((((Z)-2-((1,1,1-trifluoro-N-(prop-2-yn-1-yl)methyl)sulfonamido)vinyl)thio)methyl)-1,4,7,10,13-pentaazacyclopentadecan-2-yl)acetic acid (**4ok**)*

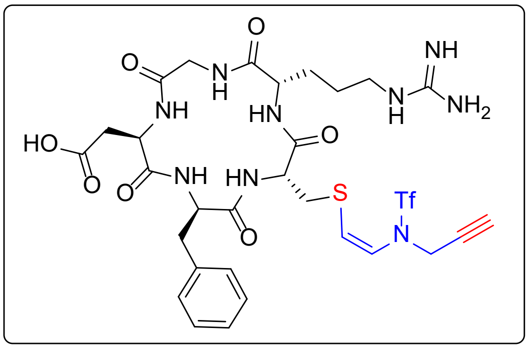

White solid, 92%, 72.7 mg, purified by recrystallization. ^{1}H NMR (400 MHz, DMSO) δ 8.31 – 8.19 (m, 1H), 8.19 – 8.06 (m, 1H), 7.96 (dd, *J* = 8.0, 4.4 Hz, 1H), 7.57 (s, 1H), 7.35 – 7.12 (m, 5H), 6.79 (d, *J* = 6.7 Hz, 1H), 6.14 (d, *J* = 6.6

Hz, 1H), 4.64 (dd, J = 14.1, 8.1 Hz, 1H), 4.58 – 4.47 (m, 1H), 4.42 (d, J = 1.9 Hz, 2H), 4.32 – 3.95 (m, 2H), 3.52 (t, J = 2.1 Hz, 1H), 3.36 – 3.20 (m, 1H), 3.09 (s, 2H), 3.02 (d, J = 7.6 Hz, 1H), 2.82 (dd, J = 14.7, 7.3 Hz, 3H), 2.76 – 2.62 (m, 2H), 2.43 – 2.29 (m, 1H), 2.19 (t, J = 8.4 Hz, 1H), 1.78 (d, J = 6.9 Hz, 1H), 1.56 – 1.33 (m, 2H), 1.23 (d, J = 7.3 Hz, 3H) ^{13}C NMR (101 MHz, DMSO) δ 172.1, 171.4, 171.3, 170.6, 170.5, 169.8, 157.1, 137.9, 134.5, 129.7, 129.6, 128.6, 126.72, 119.8 (q, J = 320.0 Hz), 119.0, 77.4, 77.1, 57.6, 54.7, 52.7, 49.4, 43.7, 37.4, 35.8, 28.4, 27.56, 26.3, 25.1, 16.1. HRMS (ESI-TOF) m/z: $C_{30}H_{39}F_3N_9O_9S_2^+$ [M + H]$^+$ Calcd. 790.2259; Found: 790.2278.

5. One-Pot Dual Functionalization of Cys with Click Chemistry

Ynamide **2k** (1.9 mg, 9 µmol, 0.45 M) was dissolved in 200 µL MeCN as **2k** stock solution. $CuSO_4 \cdot 5H_2O$ (5.1 mg, 20.4 µmol, 20 mM) was dissolved in water as $CuSO_4$ stock solution. Sodium ascorbate (41 mg, 207 µmol, 100 mM) was dissolved in 2.07 ml water as ascorbate stock solution. Bathophenanthroline disulfonic acid disodium salt (BPS ligand, 1.6 mg, 2.98 µmol, 50 mM) was dissolved in 59.6 µl water as BPS ligand stock solution.

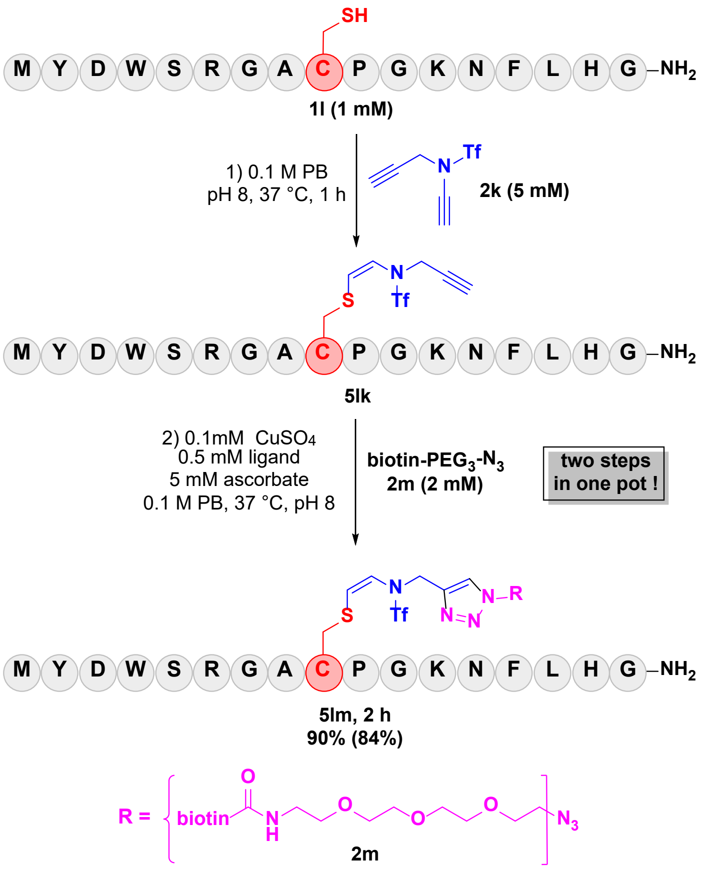

Figure 47. One-pot dual modification of peptide **1l** with **2k** and **2m**

Peptide **1l** (3.6 mg, 1.86 µmol, 1 mM) was dissolved in 1836.4 µL PB (0.1 M, pH 8) and 20.6 µL of **2k** stock solution was then added to the peptide solution. The reaction mixture was incubated at 37 °C and monitored by using RP-HPLC (XSelect C18 column (3.5 µm, 130 Å, 4.6 × 150 mm) with a gradient of 5-70% B (0.1% TFA in

MeCN) over 20 min at 220 nm), in which 10 μL aliquots of the reaction mixture were taken and quenched by 50 μL 0.1% TFA in water. The reaction was completed after 1 h and the conjugated product **5lk** was found (mass Found 2148.4 Da, Calcd. 2147.8 Da) The results are shown in **Figure S48**. Next, copper catalyzed azide-alkyne cyclization (CuAAC)[6] was performed in one-pot by adding the following: 12.3 μL of $CuSO_4$ stock solution (final concentration 0.1 mM), 123 μL of ascorbate stock solution (final concentration 5 mM), 24.6 μL of BPS ligand stock solution (final concentration 0.5 mM) and biotin-PEG_3-N_3 (1 mg, 1.2 equiv, 2.232 μmol) The reaction mixture was incubated at 37 °C. The click reaction took 30 min to give a single desired product in 84% isolated yield (mass Found 2593.6 Da, Calcd. 2593.9 Da).

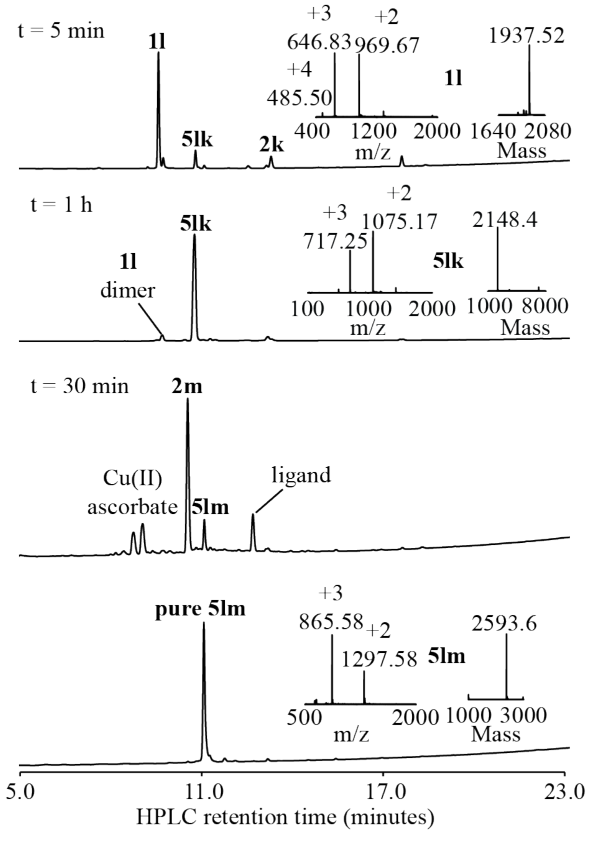

Figure S48. One pot dual modification of peptide **1l** with click chemistry

6. Modification of Cys-containing Proteins.

1) *Modification of ubiquitin(G47C) (**6**) by ynamide **2j***

Ynamide **2j** (11.7 mg, 26.4 μmol, 53 mM) was dissolved in 500 μL MeCN as **2j** stock solution. Ubiquitin(G47C) (**6**, 0.12 mg, 0.014 μmol) was dissolved in 8.4 μL degassed PB (0.1 M, 6 M Gn·HCl, pH 8), which was then diluted in 46.3 mL PB (0.1 M, pH 8) to afford ubiquitin(G47C) (**6**) solution (250 μM, 0.9 M Gn·HCl, pH 8) The protein solution was incubated for 1 h at room temperature to allow folding. Next, 1.3 μL of **2j** stock solution (5 equiv) was added to the above protein solution. The reaction mixture was incubated at 37 °C and the reaction progress was monitored by RP-HPLC (X-Bridge C4 column, 3.5 μm, 4.6 × 150 mm) with a gradient of 5-70% of B (1% TFA in MeCN) over 25 min at 220 nm **(**Figure S49) After 12 h, the modified ubiquitin(G47C) (**7**) was isolated in 43% yield (mass Found 9054.1 Da, Calcd. 9054.4 Da, Figure S105). The results are shown in **Figure S49 and S105**.

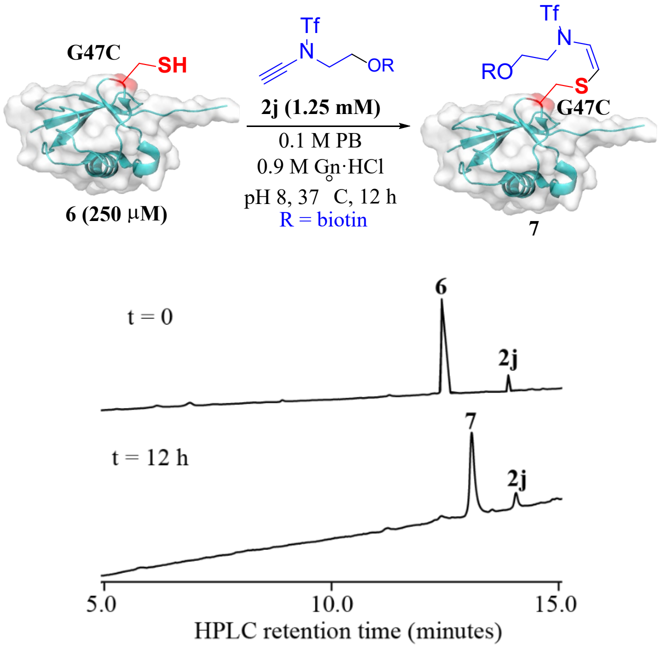

Figure S49. Modification of ubiquitin(G47C) (**6**) with biotinylated ynamide **2j**.

2) *The reaction of ubiquitin(G47A) by ynamide* ***2j***

The preparation of ubiquitin(G47A) (**6'**) was reported previously by the deselenization[7] of selenocysteine (Sec, U)-containing ubiquitin(G47U) variant[8]. The modification of ubiquitin(G47A) was performed under the same conditions as with ubiquitin(G47C) (**6**), however, no modification of ubiquitin(G47A) was found even after 18 h incubation (**Figure S50**).

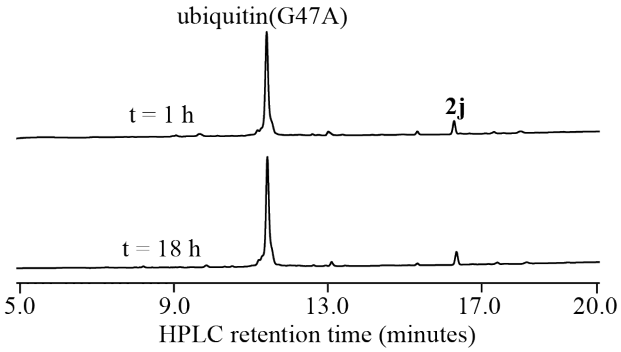

Figure S50. The modification reaction of ubiquitin(G47A) with ynamide **2j**

3) *The modification of BPTI by ynamide* ***2e***

Ynamide **2e** (15.7 mg, 72.4 μmol, 241 mM) was dissolved in 300 μL MeCN as **2e** stock solution. Folded BPTI (0.3 mg, 0.046 μmol, 250 μM) was dissolved in 182 μL PB (0.1 M, pH 8) and 1.5 equiv of TCEP was added to the reaction at room temperature, as it was reported[9]. After 3 h, the solvent exposed disulfide bond (Cys14-Cys38) was reduced (other two disulfide bonds, Cys5-Cys55 and Cys30-Cys51, remained intact) Next, 1.87 μL of **2e** stock solution (10 equiv) was added to the above reaction. The reaction mixture was incubated at 37 °C and the reaction progress was monitored by RP-HPLC (X-Bridge C4 column, 3.5 μm, 4.6 × 150 mm) using a gradient of 5-70% B (0.1% TFA in MeCN) over 25 min at 220 nm (Figure S48). After the completion of the reaction, the doubly modified BPTI was isolated in 21.5% yield by semi-prep HPLC. The concentration of the protein was determined by using UV-Vis spectrophotometer (201 Thermo Scientific Evolution TM), and the ε_{280} of WT-BPTI analogs is 5400 cm^{-1} M^{-1}.

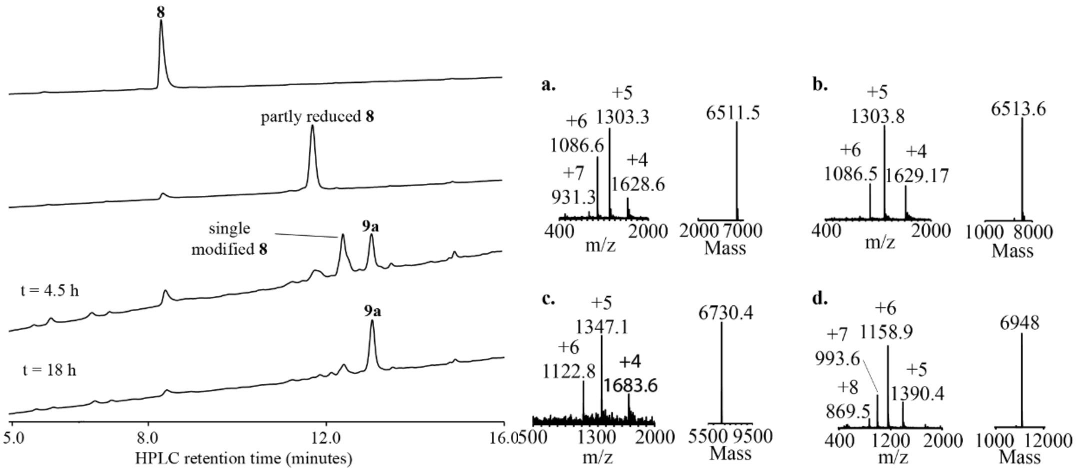

Figure S51. Selective modification of the Cys14 and Cys38 residues in BPTI with **2e**. ESI-MS of WT-BPTI (**8**), partly reduced **8**, single modified BPTI and doubly modified BPTI (**9a**): **a**. WT-BPTI (**8**, Found 6511.5 Da, Calcd. 6511.5 Da), **b**. partly reduced **8** (Found 6513.6 Da, Calcd. 6513.5 Da), **c**. Single modified BPTI with **2e** (Found 6730.4 Da, Calcd. 6730.7 Da), **d**. doubly modified BPTI (at Cys14 & Cys38) with **2e** (Found 6948.0 Da, Calcd. 6947.8 Da).

4) *The dual biotinylation of BPTI with biotinylated ynamide **2j***

The dual biotinylation of the BPTI was performed by using **2j** under the same conditions as with **2e**. After 18 h, the reaction was completed and purified to afford the dual biotinylated BPTI (**9b**) in 29% isolated yield (mass Found 7400.6 Da, Calcd. 7400.4 Da) The results are shown in **Figure S52**. and **Figure S110**

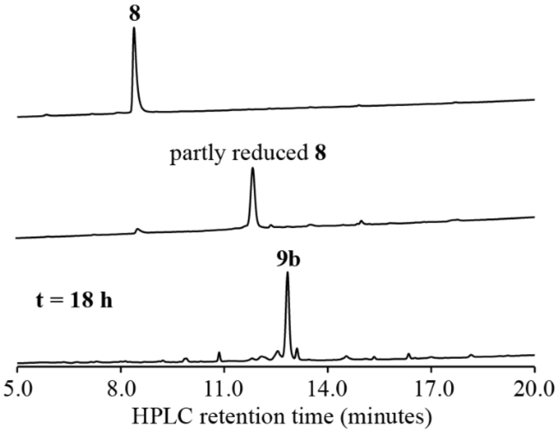

Figure S52. Dual biotinylation of BPTI by ynamide **2j**

5) Trypsin digestion of modified BPTI– C14 and C38 are in *red*, and highlighted in *yellow* (Swiss plot)

```
         10         20         30         40         50
RPDFCLEPPY TGPCKARIIR YFYNAKAGLC QTFVYGGCRA KRNNFKSAED
CMRTCGGA
```

mass	position	Name	modifications	peptide sequence
1722.7978	1-15	BPTI(1-15)		RPDFCLEPPYTGPCK
1374.6293	27-39	BPTI(27-39)		AGLCQTFVYGGCR
811.3073	47-53	BPTI(47-53)		SAEDCMR
805.3879	21-26	BPTI(21-26)		YFYNAK
522.2671	43-46	BPTI(43-46)		NNFK

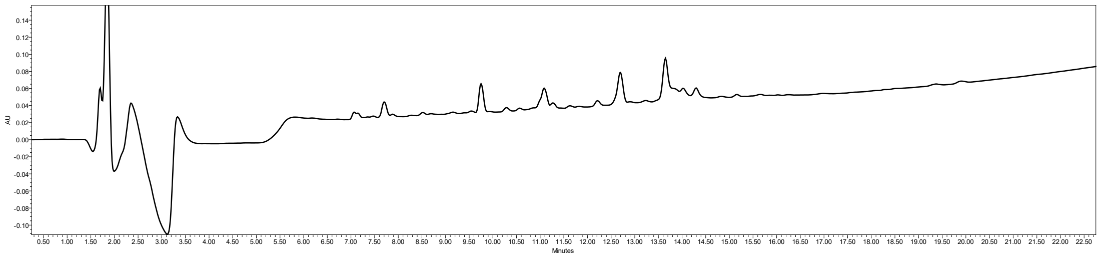

Figure S53. Trypsin digestion map for BPTI

5) HPLC chromatogram and MS analysis for modification reactions of BPTI

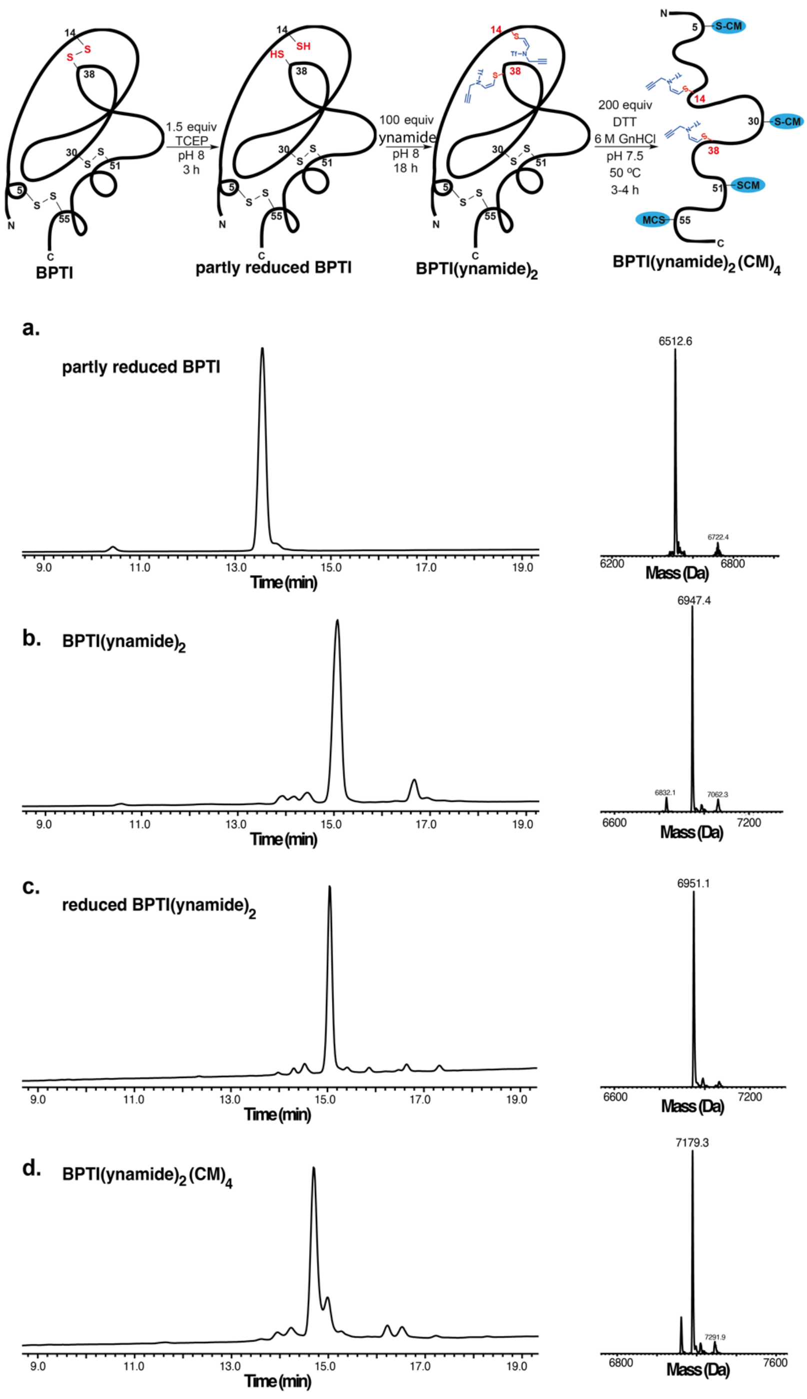

Fig. S54. HPLC chromatogram and MS analysis for modification reactions of BPTI. BPTI contains three disulfide bonds (5-55, 14-38, 30-51). Disulfide 14-38 can be selectively reduced using 1.5 equiv TCEP (Mousa, R. et. al. *Chem. Sci.* **2018**, *9*, 4814-4820), and treated with ynamide (**a** and **b**). Next disulfide bonds 5-55 and 30-55 are reduced using excess DTT, upon which IAA is added (**c** and **d**). Finally the BPTI(ynamide)$_2$(CM)$_4$ product is isolated.

6) HPLC and MS/MS analysis of trypsin digested peptides of BPTI(ynamide)$_2$(CM)$_4$.

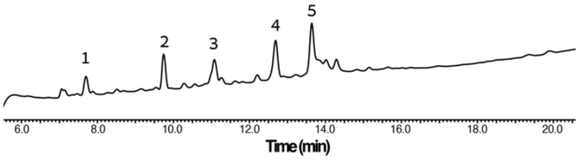

10 20 30 40 50

RPDFCLEPPY TGPCKARIIR YFYNAKAGLC QTFVYGGCRA KRNNFKSAED CMRTCGGA

mass	position	Name	modifications	peptide sequence	
1722.7978	1-15	BPTI(1-15)		RPDFCLEPPYTGPCK	Peak 4, observed
1374.6293	27-39	BPTI(27-39)		AGLCQTFVYGGCR	Peak 5, observed
811.3073	47-53	BPTI(47-53)		SAEDCMR	Peak 3, observed
805.3879	21-26	BPTI(21-26)		YFYNAK	
522.2671	43-46	BPTI(43-46)		NNFK	

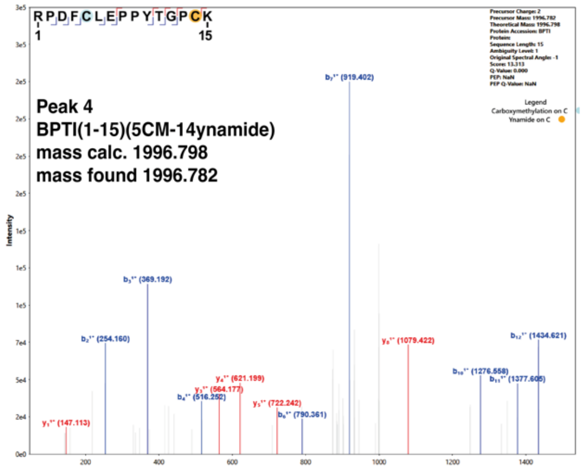

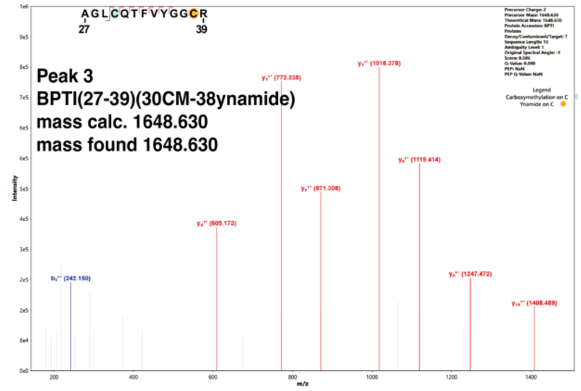

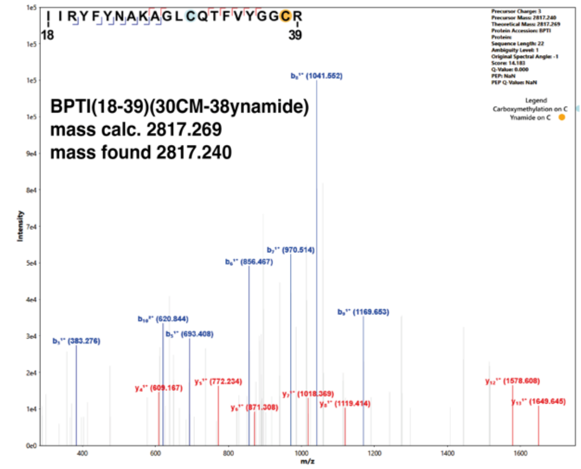

Fig. S55. HPLC and MS/MS analysis of trypsin digested peptides of BPTI(ynamide)$_2$(CM)$_4$. The product of modified BPTI(ynamide)$_2$(CM)$_4$ was treated by trypsin and the collected peptides were analyzed by MS and MS/MS. The MS/MS of the most important peptides: BPTI(1-15)(5CM-14ynamide) and BPTI(27-39)(30CM-38ynamide) and BPTI(18-39)(30CM-38ynamide) are provided, clearly showing that ynamide selectively modified C14 and C38 only, while other Cys we carboxymethylated.

7. Stability Study and Reaction Rate study of the Peptide Conjugates

1) Stability study of the peptide conjugate **3ae**

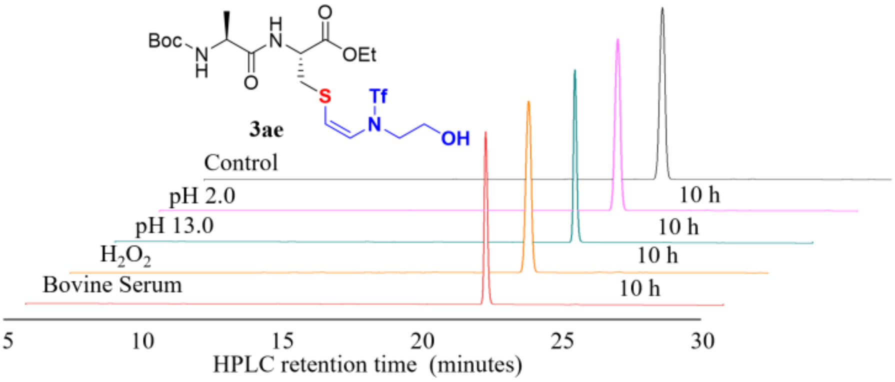

Figure S56. Stability study of the peptide conjugate **3ae**

2) Stability of **3ae** (1 mM) in buffers at pH 2.0, pH 8.0, pH 10.0, pH 13.0 at 37 °C for 10 h

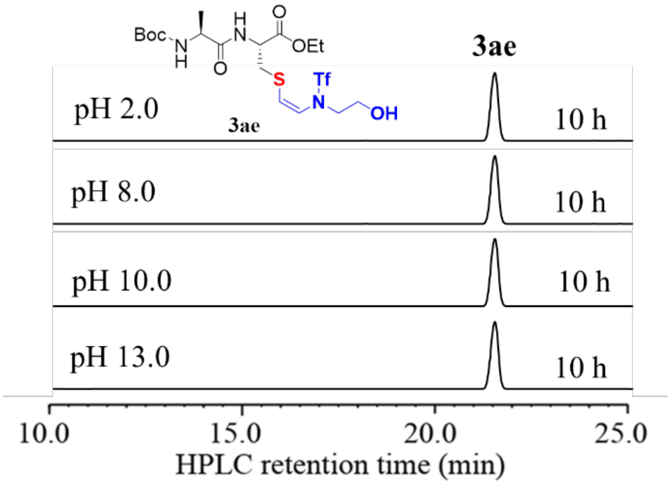

Figure S57. Stability of **3ae** (1 mM) in buffers at pH 2.0, pH 8.0, pH 10.0, pH 13.0

3) Stability of **3ae** (1 mM) in the presence of external thiol (2-mercaptoethanol)，pH 8.0

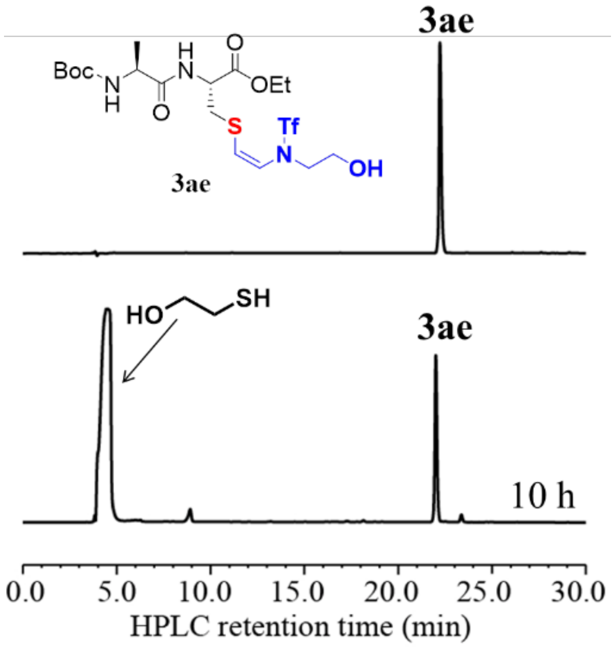

Figure S58. Stability of **3ae** (1 mM) in the presence of external thiol

4) Stability of **3ae** (10 µM) when treated with large excess ((10mM H_2O_2 in buffer) of H_2O_2 (at pH 4, 8 and 10 at 37 ºC) for 24 h

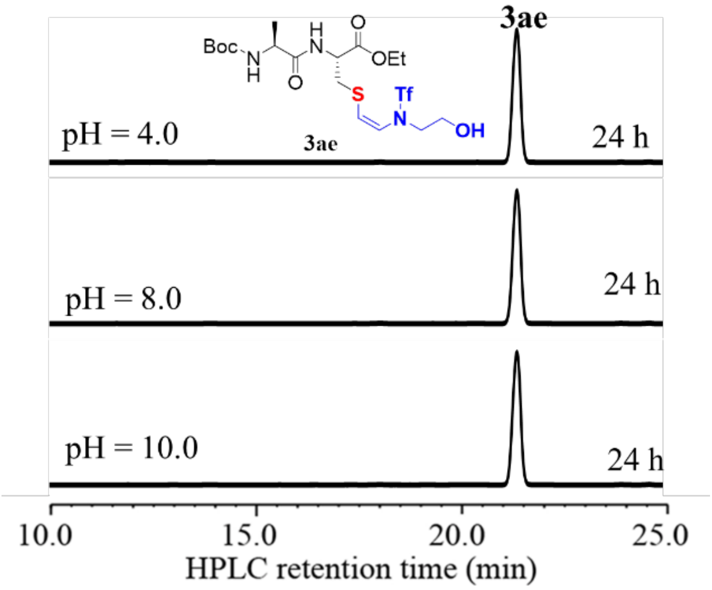

Figure S59. Stability of **3ae** (10 µM) when treated with large excess of H_2O_2 (at pH 4, 8 and 10 at 37 ºC)

5) Stability of **3ae** (10 µM) when treated with bovine serum for 24 h

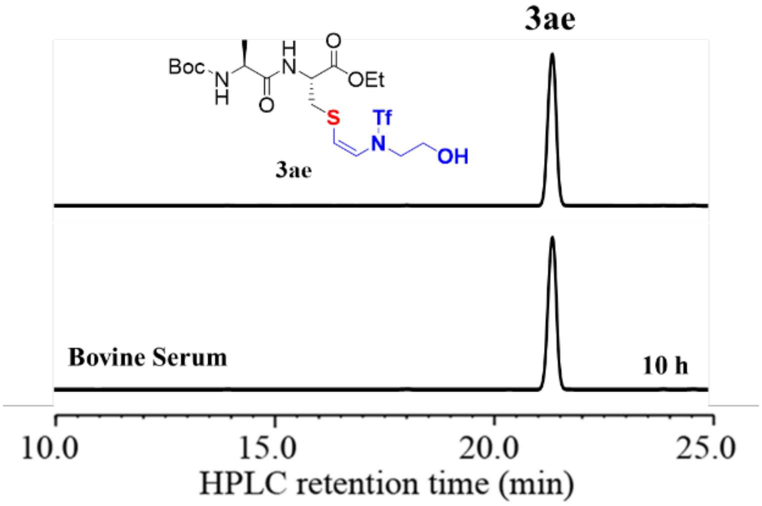

Figure S60. Stability of **3ae** (10 µM) when treated with bovine serum

6) Stability of iodoacetamide labelling product (1 mM) in buffers at pH 2.0, pH 13.0 at 37 °C for 10 h

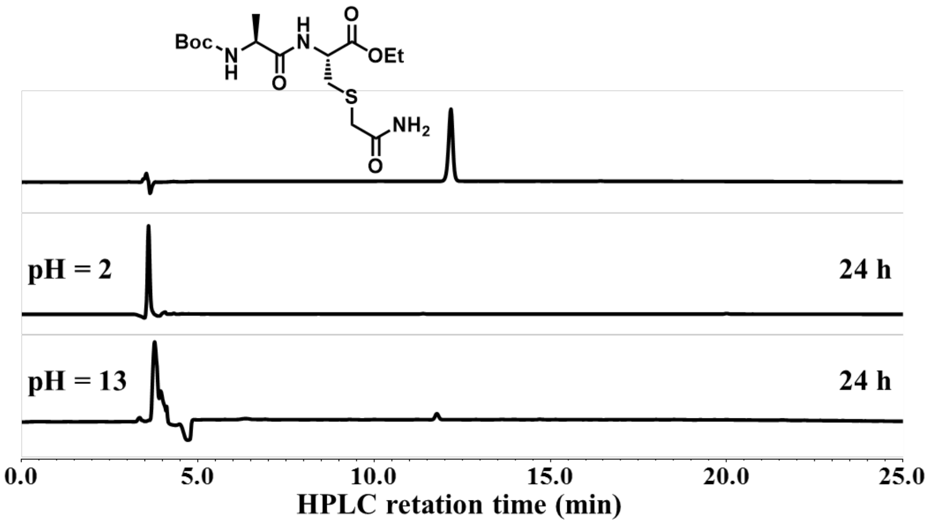

Figure S61. Stability of iodoacetamide labelling product (1 mM) in buffers at pH 2.0, pH 13.0

7) Stability of iodoacetamide labelling product (10 μM) when treated with large excess ((10mM H_2O_2 in buffer) of H_2O_2 (at pH 2 and 13 at 37 ºC) for 24 h

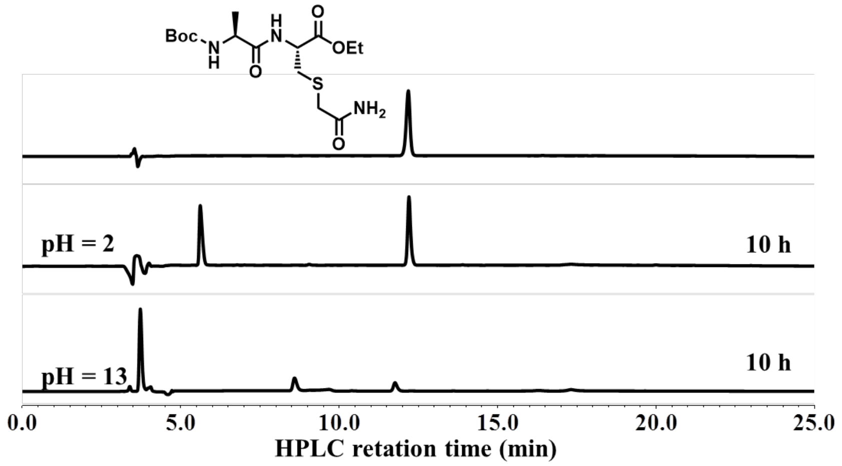

Figure S62. Stability of iodoacetamide labelling product (10 μM) when treated with large excess of H_2O_2 (at pH 2 and 13 at 37 ºC)

8) Stability of iodoacetamide labelling product (10 μM) when treated with bovine serum

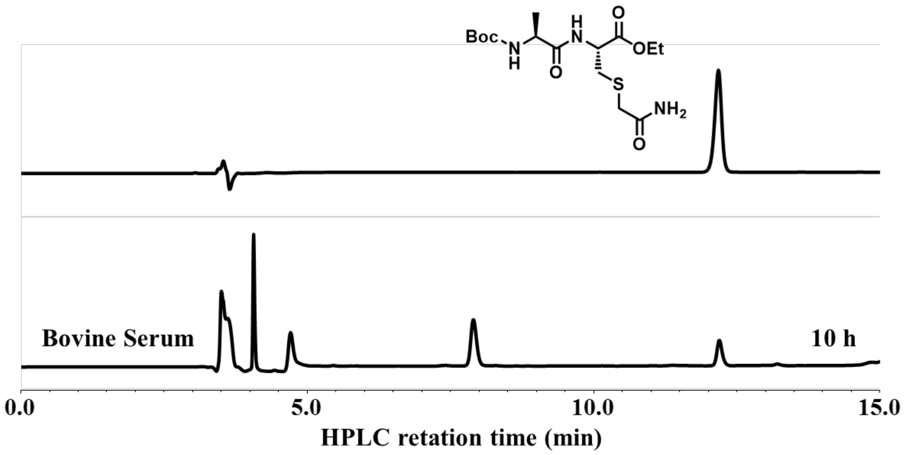

Figure S63. Stability of iodoacetamide labelling product (10 μM) when treated with bovine serum

9) Reaction Rate study of the Peptide Conjugates

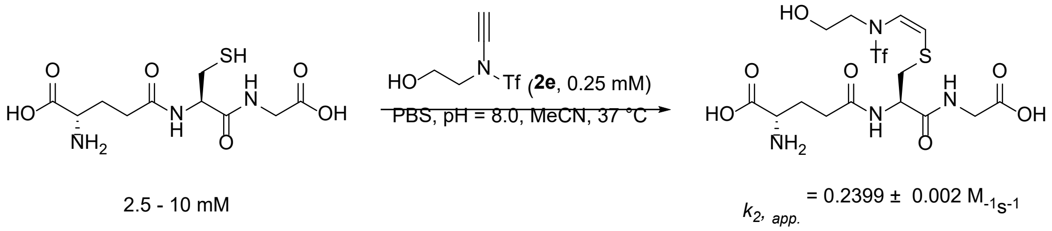

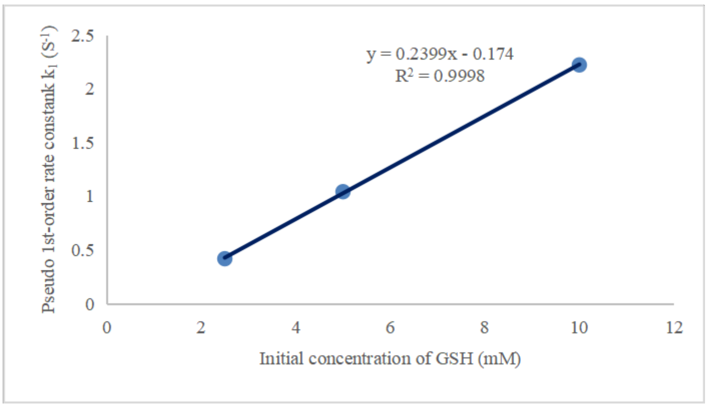

Figure S64. Reaction Rate study of the Peptide Conjugates

The reaction between Ynamide (**2e**) and glutathione (GSH) was measured under pseudo-first order conditions with 10, 20 and 40 equivalents of GSH by real-time monitor of conjugate reaction using UV-Visible absorbance at 244 nm (max absorbance wavelength for **3me**). Solutions were prepared in MeCN (5%), pH 8.0, 0.2 M PBS with Ynamide (0.25 mM) and GSH (2.5 mM, 5 mM and 10 mM). Data was recorded for 2-5 min and performed in triplicate at 37 °C. The averages of the observed rates (k_{obs}), determined by nonlinear regression analysis of the data points were plotted against the concentration of GSH and the bimolecular rate constant k_2, obtained from the slope of the plot was found to be 0.2399 ± 0.002 $M^{-1}s^{-1}$.

8. Trastuzumab Reduction

TCEP-HCl (0.8 μL out of a 1 mg/mL stock solution in H_2O, 3 nmol, 10 equiv) was added to 88 μL of Trastuzumab (0.044 mg, 0.3 nmol, c = 0.5 mg/ mL in 0.1 M PB at pH 8.0) and incubated at 37 °C for 40 min. For control reaction, this reduction step was skipped.

Trastuzumab conjugation to ynamide 2e

To the above reduced Trastuzumab was added the biotinylated ynamide (2.7 μL from 5 mg/mL solution in 30 % MeCN/H_2O, 60 nmol, 200 equiv), and the reaction was

incubated at 37 °C overnight. After conjugation, the Trastuzumab was separated from excessive ynamide and buffer exchanged to 0.1 M PB, pH 8.0 by Amicon Ultra-0.5 mL centrifugal filter (3K MWCO, Merck). To do so, we first fill the sample with PB buffer to make the final volume 500 μL, and transfer the 500 μL of sample to the centrifugal filter device and cap it. Spin the device at 14,000 x g for 10 min. The remaining volume is approximately 114 μL (antibody concentration is around 0.4 mg/mL). Discard the filtrate and refill the volume to 500 μL with pH 8.0 PB. Repeat the centrifugation using the above-described procedure. To recover the concentrated sample, place the device upside down in a clean microcentrifuge tube. Centrifuge at 1,500 x g for 2 min to transfer the sample from the device to the tube.

MS analysis of sample

To simplify the analysis, deglycosylation was first performed to remove the N-glycan heterogeneity of antibody. To do so, 30 μL of antibody was mixed with 0.5 μL of PNGase F solution (500,000 units/mL, New England Biolabs). The enzymatic reaction was incubated at 37 °C for 2-4 hours after which samples were diluted with water to a final volume of 100 μL. Prior to LC-MS analysis, 2 μL of TCEP (1 mg/mL) was added and let the reaction stand for 30 mins to maintain the reaction in a reducing environment (except for analyses of intact mass and negative control). 5 μL of the above sample was subjected for LC-MS analysis.

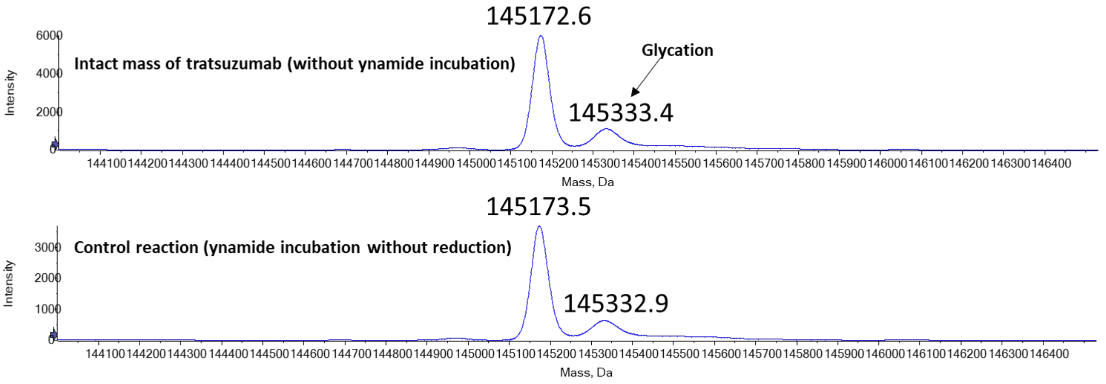

Figure S65. Deconvoluted deglycosylated intact MS spectra of tratsuzumab starting material and negative control without TCEP reduction

9. The Chemoselectivity Evaluation of Cys Modification by Different Reagents

We investigated the chemoselectivity of three classical reactive reagents toward Cys residue. The reactions were performed with two model peptides (**14** and **21**) at different pH, which were monitored by analytical RP-HPLC (C18 column) with a gradient of 5-70% B (0.1% TFA in MeCN) over 20 min at 220 nm.

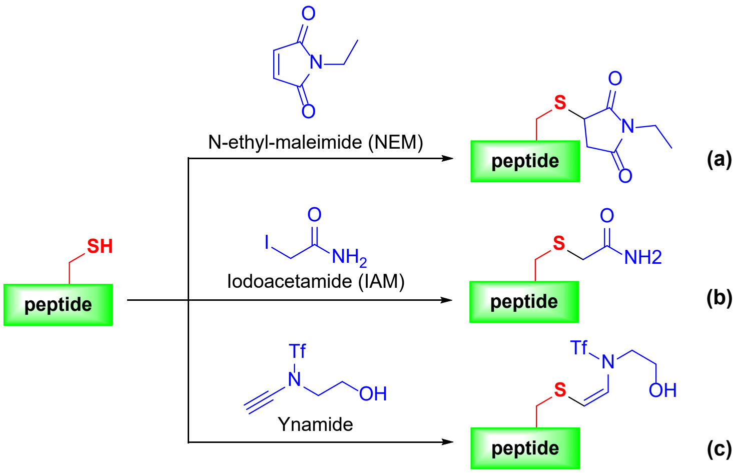

Figure S66. Modification reactions of Cys via different reagents: (**a**) *N*-ethylmaleimide (NEM), (**b**) iodoacetamide (IAM), and (**c**) ynamide **2e**

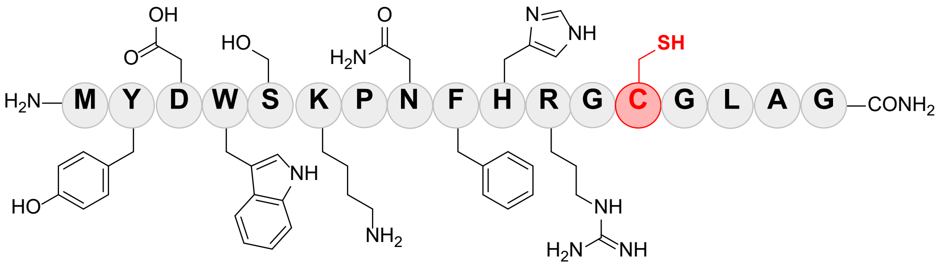

Figure S67. The sequence of model peptide **14**

Model peptide **14**, containing all reactive amino acid side chains, was prepared according to the general procedure described previously. The crude peptide **14** was purified by Prep RP-HPLC (C18 column) to obtain **14** in ~ 38% yield (mass Found 1937.5 Da, Calcd. 1936.8 Da). Peptide **14** (3.5 mg, 1.8 µmol, 16.3 mM) was dissolved in 110.4 µL water as peptide **14** stock solution. Ynamide (**2e,** 8.9 mg, 41 µmol, 105 mM) was dissolved in 390.6 µL MeCN as ynamide **2e** stock solution. NEM (22.8 mg,

182.2 μmol, 105 mM) was dissolved in 1735.7 μL MeCN as NEM stock solution. IAM (56.6 mg, 306 μmol, 105 mM) was dissolved in 2914.4 μL MeCN as IAM stock solution. Three aliquots of 9.2 μL of peptide **14** stock solution (0.15 μmol) were added to three aliquots of 133.7 μL PB (0.1 M, peptide final conc. 1 mM) at pH 4, 6 and 8, respectively. To these solutions, three aliquots of 7.1 μL of reactive reagents (ynamide **2e**, NEM and IAM, 5 equiv) were added, respectively. The reactions were incubated at 37 °C and were monitored by analytical RP-HPLC (XSelect C18 column (3.5 μm, 130 Å, 4.6 × 150 mm)) with a gradient of 5-70% B (0.1% TFA in MeCN) over 25 min at 220 nm) The yields were calculated according to integrated areas of HPLC peaks.

1) *The reaction of peptide **14** with ynamide **2e***

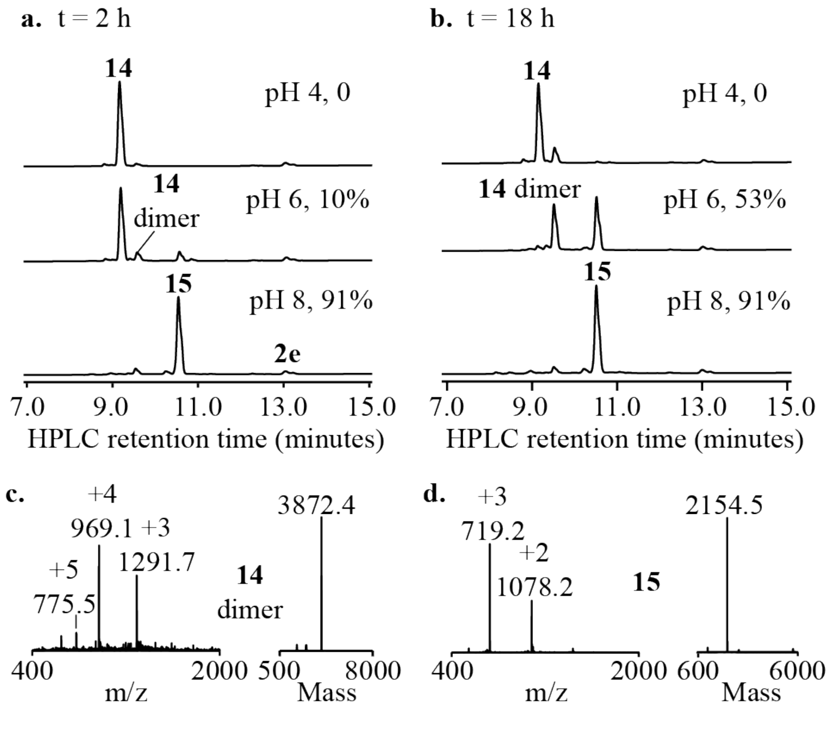

Figure S68. The modification reactions of model peptide **14** (1 mM) with ynamide (**2e**, 5 equiv) at pH 4, 6 and 8, respectively. **a**. The progress of the reactions after 2 h; **b**. The progress of reactions after 18 h. **c**. ESI-MS of peptide **14** dimer side-product (Found 3872.4 Da, Calcd. 3872.8 Da) **d**. ESI-MS of modified product **15** (Found 2154.5 Da, Calcd. 2154.9 Da).

2) *The reaction of peptide **14** with N-ethylmaleimide (NEM)*

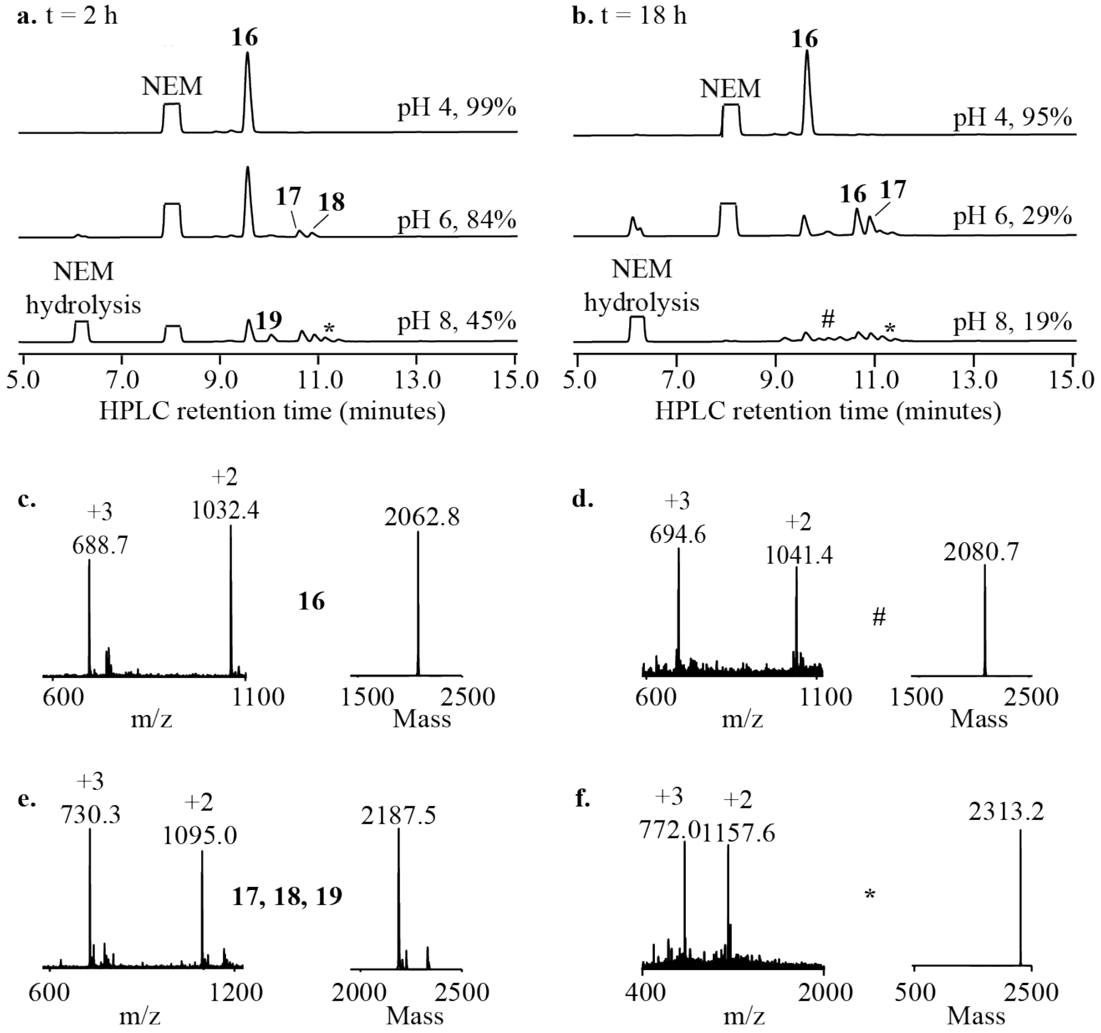

Figure S69. The modification reactions of model peptide **14** (1 mM) with NEM (5 equiv) at pH 4, 6 and 8, respectively. **a**. The progress of the reactions after 2 h; **b**. The progress of the reactions after 18 h. **16** is single modified product, **17**, **18** and **19** are doubly modified products. The product of peptide **15** with hydrolyzed NEM was found from peak # and triple modified product was found from peak *. **c.** ESI-MS of single NEM modified product, **16** (Found 2062.8 Da, Calcd. 2062.9 Da); d. ESI-MS of the modified product of peptide **14** with hydrolyzed NEM (Found 2080.7 Da, Calcd. 2080.9 Da); **e**. ESI-MS of doubly NEM modified products **17**, **18** and **19** (Found 2187.5 Da, Calcd. 2188.0 Da); f. ESI-MS of triple NEM modified product (Found 2313.2 Da, Calcd. 2313.0 Da).

3) *The reaction of peptide **14** with iodoacetamide (IAM)*

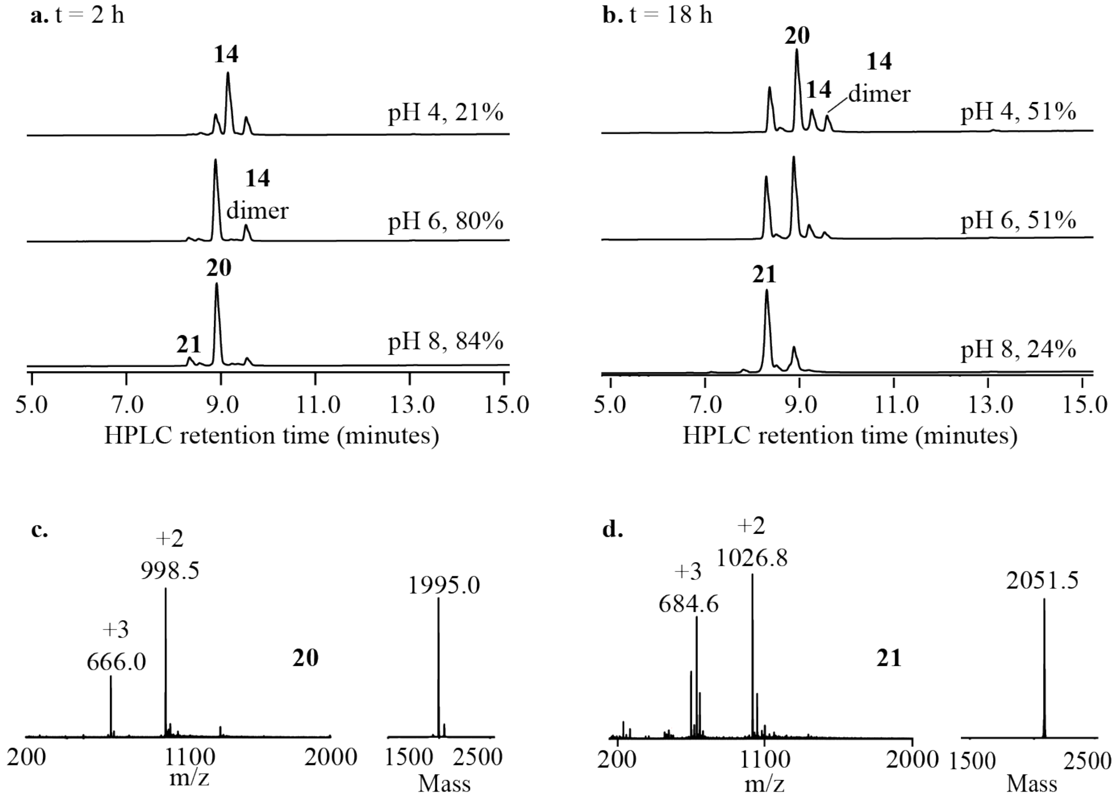

Figure S70. The modification of model peptide **14** (1 mM) with IAM (5 equiv), at pH 4, 6 and 8, respectively. **a**. The progress of the reactions after 2 h; **b**. The progress of the reactions after 18 h. **20** is singly IAM modified product, **21** is doubly IAM modified product. **c**. ESI-MS of singly IAM modified product, **20** (Found 1995.0 Da, Calcd. 1995.3 Da); d. ESI-MS of doubly IAM modified product, **21** (Found 2051.5 Da, Calcd. 2050.9 Da).

These results show the excellent chemoselectivity of peptide **14** modification with ynamide **2e** under the above reaction conditions, even after 18 h (Figure S68); whereas the other two classical and extensively used reagents, NEM and IAM, presented worse chemoselectivity toward Cys (Figure S69 and S70). Considerable amount of double and triple modification of peptide **14** with NEM and IAM were found under the same conditions (Figure S69 and S70), and only few singly modified products with NEM or IAM were obtained after 18 h.

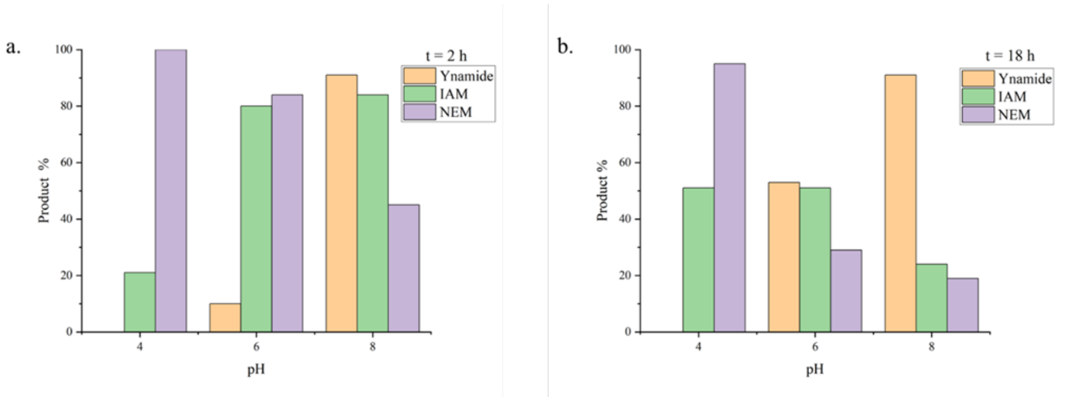

Figure S71. Modification of peptide **14** with ynamide **2e**, NEM and IAM. The graphs show only the percentage of singly modified peptide **14** with the different reagents; ynamide, NEM and IAM at different pH and after 2 h **(a)** or 18 h (**b**).

4) *Chemoselectivity evaluation of different reactive reagents with Cys-free peptide 22*

To further confirm the above conclusion (reactions with model peptide **14**), we designed negative control experiments. A cysteine-free model peptide **22** with the same sequence as peptide **14** except that the Cys was replaced with Ala, was utilized to examine the rectivity of the three reagnets (ynamide, NEM, and IAM) toward additional amino acids. The same reaction conditions were tested for peptide **22**.

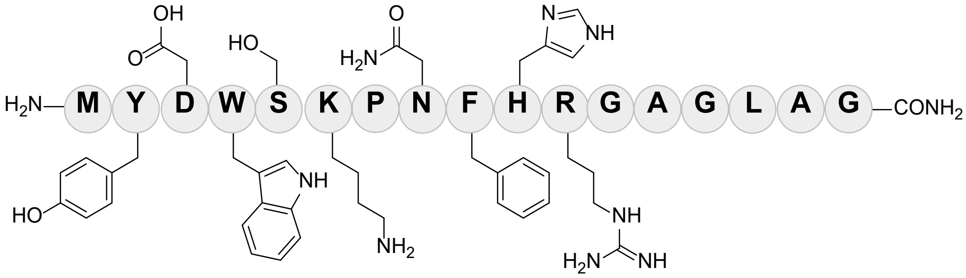

Figure S72. The sequence of peptide **22**

Peptide **22**, containing all amino acid side chains except Cys residue, was prepared by desulfurization reaction of peptide **14**. Peptide **14** (10 mg, 5.16 µmol, 1 mM) were dissolved in 5.16 mL PB (0.1 M, pH 5) TCEP (200 equiv), V-044 initiator (10 equiv) and 10 µL of tert-butyl mercaptane (17 equiv) were then added to the peptide solution. The reaction mixture was incubated at 37 °C for 2 h. Upon reaction completed (confirmed by HPLC and MS), peptide **22** was purified by semi-prep RP-HPLC (C18 column) with ~ 84% yield (8.3 mg, Figure S148). Peptide **22** (3.3 mg, 1.73 µmol, 16.3

mM) was dissolved in 106.3 μL water as peptide **22** stock solution. The stock solutions (105 mM in MeCN) of ynamide **2e**, NEM and IAM were prepared as described previously. Three aliquots of 9.2 μL of peptide **22** stock solution (0.15 μmol) were added to three aliquots of 133.7 μL PB (0.1 M, peptide final conc. 1 mM) at pH 4, 6 and 8, respectively. To these solutions, three aliquots of 7.1 μL of reactive reagents (ynamide **2e**, NEM and IAM, 5 equiv**)** were added, respectively. The reactions were incubated at 37 °C and were monitored by analytical RP-HPLC (XSelect C18 column (3.5 μm, 130 Å, 4.6 × 150 mm)) with a gradient of 5-70% B (0.1% TFA in ACN) over 25 min at 220 nm) The yields were calculated according to integrated areas of HPLC peaks.

5) *The reaction of peptide **22** with ynamide **2e***

No modification of peptide **22** with ynamide **2e** was found under the above conditions, even after 18 h.

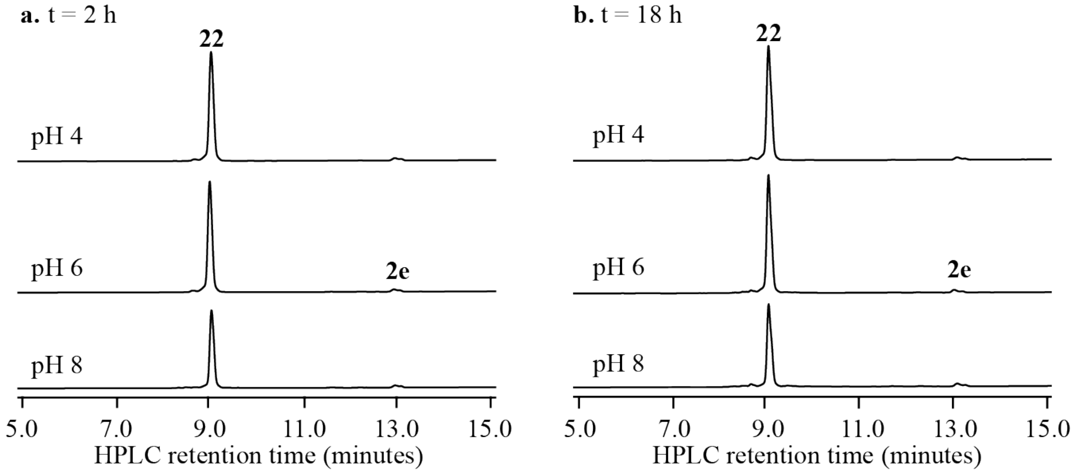

Figure S73. The reaction of peptide **22** and ynamide **2e**

6) *The reaction of peptide **22** with N-ethylmaleimide (NEM)*

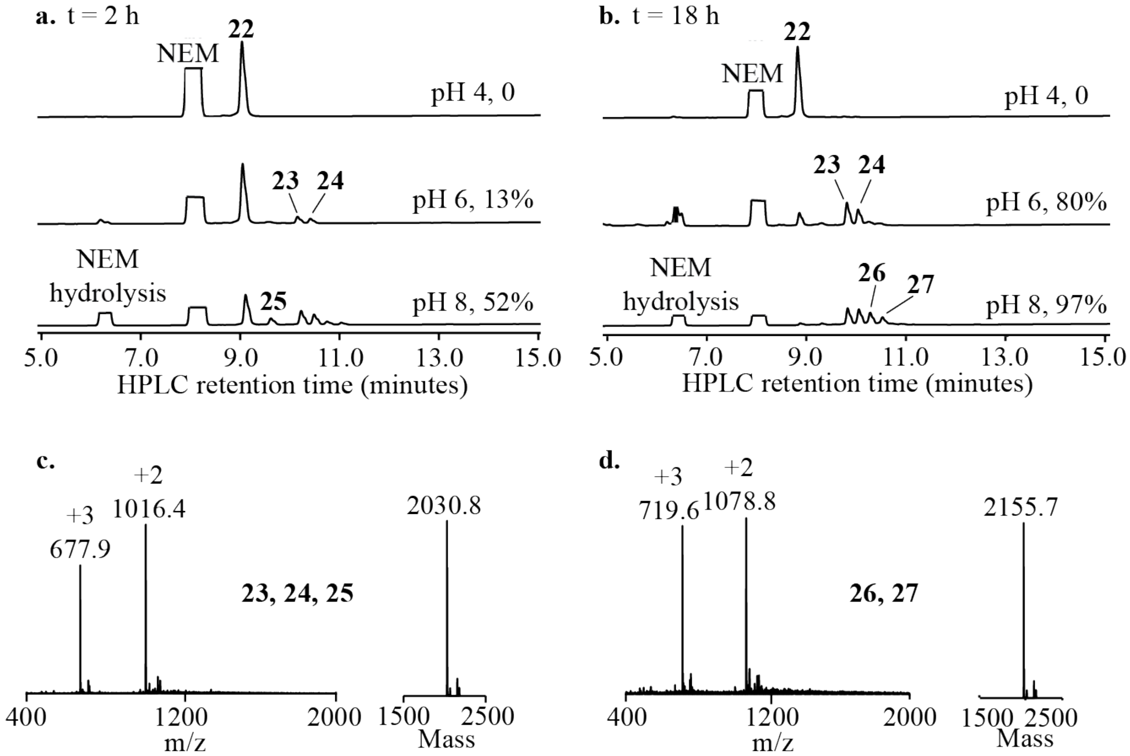

Figure S74. The modification reaction of model peptide **22** (1 mM) with NEM (5 equiv) at pH 4, 6 and 8, respectively. **a**. The progress of the reactions after 2 h; **b**. The progress of the reactions after 18 h. **23**, **24** and **25** are singly NEM modified products, **26** and **27** are doubly NEM modified products. **c**. ESI-MS of the singly NEM modified products **23**, **24** and **25** (Found 2030.8 Da, Calcd. 2031.0 Da); **d**. ESI-MS of doubly NEM modified products **26** and **27** (Found 2155.7 Da, Calcd. 2156.0 Da).

7) *The reaction of peptide **22** with iodoacetamide (IAM)*

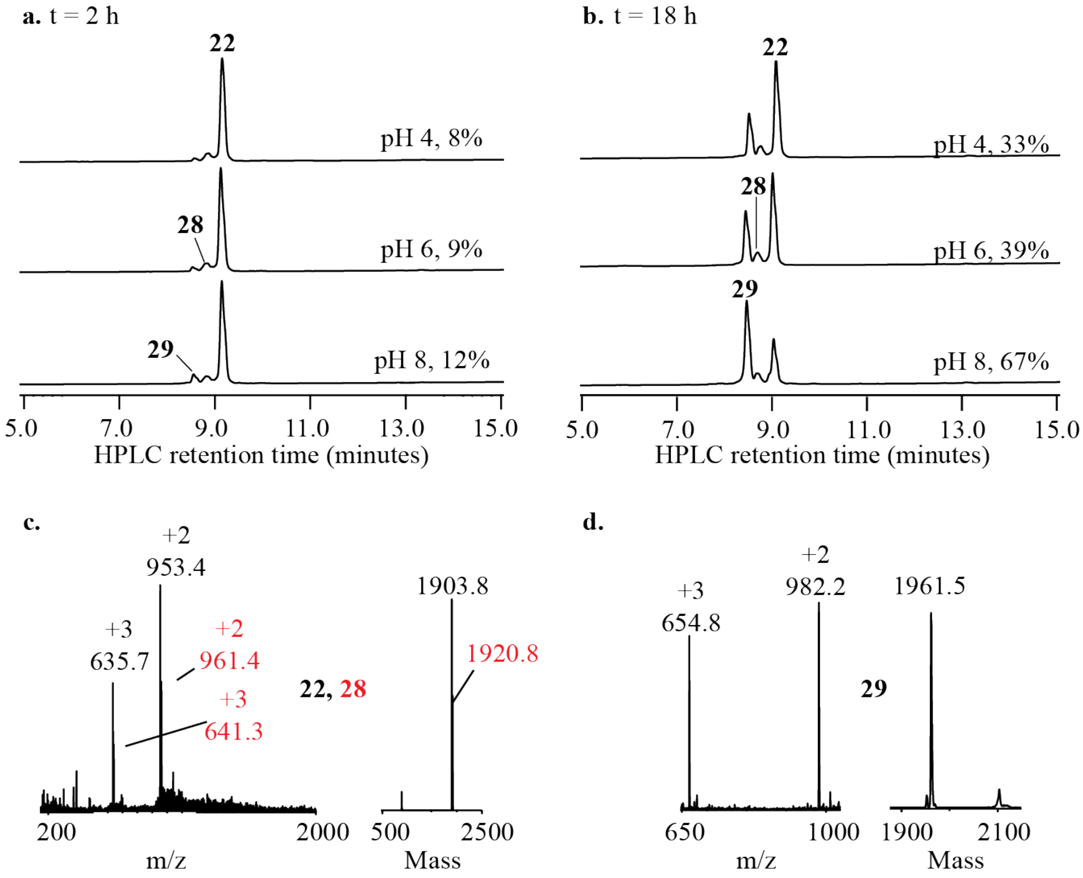

Figure S75. The modification reaction of model peptide **22** (1 mM) with IAM (5 equiv) at pH 4, 6 and 8, respectively. **a**. The progress of the reactions after 2 h; **b**. The progress of the reactions after 18 h. **28** is the oxidized peptide **22**, **29** is single NEM modified product. **c**. ESI-MS of peptide **22** with oxidation of methionine residue **28** (Found 1920.8 Da, Calcd. 1920.9 Da); **d**. ESI-MS of the single IAM modified product **29** (Found 1961.5 Da, Calcd. 1961.9 Da).

Based on the above results, peptide **22** did not react with ynamide **2e** (Figure S73), which supports our conclusions for the high chemoselectivity of ynamide toward Cys. However, large amounts of modified products, including single and double modification, were found from the reaction of peptide **22** with NEM and IAM (Figure S74 and S75), which showed NEM and IAM could also be reactive toward other amino acids except Cys.

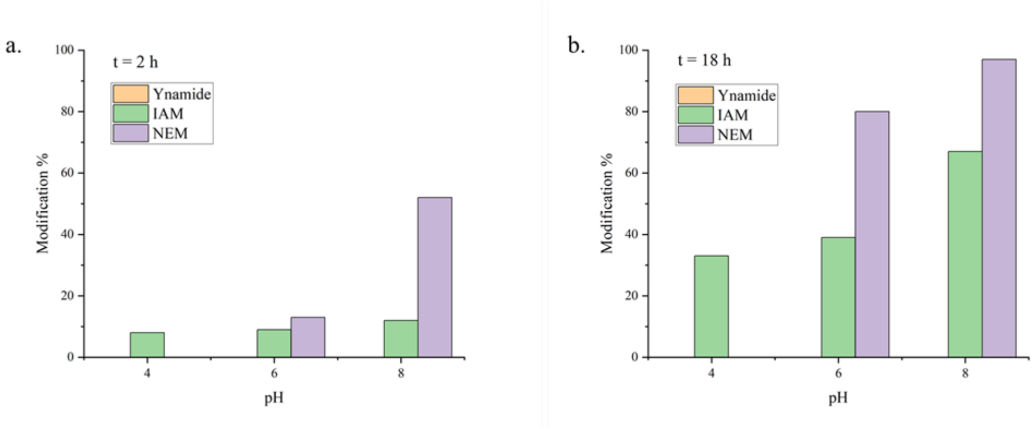

Figure S76. Modification of Cys-free model peptide **22** with ynamide **2e**, NEM and IAM. The graphs show the percentage of modified peptide **22** (including single and double) with the different reagents; ynamide, NEM and IAM at different pH after 2 h (**a**) and 18 h (**b**).

10. Radical Experiments

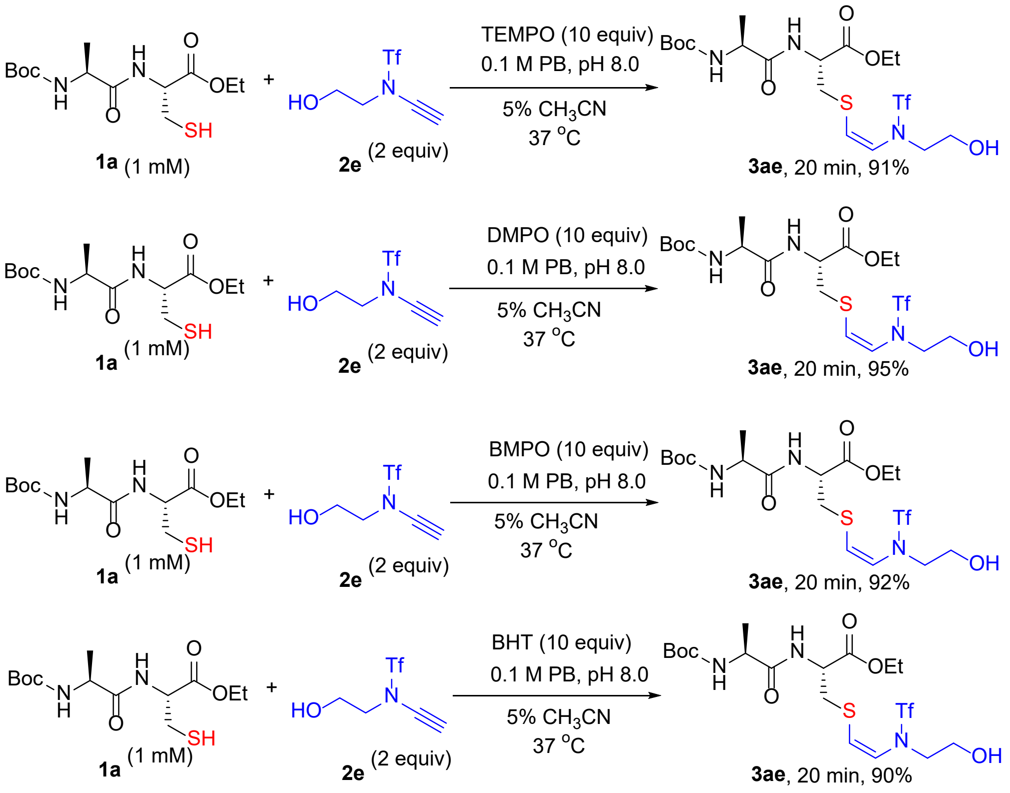

Scheme S15. The control experiment with different radical inhibitor

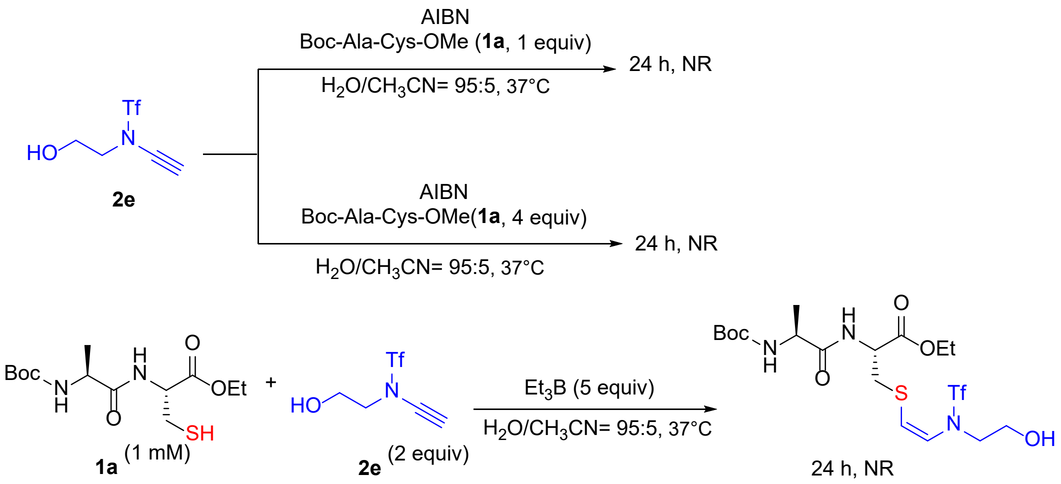

Scheme S16. The control experiment with different radical initiator between **1a** and **2e**

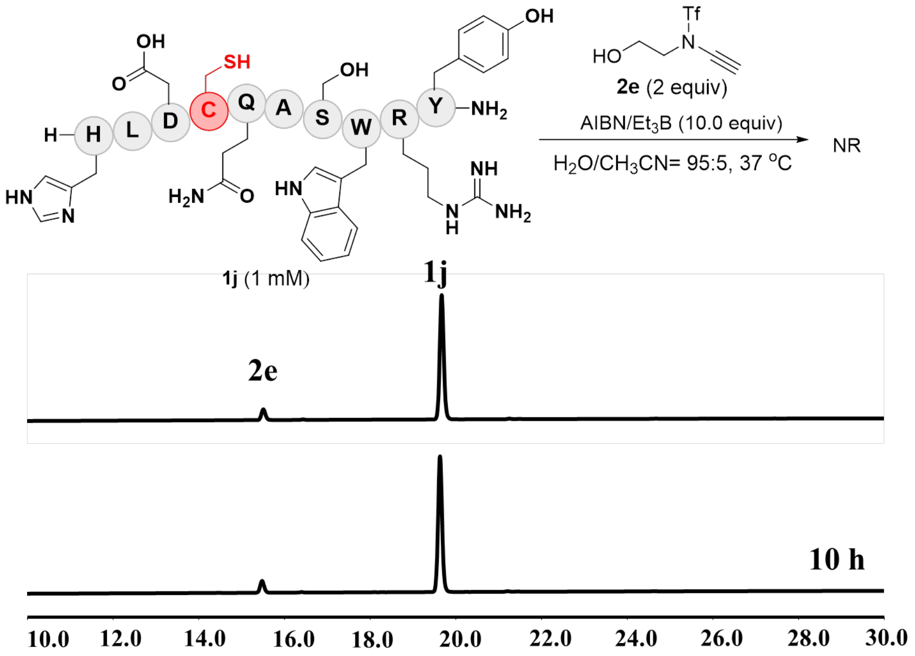

Figure S77. The control experiment with different radical initiator between **1j** and **2e**

11. Generation of a trifunctional protein by ynamide-based bioconjugation with fluorescein or PEG polymers.

(1) The complete sequences of genes and proteins used and created:

The amino acid sequence of affibody-mCherry-Cys (**30**) containing a single Cys at the C-terminus:

MLQVDNKFNKEMWAAWEEIRNLPNLNGWQMTAFIASLVDDPSQSANLLAEA
KKLNDAQAPKVDGGGGSGGSSGVSKGEDDNMAIIKEFMRFKVHMEGSVNG
HEFEIEGEGEGRPYEGTQTAKLKVTKGGPLPFAWDILSPQFMYGSKAYVKHPA
DIPDYLKLSFPEGFKWERVMNFEDGGVVTVTQDSSLQDGEFIYKVKLRGTNF
PSDGPVMQKKTMGWEASSERMYPEDGALKGEIKQRLKLKDGGHYDAEVKT
TYKAKKPVQLPGAYNVNIKLDITSHNEDYTIVEQYERAEGRHSTGGMDELYK
GSNCLHHHHHHWSHPQFEK*

(2) *The amino acids of mCherry-SpyCatcher003 (**33**):*

MVSKGEDDNMAIIKEFMRFKVHMEGSVNGHEFEIEGEGEGRPYEGTQTAKLK
VTKGGPLPFAWDILSPQFMYGSKAYVKHPADIPDYLKLSFPEGFKWERVMNFE
DGGVVTVTQDSSLQDGEFIYKVKLRGTNFPSDGPVMQKKTMGWEASSERMY
PEDGALKGEIKQRLKLKDGGHYDAEVKTTYKAKKPVQLPGAYNVNIKLDITS
HNEDYTIVEQYERAEGRHSTGGMDELYKGGSGGSVTTLSGLSGEQGPSGDMT
TEEDSATHIKFSKRDEDGRELAGATMELRDSSGKTISTWISDGHVKDFYLYPG
KYTFVETAAPDGYEVATPIEFTVNEDGQVTVDGEATEGDAHTLPETGGHHHH
HH*

*(3) The gene sequence of affibody-mCherry-Cys (**30**):*

AtgCTGCAGGTAGATAACAAATTCAACAAAGAAATGTGGGCGGCGTGGGAA
GAAATTCGCAACCTGCCGAACCTGAACGGCTGGCAGATGACCGCGTTTATT
GCGAGCCTGGTGGATGACCCAAGCCAAAGCGCTAACTTGCTAGCAGAAGC
TAAAAAGCTAAATGATGCTCAGGCGCCGAAAGTAGACGGAGGTGGAGGAT
CTGGTGGAAGCAGCGGCGTGAGCAAGGGCGAGGACGACAACATGGCCAT
CATCAAGGAGTTCATGCGCTTCAAGGTGCACATGGAGGGCTCCGTGAACG
GCCACGAGTTCGAGATCGAGGGCGAGGGCGAGGGCCGCCCCTACGAGGG
CACCCAGACCGCCAAGCTGAAGGTGACCAAGGGCGGCCCCCTGCCCTTCG
CCTGGGACATCCTGTCCCCTCAGTTCATGTACGGCTCCAAGGCCTACGTGA
AGCACCCCGCCGACATCCCCGACTACTTGAAGCTGTCCTTCCCCGAGGGCT
TCAAGTGGGAGCGCGTGATGAACTTCGAGGACGGCGGCGTGGTGACCGTG

ACCCAGGACTCCTCCCTGCAGGACGGCGAGTTCATCTACAAGGTGAAGCT
GCGCGGCACCAACTTCCCCTCCGACGGCCCCGTAATGCAGAAGAAGACCA
TGGGCTGGGAGGCCTCCTCCGAGCGGATGTACCCCGAGGACGGCGCCCTG
AAGGGCGAGATCAAGCAGAGGCTGAAGCTGAAGGACGGCGGCCACTACG
ACGCCGAGGTCAAGACCACCTACAAGGCCAAGAAGCCCGTGCAGCTGCCC
GGCGCCTACAACGTCAACATCAAGCTGGACATCACCTCCCACAACGAGGA
CTACACCATCGTGGAACAGTACGAGCGCGCCGAGGGCCGCCACTCCACCG
GCGGCATGGACGAGCTGTACAAGGGTTCTAACTGTCTGCATCACCACCACC
ACCACTGGTCTCACCCGCAGTTCGAAAAATAA

*(4) The gene sequence of mCherry-SpyCatcher003 (**33**):*

ATGGTGAGCAAGGGCGAGGACGACAACATGGCCATCATCAAGGAGTTCAT
GCGCTTCAAGGTGCACATGGAGGGCTCCGTGAACGGCCACGAGTTCGAGA
TCGAGGGCGAGGGCGAGGGCCGCCCCTACGAGGGCACCCAGACCGCCAA
GCTGAAGGTGACCAAGGGCGGCCCCCTGCCCTTCGCCTGGGACATCCTGT
CCCCTCAGTTCATGTACGGCTCCAAGGCCTACGTGAAGCACCCCGCCGACA
TCCCCGACTACTTGAAGCTGTCCTTCCCCGAGGGCTTCAAGTGGGAGCGCG
TGATGAACTTCGAGGACGGCGGCGTGGTGACCGTGACCCAGGACTCCTCC
CTGCAGGACGGCGAGTTCATCTACAAGGTGAAGCTGCGCGGCACCAACTT
CCCCTCCGACGGCCCCGTAATGCAGAAGAAGACCATGGGCTGGGAGGCCT
CCTCCGAGCGGATGTACCCCGAGGACGGCGCCCTGAAGGGCGAGATCAAG
CAGAGGCTGAAGCTGAAGGACGGCGGCCACTACGACGCCGAGGTCAAGA
CCACCTACAAGGCCAAGAAGCCCGTGCAGCTGCCCGGCGCCTACAACGTC
AACATCAAGCTGGACATCACCTCCCACAACGAGGACTACACCATCGTGGA
ACAGTACGAGCGCGCCGAGGGCCGCCACTCCACCGGCGGCATGGACGAGC
TGTACAAGGGTGGTTCTGGTGGTTCTGTAACCACCTTATCAGGTTTATCAGG
TGAGCAAGGTCCGTCCGGTGATATGACAACTGAAGAAGATAGTGCTACCCA
TATTAAATTCTCAAAACGTGATGAGGACGGCCGTGAGTTAGCTGGTGCAAC
TATGGAGTTGCGTGATTCATCTGGTAAAACTATTAGTACATGGATTTCAGAT
GGACATGTGAAGGATTTCTACCTGTATCCAGGAAAATATACATTTGTCGAAA
CCGCAGCACCAGACGGTTATGAGGTAGCAACTCCAATTGAATTTACAGTTA
ATGAGGACGGTCAGGTTACTGTAGATGGTGAAGCAACTGAAGGTGACGCT
CATACTCTGCCGGAAACCGGTGGTCACCACCACCATCACCATTAA

(5) Protein expression and purification

Genes encoding the desired protein sequences either affibody-mCherry-Cys (**30**) or mCherry-SpyCatcher003 (**33**) were cloned into the pETDuet vector and the plasmids

were then transformed into *Escherichia coli* BL21 (DE3) competent cells using the standard 90 s heat shock protocol. The cells were incubated for 12 hours at 37 °C and 250 rpm in LB medium. This overnight cell culture was subsequently diluted at a ratio of 1:100 into 800 ml of LB media supplemented with ampicillin (100 mg/L) and incubated at 37 °C. When the OD reached 0.6, protein expression was induced by adding 1 mM IPTG to the culture for 6-8 hours at 37 °C. Subsequently, cells were harvested and lysed by sonication in a lysis buffer containing 50 mM sodium phosphate and 500 mM NaCl (pH 8.0). After centrifugation, the supernatant was loaded onto a column of Ni-NTA beads and incubated at 4 °C for 1 hour. The beads were washed three times with lysis buffer, and the protein was subsequently eluted with lysis buffer containing 250 mM imidazole. The purified protein was transferred into a 50 mL Amicon Ultra-4 Centrifugal Filter with a 10 kDa cutoff for buffer exchange into phosphate buffer (pH 7.2) and finally stored in a freezer at -20 °C. The purity of the protein was analyzed by SDS-PAGE and analytical HPLC using a C4 column.

*(6) Conjugation of fluoresceine (**31**) and PEG molecules (**34-37**) to affibody-mCherry-Cys (**30**)*

All ynamide-functionalized labeling reagents were first dissolved in DMSO at a concentration of 100-200 mM and stored in a freezer at -20 °C. Whenever needed, the stock solution was then diluted into the PB buffer to the expected concentrations. A typical ynamide-mediated bioconjugation reaction was performed as follows: The protein affibody-mCherry-Cys (**30**, 100 μM, 1 equiv) in PB buffer pH 8.0 (20 μL-1 mL) was treated with TCEP (1 mM, 10 equiv) and incubated for 1 h at room temperature to reduce any potential disulfide bonds into free thiols. Subsequently, ynamide labeling reagents (**31**, **34-37**, 1 mM, 10 equiv) were added. The reaction was maintained at room temperature or 37 °C for 12-18 h, followed by SDS-PAGE analysis to monitor the reaction process. The conjugated product would be purified when necessary through simple buffer exchange using an Amicon Ultra-4 Centrifugal Filter or a semi-prep HPLC column.

(7) Tissue culture

Cells were maintained in 10% FBS in DMEM (high glucose) at 37 °C in an incubator under 5% CO_2. For passaging, cells were first washed three times with trypsin-EDTA (0.25%) to detach the cells from the tissue culture plates. Then, a 3-fold volume of complete DMEM medium was added to neutralize trypsin activity. Cells were grown until 40-60% confluency for the cell labeling assay.

(8) Cell staining and imaging

A549 and SW620 cells cultured in 24-well plates were washed three times with PBS. paraformaldehyde (4%, w/v in PBS) was then added to each well for 15 min to fix the cells. Then, the cells were washed with PBS three times to remove residual paraformaldehyde. To permeabilize the cells, Triton X-100 (0.1%, w/v in PBS) was added to the wells for 5 min. Then, PBS was used to wash the cells another three times before staining. To stain the cells, protein affibody-mCherry-Cys-FAM **32** were diluted in PBS to a concentration of 1 μM and 500 nM, respectively. Then, the solution was added to each well for 40 min. Next, the cells were washed with PBS three times and subjected to imaging analysis using confocal microscopy. The “Red” channel (filter cube: 550 nm) was used to obtain the mCherry fluorescence, while the “Green” channel (Filter Cube: 488 nm) was used for fluorescein.

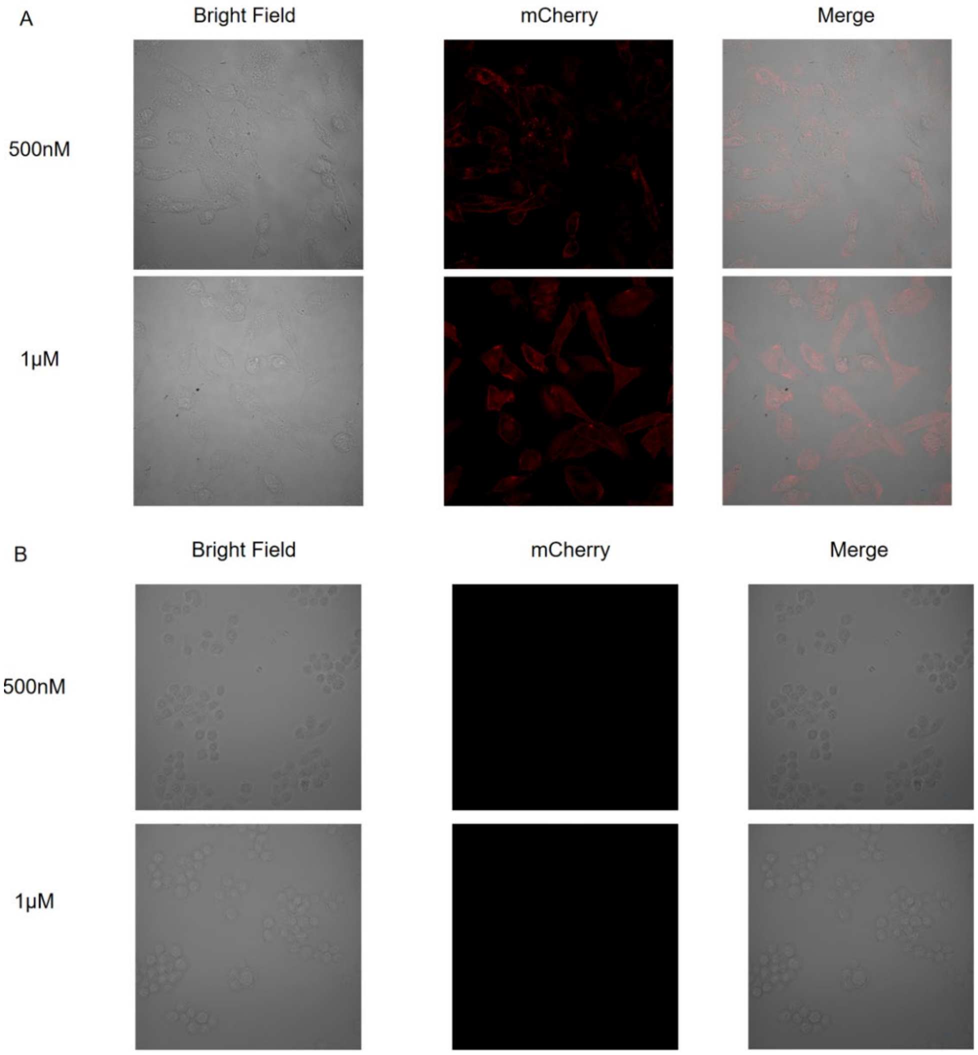

Figure S78. Confocal microscopy images of A549 (A) and SW620 (B) stained, respectively, with 1 μM and 500 nM affibody-mCherry-Cys (**30**). This data shows that only EGFR-positive A549 cells were successfully labeled with an obvious red color

rim on the cells, while there were no signals detected in the EGFR-negative SW620 cells. This confirms that affibody-mCherry-Cys (**30**) has the expected dual functions: selective targeting of EGFR-positive cells and red fluorescence for detection and visualization under fluorescent microscopy.

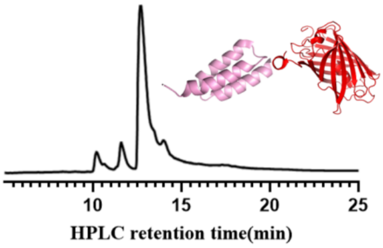

Figure S79. HPLC profile of the reduced affibody-mCherry-Cys by TCEP for 30 mins using C4 column.

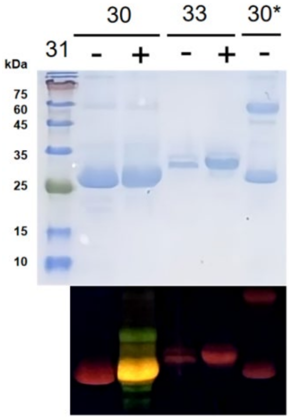

Figure S80. Thiol-specific fluorescent labeling of affibody-mCherry-Cys (**30**). The commassie blue staining and fluorescent imaging of affibody-mCherry-Cys protein and the mCherry-SpyCatcher003 that were labeled with ynamide-FAM reagent (**31)**. The fluorescent image shows red and yellow colors when excited by blue light and captured by a regular camera. The yellow color band indicates the desired product labeled with fluorescein, which is attributed to the colocalization of red and green fluorescence emitted by the mCherry and fluorescein, respectively. The green band may be caused by the nonspecific labeling of unknown thiol-containing proteins in the samples. Asterisks **(30*)** represent the affibody-mCherry-Cys without TECP treatment, showing the occurrence of dimerized protein caused by disulfide formation.

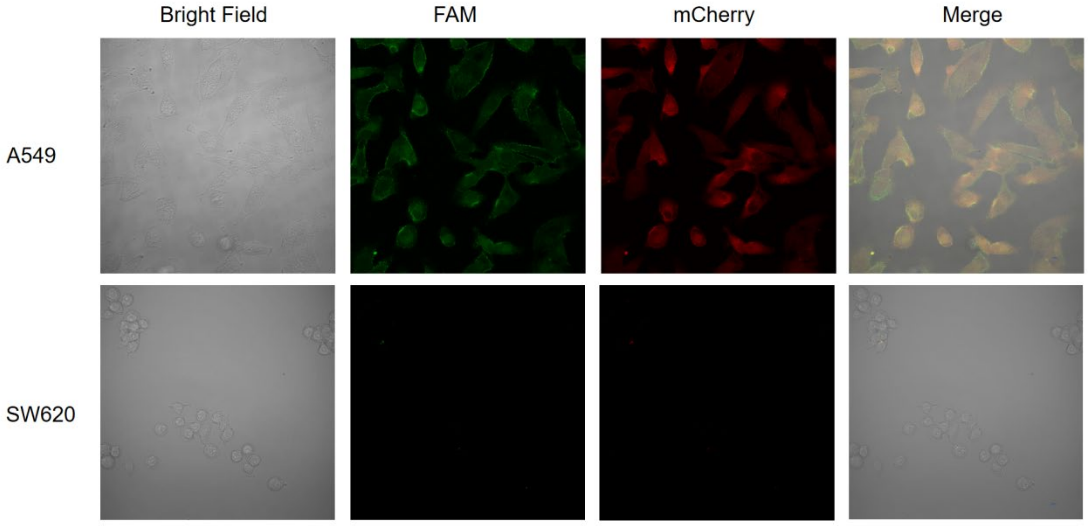

Figure S81. Confocal microscopy images of A549 (A) and SW620 (B) stained with 1 μM affibody-mCherry-FAM. This data shows that only EGFR-positive A549 cells exhibited green and red colors at the same time, whereas no signals were observed in the EGFR-negative SW620 cells.

12. Supplementary Figures

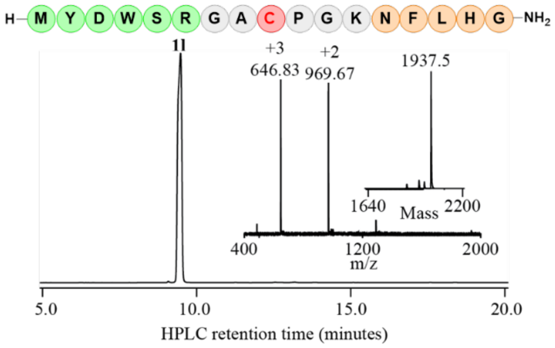

Figure S82. HPLC trace and ESI-MS of **1l** (Found 1937.5, Calcd. 1936.8)

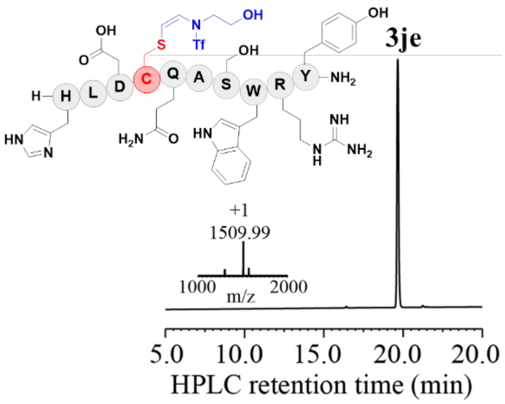

Figure S83. HPLC trace and ESI-MS of **3je** ($[M + H]^+$ Found 1509.99, Calcd. 1509.60)

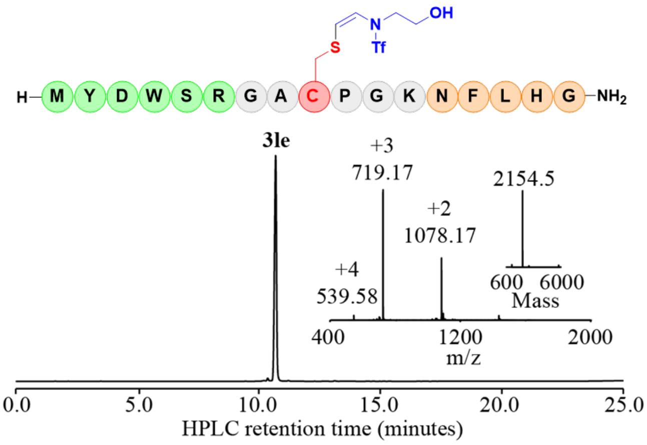

Figure S84. HPLC trace and ESI-MS of **3le** (Found 2154.5, Calcd. 2153.9)

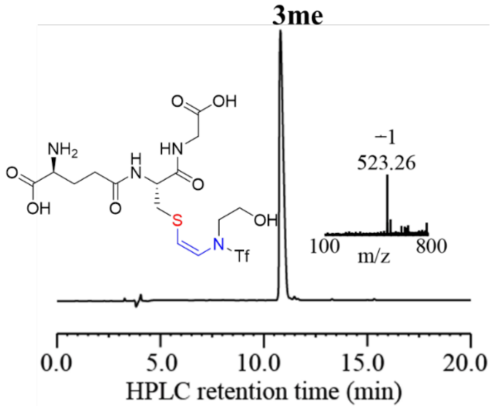

Figure S85. HPLC trace and ESI-MS of **3me** ([M - H]$^-$ Found 523.26, Calcd. 523.19)

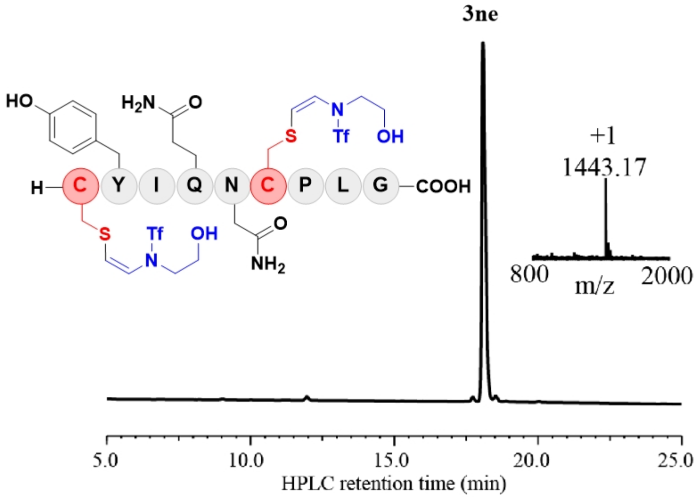

Figure S86. HPLC trace and ESI-MS of of **3ne** ([M + H]$^+$ Found 1443.17, Calcd. 1443.46)

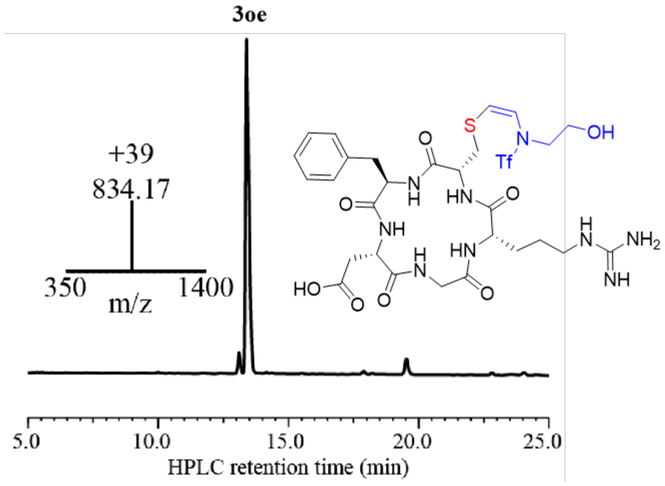

Figure S87. HPLC trace and ESI-MS of **3oe** ([M + K]$^+$ Found 834.17, Calcd. 834.19)

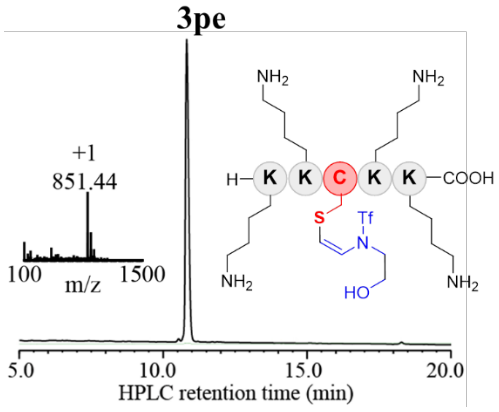

Figure S88. HPLC trace and ESI-MS of **3pe** ([M + H]$^+$ Found 851.44, Calcd. 851.41)

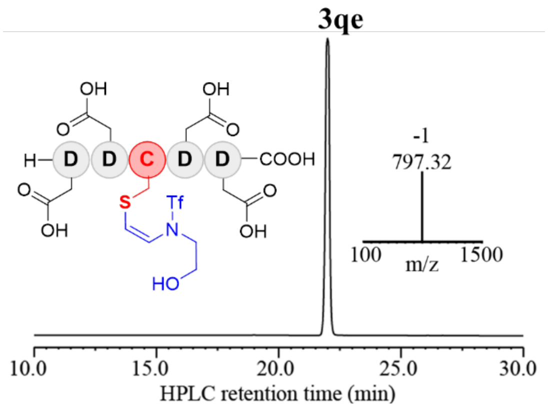

Figure S89. HPLC trace and ESI-MS of **3qe** ([M - H]$^-$ Found 797.32, Calcd. 797.12)

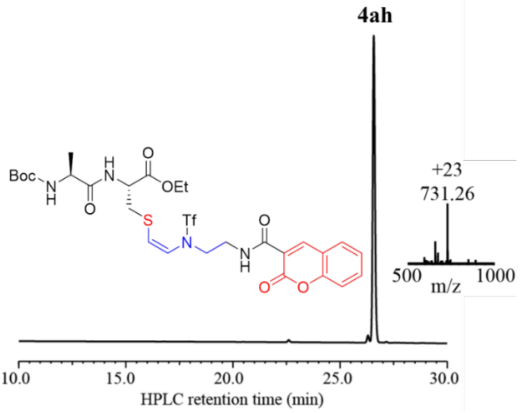

Figure S90. HPLC trace and ESI-MS of **4ah** ([M + Na]$^+$ Found 731.26, Calcd. 731.16)

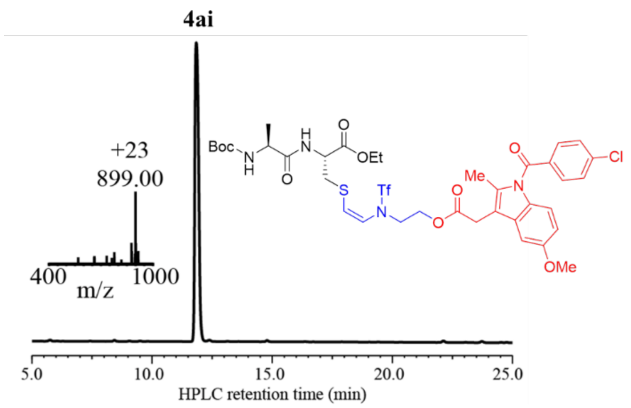

Figure S91. HPLC trace and ESI-MS of **4ai** ($[M + Na]^+$ Found 899.00, Calcd. 899.20)

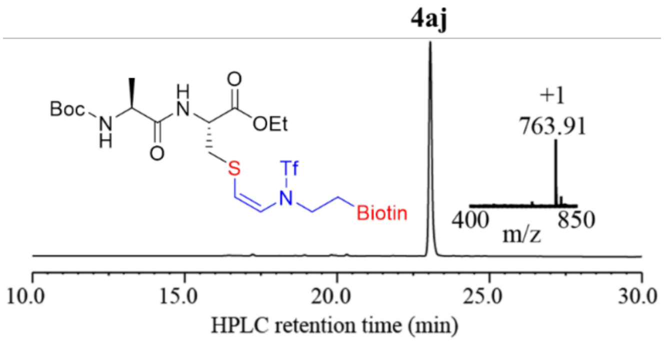

Figure S92. HPLC trace and ESI-MS of **4aj** ($[M + H]^+$ Found 763.91, Calcd. 764.23)

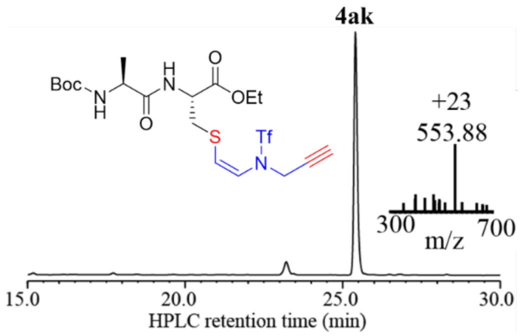

Figure S93. HPLC trace and ESI-MS of **4ak** ($[M + Na]^+$ Found 553.88, Calcd. 554.12)

4oh

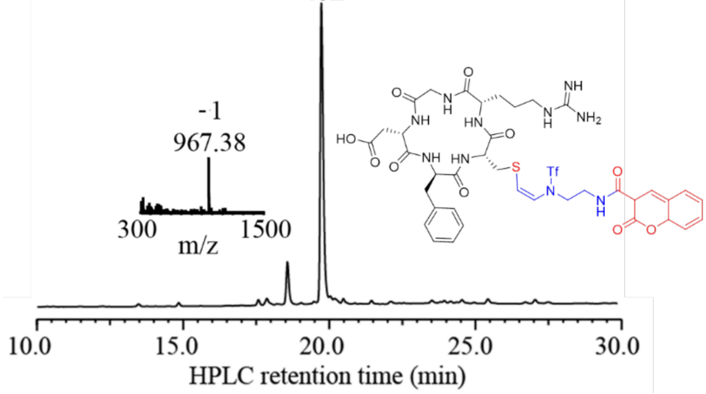

Figure S94. HPLC trace and ESI-MS of **4oh** ([M + H]$^+$ Found 967.38, Calcd. 967.27)

4oi

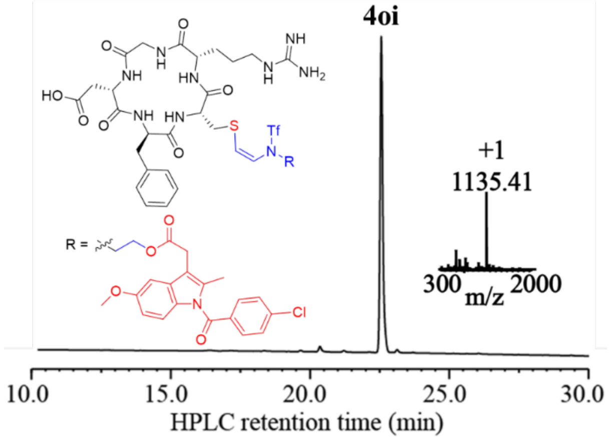

Figure S95. HPLC trace and ESI-MS of **4oi** ([M + H]$^+$ Found 1135.41, Calcd. 1135.30)

4oj

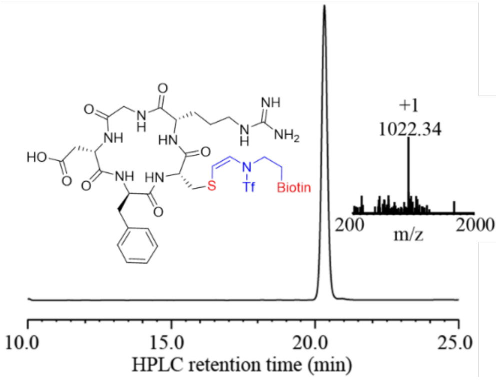

Figure S96. HPLC trace and ESI-MS of **4oj** ([M + H]$^+$ Found 1022.34, Calcd. 1022.31)

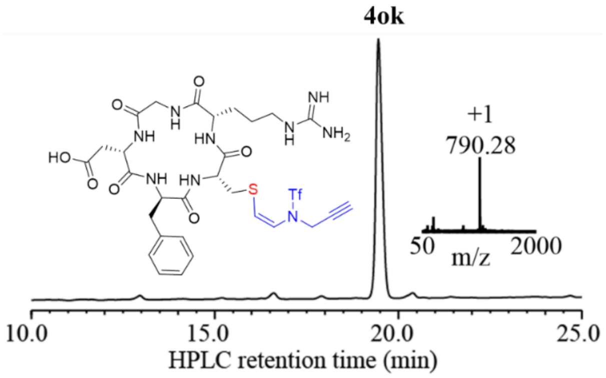

Figure S97. HPLC trace and ESI-MS of **4ok** ([M + H]$^+$ Found 790.28, Calcd. 790.23)

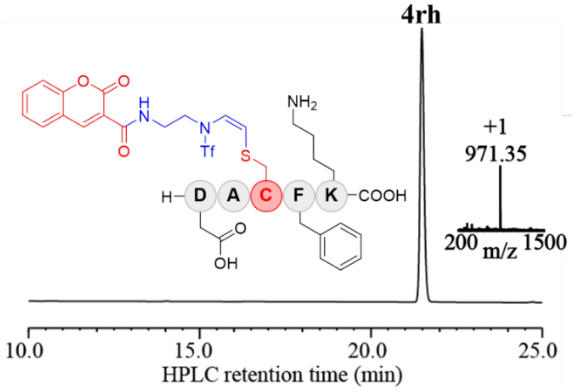

Figure S98. HPLC trace and ESI-MS of **4rh** ([M + H]$^+$ Found 971.35, Calcd. 971.29)

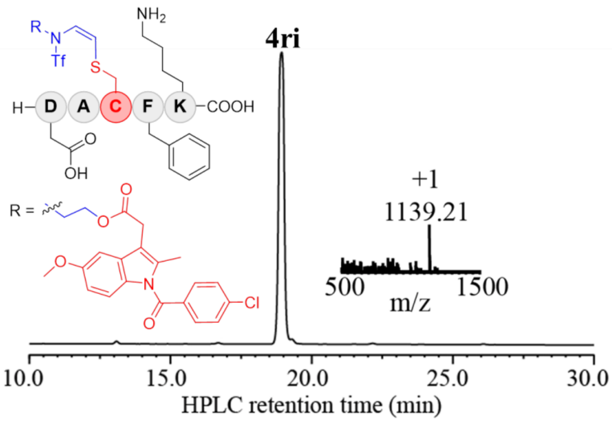

Figure S99. HPLC trace and ESI-MS of **4ri** ([M + H]$^+$ Found 1139.21, Calcd. 1139.32)

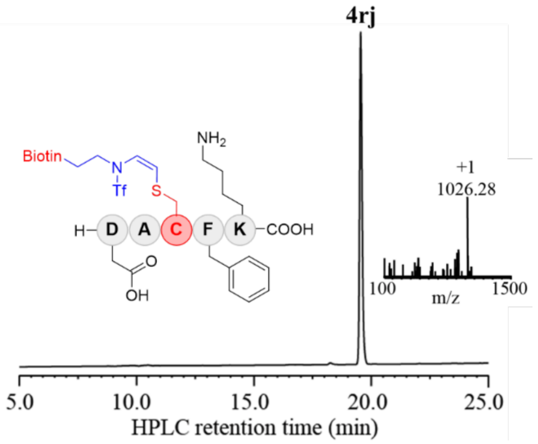

Figure S100. HPLC trace and ESI-MS of **4rj** ($[M + H]^+$ Found 1026.28, Calcd. 1026.33)

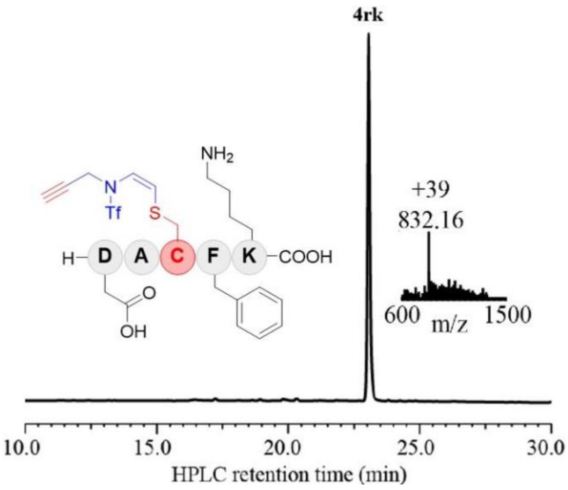

Figure S101. HPLC trace and ESI-MS of **4rk** ($[M + K]^+$ Found 832.16, Calcd. 832.20)

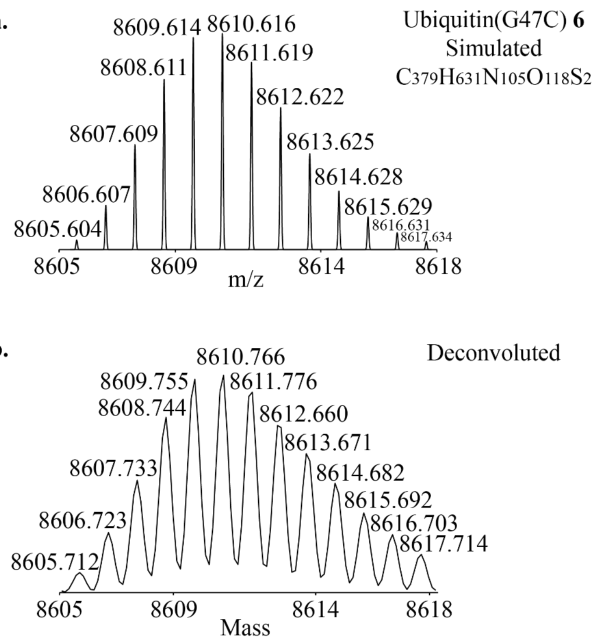

Figure S102. HR-MS analysis of the ubiquitin(G47C) (**6**) **a**. The simulated HR-MS of **6** with chemical formula $C_{379}H_{631}N_{105}O_{118}S_2$ is shown; **b**. The deconvoluted HR-MS of **6**

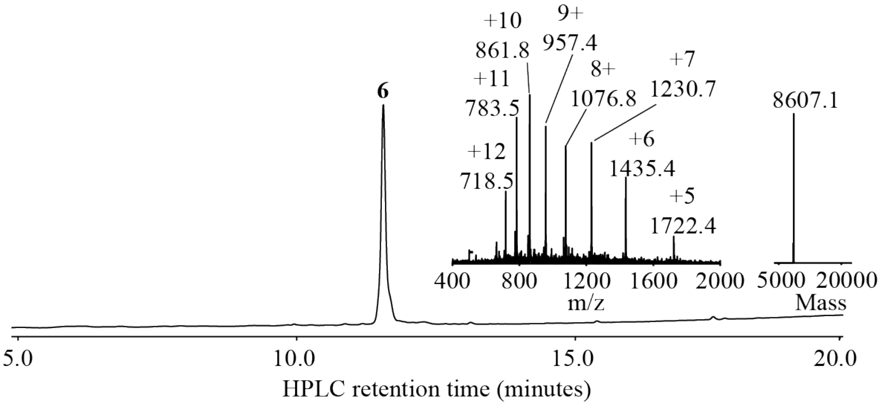

Figure S103. HPLC trace and ESI-MS of ubiquitin(G47C) (**6**)

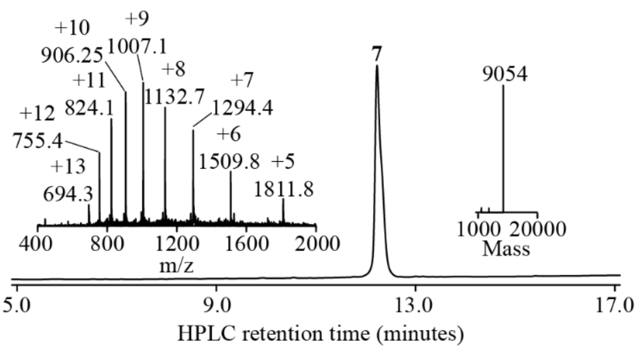

Figure S104. HPLC trace and ESI-MS of modified ubiquitin(G47C) (**7**)

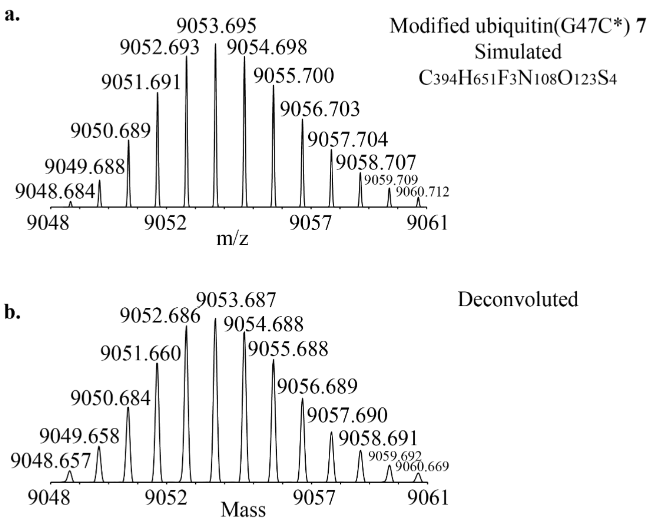

Figure S105. HR-MS analysis of the modified ubiquitin(G47C) (**7**) **a**. The simulated HR-MS of **7** with chemical formula $C_{394}H_{651}F_3N_{108}O_{123}S_4$ is shown; **b**. The deconvoluted HR-MS of **7**

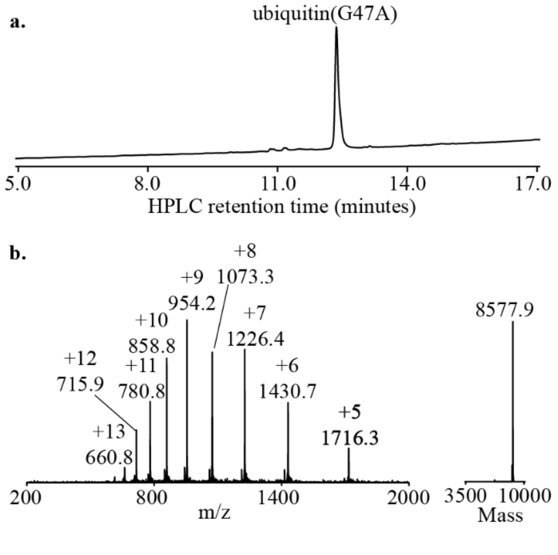

Figure S106. HPLC trace and ESI-MS of ubiquitin(G47A) (Found 8577.9, Calcd. 8578.9)

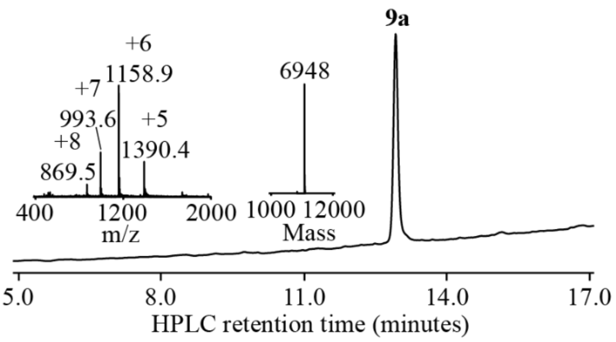

Figure S107. HPLC trace and ESI-MS of doubly modified BPTI (**9a**)

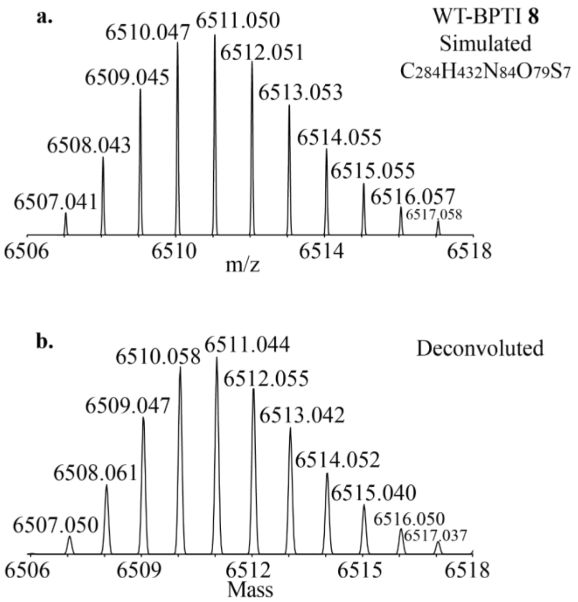

Figure S108. HR-MS analysis of the WT-BPTI (**8**) **a**. The simulated HR-MS of **8** with chemical formula $C_{284}H_{432}N_{84}O_{79}S_7$ is shown; **b**. The deconvoluted HR-MS of **8**

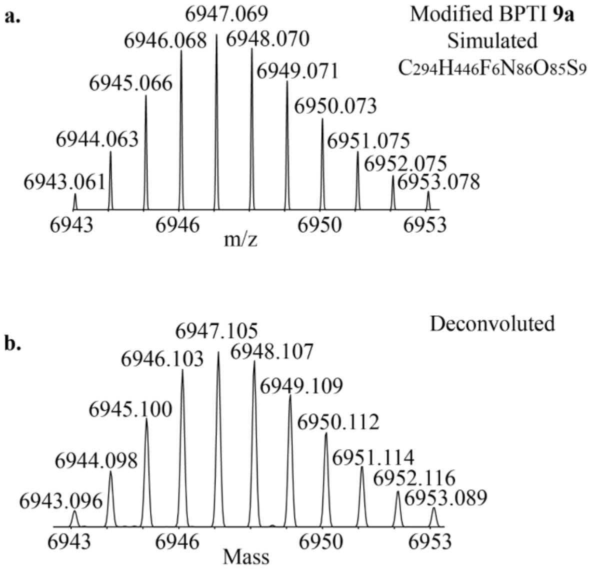

Figure S109. HR-MS analysis of the ubiquitin(G47C) (**9a**) **a**. The simulated HR-MS of **9a** with chemical formula $C_{294}H_{446}F_6N_{86}O_{85}S_9$ is shown; **b**. The deconvoluted HR-MS of **9a**

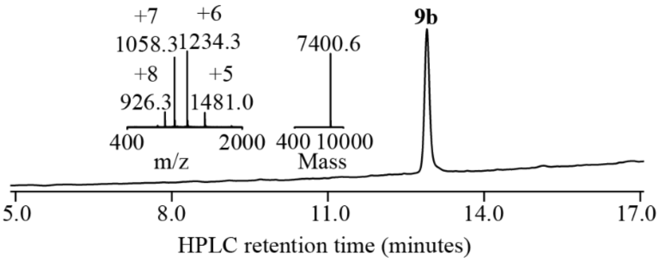

Figure S110. HPLC trace and ESI-MS of the doubly modified BPTI (**9b**) (Found 7400.6, Calcd. 7400.4)

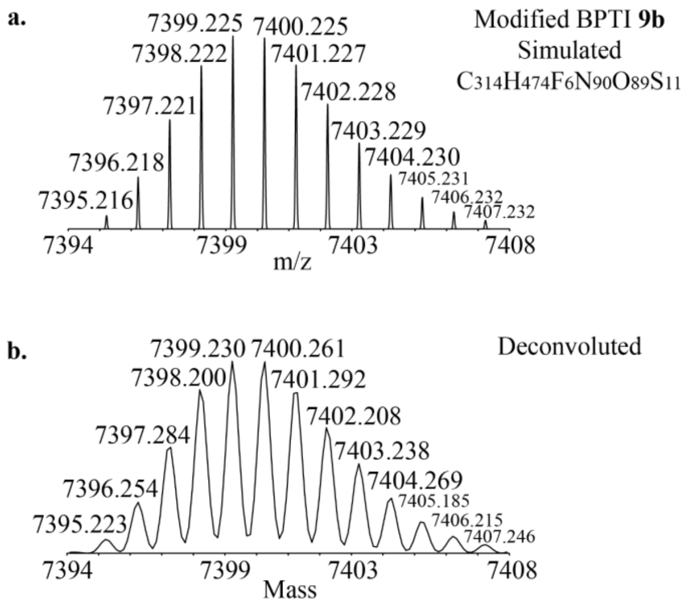

Figure S111. HR-MS analysis of the doubly modified BPTI (**9b**) **a**. The simulated HR-MS of **9b** with chemical formula $C_{314}H_{474}F_6N_{90}O_{89}S_{11}$ is shown; **b**. The deconvoluted HR-MS of **9b**

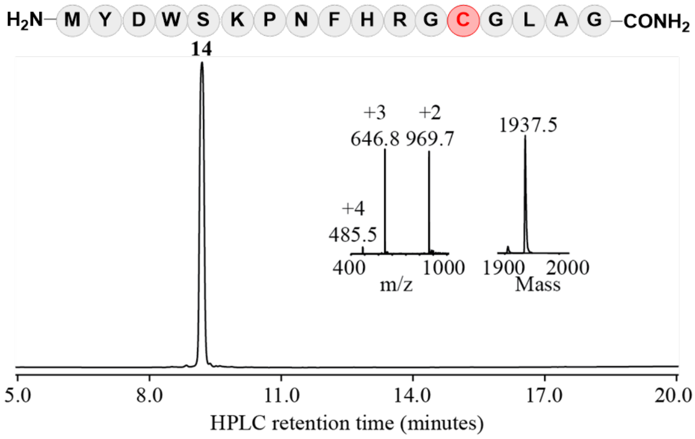

Figure S112. HPLC trace and ESI-MS of peptide **14** (Found 1937.5, Calcd. 1936.8)

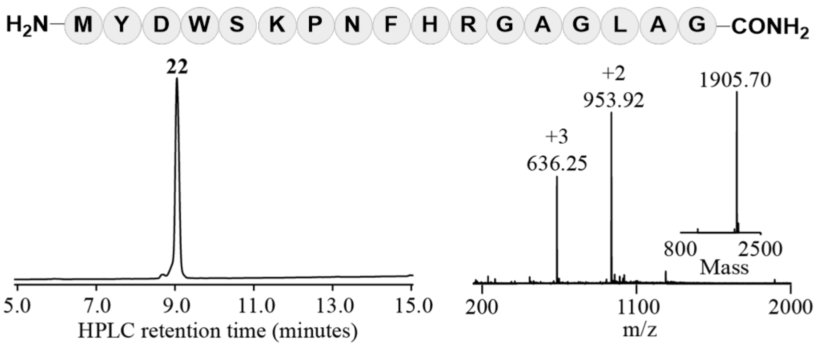

Figure S113. HPLC trace and ESI-MS of peptide **22** (Found 1905.7, Calcd. 1905.9)

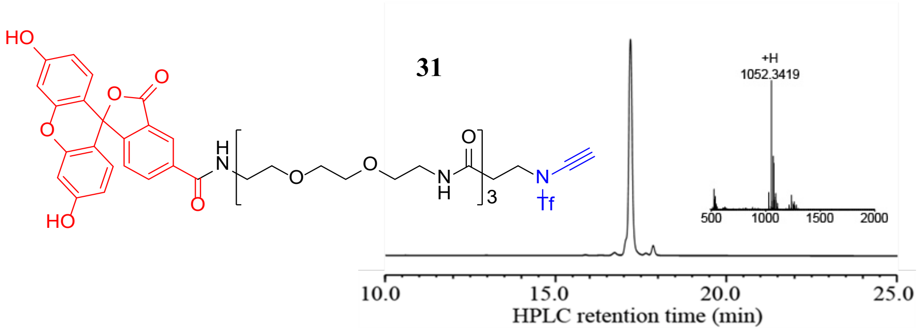

Figure S114. HPLC trace and ESI-MS of ynamide **31** (Calcd. 1052.3417; Found:1052.3412)

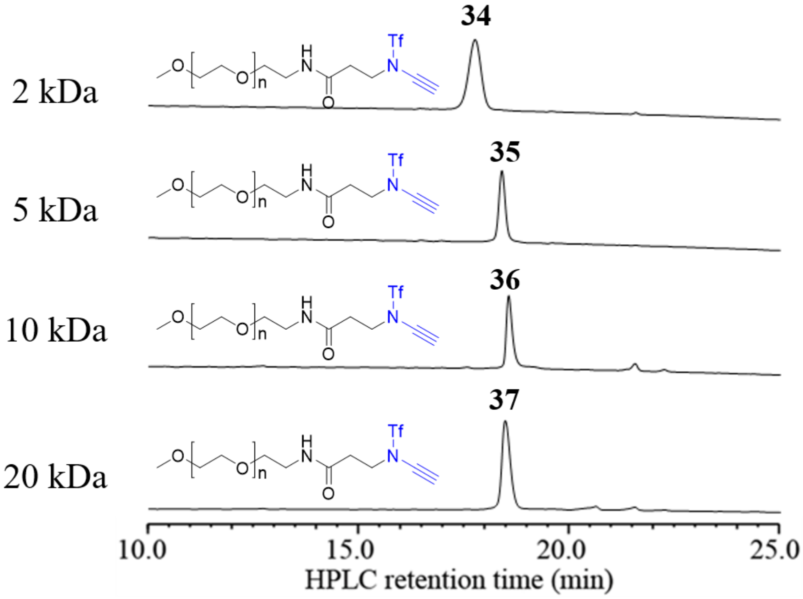

Figure S115. HPLC trace of ynamide **34-37**

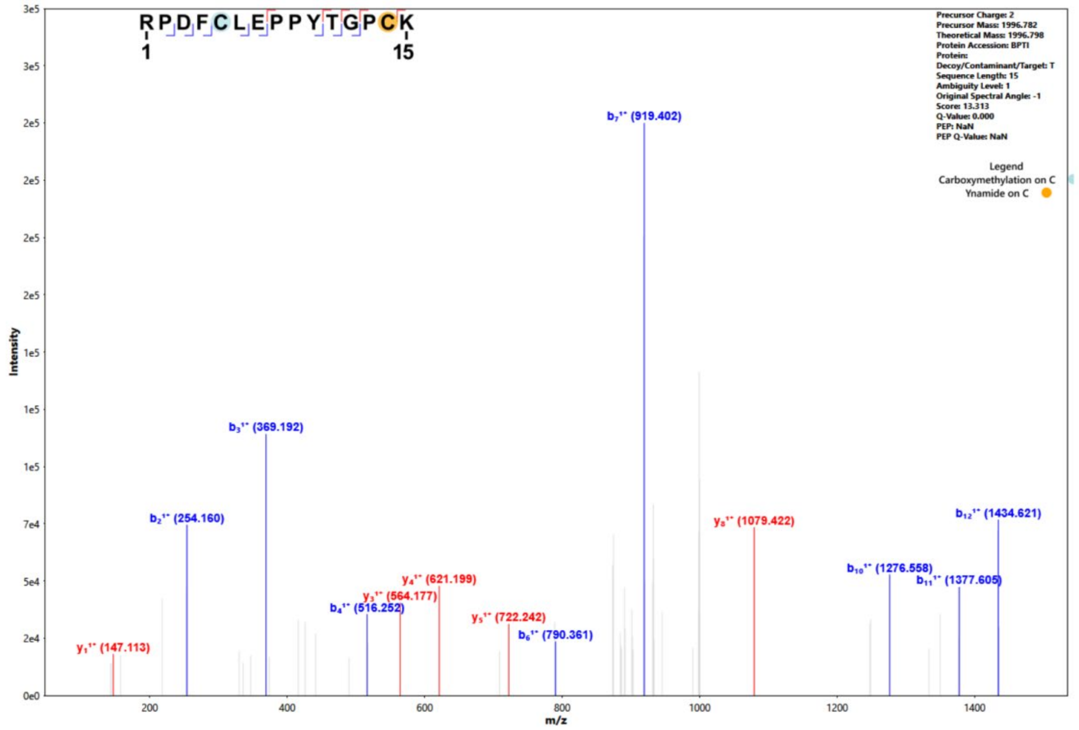

Figure S116. MS/MS for BPTI(1-15)(5CM-14ynamide), obs. mass 1996.782 Da.

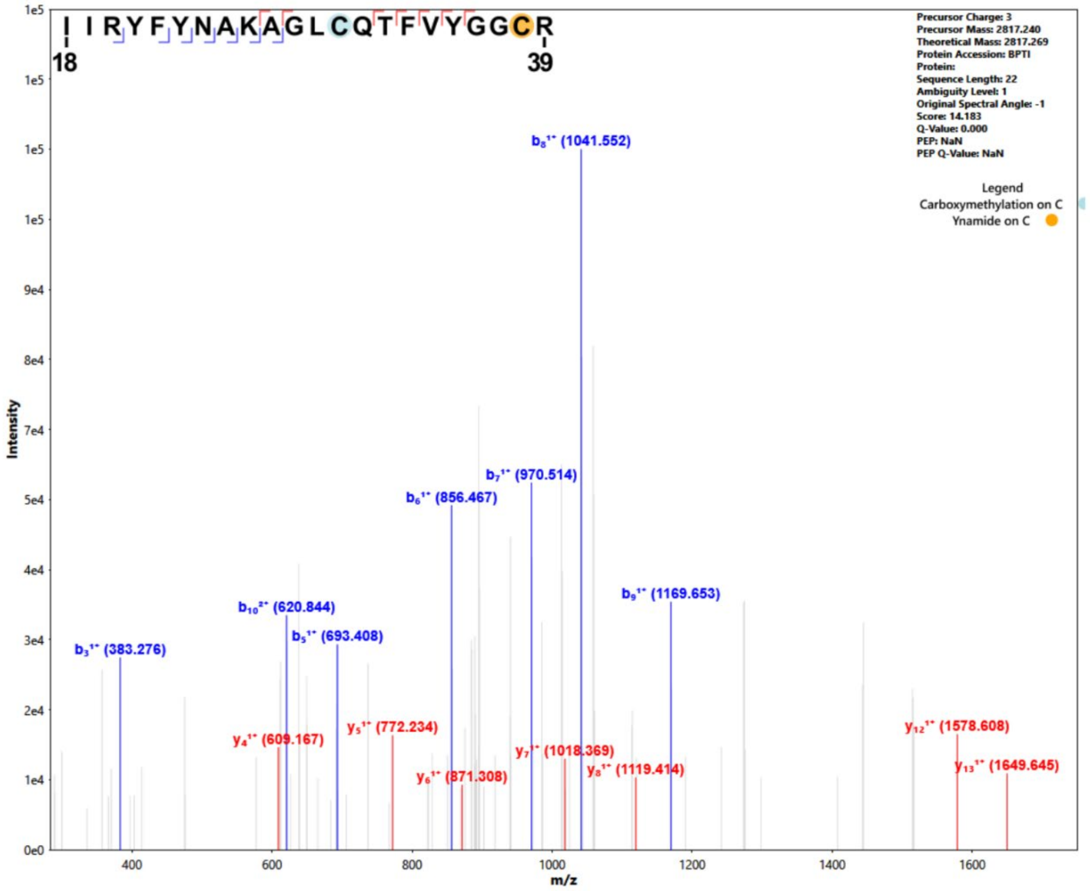

Figure S117. MS/MS for BPTI(18-39)(30CM-38ynamide), obs. mass 2817.240 Da.

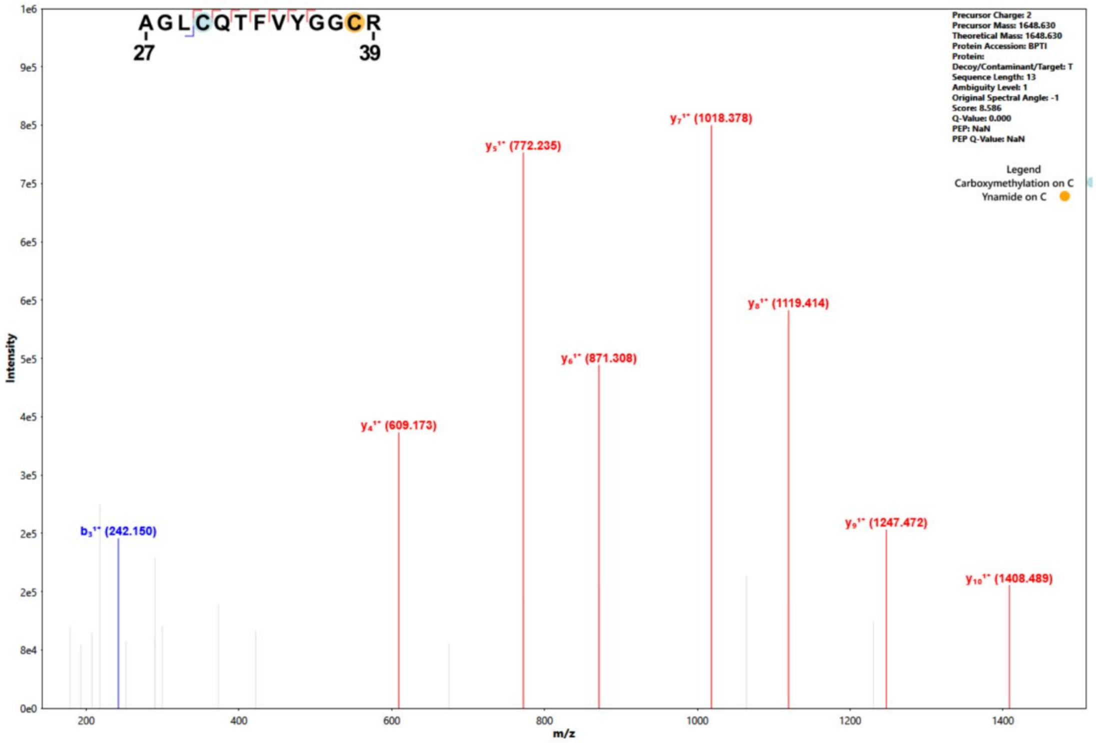

Figure S118. MS/MS for BPTI(27-39)(30CM-38ynamide), obs. mass 1648.630 Da.

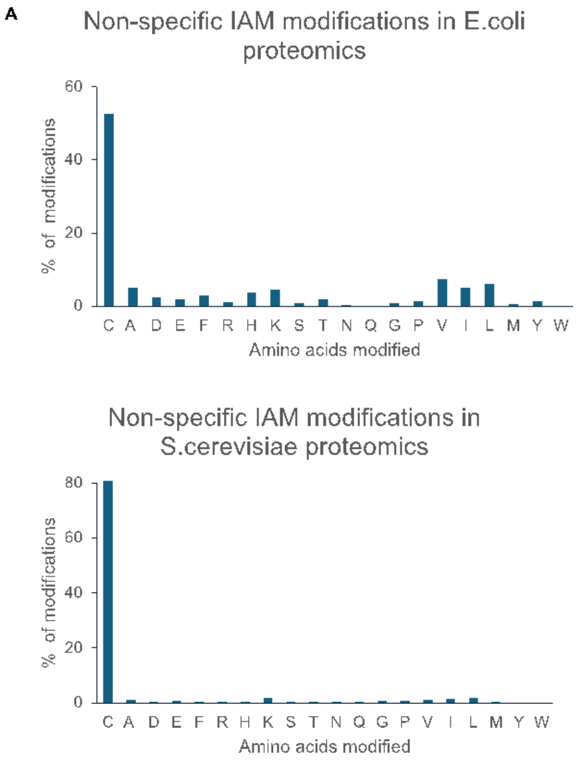

B

amino acid	*E.coli*	*S. cerevisiae*
C	52.55	86.03
A	5.11	1.04
D	2.31	0.54
E	1.98	0.87
F	2.97	0.39
R	0.99	0.26
H	3.79	0.47
K	4.45	2.09
S	0.82	0.59
T	1.81	0.61
N	0.33	0.36
Q	0.16	0.42
G	0.82	0.88
P	1.32	0.75
V	7.41	1.01
I	4.94	1.32
L	6.10	1.70
M	0.66	0.38
Y	1.32	0.20
W	0.16	0.09

Figure S119. Non-cysteine carbamidomethylation upon iodoacetamide treatment in two proteomics experiments of *E. coli* and *S. cerevisiae* proteomes.

13. Proteomic Implementation and Analysis of Ynamide in E. coli Lysate Samples

Methods:

Proteomic sample preparation:

Cellular extract was prepared as following: *E. coli* strain BL-21 (DE3) cells were grown in LB media until O.D. 0.7 and were then centrifuged at 5,000xg for 10 minutes at room temperature, resuspended in 500 uL of lysis buffer (20mM Hepes pH 7, 300 mM NaCl, 0.2 mg/mL Lysozyme, 5 mM $MgCl_2$, 10 µg/mL DNAse) and lysed using 3 freeze/thaw cycles.

After ultracentrifugation at 14,000xg at 4 °C for 20 min, samples were diluted at a ratio of 1:4 with urea buffer (8 M urea in 0.1 M Tris HCl, pH 8.5) and incubated with 10 mM DTT for 1 h at 37 °C, 350 rpm. Followed by the FASP protocol[10]: Samples were loaded onto 10 MW amicon filters (UFC5010BK), centrifuged for 10min at 12,000xg then alkylated by incubation with 50 mM IAM or Ynamide (**2e**) dissolved in urea buffer for 2 or 10 h, for IAM at room temp in the dark at 350 rpm, and for Ynamide at 37°C at 350 rpm. The samples were washed several times with the urea buffer and then twice with digestion buffer (10% ACN, 25 mM Tris HCl, pH 8.5), and centrifuged at 12,000xg for 10 min. Samples were subsequently transferred into new tubes and digested with 1 µL of trypsin (Promega) in 300 µL of digestion buffer, followed by overnight incubation at 37 °C, 350 rpm. Finally, samples were centrifuged at 12,000xg and desalted using C18 stage tips as described[11]. Samples were eluted from stage tips using 90 µL of elution buffer (80% ACN, 0.1% HPLC-grade TFA) and centrifuged at 350 g for 2 min. Peptides were then dried using a Speed Vac for 18min at 1,300 rpm at 35 °C after which they were dissolved in 10 µL of 0.1% Formic acid.

LC-MS/MS analysis:

0.3 µg of peptides were injected into the Nano Trap Column 100 mm i.d. x 2 cm, which is packed with Acclaim Pep-Map100 (C18, 5 mm, 100 Å, Thermo Scientific), from each sample, at 5uL/min for 8 min. The peptides were then separated on a C-18 reverse-phase column coupled to the Nano electrospray Easy-spray (PepMap, 75 mm x 25 cm, Thermo Scientific) at a flow of 300 nl/min using the Dionex Nano-HPLC system (Thermo Scientific) coupled with the Orbitrap Mass Spectrometer, Q Exactive Plus (Thermo Scientific). For optimal separation of the peptides a linear gradient was applied with a flow of 300 nl/min at 40°C: from 1% to 4% for 4 min, followed by steady 4% for additional 5 min, then 4% to 28% for 90 min, 28% to 50% for 17 min, steady on 50% for another 5min, finally 50% to 80% for 10 min where (solvent A is

0.1% formic acid, and solvent B is 80% acetonitrile, 0.1% formic acid). Run was finished by a steady flow of 80% for 12 min followed by wash and equilibration at 2% ACN for 55 min to prevent carryover of peptides. The Q Exactive mass spectrometer was operated in a data-dependent mode and the survey scan range was set to 200 to 2000 m/z, with a resolution of 70,000 at m/z. The 12 most abundant isotope patterns with a charge of ≥2 and less than seven were subjected to a higher-energy collisional dissociation, with a normalized collision energy of 28 with an isolation window of 1.5 m/z and a resolution of 17,500 at m/z. Data was acquired using the Xcalibur software (Thermo Scientific).

Data analysis of the proteomic data

For identification and quantification of protein we employed the MetaMorpheus software version 1.0.504[12]. We used default parameters of the spectra recalibration, G-PTM-D and database search[15]. For the MS/MS spectra we used 'database search' to search against a UniProtKB database of the E. coli proteome (UniProt ID: UP000000625) with the addition of common contaminants. Identification parameters allowed for two missed cleavages, enzyme specificity was set to trypsin, and peptide length was limited to minimum length of 5 amino acids to be considered for identification. Samples were analyzed first with Carbamidomethylation or Ynamide mass set as a fixed modification, followed by analysis with carbamidomethyl and Ynamide as variable modifications for comparison purposes, with maximum number of modifications per peptides set to 5. A false discovery (FDR) of 1% was applied using the decoy database strategy. An initial precursor mass deviation of up to 5 ppm and fragment mass deviation up to 20 ppm were allowed. The G-PTM-D variable modifications included: 'common biological', 'common artifact', potential modifications due to crosslinking of carbon adduct on the peptide N-terminal and carbamidomethyl on non-cysteine amino acids as well as Ynamide mass on cysteine containing and non-cysteine amino acids. Only proteins identified by two or more unique peptides were considered for analysis, also the match-between-runs option was checked, and data was normalized. To quantify the changes, we used the embedded flash - label free quantification (FlashLFQ) using the default parameters[16]. Proteomic data was uploaded to the PRIDE database[17] with the dataset identifier: 1-20221212-125206.

14. References

1. (1) Tu, Y.; Zeng, X.; Wang, H.; Zhao, J. A Robust One-Step Approach to Ynamides. *Org. Lett.*, **20**, 280-283, (2018); (2) Mansfield, S. J.; Campbell, C. D.; Jones, M. W.; Anderson, E. A. A robust and modular synthesis of ynamides. Chem. Commun.,**51**, 3316 (2015).
2. Dawson, P. E.; Muir, T. W.; Clark-Lewis, I.; Kent, S. B. H., Synthesis of proteins by native chemical ligation. *Science*, **266**, 776-779, (1994).
3. Flood, D. T.; Hintzen, J. C. J.; Bird, M. J.; Cistrone, P. A.; Chen, J. S.; Dawson, P. E., Leveraging the Knorr Pyrazole Synthesis for the Facile Generation of Thioester Surrogates for use in Native Chemical Ligation. *Angew Chem Int Ed,* **57**, 11634-11639 (2018).
4. Hu, L.; Xu, S.; Zhao, Z.; Yang, Y.; Peng, Z.; Yang, M.; Wang, C.; Zhao, J., Ynamides as Racemization-Free Coupling Reagents for Amide and Peptide Synthesis. *J. Am. Chem. Soc.*, **138**, 13135-13138 (2016).
5. Yang, Y.; Liu, H.; Peng, C.; Wu, J.; Zhang, J.; Qiao, Y.; Wang, X.-N.; Chang, J., $AlCl_3$-Catalyzed Annulations of Ynamides Involving a Torquoselective Process for the Simultaneous Control of Central and Axial Chirality. *Org. Lett.*, **18**, 5022-5025 (2016).
6. Lewis, W. G.; Magallon, F. G.; Fokin, V. V.; Finn, M. G., Discovery and Characterization of Catalysts for Azide−Alkyne Cycloaddition by Fluorescence Quenching. *J Am Chem Soc*, **126**, 9152-3 (2014).
7. Dery, S.; Reddy, P. S.; Dery, L.; Mousa, R.; Dardashti, R. N.; Metanis, N., Insights into the deselenization of selenocysteine into alanine and serine. *Chem Sci*, ***6***, 6207-6212 (2015).
8. Zhao, Z.; Shimon, D.; Metanis, N., Chemoselective Copper-Mediated Modification of Selenocysteines in Peptides and Proteins. *J Am Chem Soc*, **143**, 12817-12824 (2021).
9. Mousa, R.; Lansky, S.; Shoham, G.; Metanis, N., BPTI folding revisited: switching a disulfide into methylene thioacetal reveals a previously hidden path. *Chem. Sci.*, **9**, 4814-4820 (2018).
10. Wiśniewski, J. R.; Zougman, A.; Nagaraj, N.; Mann, M. Universal sample preparation method for proteome analysis. *Nat. Methods* **6**, 359-362, (2009).
11. Radzinski, M.; Fassler, R.; Yogev, O.; Breuer, W.; Shai, N.; Gutin, J.; Ilyas, S.; Geffen, Y.; Tsytkin-Kirschenzweig, S.; Nahmias, Y.; Ravid, T.; Friedman, N.; Schuldiner, M.; Reichmann, D. Temporal profiling of redox-dependent heterogeneity in single cells. *eLife* **7**, e37623, (2018).
12. Solntsev, S. K.; Shortreed, M. R.; Frey, B. L.; Smith, L. M. Enhanced Global Post-translational Modification Discovery with MetaMorpheus. *J. Proteome Res.* **17**, 1844-1851, (2018).
13. Nielsen, M. L.; Vermeulen, M.; Bonaldi, T.; Cox, J.; Moroder, L.; Mann, M.

Iodoacetamide-induced artifact mimics ubiquitination in mass spectrometry. *Nat. Methods* **5**, 459-460, (2008).
14. Wang, R.; Fabregat, A.; Ríos, D.; Ovelleiro, D.; Foster, J. M.; Côté, R. G.; Griss, J.; Csordas, A.; Perez-Riverol, Y.; Reisinger, F.; Hermjakob, H.; Martens, L.; Vizcaíno, J. A. PRIDE Inspector: a tool to visualize and validate MS proteomics data. *Nat. Biotechnol.* **30**, 135-137, (2012).
15. Schoffman, H. *et al.* Comparison of matched formalin-fixed paraffin embedded and fresh frozen meningioma tissue reveals bias in proteomic profiles. *PROTEOMICS* **22**, 2200085, doi:https://doi.org/10.1002/pmic.202200085 (2022).
16. Millikin, R. J.; Solntsev, S. K.; Shortreed, M. R.; Smith, L. M., Ultrafast Peptide Label-Free Quantification with FlashLFQ. *J.Pro. Res.***17**, 386-391 (2018).
17. Vizcaino, J. A. et al. The PRoteomics IDEntifications (PRIDE) database and associated tools: status in 2013. *Nucleic Acids Res.* **41**, D1063–D1069 (2013).

15. NMR Spectrum

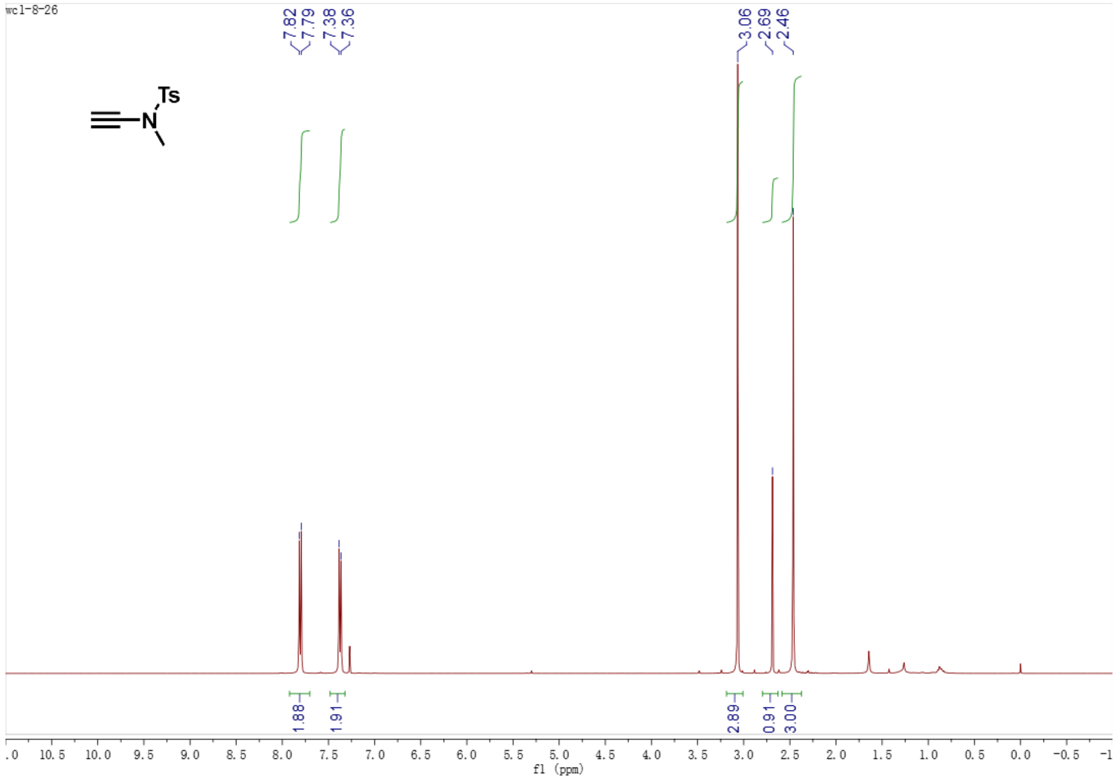

Figure S120. ^{1}H-NMR (400 MHz) spectrum of compound **2a** in $CDCl_3$

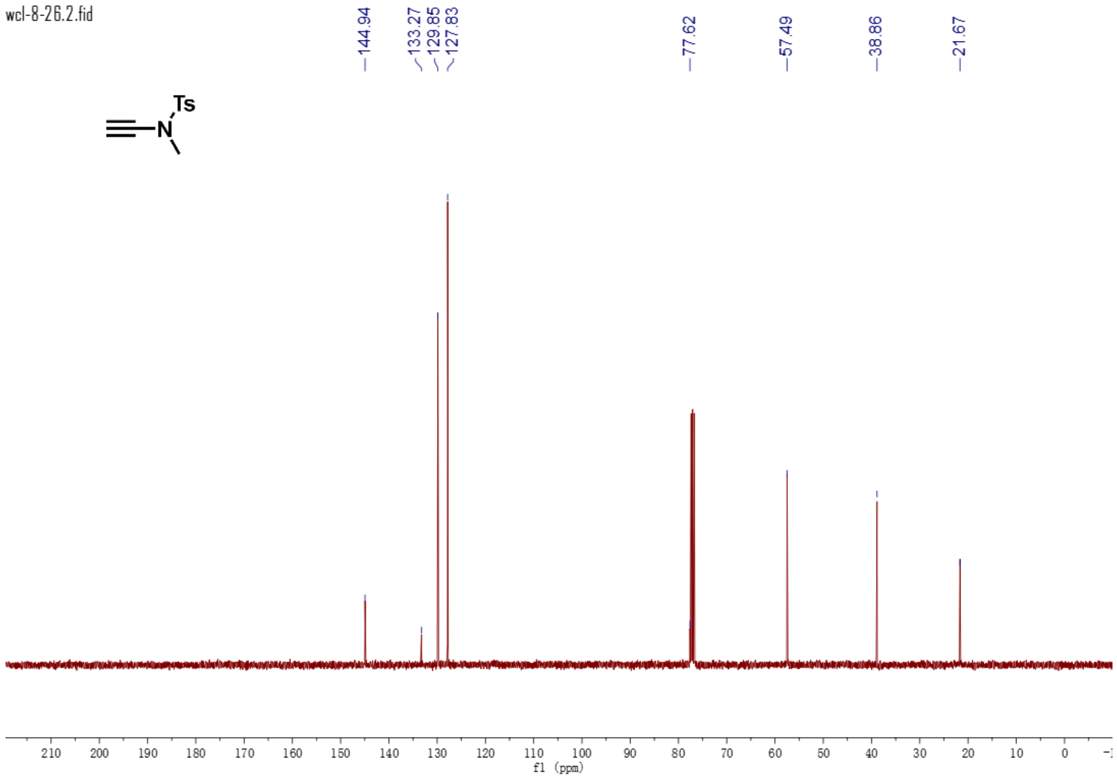

Figure S121. ^{13}C-NMR (100 MHz) spectrum of compound **2a** in $CDCl_3$

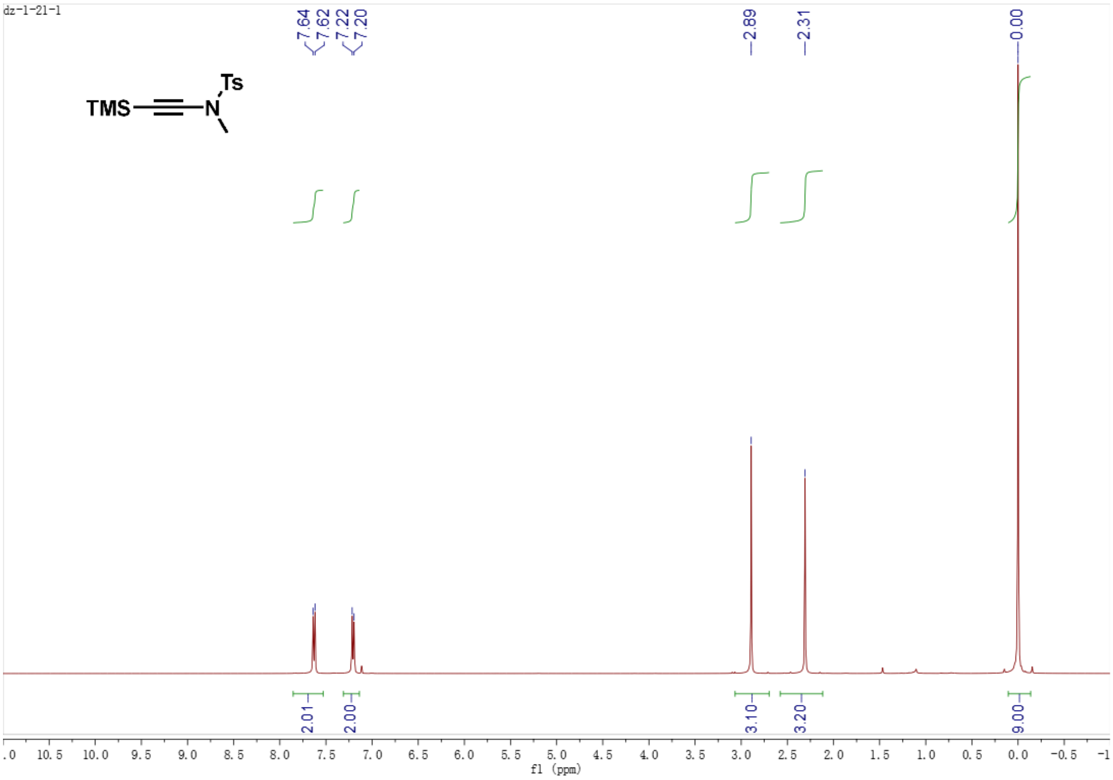

Figure S122. ^{1}H-NMR (400 MHz) spectrum of compound **2c** in $CDCl_3$

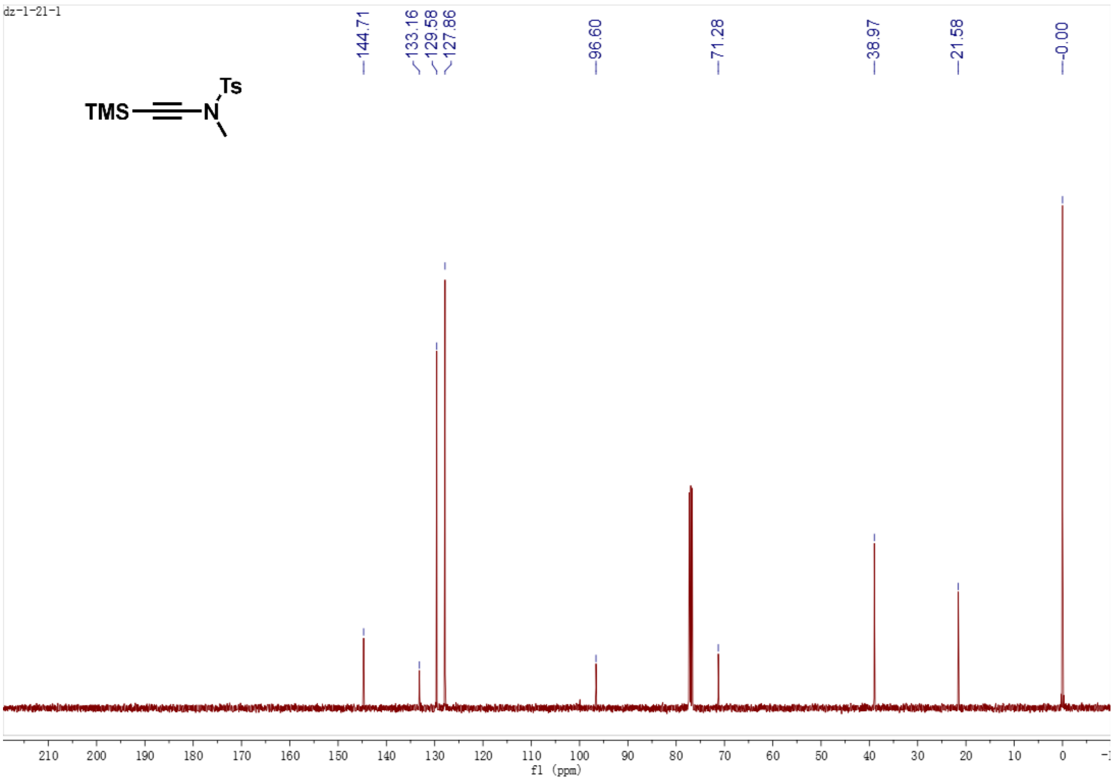

Figure S123. ^{13}C-NMR (100 MHz) spectrum of compound **2c** in $CDCl_3$

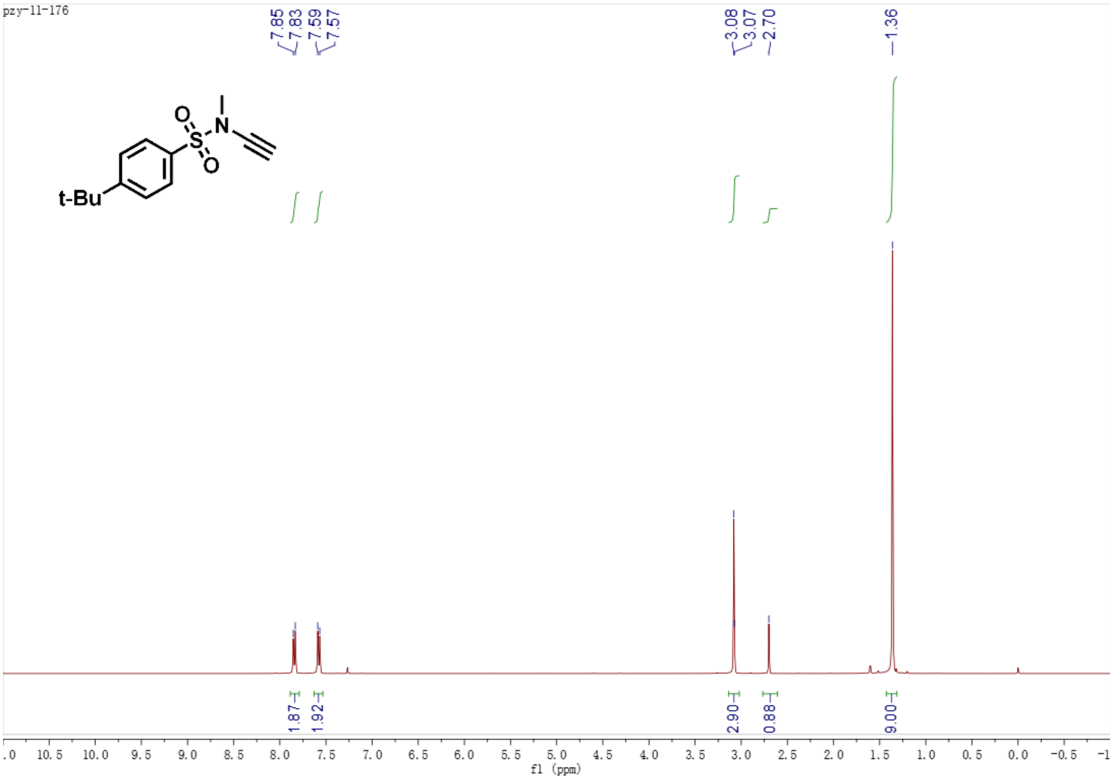

Figure S124. ^{1}H-NMR (400 MHz) spectrum of compound **2d** in $CDCl_3$

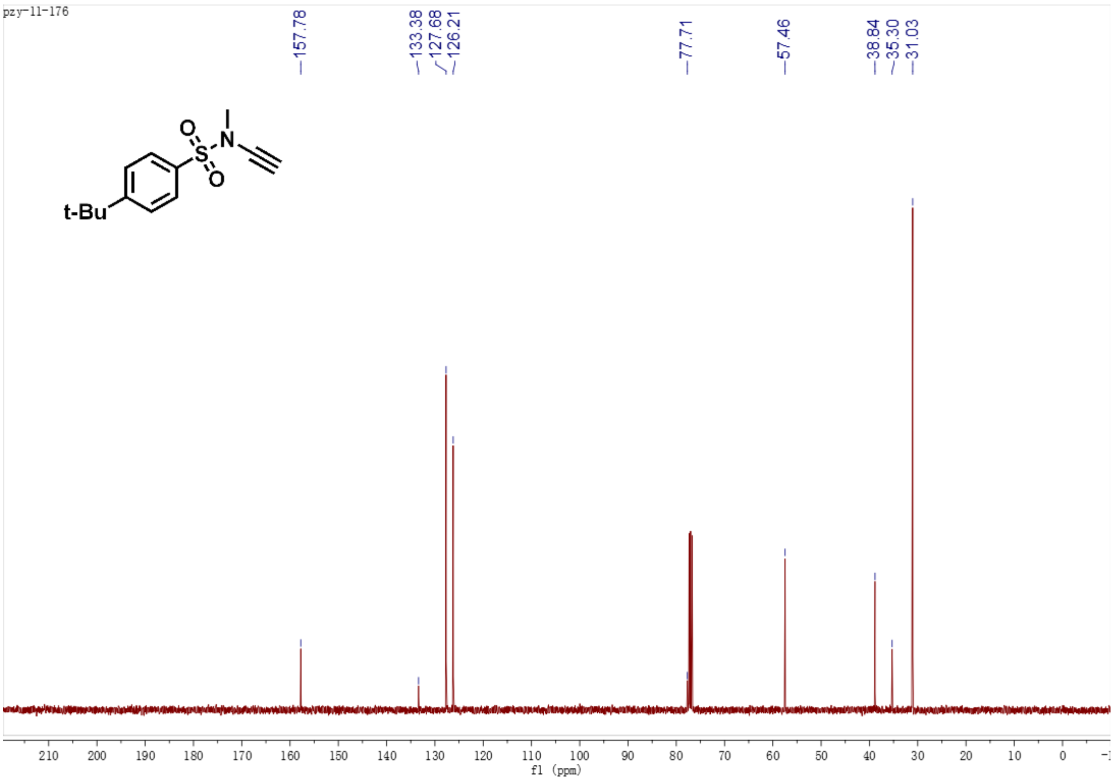

Figure S125. ^{13}C-NMR (100 MHz) spectrum of compound **2d** in $CDCl_3$

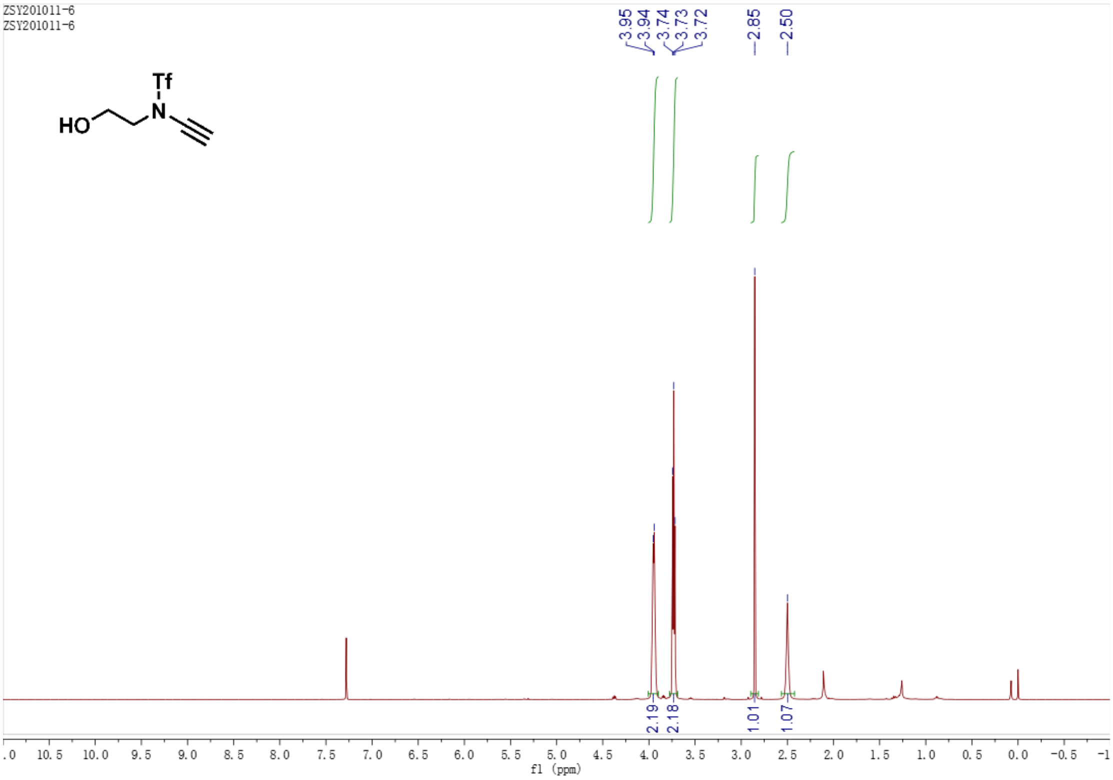

Figure S126. ^{1}H-NMR (400 MHz) spectrum of compound **2e** in $CDCl_3$

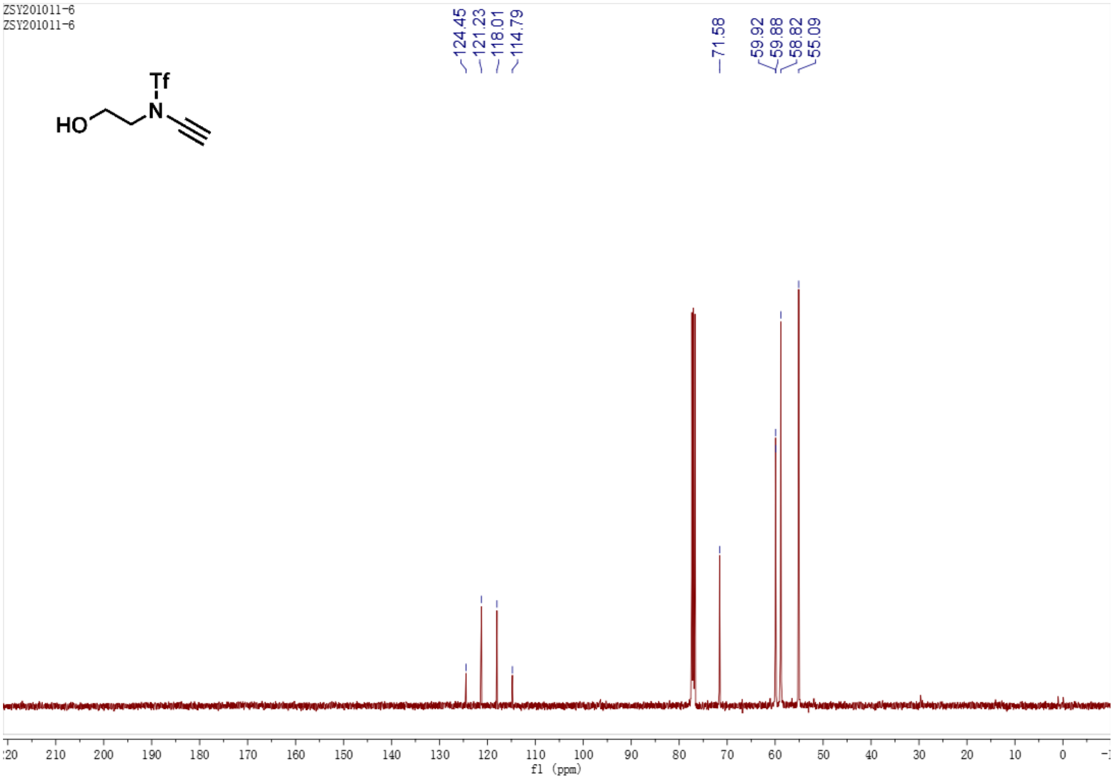

Figure S127. ^{13}C-NMR (100 MHz) spectrum of compound **2e** in $CDCl_3$

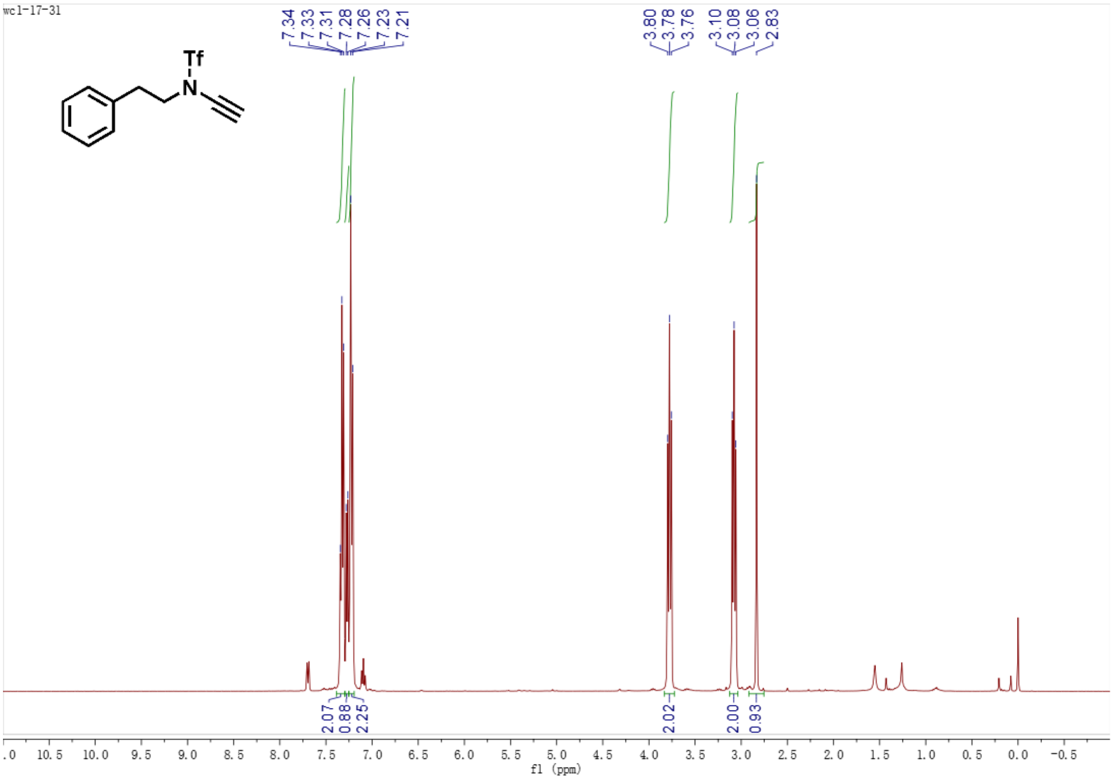

Figure S128. ^{1}H-NMR (400 MHz) spectrum of compound **2f** in $CDCl_3$

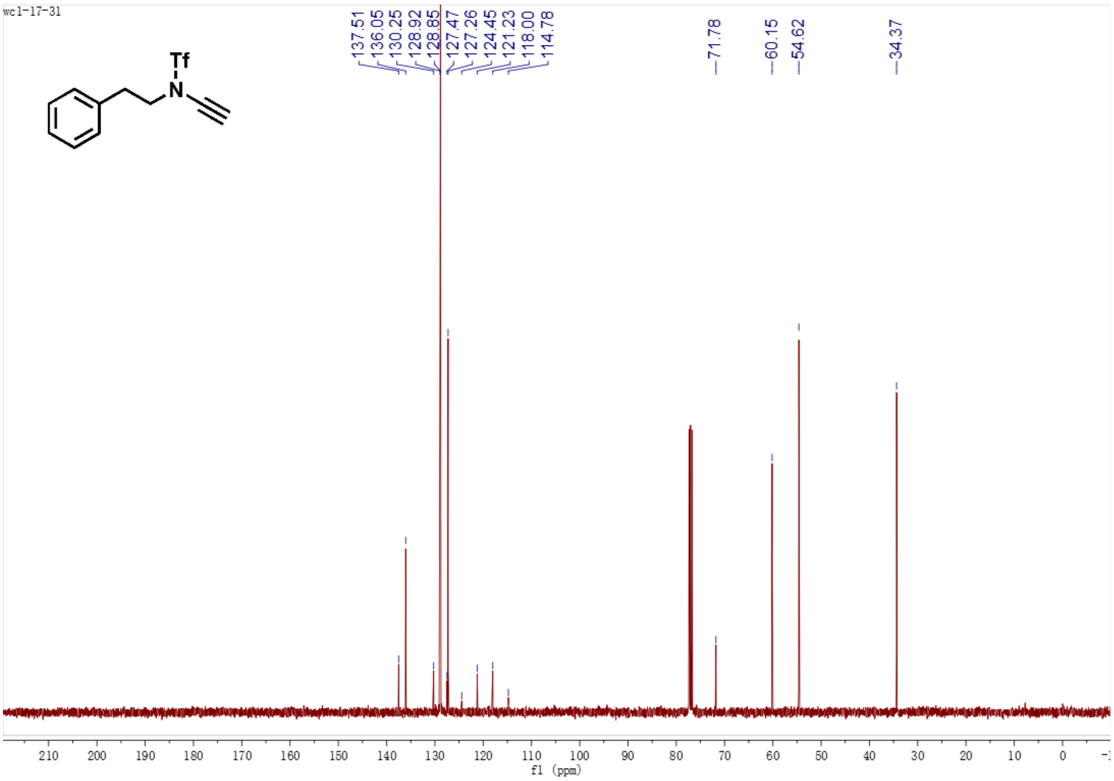

Figure S129. ^{13}C-NMR (100 MHz) spectrum of compound **2f** in $CDCl_3$

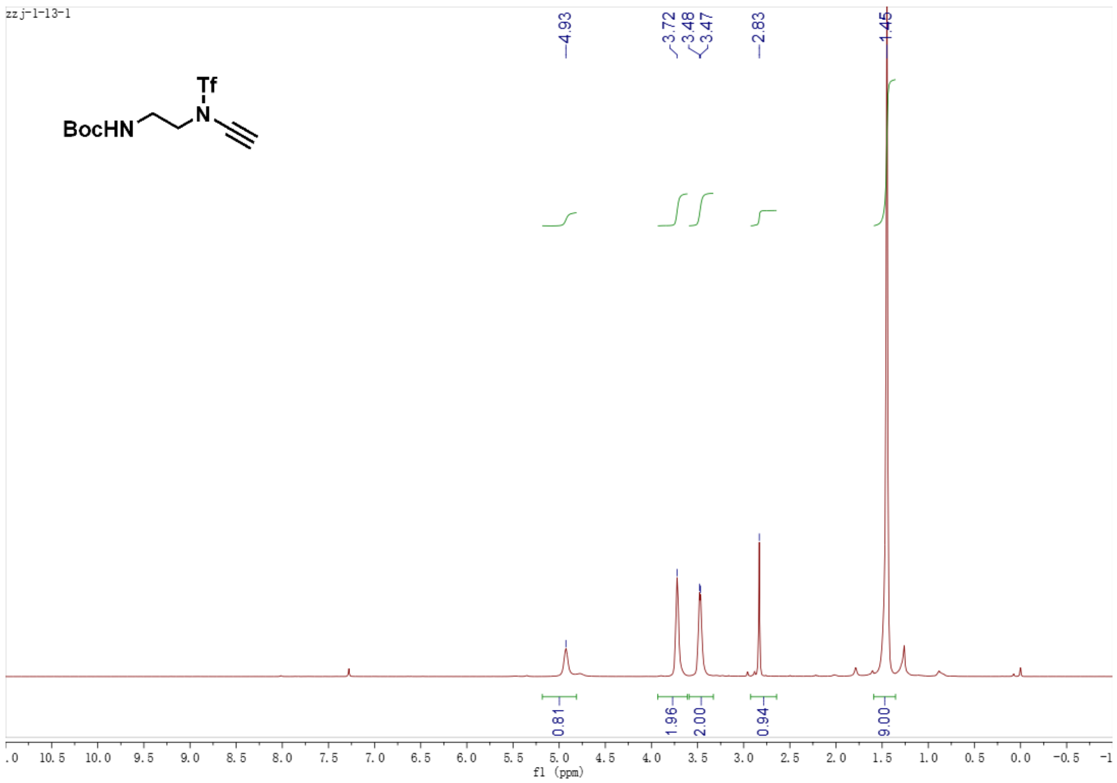

Figure S130. ^{1}H-NMR (400 MHz) spectrum of compound **2g** in $CDCl_3$

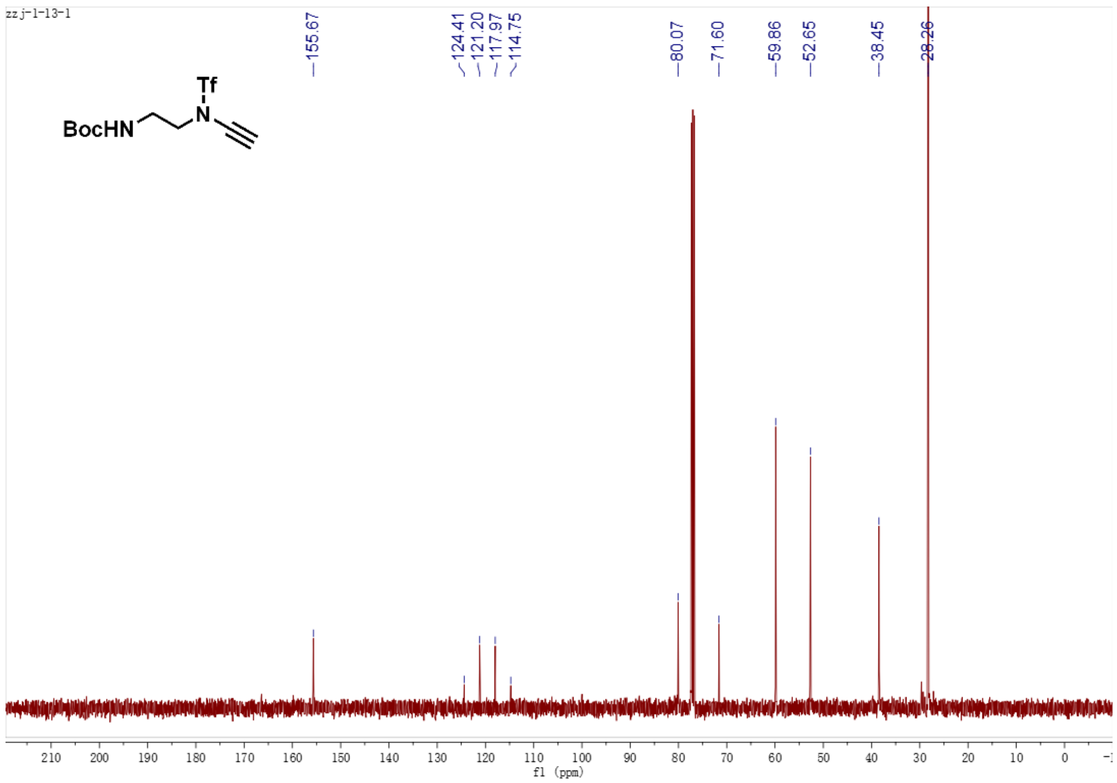

Figure S131. ^{13}C-NMR (100 MHz) spectrum of compound **2g** in $CDCl_3$

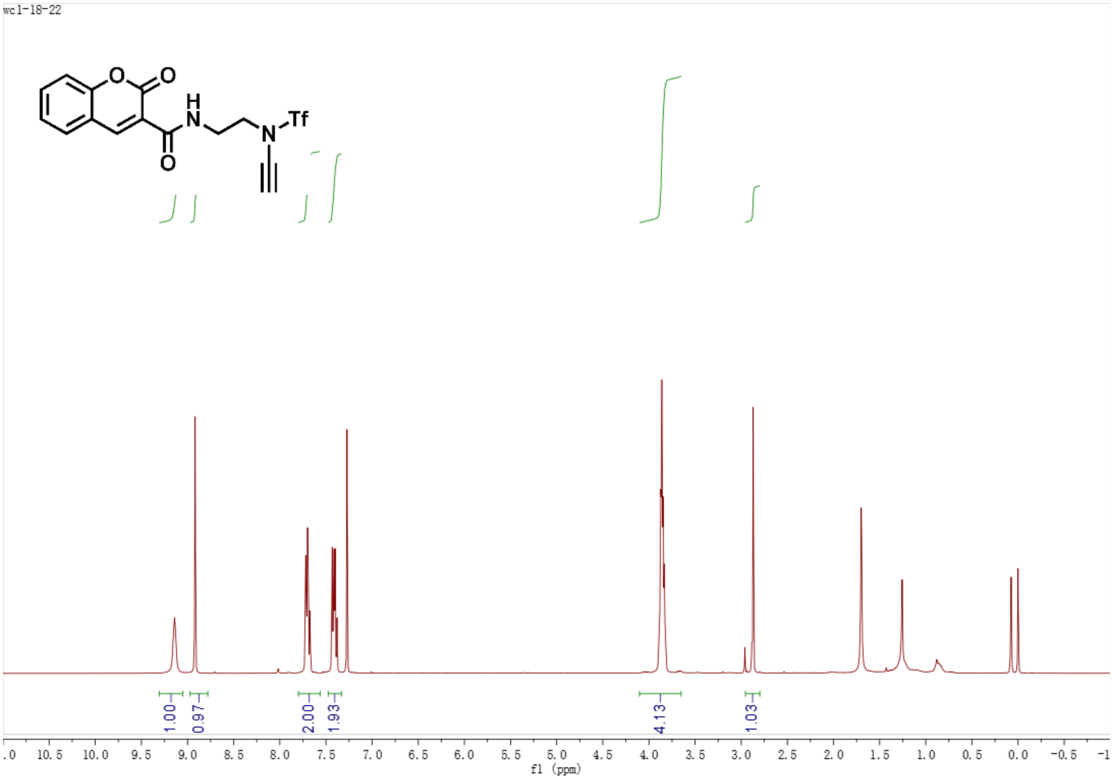

Figure S132. ^{1}H-NMR (400 MHz) spectrum of compound **2h** in $CDCl_3$

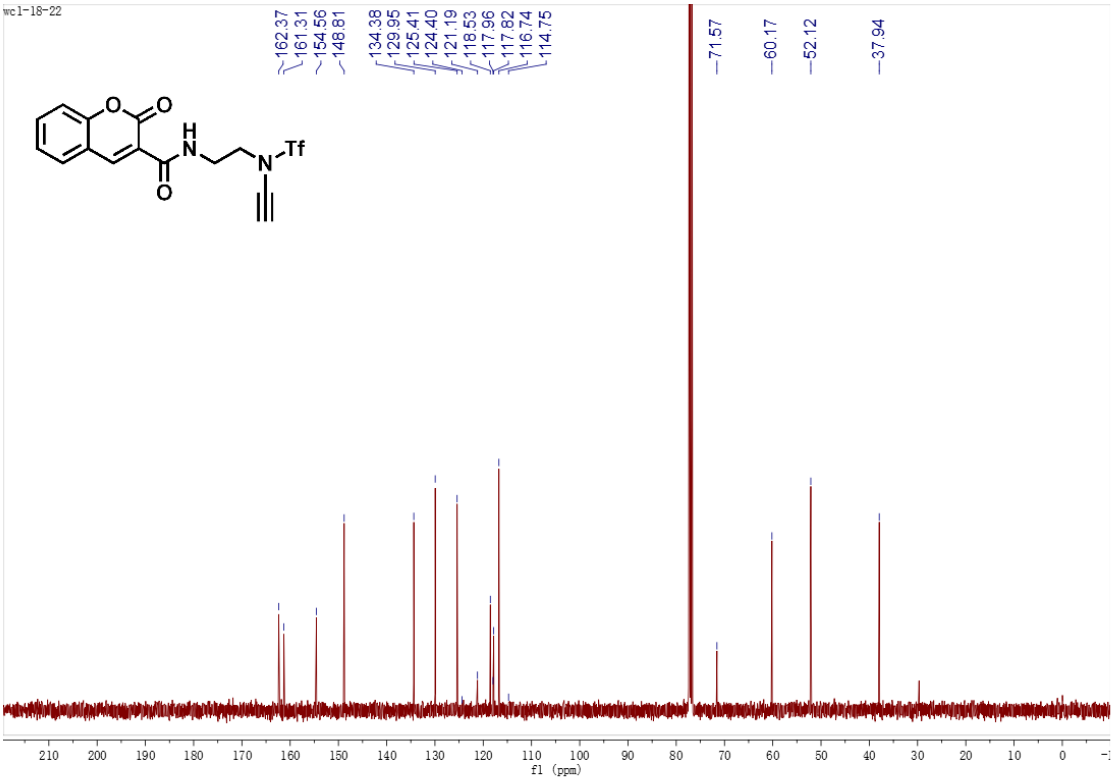

Figure S133. ^{13}C-NMR (100 MHz) spectrum of compound **2h** in $CDCl_3$

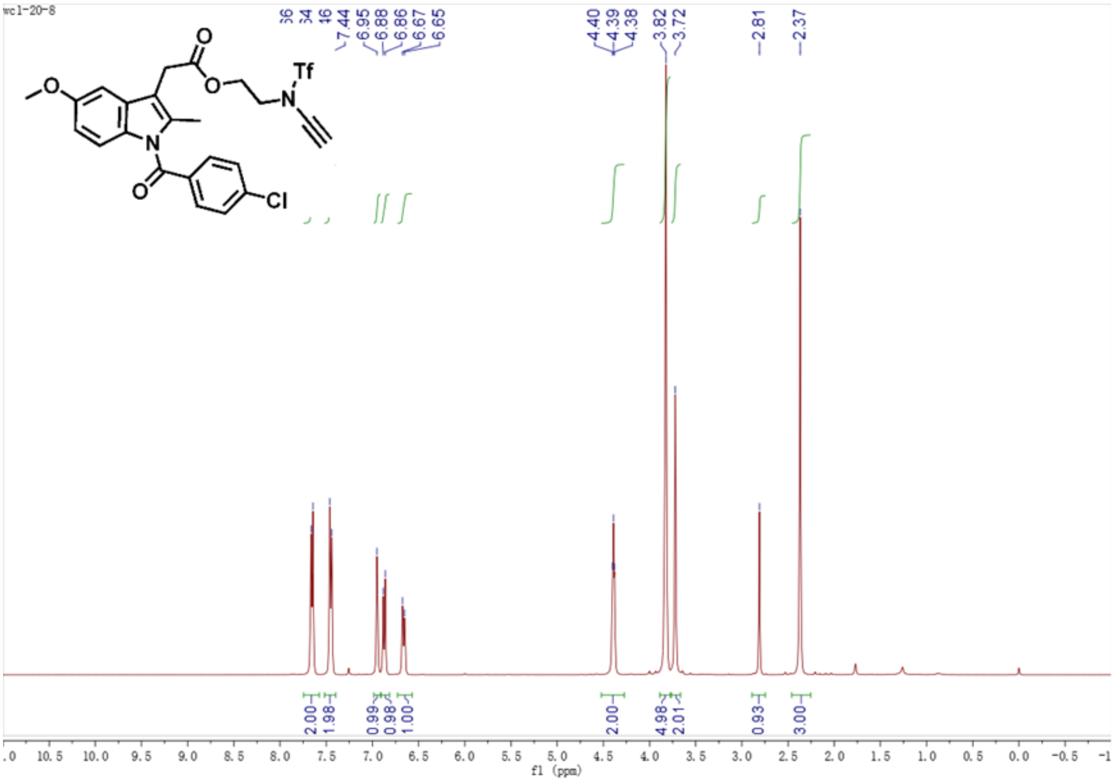

Figure S134. ^{1}H-NMR (400 MHz) spectrum of compound **2i** in $CDCl_3$

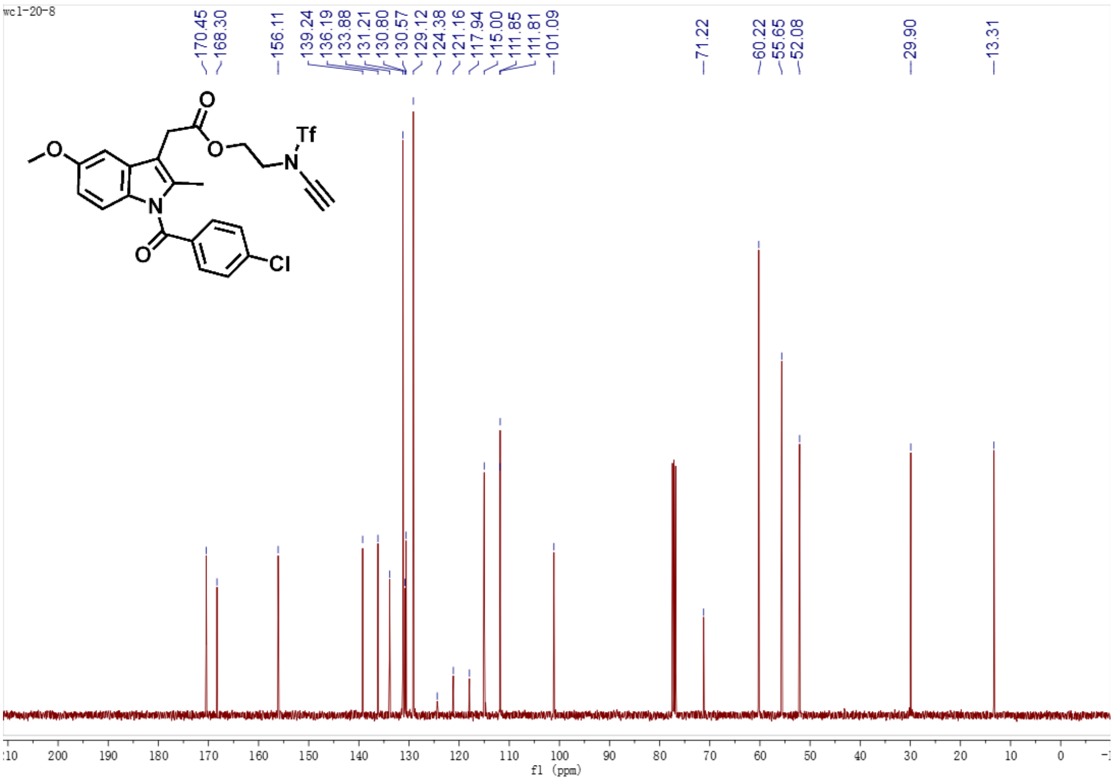

Figure S135. ^{13}C-NMR (100 MHz) spectrum of compound **2i** in $CDCl_3$

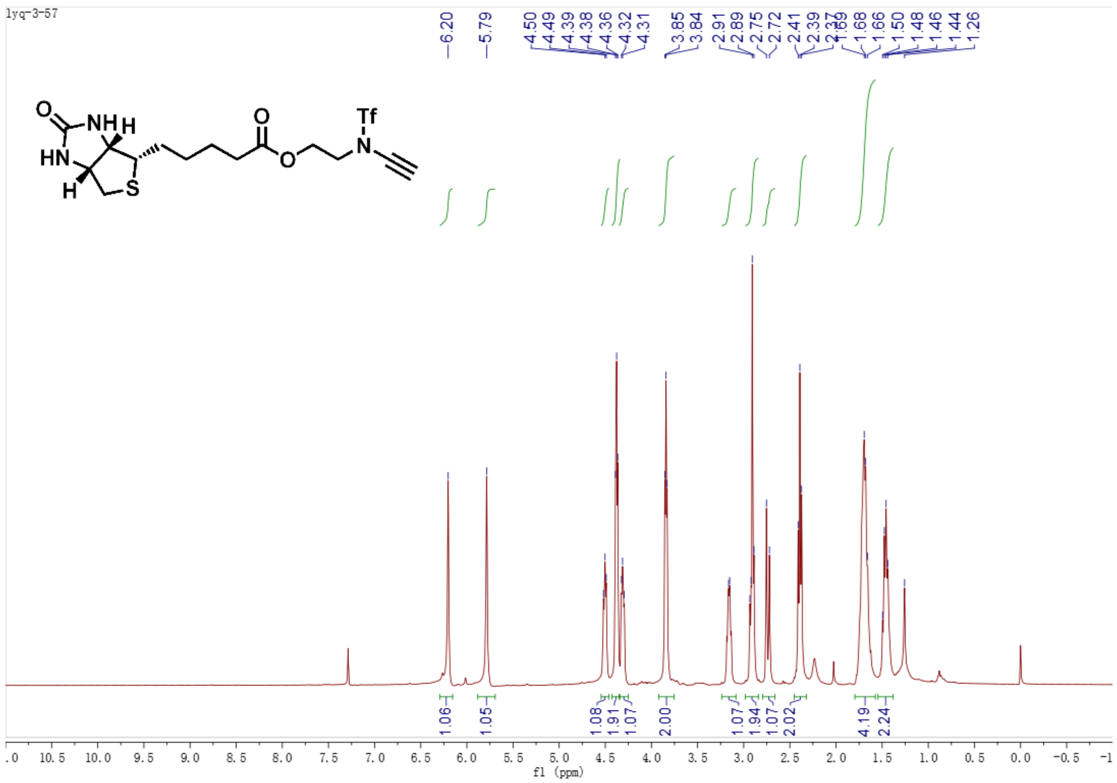

Figure S136. ^{1}H-NMR (400 MHz) spectrum of compound **2j** in $CDCl_3$

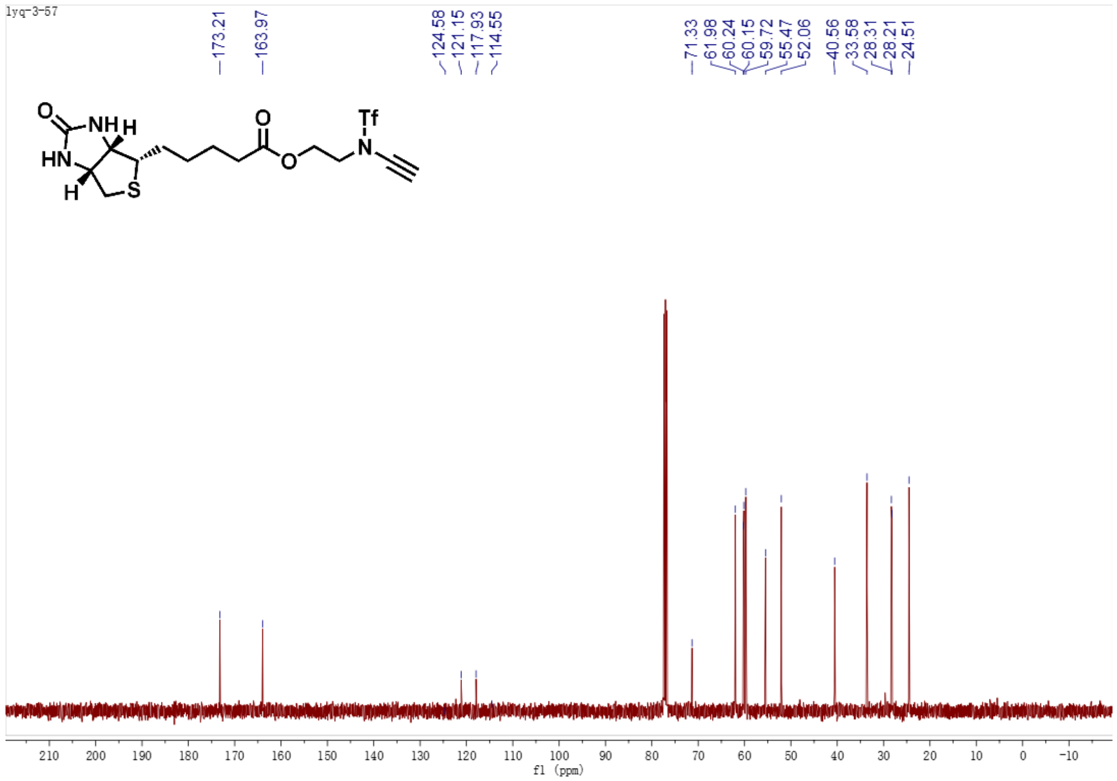

Figure S137. ^{13}C-NMR (100 MHz) spectrum of compound **2j** in $CDCl_3$

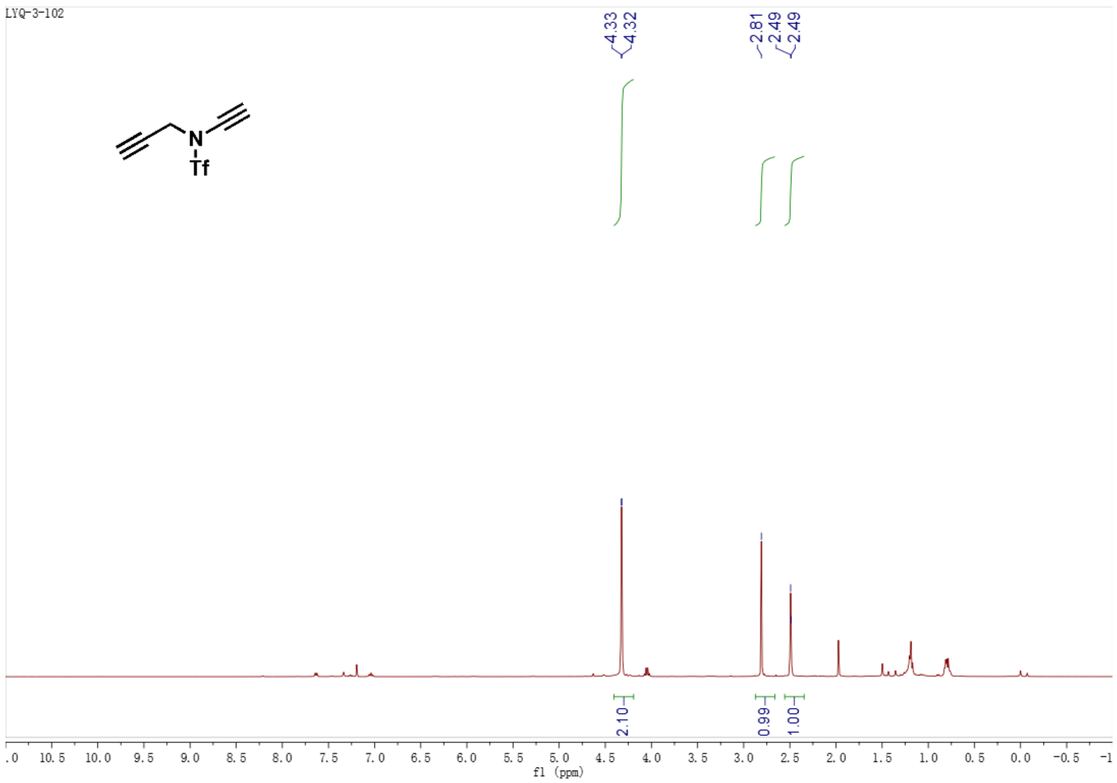

Figure S138. ^{1}H-NMR (400 MHz) spectrum of compound **2k** in $CDCl_3$

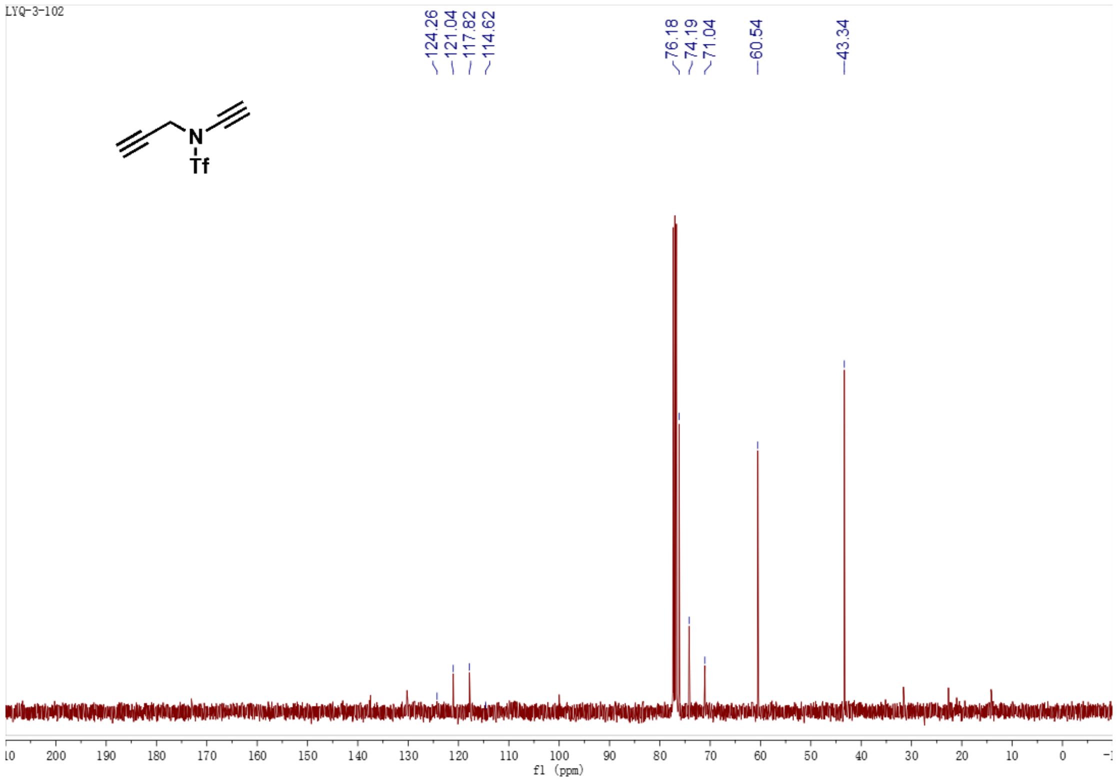

Figure S139. ^{13}C-NMR (100 MHz) spectrum of compound **2k** in $CDCl_3$

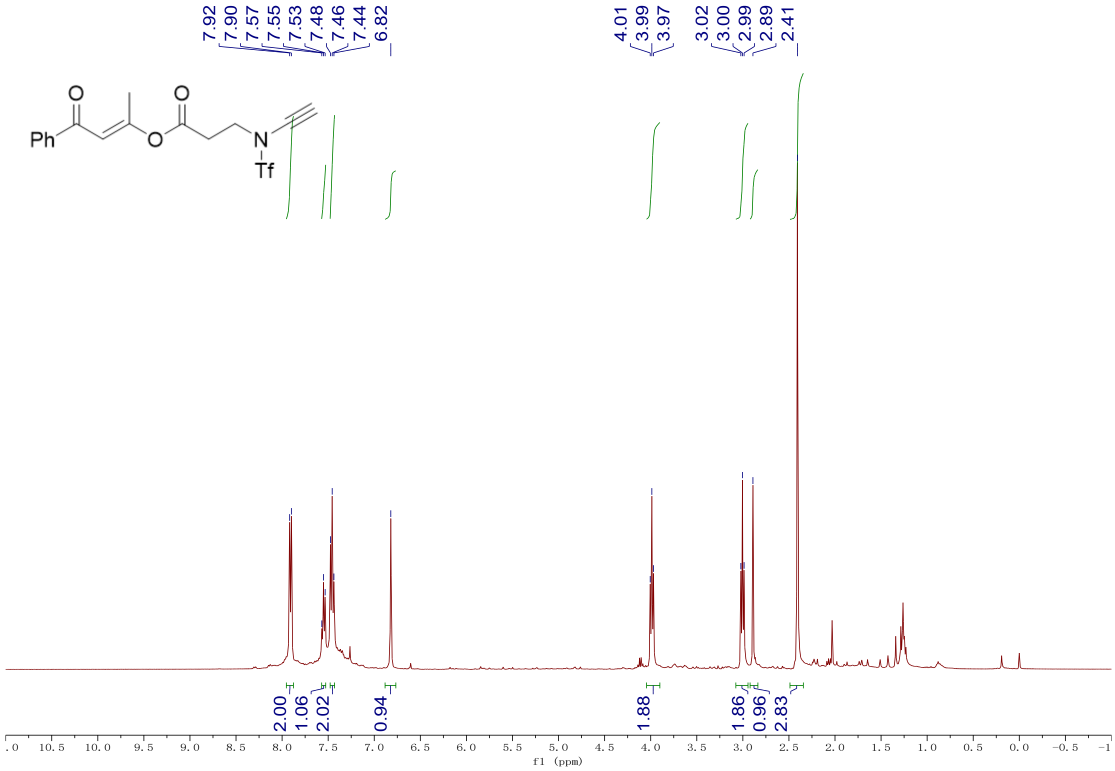

Figure S140. ^{1}H-NMR (400 MHz) spectrum of compound **31^{d}** in $CDCl_3$

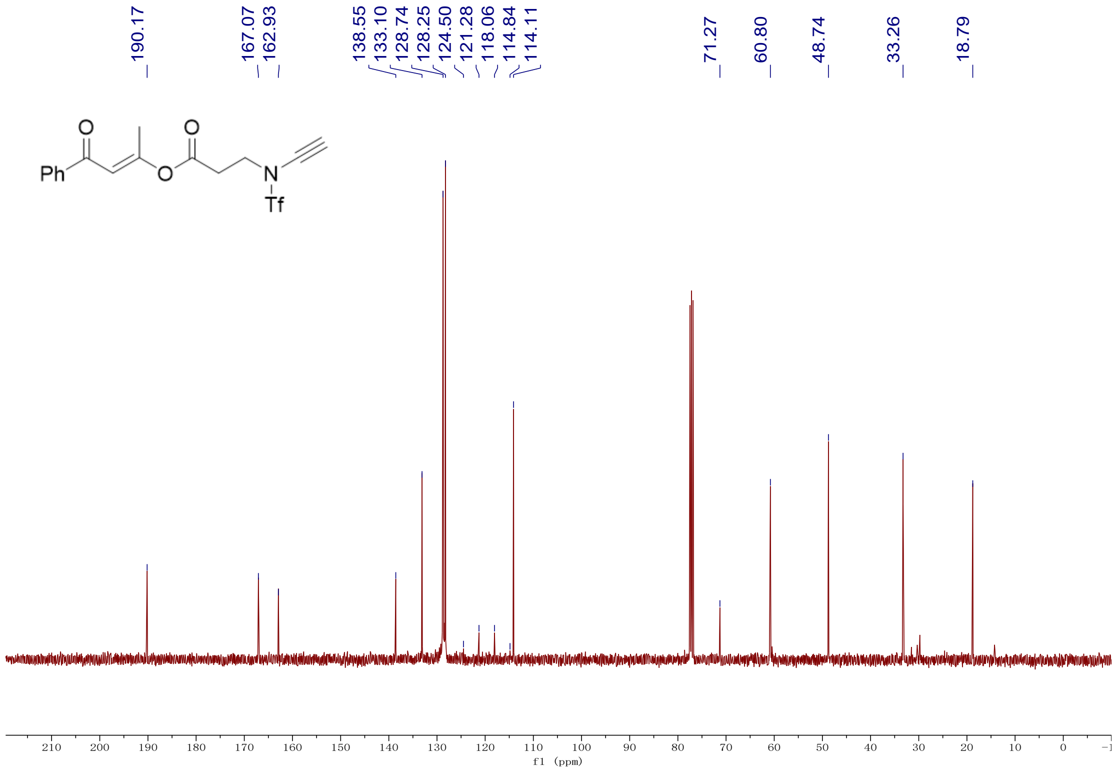

Figure S141. ^{13}C-NMR (100 MHz) spectrum of compound **31^{d}** in $CDCl_3$

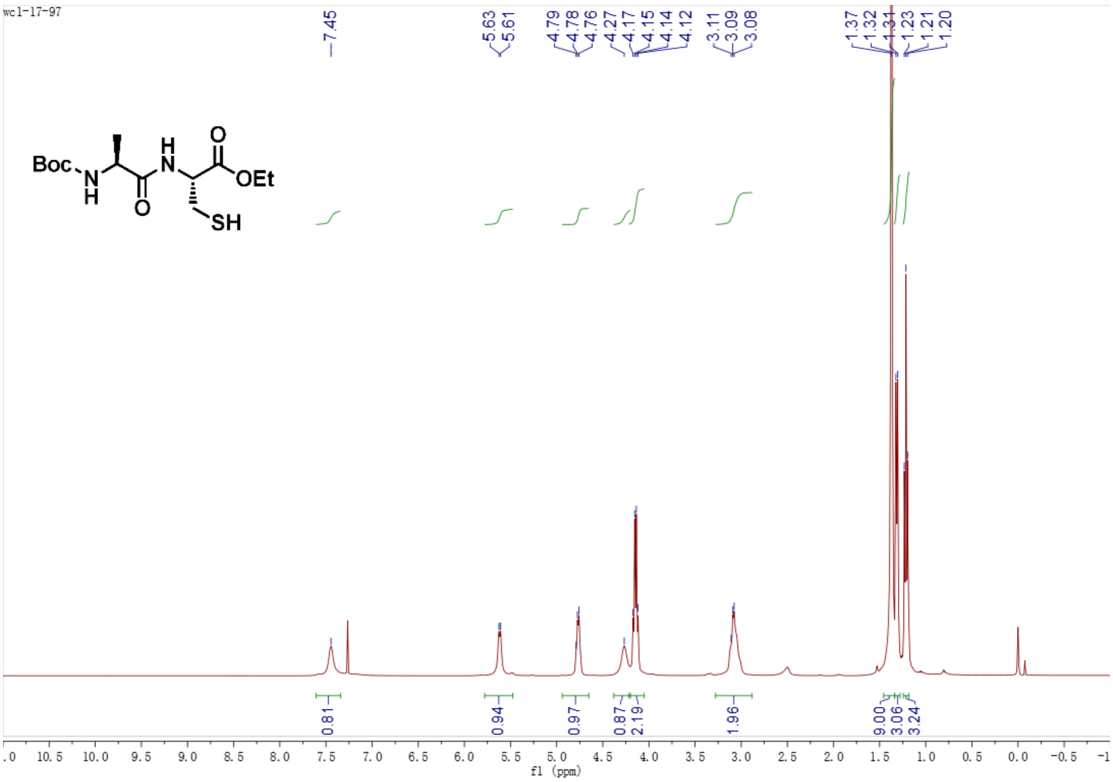

Figure S142. ^{1}H-NMR (400 MHz) spectrum of compound **1a** in $CDCl_3$

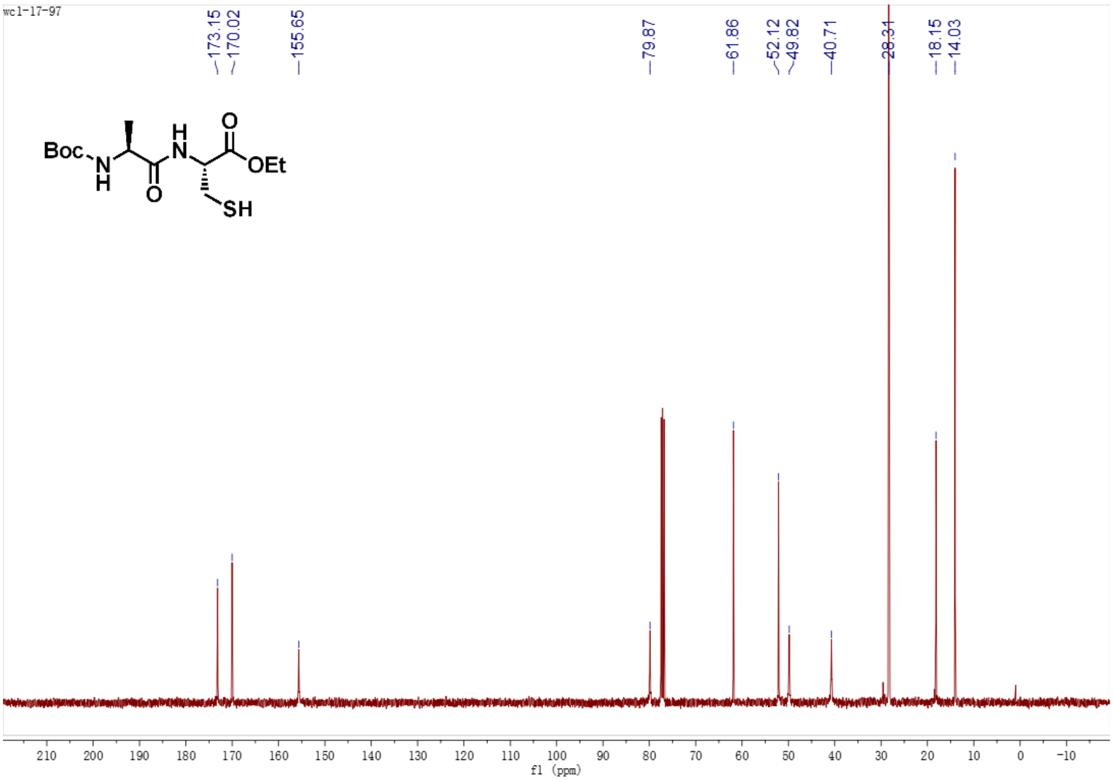

Figure S143. ^{13}C-NMR (100 MHz) spectrum of compound **1a** in $CDCl_3$

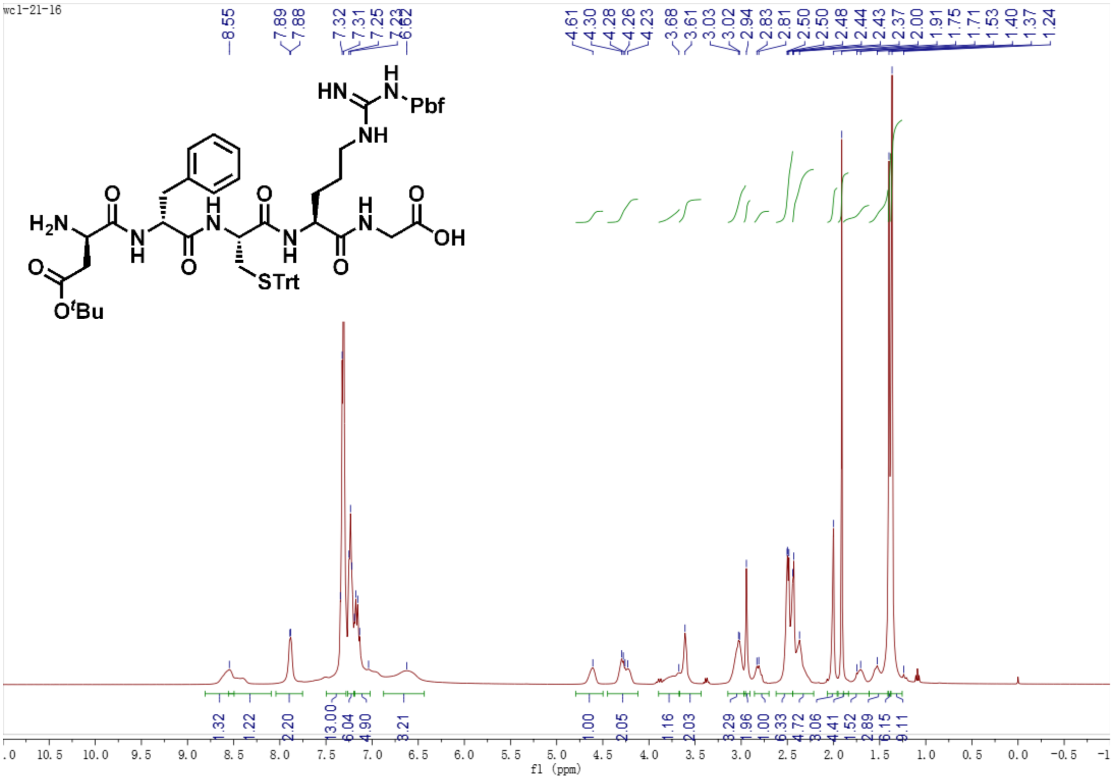

Figure S144. ^{1}H-NMR (400 MHz) spectrum of compound in DMSO-d_6

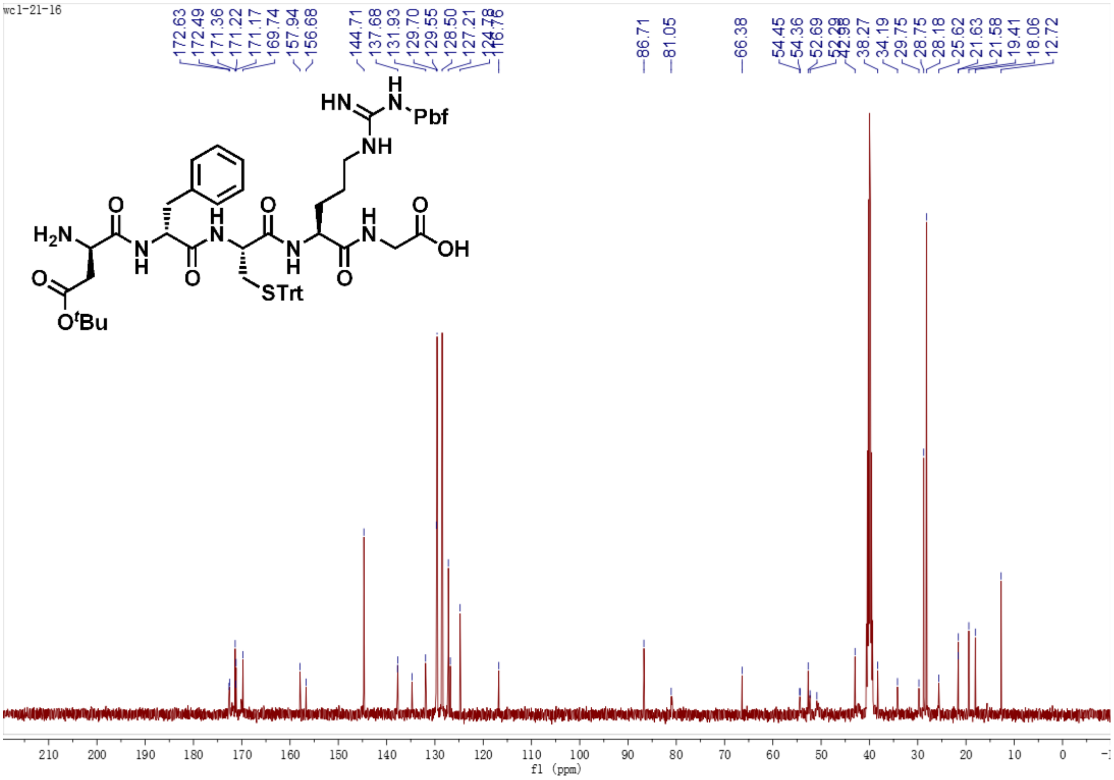

Figure S145. ^{13}C-NMR (100 MHz) spectrum of compound in DMSO-d_6

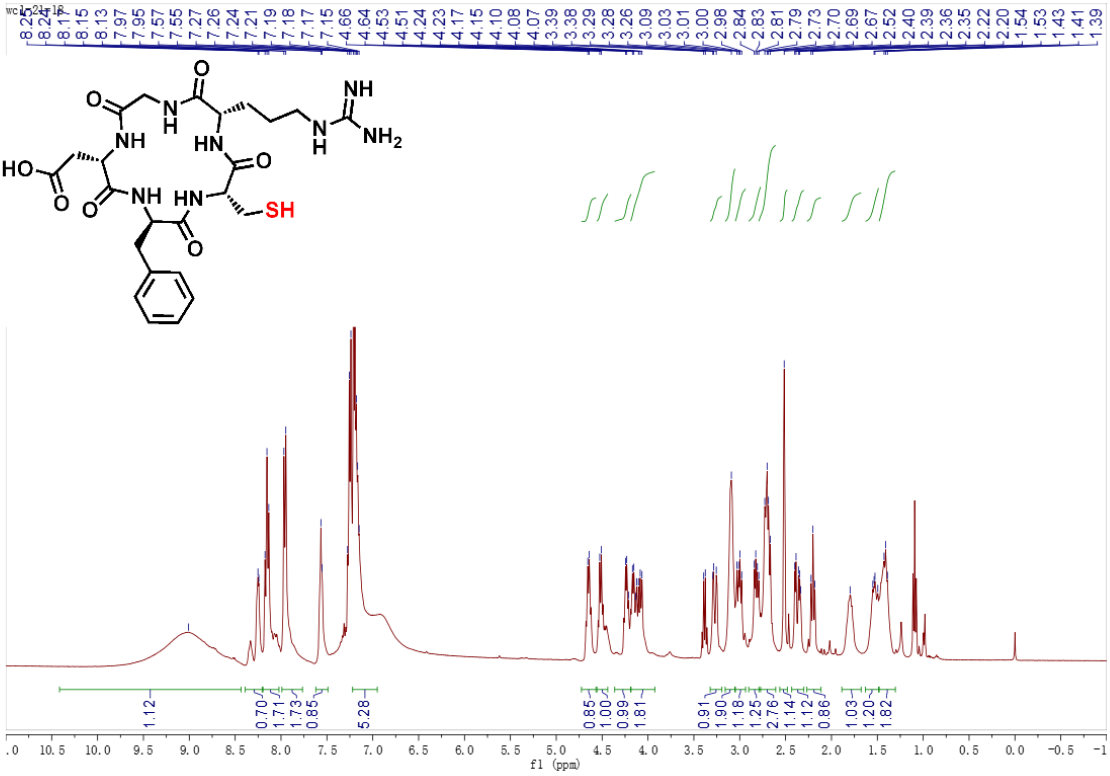

Figure S146. ^{1}H-NMR (400 MHz) spectrum of compound **1o** in DMSO-d_6

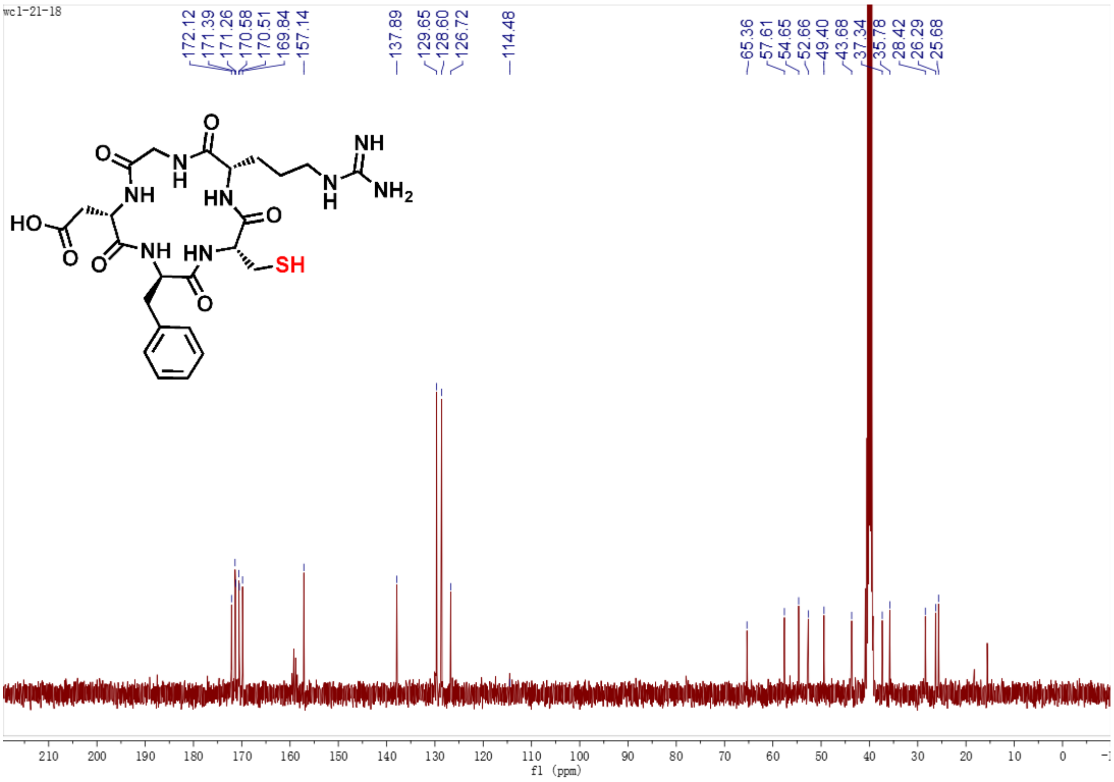

Figure S147. ^{13}C-NMR (100 MHz) spectrum of compound **1o** in DMSO-d_6

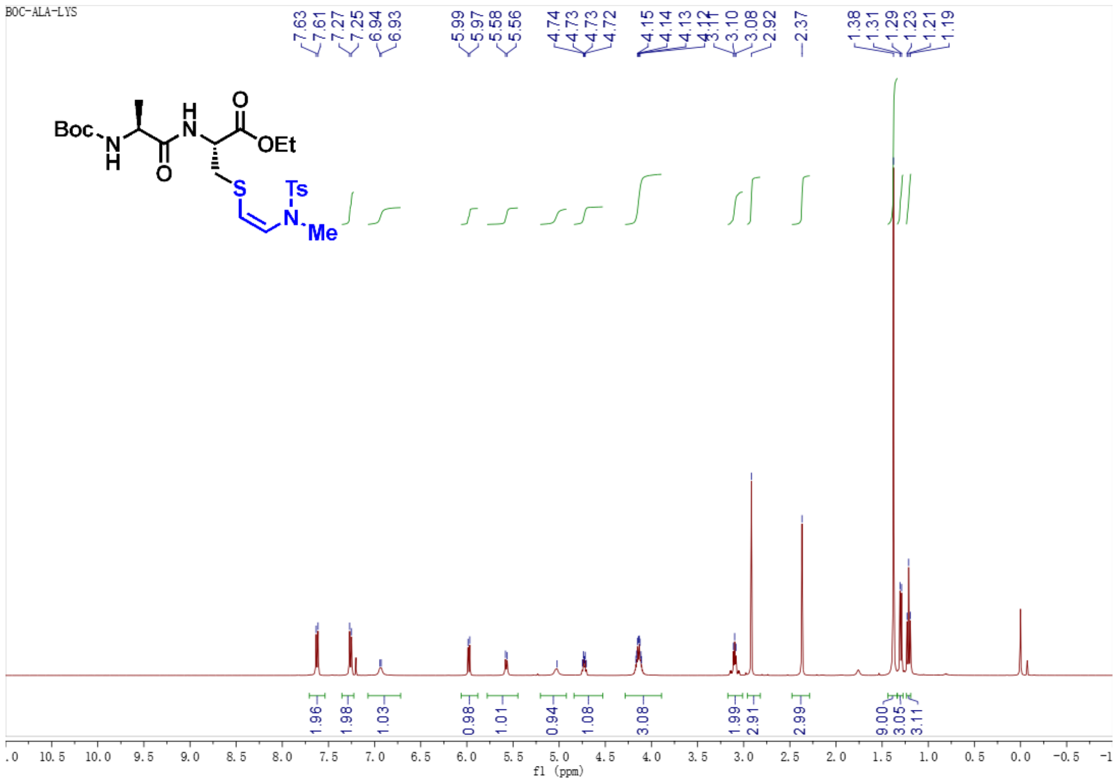

Figure S148. ^{1}H-NMR (400 MHz) spectrum of compound **3aa** in $CDCl_3$

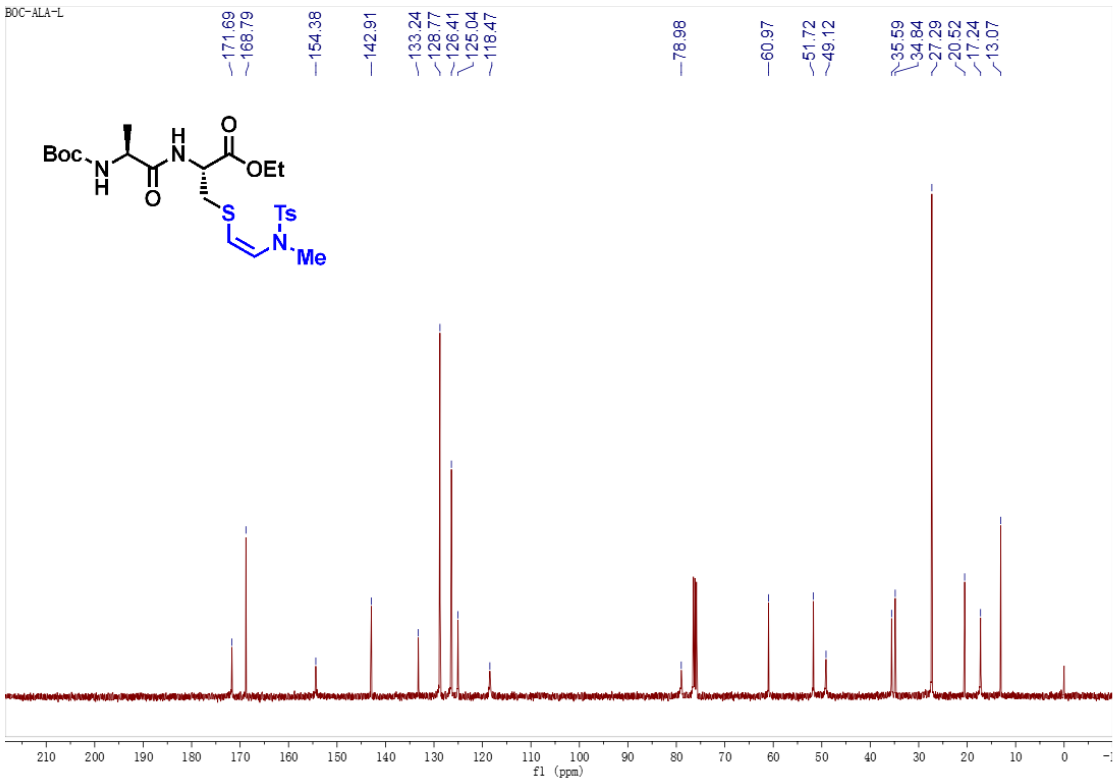

Figure S149. ^{13}C-NMR (100 MHz) spectrum of compound **3aa** in $CDCl_3$

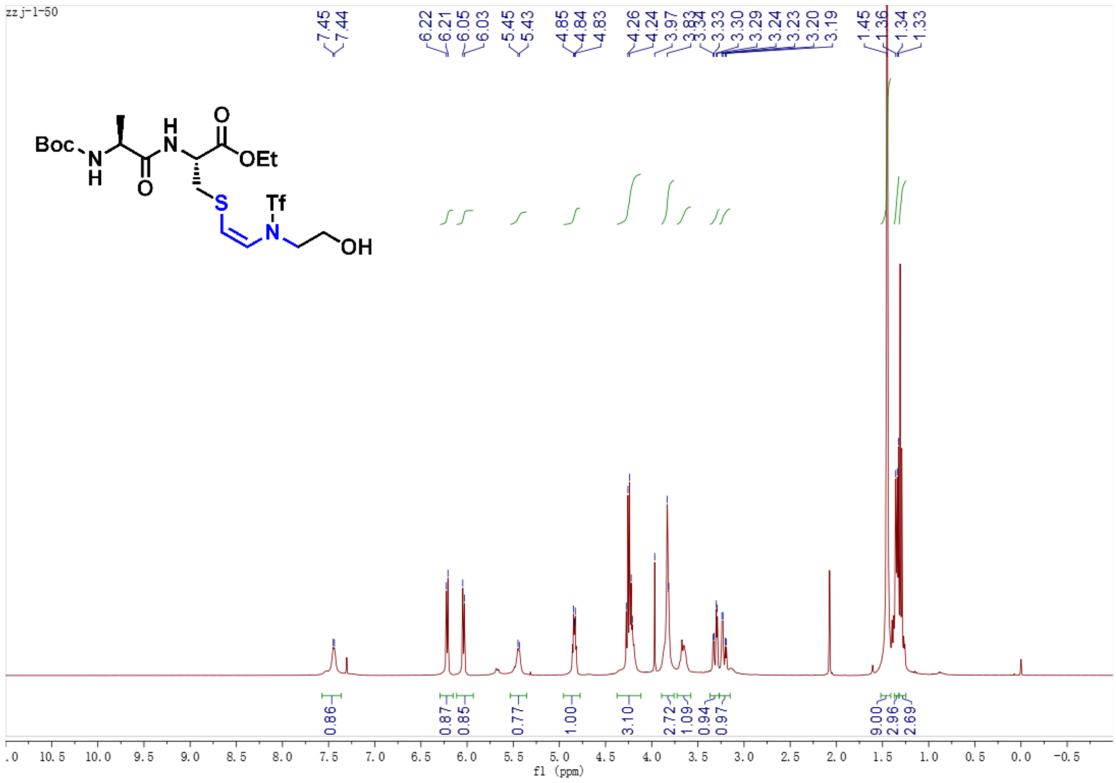

Figure S150. ^{1}H-NMR (400 MHz) spectrum of compound **3ae** in $CDCl_3$

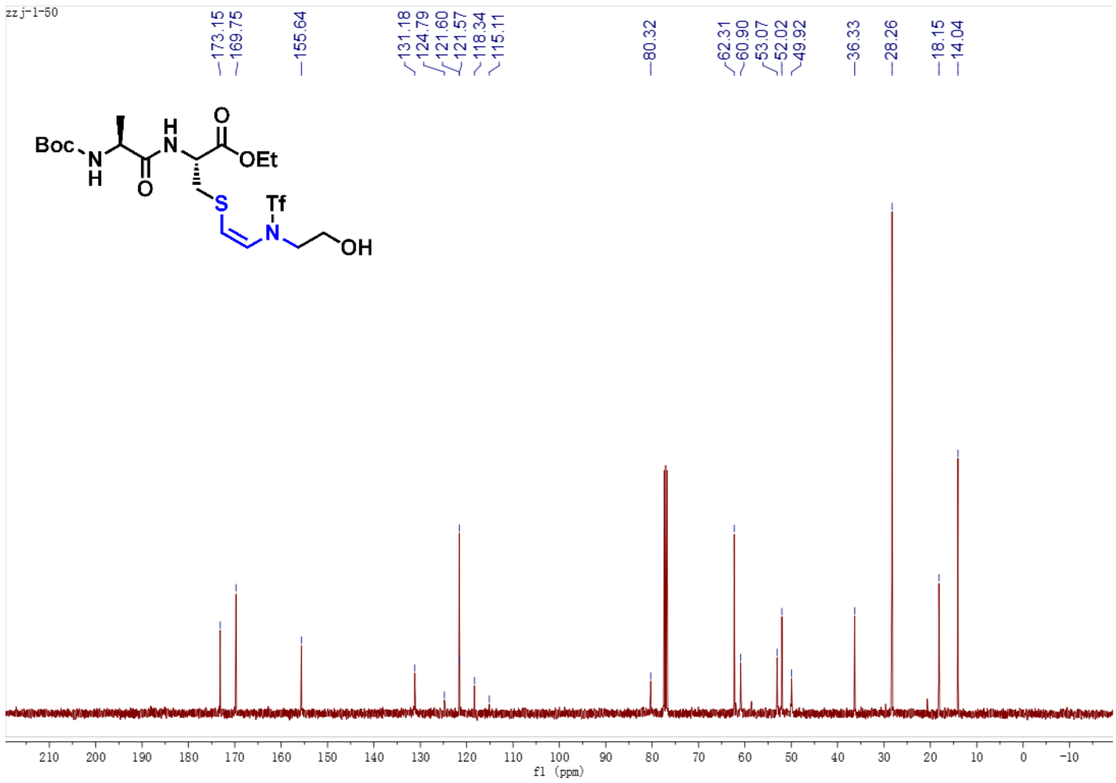

Figure S151. ^{13}C-NMR (100 MHz) spectrum of compound **3ae** in $CDCl_3$

NOESY, $CDCl_3$

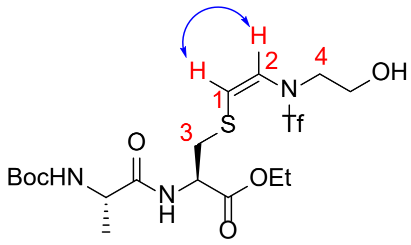

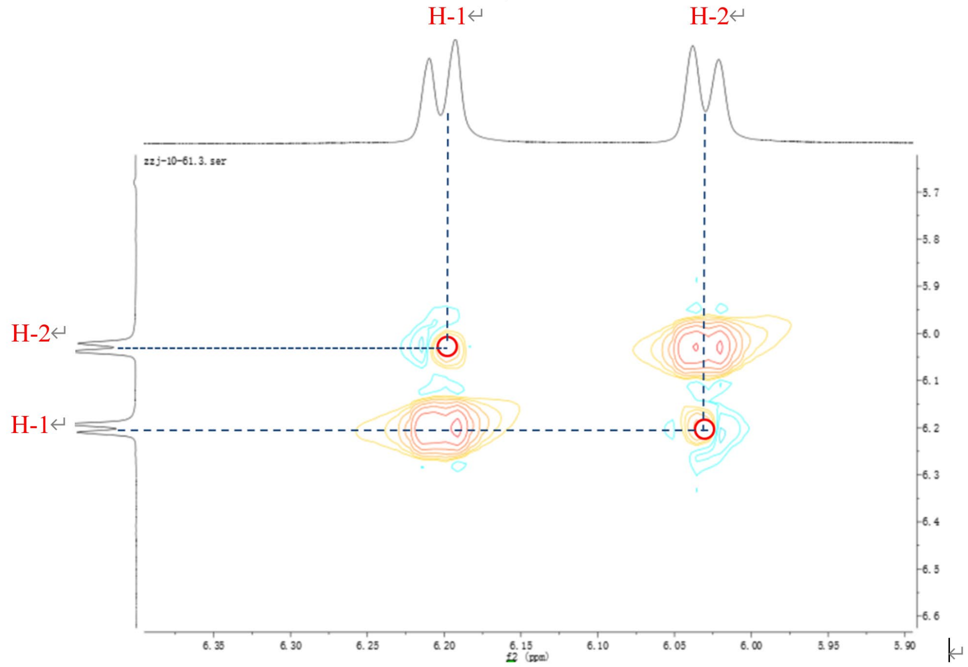

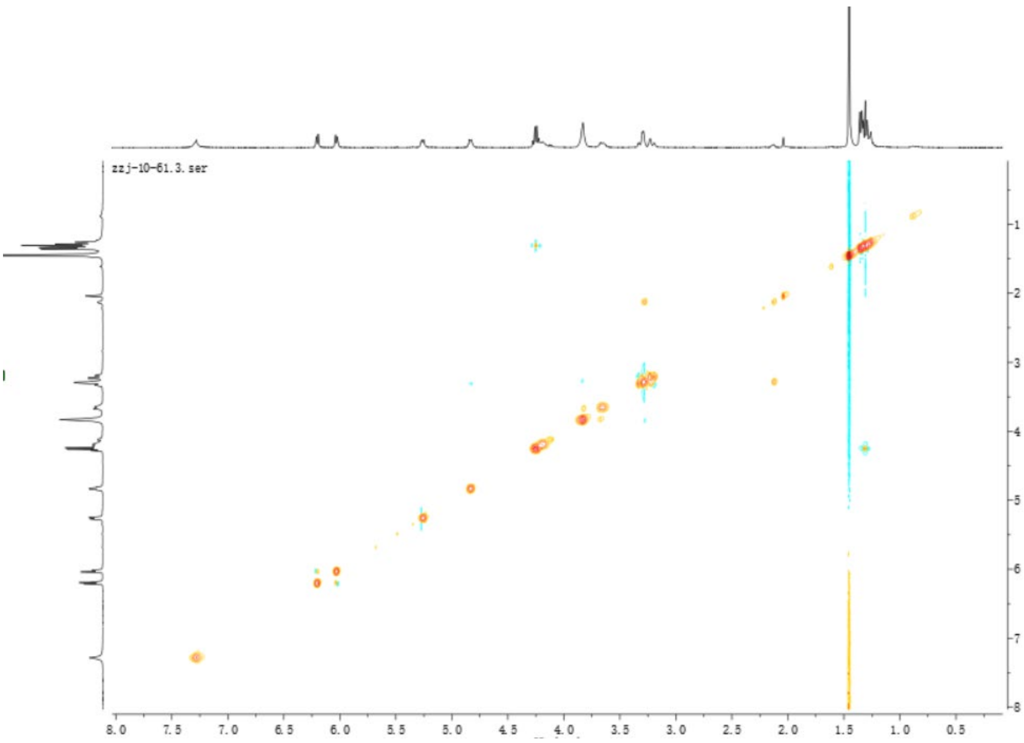

Figure S152. NOESY spectrum of compound **3ae** in $CDCl_3$

COSY, $CDCl_3$

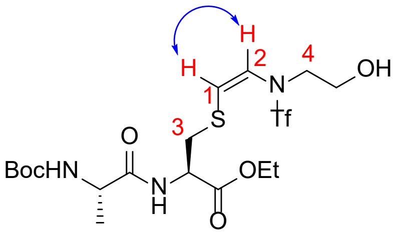

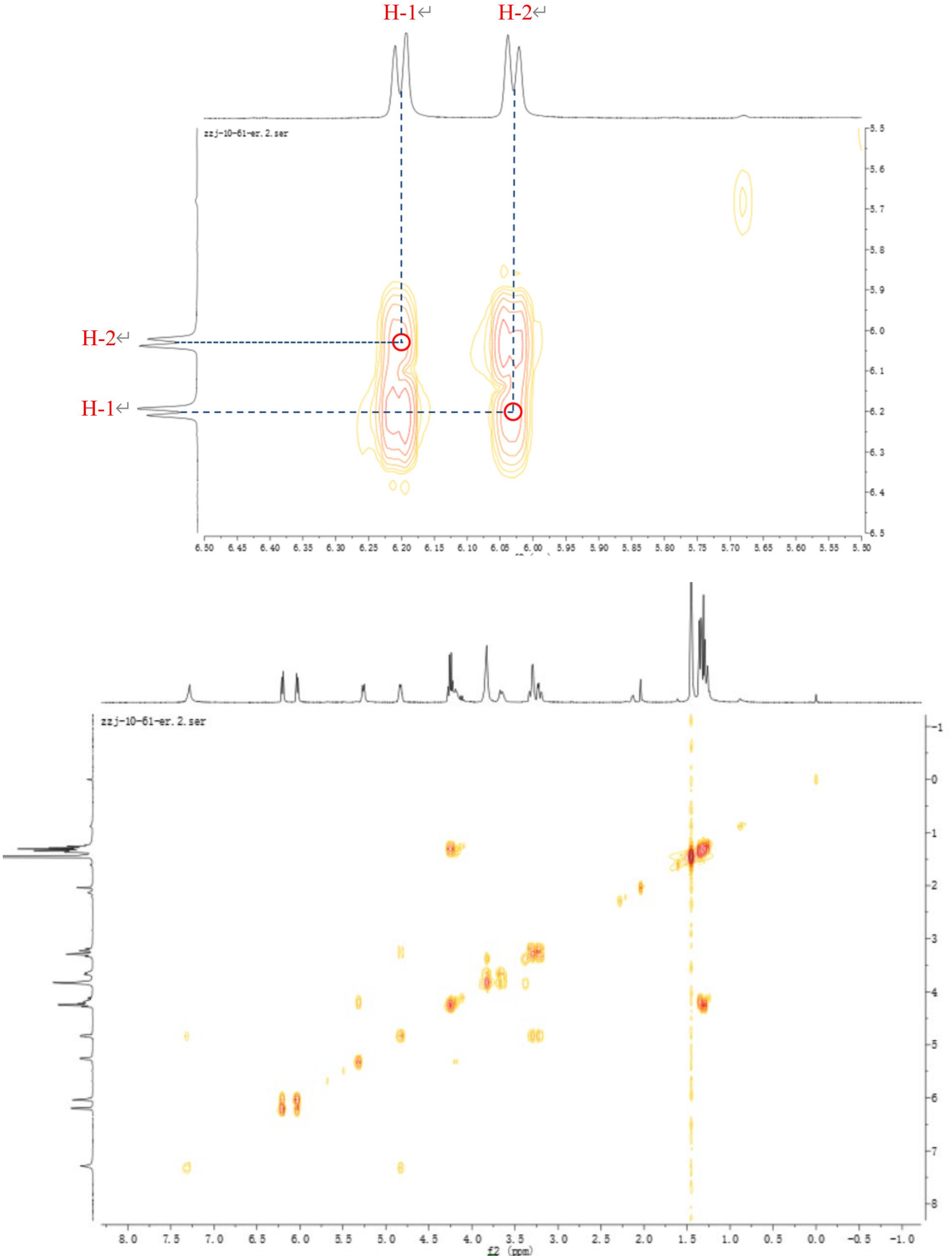

Figure S153. COSY spectrum of compound **3ae** in $CDCl_3$

HMBC, $CDCl_3$

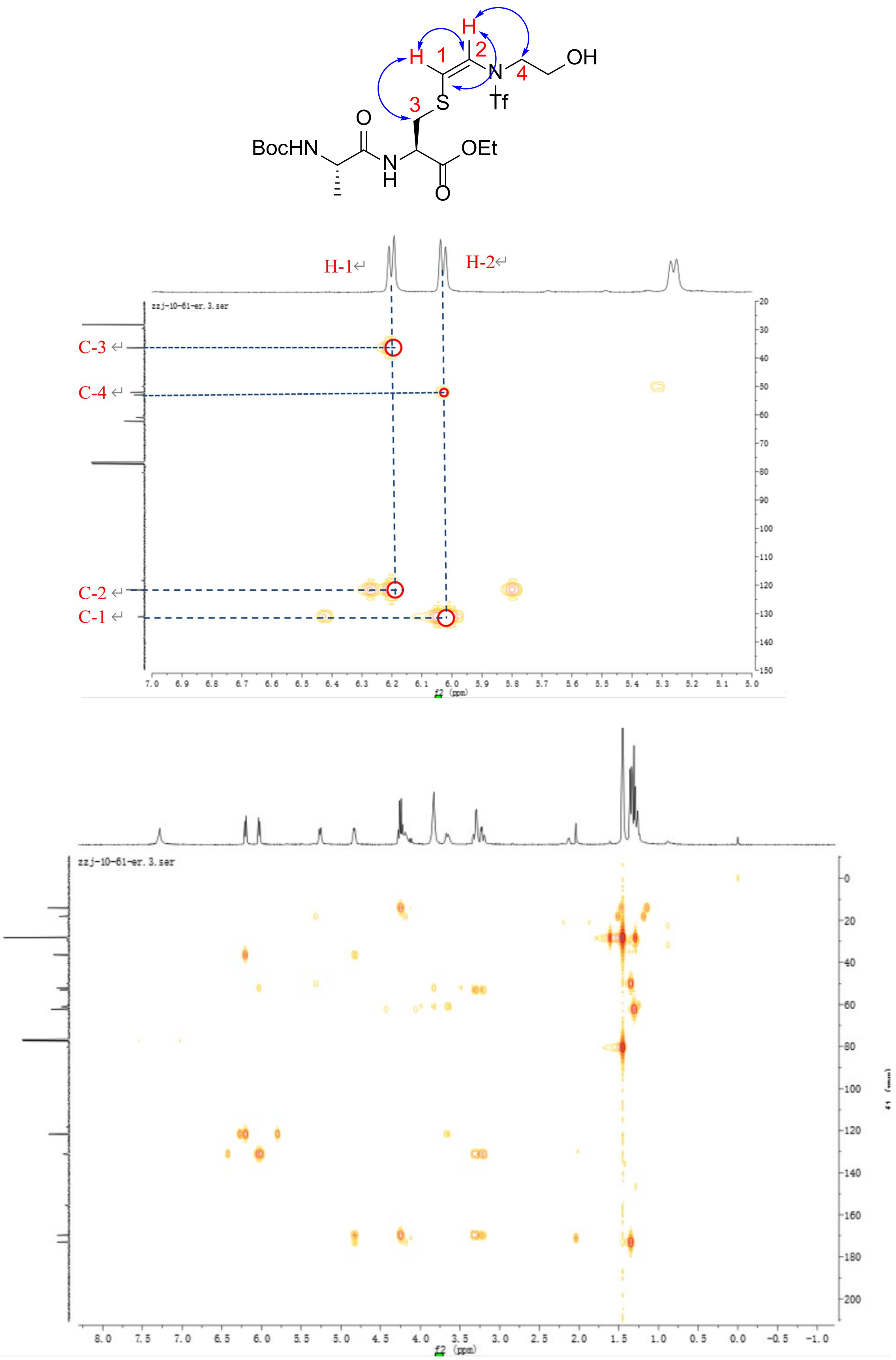

Figure S154. HMBC spectrum of compound **3ae** in $CDCl_3$

HSQC, $CDCl_3$

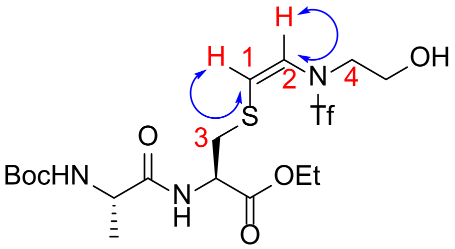

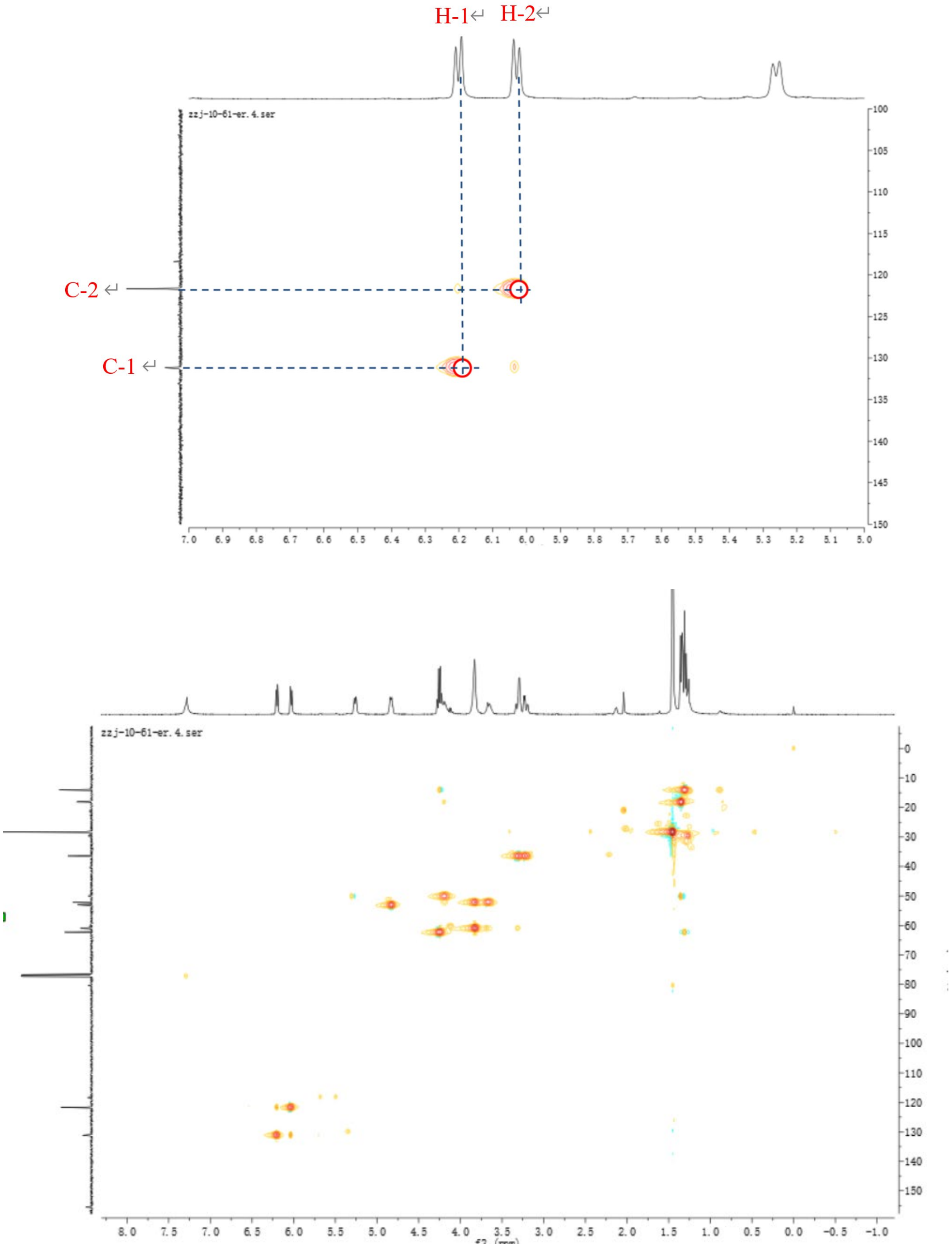

Figure S155. HSQC spectrum of compound **3ae** in $CDCl_3$

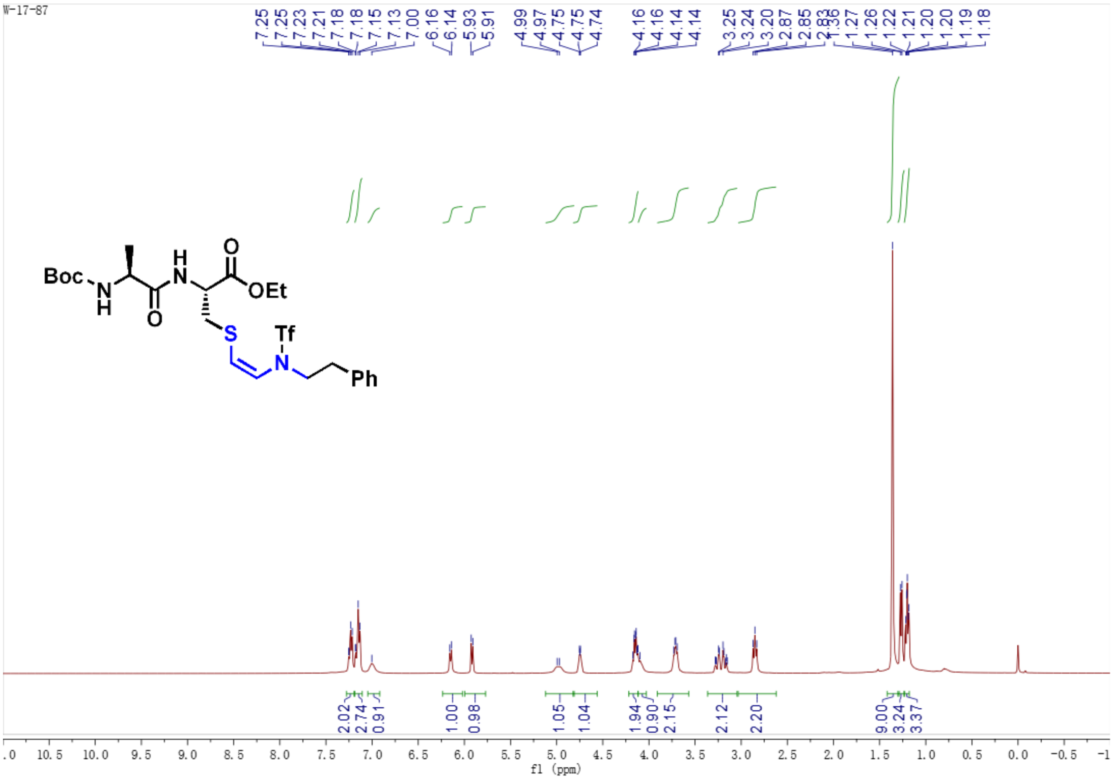

Figure S156. ^{1}H-NMR (400 MHz) spectrum of compound **3af** in $CDCl_3$

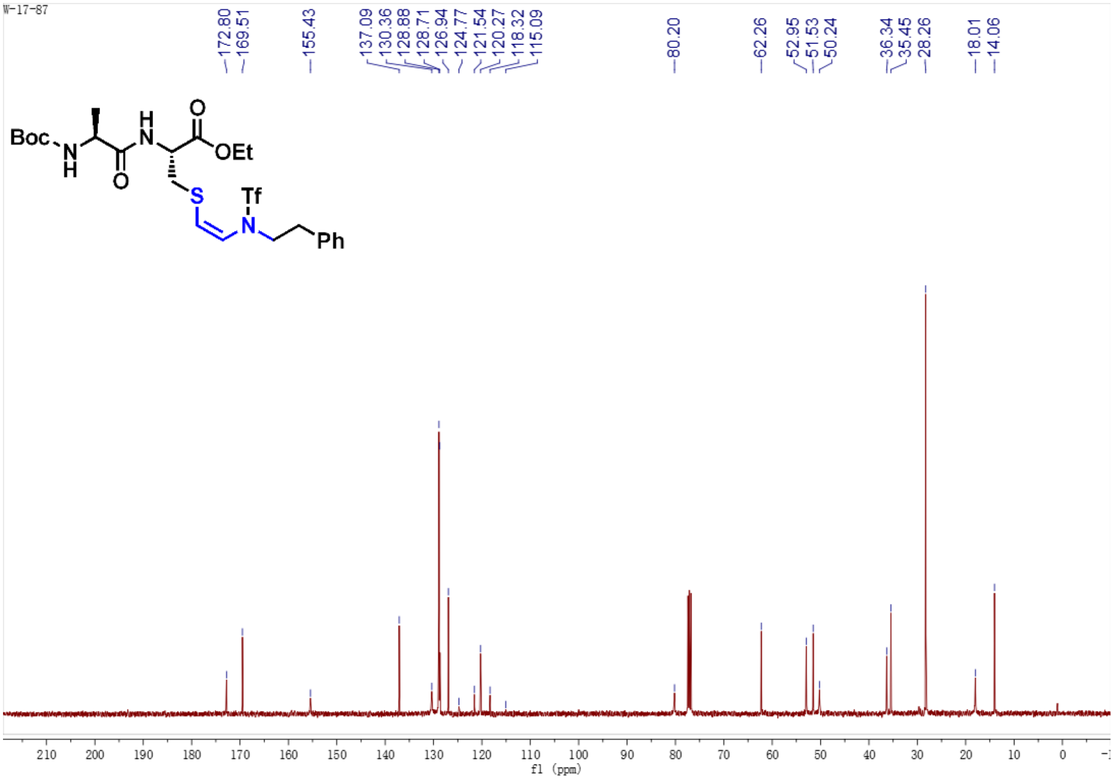

Figure S157. ^{13}C-NMR (100 MHz) spectrum of compound **3af** in $CDCl_3$

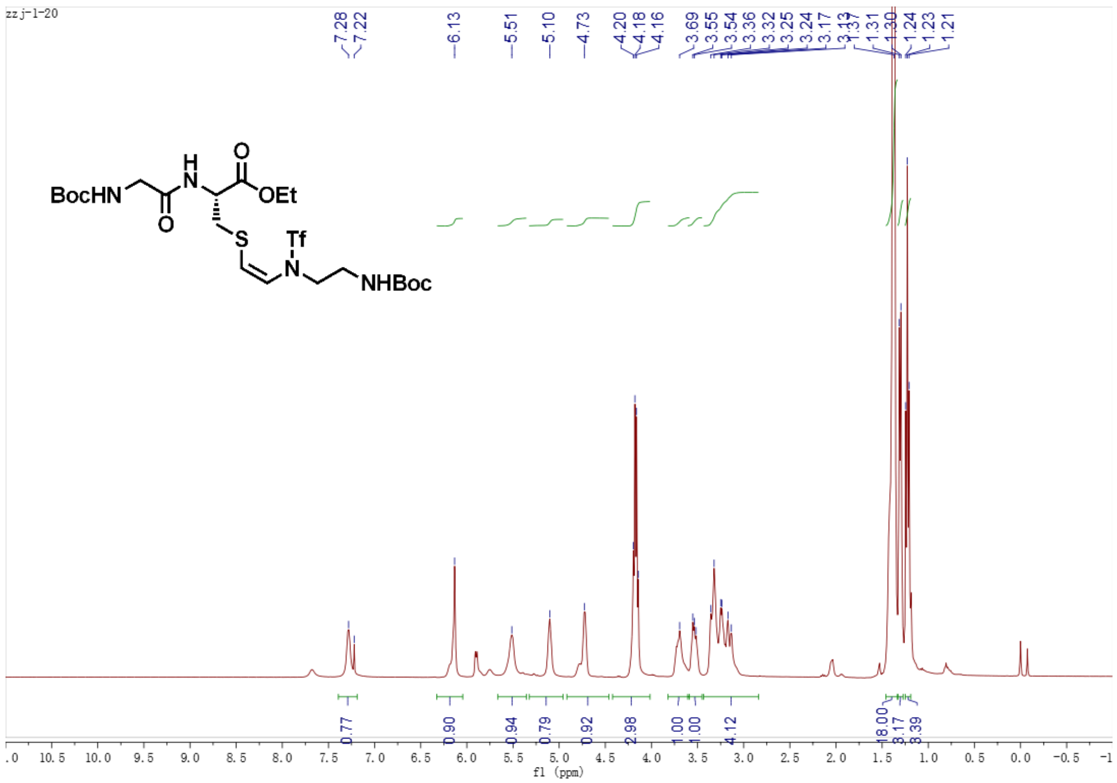

Figure S158. ^{1}H-NMR (400 MHz) spectrum of compound **3ag** in $CDCl_3$

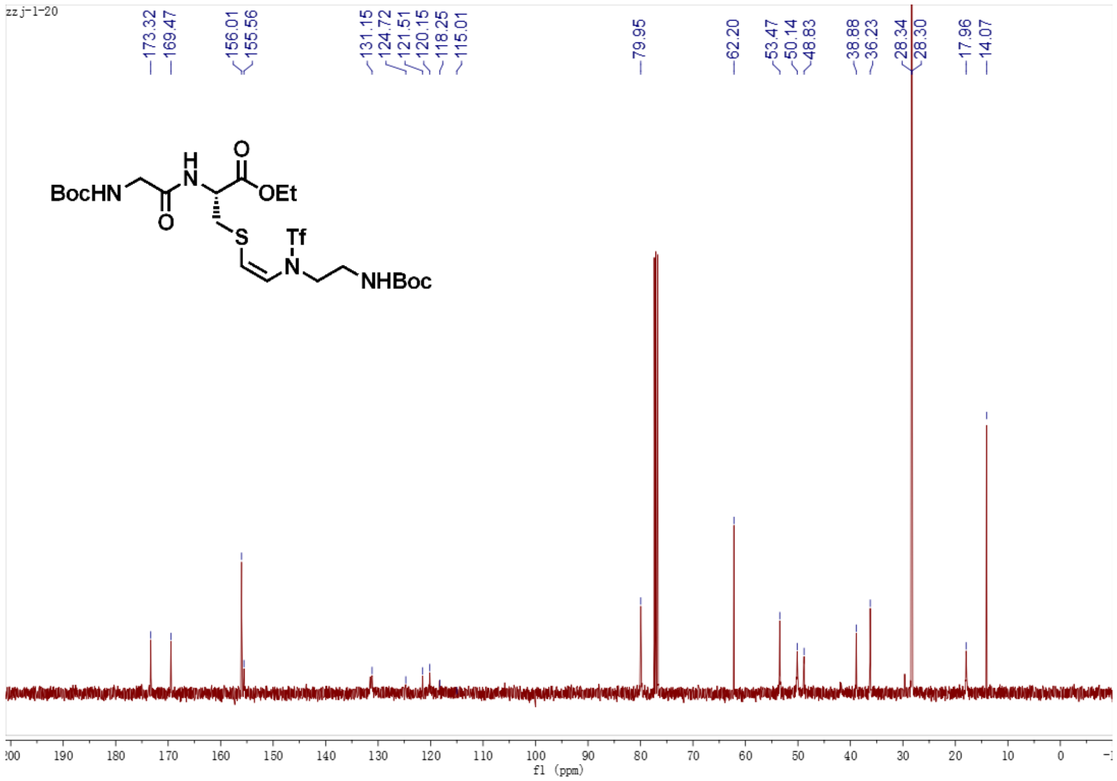

Figure S159. ^{13}C-NMR (100 MHz) spectrum of compound **3ag** in $CDCl_3$

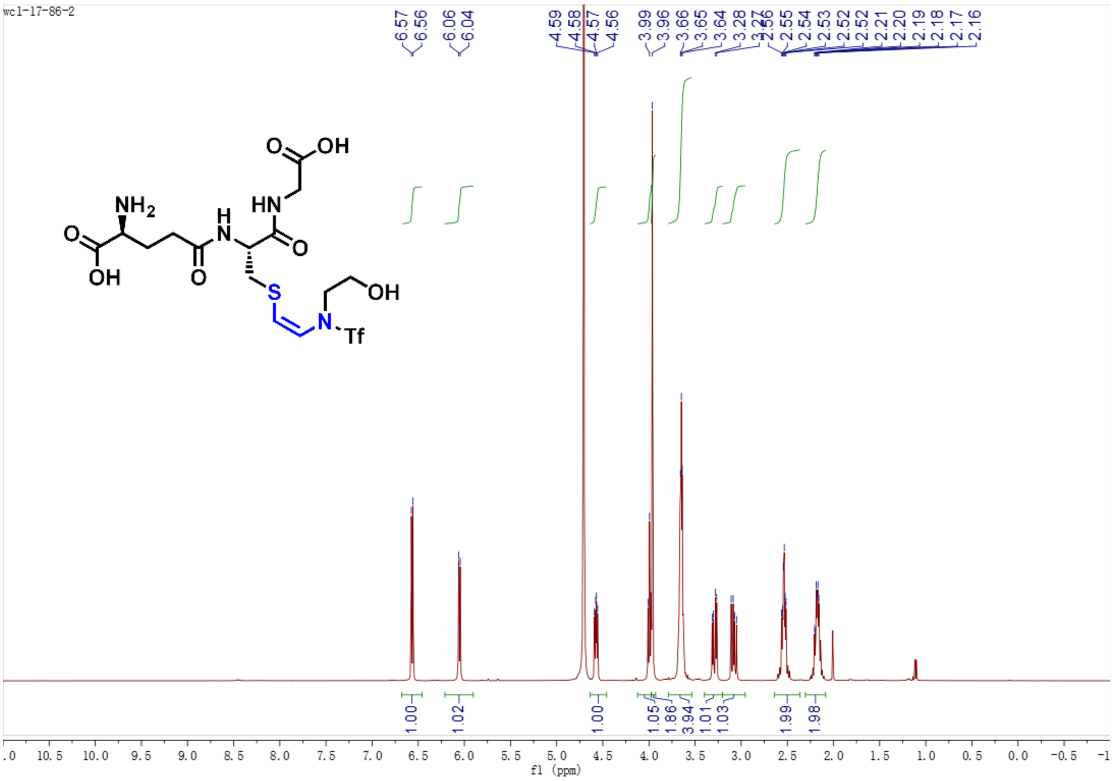

Figure S160. ^{1}H-NMR (400 MHz) spectrum of compound **3am** in D_2O

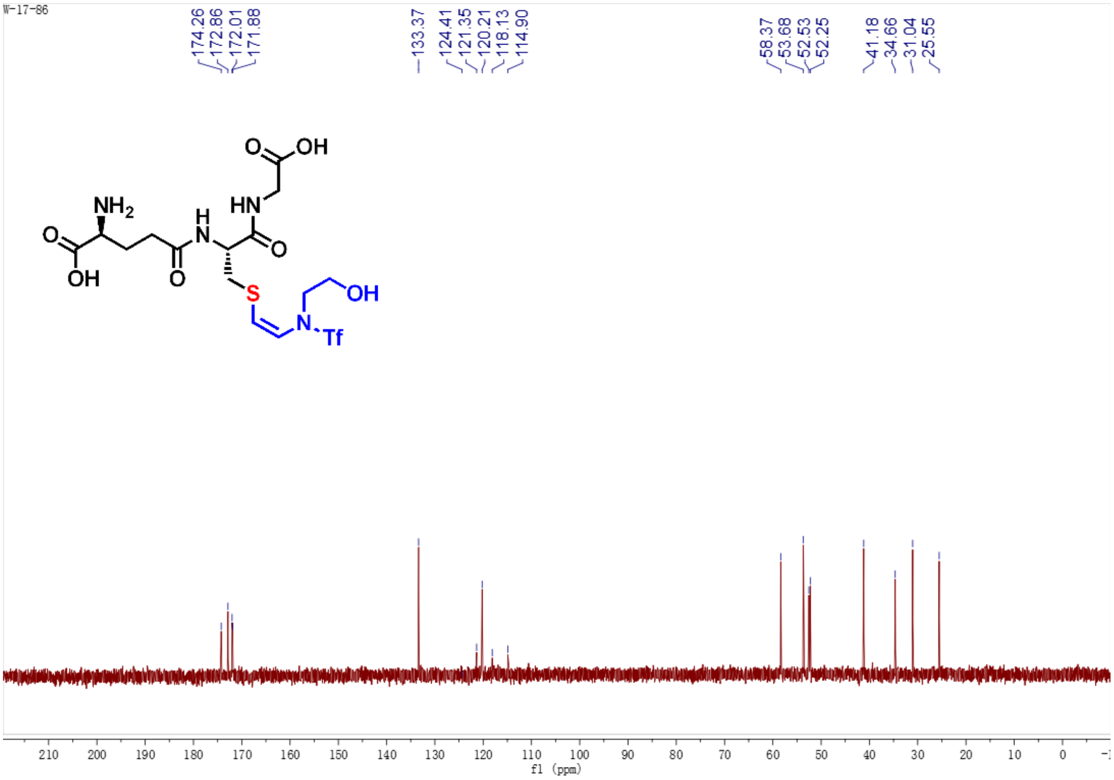

Figure S161. ^{13}C-NMR (100 MHz) spectrum of compound **3am** in D_2O

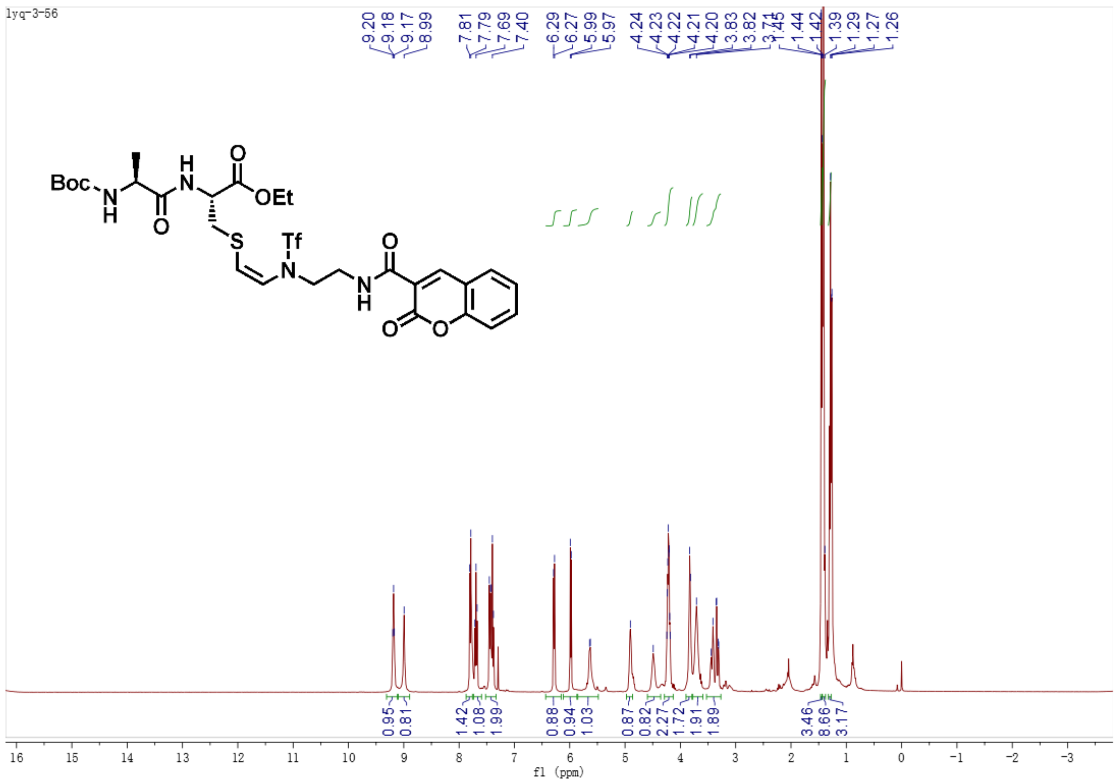

Figure S162. ^{1}H-NMR (400 MHz) spectrum of compound **4ah** in $CDCl_3$

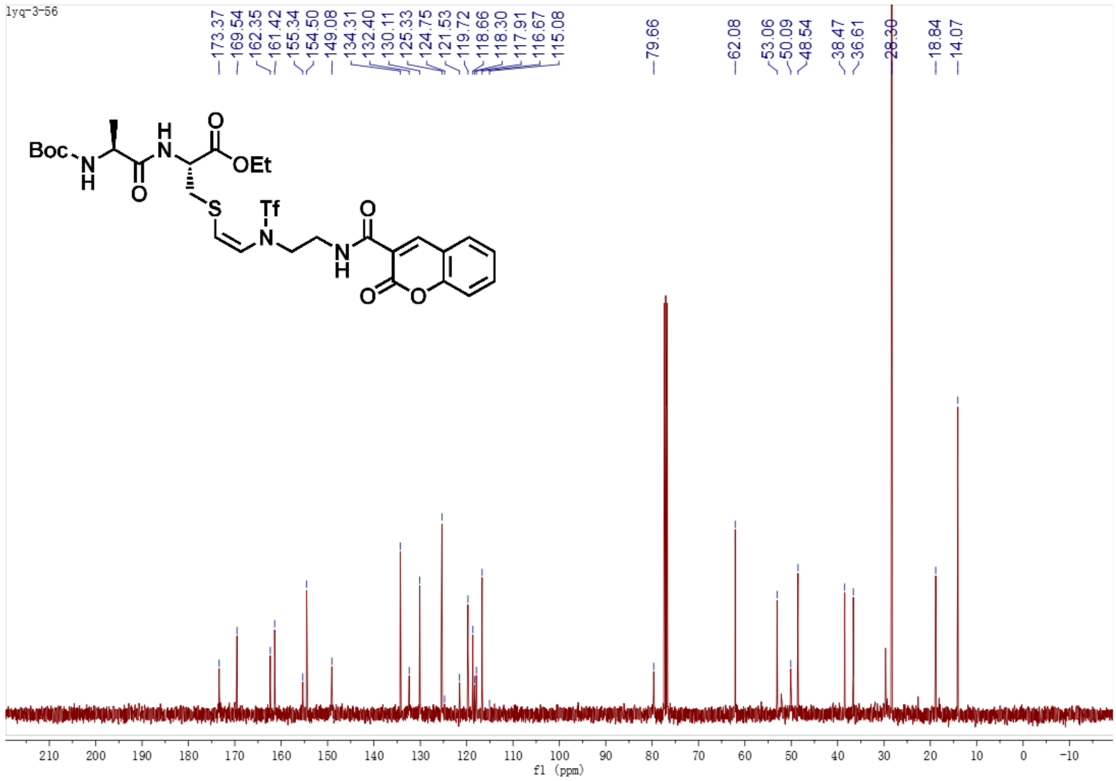

Figure S163. ^{13}C-NMR (100 MHz) spectrum of compound **4ah** in $CDCl_3$

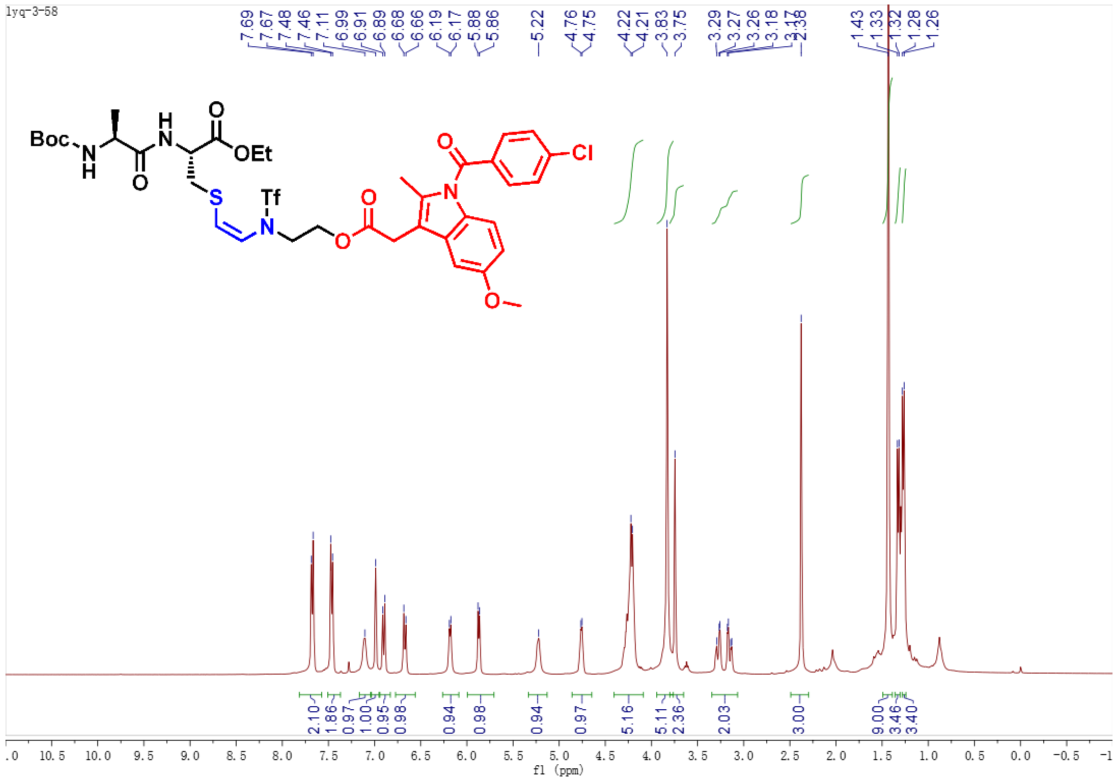

Figure S164. ^{1}H-NMR (400 MHz) spectrum of compound **4ai** in $CDCl_3$

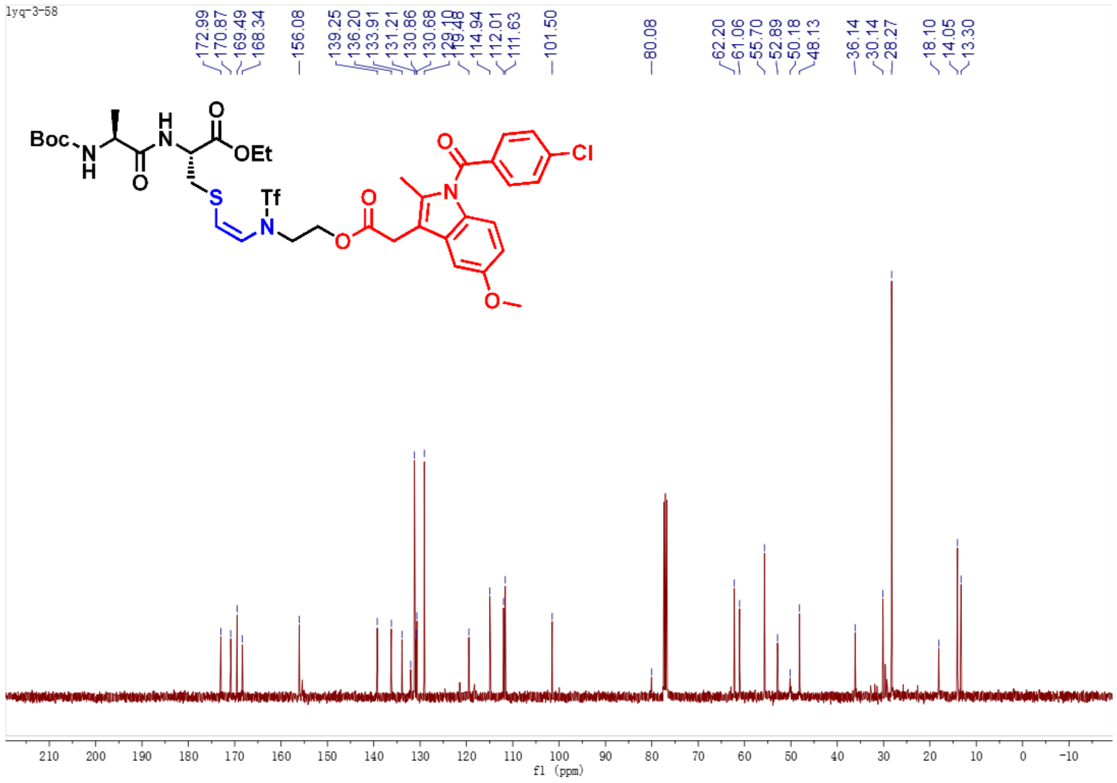

Figure S165. ^{13}C-NMR (100 MHz) spectrum of compound **4ai** in $CDCl_3$

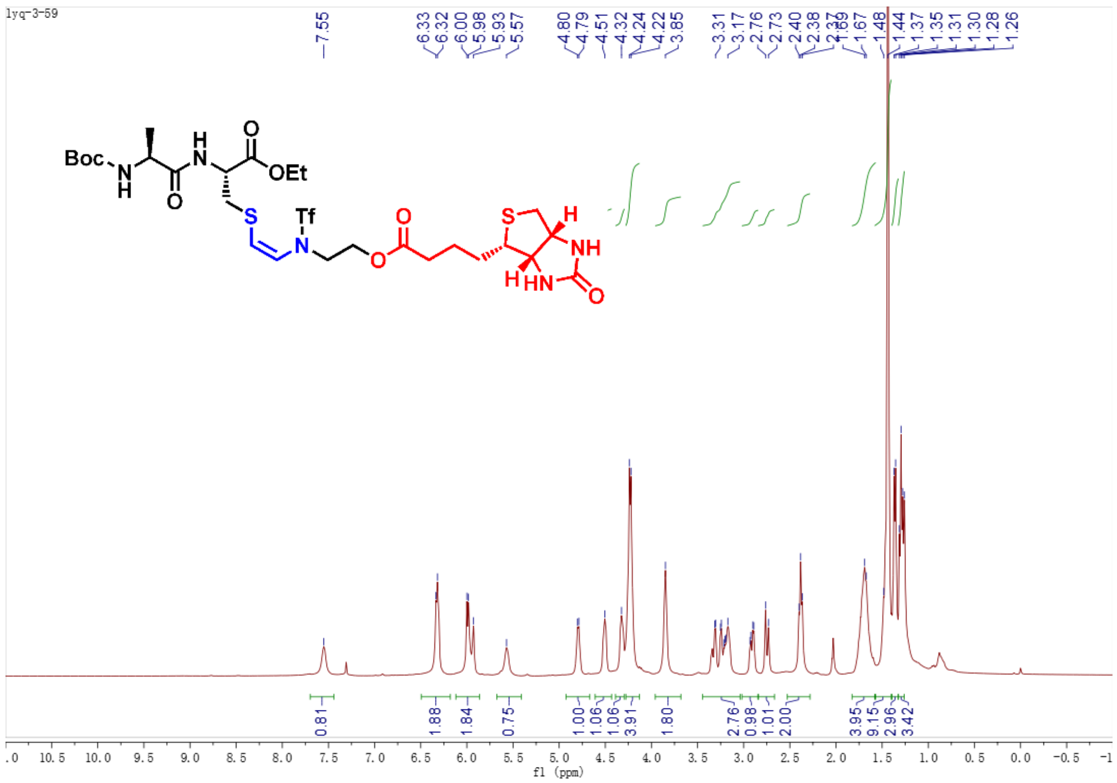

Figure S166. ^{1}H-NMR (400 MHz) spectrum of compound **4aj** in $CDCl_3$

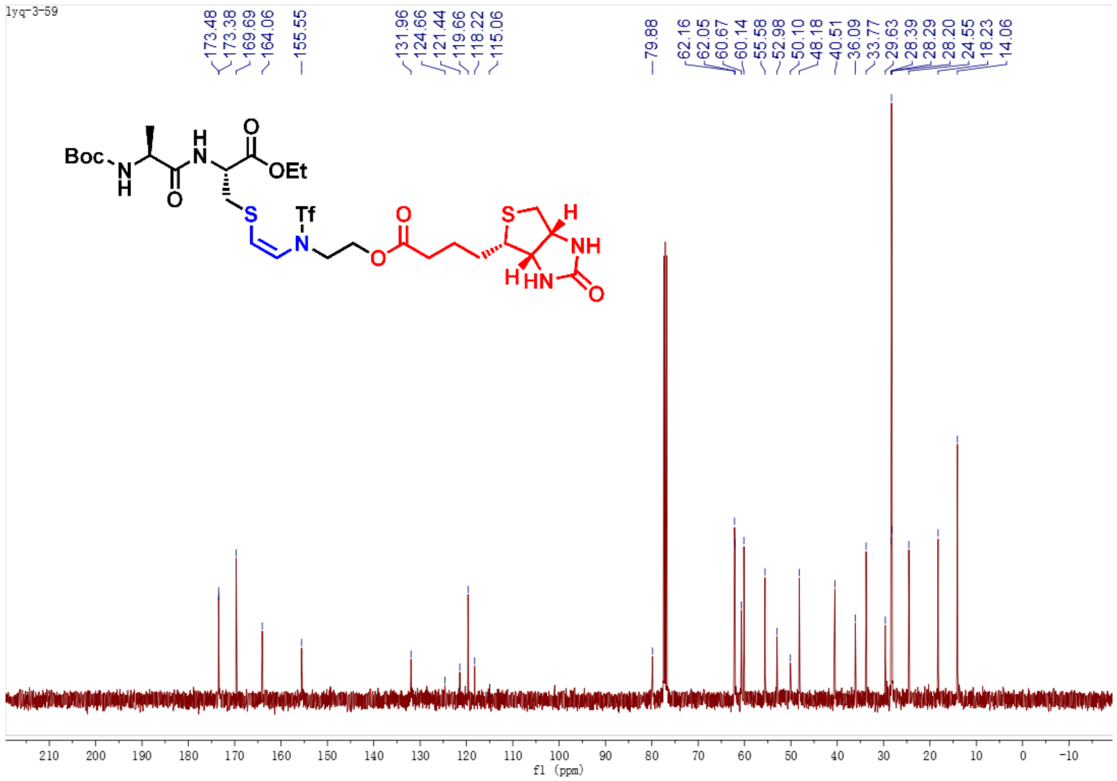

Figure S167. ^{13}C-NMR (100 MHz) spectrum of compound **4aj** in $CDCl_3$

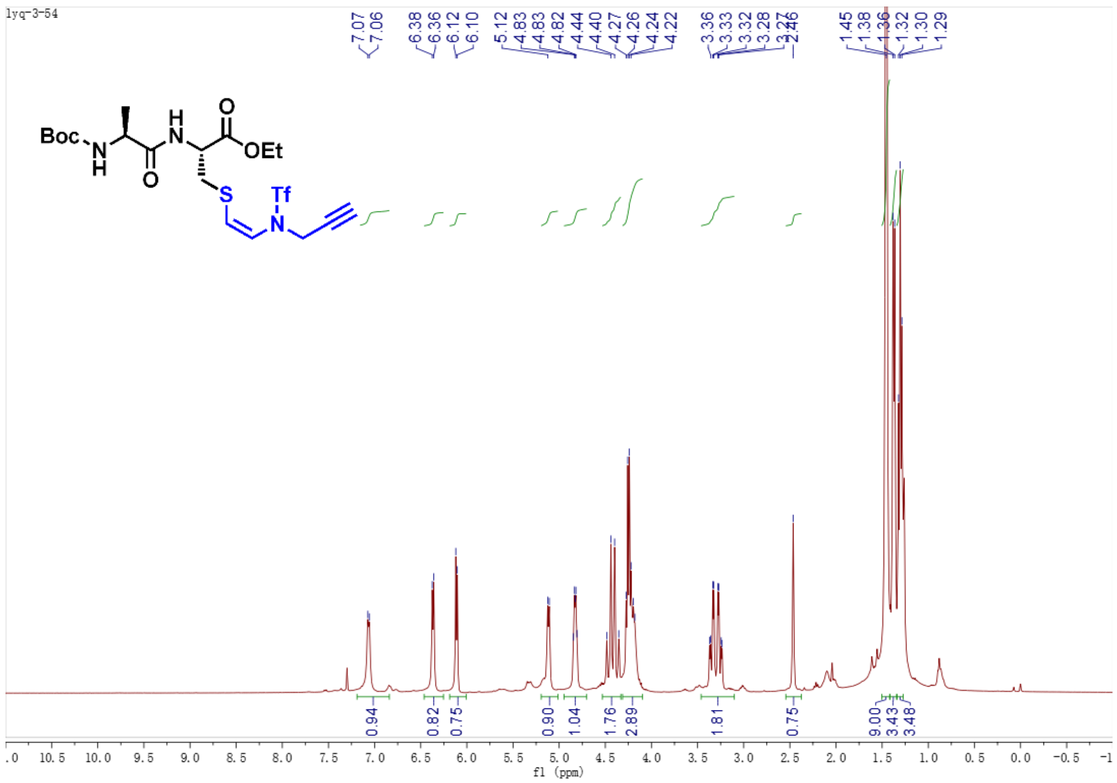

Figure S168. ^{1}H-NMR (400 MHz) spectrum of compound **4ak** in $CDCl_3$

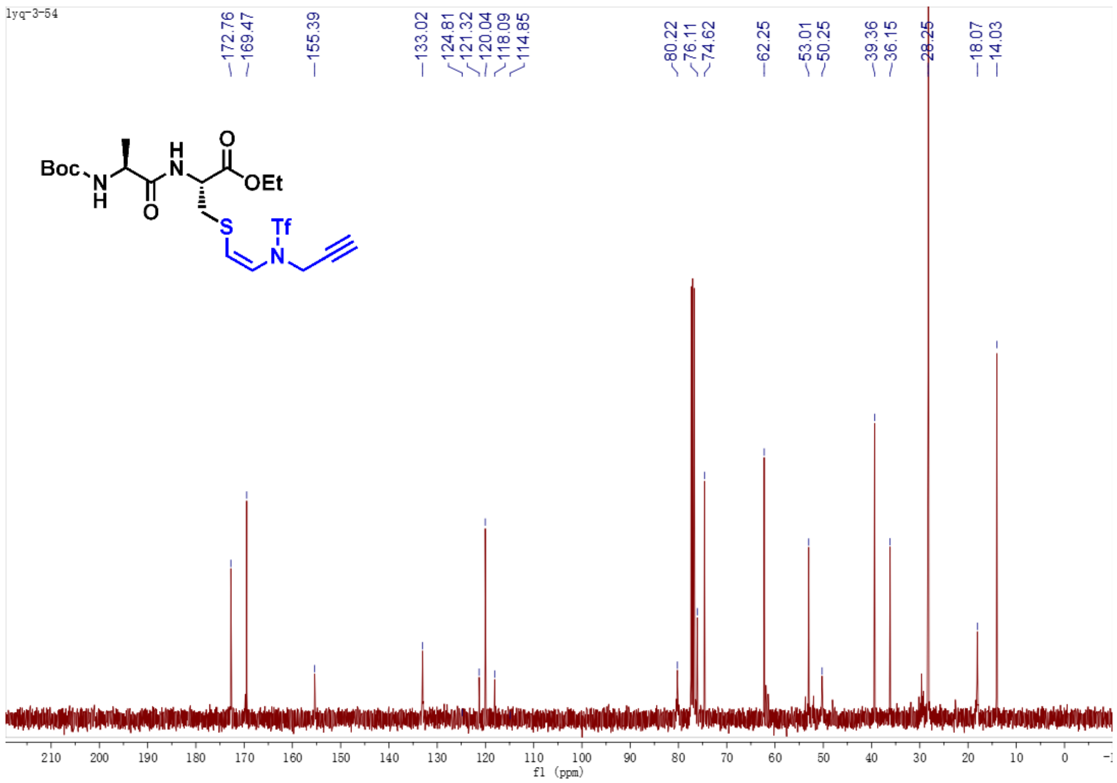

Figure S169. ^{13}C-NMR (100 MHz) spectrum of compound **4ak** in $CDCl_3$

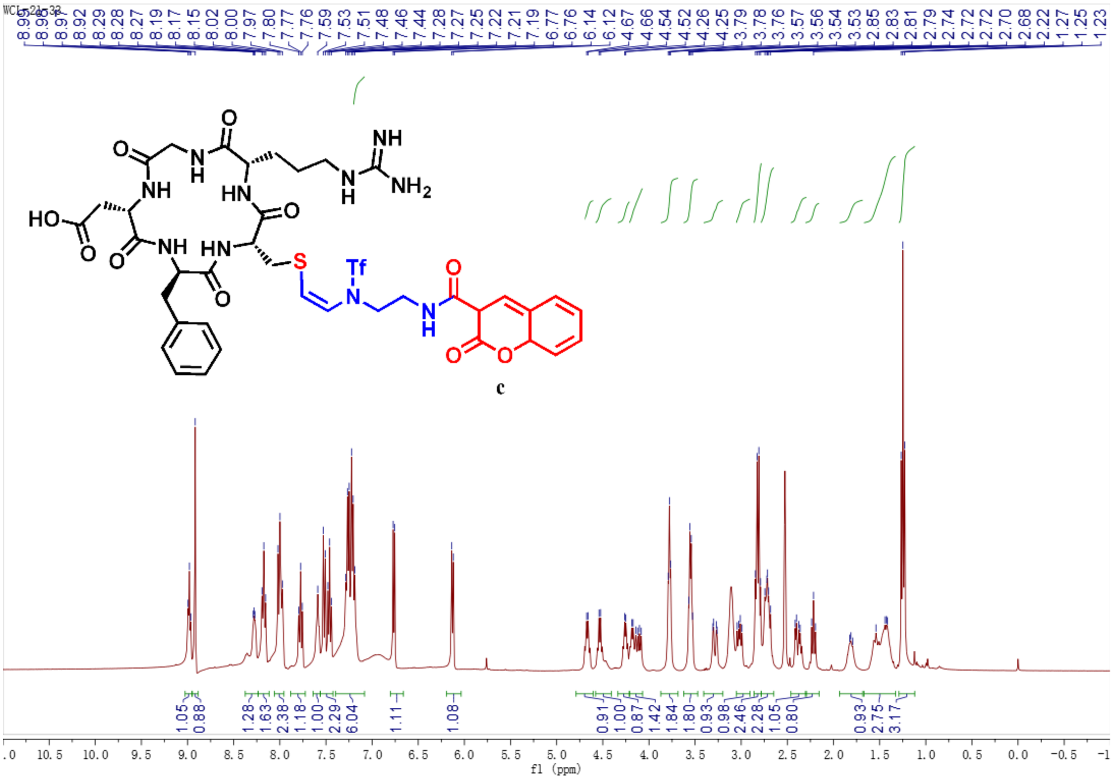

Figure S170. ^{1}H-NMR (400 MHz) spectrum of compound **4oh** in DMSO-d_6

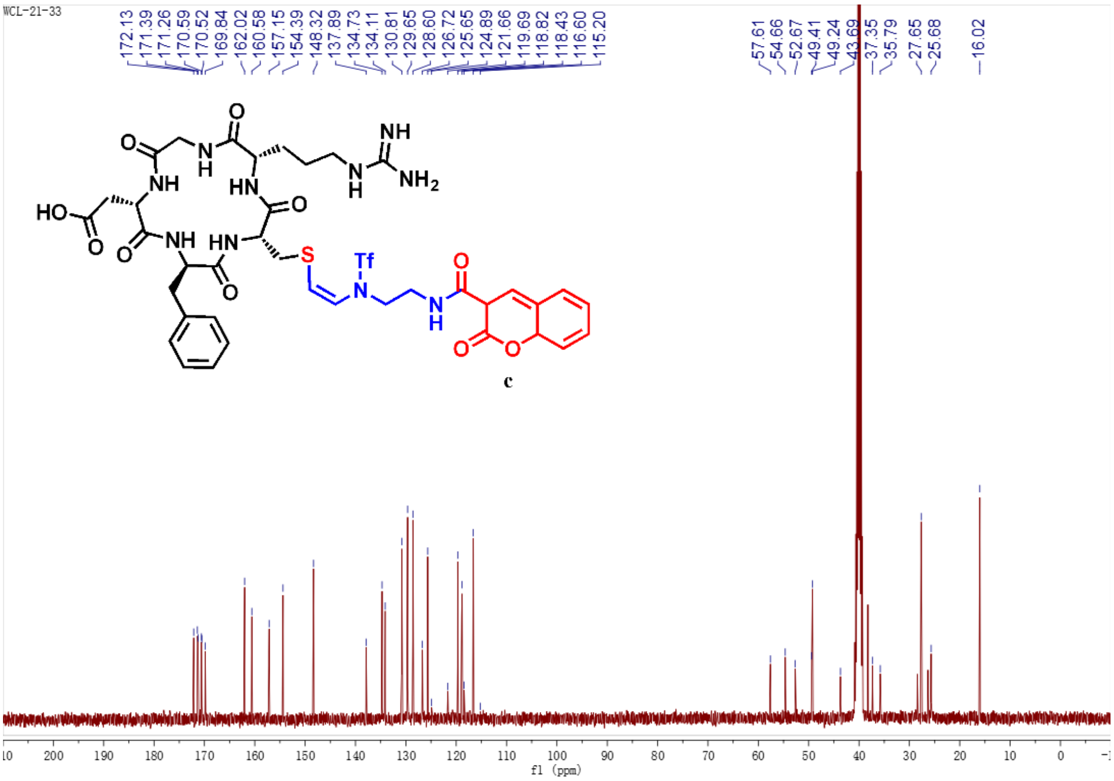

Figure S171. ^{13}C-NMR (100 MHz) spectrum of compound **4oh** in DMSO-d_6

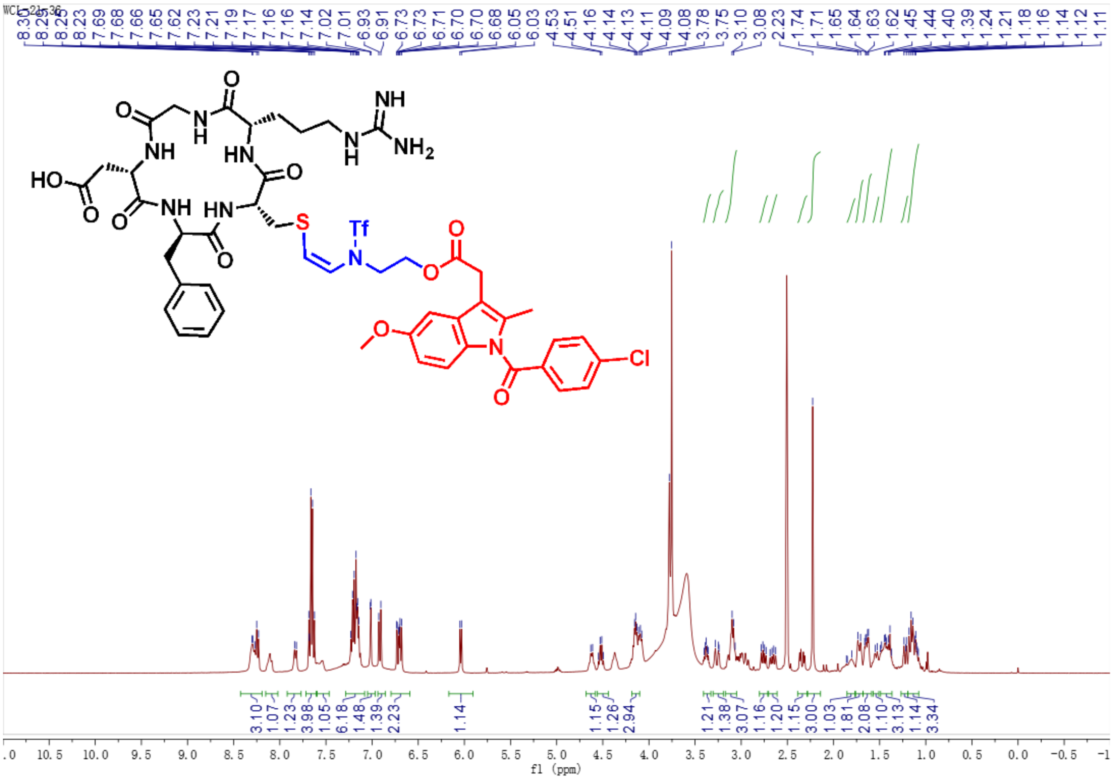

Figure S172. ^{1}H-NMR (400 MHz) spectrum of compound **4oi** in DMSO-d_6

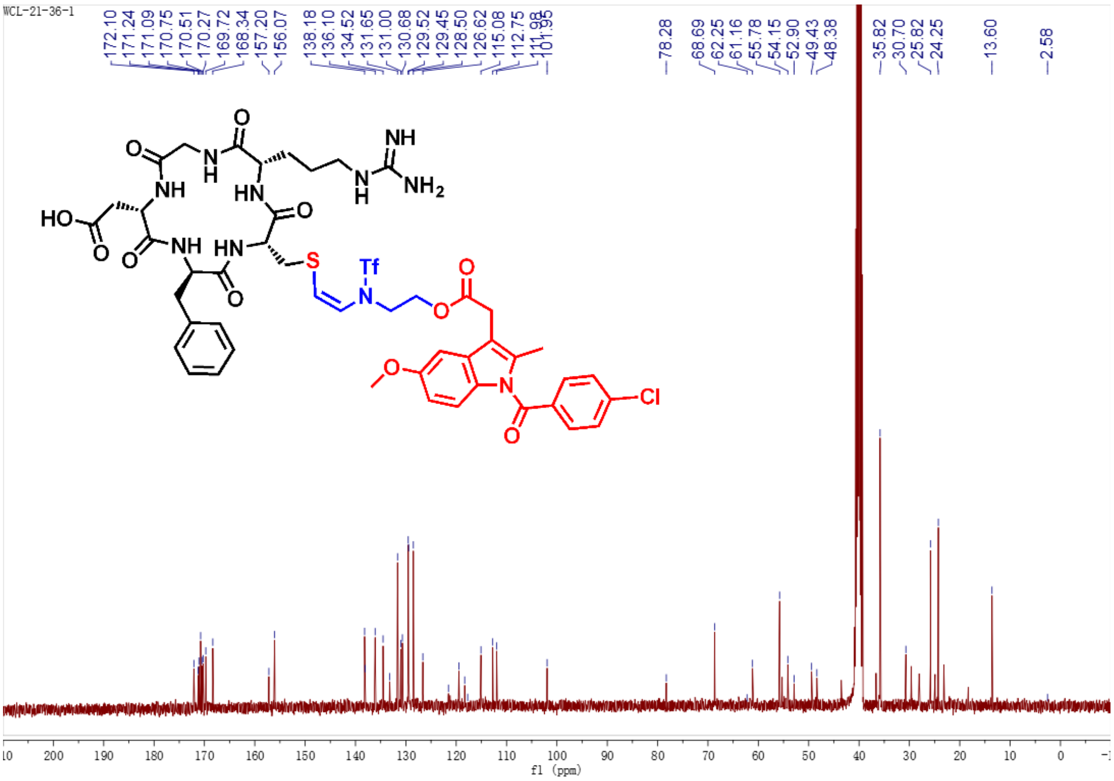

Figure S173. ^{13}C-NMR (100 MHz) spectrum of compound **4oi** in DMSO-d_6

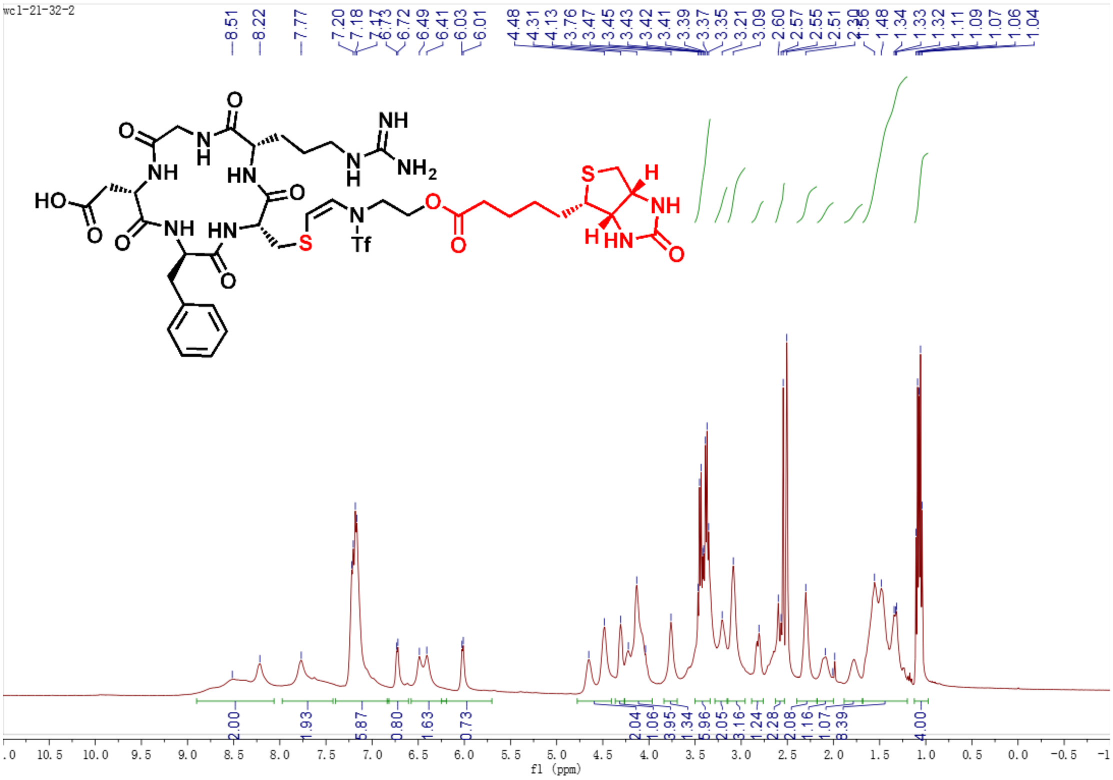

Figure S174. ^{1}H-NMR (400 MHz) spectrum of compound **4oj** in DMSO-d_6

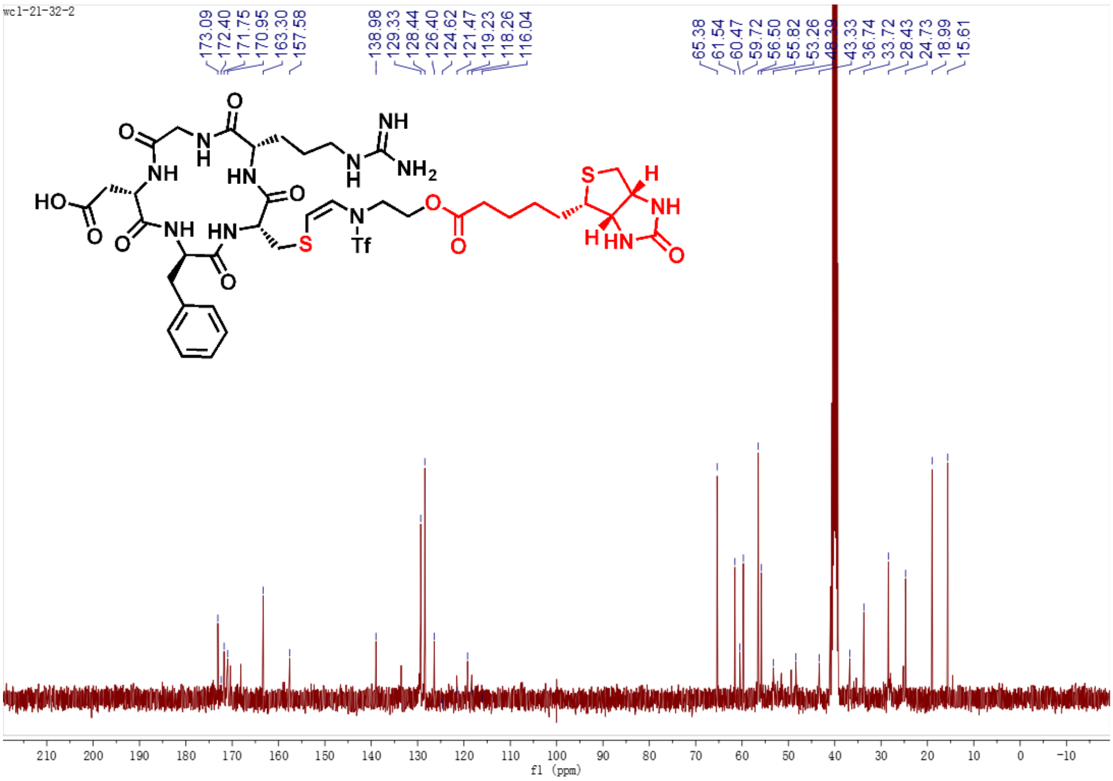

Figure S175. ^{13}C-NMR (100 MHz) spectrum of compound **4oj** in DMSO-d_6

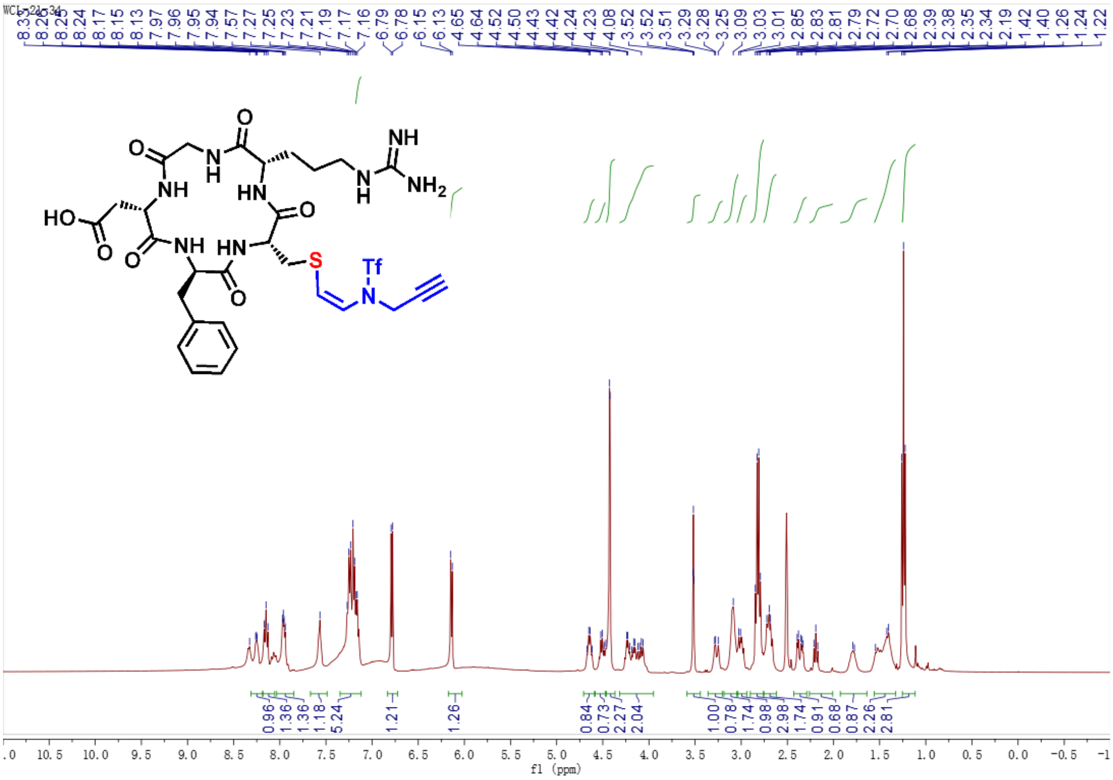

Figure S176. ^{1}H-NMR (400 MHz) spectrum of compound **4ok** in DMSO-d_6

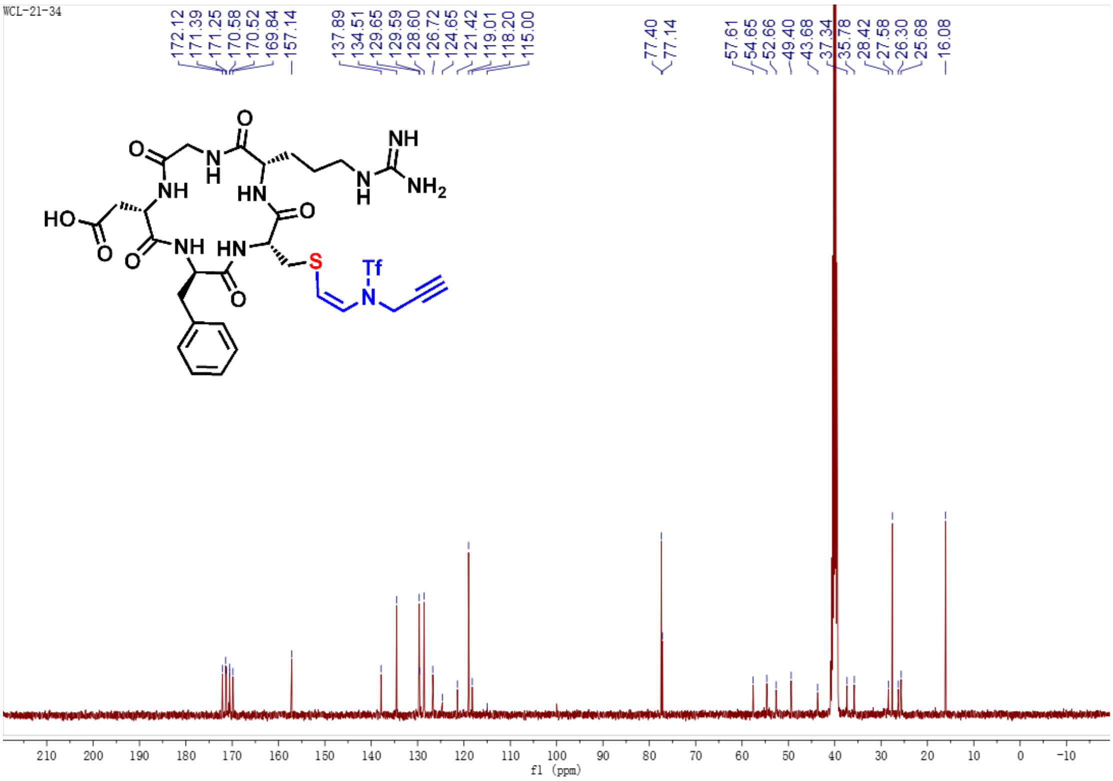

Figure S177. ^{13}C-NMR (100 MHz) spectrum of compound **4ok** in DMSO-d_6
